# The conserved two-component systems CutRS and CssRS control the protein secretion stress response in *Streptomyces*

**DOI:** 10.1128/mbio.02991-25

**Published:** 2025-12-15

**Authors:** Thomas C. McLean, Ainsley D. M. Beaton, Neil A. Holmes, Carlo Martins, Gerhard Saalbach, Govind Chandra, Sibyl F. D. Batey, Jana China, Barrie Wilkinson, Matthew I. Hutchings

**Affiliations:** 1 Department of Molecular Microbiology, John Innes Centre 534072 , Norwich, United Kingdom; 2 The Centre for Microbial Interactions, Norwich Research Park 455075, Norwich, United Kingdom; 3 Department of Biochemistry and Metabolism, Proteomics Facility, John Innes Centre, Norwich Research Park 15549, Norwich, United Kingdom; Freie Universitat Berlin, Berlin, Germany

**Keywords:** secretion stress, *Streptomyces*, gene regulation, secondary metabolism

## Abstract

*Streptomyces* specialized metabolites account for over half of all clinically used antibiotics, as well as numerous antifungal, anticancer, and immunosuppressant agents. Two-component systems, which are widespread in bacteria, are key regulators of antibiotic production in *Streptomyces* species, yet their activating signals remain poorly understood. CutRS was the first two-component system identified in the genus *Streptomyces,* and deletion of *cutRS* in *Streptomyces coelicolor* was shown to enhance antibiotic production, although its CutR regulon does not include biosynthetic genes. Here, we used *Streptomyces venezuelae* NRRL B-65442 to further investigate CutRS function. We show that deletion of *cutRS* increases growth rate and a reversal of the glucose-mediated carbon catabolite repression typically observed in *Streptomyces* species. We also demonstrate that CutR DNA binding is glucose-dependent, but CutR does not directly regulate genes involved in growth, antibiotic biosynthesis, or glucose metabolism. The only CutR targets conserved in both *S. coelicolor* and *S. venezuelae* are the foldase genes *htrA3* and *htrB*, which are involved in the protein secretion stress response. Consistent with this, we show that CutS homologs all contain two conserved cysteine residues in their extracellular sensor domains and that changing these residues to serine constitutively activates *S. venezuelae* CutRS. We propose that failure of a disulfide bond to form between these cysteine residues indicates secretion stress and leads to activation of the CutRS system and the secretion stress response.

**IMPORTANCE:**

*Streptomyces* bacteria are the primary source of clinically useful antibiotics. While many two-component systems have been linked to antibiotic biosynthesis in *Streptomyces* species, few have been well characterized. Here, we characterize a secretion stress-sensing two-component system called CutRS and propose a model for how the sensor kinase detects extracellular protein misfolding via two highly conserved cysteine residues. Importantly, we also show that deletion of *cutRS* triggers antibiotic overproduction in the presence of glucose. Since glucose normally represses antibiotic biosynthesis in *Streptomyces* species through carbon catabolite repression, this finding reveals a simple genetic route to bypass this barrier. This has significant implications for antibiotic discovery pipelines and industrial production, where glucose-rich media are preferred for cost and scalability. Our results position CutRS as a key target for future strain-improvement strategies.

## INTRODUCTION

*Streptomyces* species are studied for their complex life cycles and prolific production of specialized metabolites, including many clinically important antibiotics (1). These metabolites are typically encoded by biosynthetic gene clusters (BGCs), but despite the wealth of genomic data available, only ~3% of BGCs have been linked to their corresponding natural products (2). The vast majority remain cryptic under standard laboratory conditions, representing untapped reservoirs for novel bioactive compounds (3, 4). Unlocking these cryptic BGCs requires a better understanding of the regulatory mechanisms, including signal transduction pathways, that control specialized metabolism.

BGC expression is often regulated by transcription factors encoded within the cluster itself (cluster-situated regulators), as well as by global regulatory systems that integrate signals related to nutrient availability, growth phase, and environmental stress to specialized metabolite production (5). Among these global regulators, two-component systems (TCSs) are particularly important. TCSs are widespread in bacteria, fungi, and plants, and many have been shown to influence antibiotic production in *Streptomyces* species. However, the mechanisms by which they exert this control are often poorly understood (6–8).

TCSs typically consist of a membrane-bound sensor kinase (SK) and a cognate response regulator (RR). Upon detection of an external signal, the SK autophosphorylates and transfers the phosphate to the RR, which then dimerizes and binds DNA to modulate the transcription of target genes. *Streptomyces* genomes encode between 50 and 100 TCSs, yet only 15 are highly conserved across species (9). Of these, several have been shown to impact antibiotic biosynthesis, but only a small number have been functionally characterized, including their target regulons.

CutRS was the first TCS identified in the genus *Streptomyces* (10), and its disruption in *S. lividans* and *S. coelicolor* resulted in overproduction of the antibiotic actinorhodin (10). However, a subsequent study in *S. coelicolor* revealed that none of the 88 genes directly regulated by CutR were involved in actinorhodin biosynthesis (11). Instead, the CutRS system was implicated in the protein secretion stress response, with CutR activating *htrA3* and repressing *htrB*, both of which encode high-temperature requirement (Htr) chaperone-proteases that assist in folding or degradation of misfolded proteins in the extracellular space (11).

Here, we investigated the CutRS system in *Streptomyces venezuelae*, a genetically tractable and rapidly growing model organism that has emerged as a powerful system for studying *Streptomyces* development and regulation (12). We found that *S. venezuelae* CutR binds nine promoter regions, but the core CutR regulon shared with *S. coelicolor* includes only *htrA3* and *htrB*, reinforcing a conserved role in the protein secretion stress response. Alignment of >100 CutS homologs across *Streptomyces* species revealed two invariant cysteine residues as the only conserved features in their predicted extracellular sensor domains. We demonstrate that changing these cysteines to serine constitutively activates CutRS, suggesting that the system senses protein misfolding via disulfide bond status outside the cell.

Although CutRS is only conserved in *Streptomyces* species, analysis of SK sequences from 12,799 bacterial genomes revealed that 99% encode at least one SK with two or more cysteines in their predicted extracellular sensor domains. We therefore propose that extracellular redox sensing could be a widespread signaling mechanism across the bacterial domain.

## RESULTS

### Deletion of *cutRS* affects growth and antibiotic production in *S. venezuelae*

**Fig 1:**
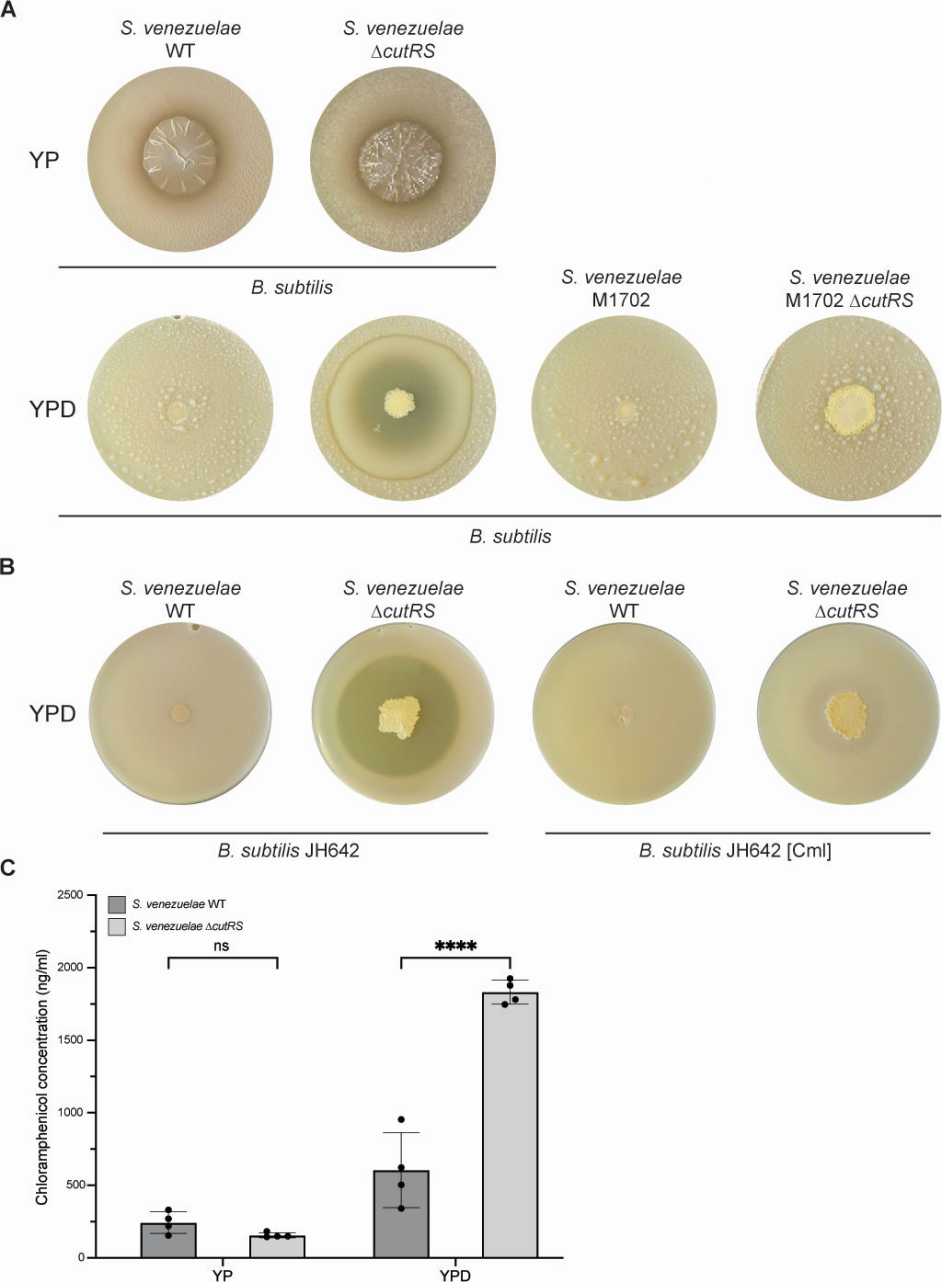
Deletion of *cutRS* results in the overproduction of chloramphenicol. (**A**) Antibacterial overlay assays in which colonies of the *S. venezuelae* wild-type (WT), Δ*cutRS*, M1702 (Δ*cml*Δ*jad*), and M1702 Δ*cutRS* strains (grown for 5 days) are overlaid with soft agar containing the indicator strain *Bacillus subtilis*. M1702 cannot make the antibiotics chloramphenicol or jadomycin, suggesting that the zone of inhibition in the Δ*cutRS* mutant growing on YPD agar is due to the production of one of these antibiotics. (**B**) Left: overlays of *S. venezuelae* wild-type and Δ*cutRS* strains with the chloramphenicol-sensitive strain *B. subtilis* JH642 on YPD agar. Right: overlays of *S. venezuelae* wild-type and Δ*cutRS* strains with the chloramphenicol-resistant strain *B. subtilis* JH642 on YPD agar. These experiments show that the Δ*cutRS* mutant is overproducing chloramphenicol on YPD agar but not on YP agar. (**C**) HPLC quantification of chloramphenicol extracted from *S. venezuelae* wild-type and Δ*cutRS* strains grown on YP and YPD agar. These data confirm that the Δ*cutRS* mutant produces more chloramphenicol relative to the wild-type, which may be in part due to the fact that it produces more biomass. ****Two-way ANOVA, *P*-value <0.0001. ns, not significant.

**Fig 2:**
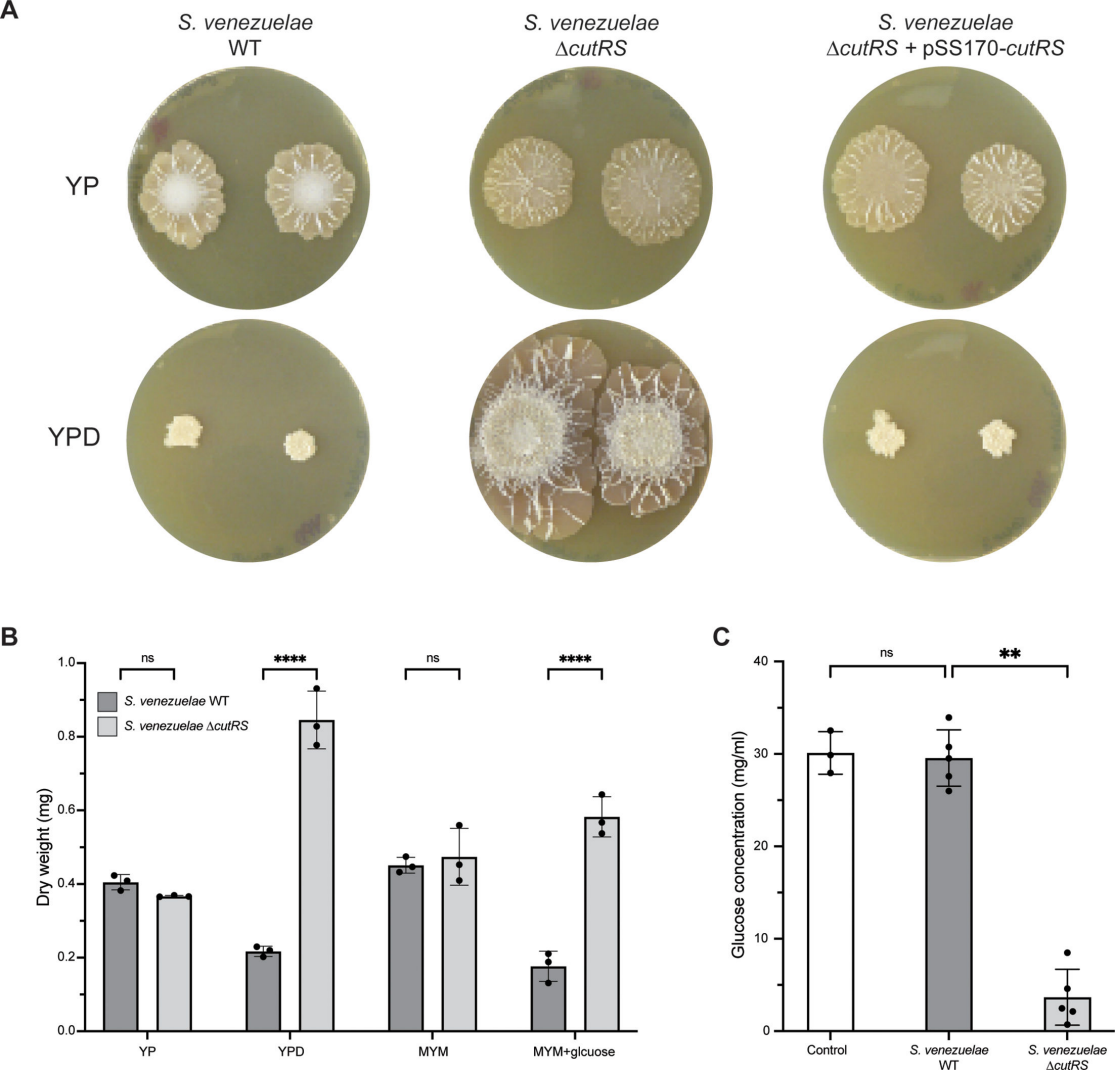
The CutRS two-component system is involved in colony growth and glucose consumption. (**A**) *S. venezuelae* wild-type, Δ*cutRS,* and Δ*cutRS* + pSS170-*cutRS* (complemented strain) colonies grown on YP (− glucose) and YPD (+ glucose) agar for 10 days at 30°C. (**B**) The colony dry weight (mg) of *S. venezuelae* wild-type and Δ*cutRS* strains after 10 days of growth at 30°C on YP, YPD, MYM, and MYM + glucose. ****Two-way ANOVA, *P*-value <0.0001. (**C**) The remaining glucose concentration (mg/mL) in spent media after 10 days of growth at 30°C (*S. venezuelae* wild-type or Δ*cutRS*). One-way ANOVA followed by Tukey’s multiple comparisons test: ** = adj. *P*-value <0.005 (0.0011). ns, not significant.

To investigate the function of CutRS, we deleted the *cutRS* operon in *S. venezuelae* and found that the ∆*cutRS* mutant exhibits a striking phenotype when grown on yeast peptone + d-glucose (YPD) agar. YP and YPD agar were chosen because *S. venezuelae* explores on YP, and this is inhibited by the addition of d-glucose (YPD). The mutant displays increased antibacterial activity and accelerated growth on YPD agar, suggesting it explores even in the presence of glucose (Fig. 1, 2A and B). Introduction of *cutRS in trans* on a phage integrative plasmid restores the wild-type phenotype to the ∆*cutRS* mutant, indicating that the observed phenotypes are solely due to loss of *cutRS*. Further analysis revealed that the ∆*cutRS* strain produces 10-fold more biomass than the wild-type strain on YPD agar. This phenotype was not restricted to YPD medium; a similar increase in biomass was observed on MYM agar, but again only in the presence of d-glucose (Fig. 2B). This increased growth correlates with the rapid depletion of d-glucose from the medium (Fig. 2C). Consistent with this, in the absence of d-glucose (YP agar), no phenotypic differences were observed between the wild-type and ∆*cutRS* strains (Fig. 1A, 2A and B).

When challenged with *Bacillus subtilis* on YP agar, both the wild-type and ∆*cutRS* strains produced small zones of inhibition (Fig. 1A). This is consistent with previous reports of *S. venezuelae* exploration, where trimethylamine production caused an increase in pH, leading to inhibition of localized bacterial growth by reducing iron availability (13). However, on YPD agar, bioactivity was lost in the wild-type strain, consistent with the known inhibition of antibiotic biosynthesis by d-glucose via carbon catabolite repression (14), while the ∆*cutRS* strain exhibited a markedly larger zone of inhibition (Fig. 1A). This suggests that d-glucose triggers antibiotic overproduction in the ∆*cutRS* strain, supporting previous observations that glucose activates actinorhodin production in the *S. coelicolor* ∆*cutRS* mutant (11). Notably, this is atypical, as d-glucose generally represses antibiotic production in *Streptomyces* species (14, 15).

*S. venezuelae* is known to produce chloramphenicol and jadomycin, both of which inhibit *B. subtilis*. To determine which antibiotic was responsible for the observed activity, we deleted both BGCs to generate strain M1702. This strain failed to inhibit *B. subtilis* growth, indicating that the bioactivity of the parent strain was due to either chloramphenicol or jadomycin (Fig. 1A). To further resolve this, we challenged the wild-type and ∆*cutRS* strains with a chloramphenicol-resistant *B. subtilis* strain. The ∆*cutRS* strain produced only a faint zone of inhibition, markedly smaller than that observed against the chloramphenicol-sensitive strain, confirming that the bioactivity was primarily due to chloramphenicol (Fig. 1B). The faint zone of inhibition observed for the chloramphenicol-resistant *B. subtilis* strain is likely due to some residual sensitivity to this antibiotic.

Examination of the tandem mass tag (TMT) proteomics data for the wild-type and ∆*cutRS* strains grown for 2 or 9 days on YPD agar shows that the chloramphenicol BGC is slightly upregulated (<2-fold) in the ∆*cutRS* mutant after 2 days growth and is significantly downregulated (>two-fold) after 9 days growth. The only BGC-encoded protein that is upregulated significantly is the chloramphenicol efflux pump CmlF, which is increased two-fold in 2-day-old cultures and seven-fold in 9-day-old cultures of the ∆*cutRS* strain. Increased export of chloramphenicol may account for the increased zone of inhibition in the ∆*cutRS* mutant grown on YPD agar but does not explain why we see more chloramphenicol being produced (Fig. 1C). It is possible that this is due to increased pathway activity, which can only be measured by quantifying chloramphenicol. The ∆*cutRS* strain produced approximately three times more chloramphenicol than the wild-type in the presence of d-glucose (Fig. 1C).

### Identifying CutR target promoters and binding sites in *S. venezuelae*

Two-component systems typically regulate gene expression through their RRs, in this case, CutR. To define the CutR regulon, we complemented the *S. venezuelae* ∆*cutRS* strain with a modified *cutRS* operon encoding wild-type CutS and a C-terminally 3×Flag-tagged CutR. We then cultured both the wild-type and Flag-tagged strains on YP and YPD agar and performed chromatin immunoprecipitation followed by sequencing (ChIP-seq) using monoclonal α-Flag antibodies.

**Fig 3:**
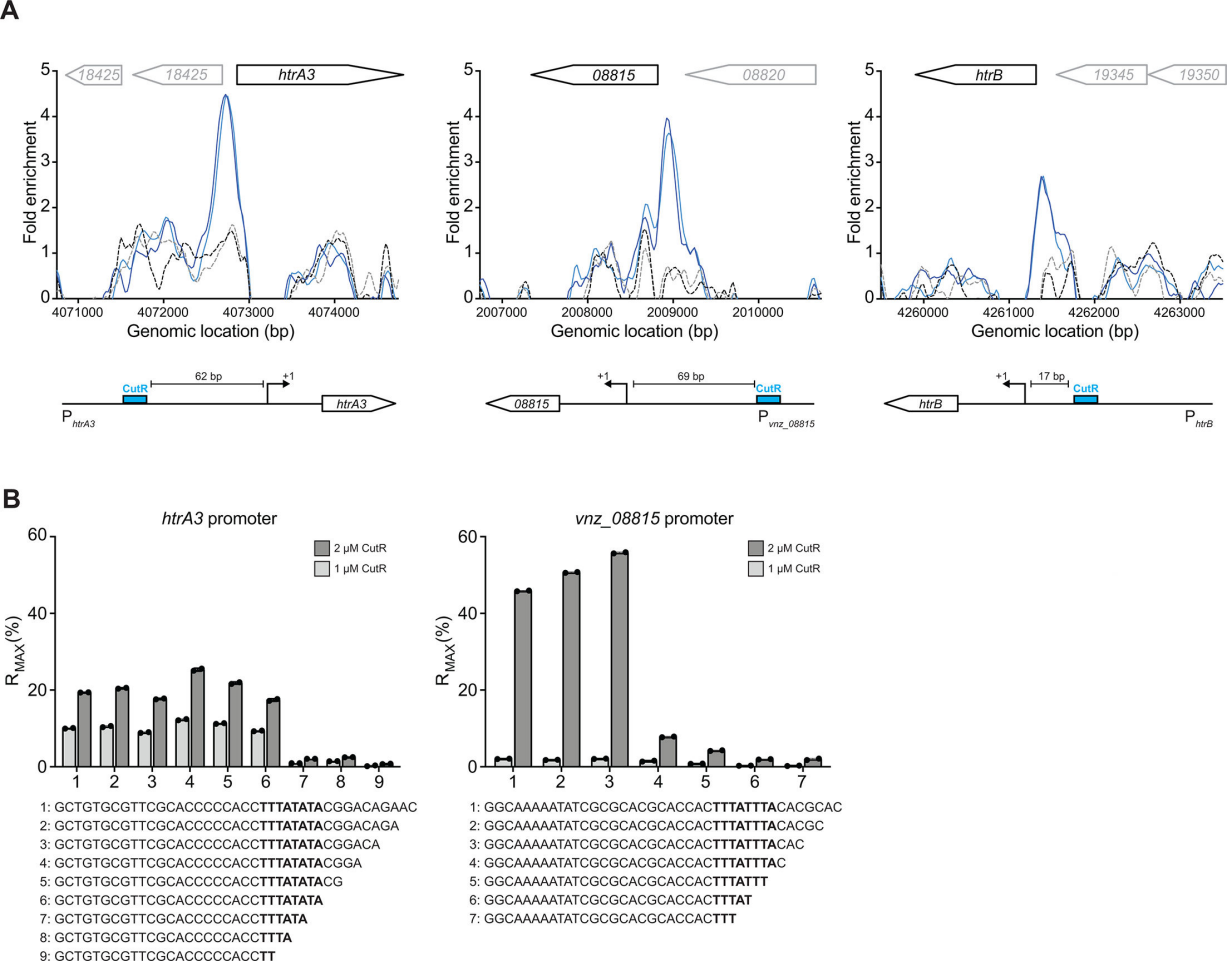
The defined DNA binding site for the CutR response regulator. (**A**) ChIP-seq peaks for biological replicates of CutR-3×FLAG (blue lines) and the wild-type control (gray lines). CutR binds upstream of the genes *htrA3*, *vnz_08815,* and *htrB*. (**B**) ReDCaT SPR was used to determine the binding site of 6xHis-CutR to the promoter regions of *htrA3* and the putative target gene *vnz_08815*. Sequential truncations of 2 bp were used to define the precise binding motif of TAWATAAA. The %RMAX was determined using the molecular masses of the annealed oligo duplex and monomeric 6xHis-CutR. This binding motif was then used to identify the CutR binding site upstream of *htrB* (**A**).

Analysis of the sequencing data (accession number GSE225370) revealed a single significantly enriched peak in the Flag-tagged CutR strain grown on YP agar (absence of d-glucose), located upstream of the *cutRS* operon. In contrast, 10 enriched peaks were detected in cultures grown on YPD agar (presence of d-glucose). These included peaks upstream of *cutRS*, *htrA3,* and *htrB*, which are also known CutR targets in *S. coelicolor* (Fig. 3A), as well as a strong peak upstream of *vnz_08815*, a putative cell wall amidase gene.

To precisely define CutR binding sites, we expressed and purified hexahistidine-tagged CutR for use in a DNA footprinting method called Reusable DNA Capture Technology Surface Plasmon Resonance (ReDCaT SPR) (16). Using tiled, double-stranded oligonucleotide probes spanning the promoters of *htrA3* and *vnz_08815*, we measured CutR binding affinity via SPR. A preliminary scan identified the core binding regions, and these probes were then truncated in 2 bp steps to determine the minimal binding sequences (Fig. 3B). CutR bound the sequence TATATAAA in the *htrA3* promoter and TAAATAAA in the *vnz_08815* promoter (Fig. 3B). From these and other enriched regions, we defined the CutR consensus binding sequence as TAWATAAA. Despite being enriched in the ChIP-seq data, the *cutRS* operon promoter was not bound by CutR in the ReDCaT SPR experiments, and we could not identify a CutR binding site at this promoter, so the *cutRS* operon is not included in the list of CutR-dependent promoters (Table 1).

**TABLE 1:** Gene promoters with CutR binding sites that are enriched in the ChIP-seq data set^*a*^

Gene	Predicted function	CutR binding site relative to TSS	Binding site sequence	Predicted effect	Protein abundance (WT/∆*cutRS* on YPD)	
					2 days	9 days
*vnz_06385*	Acyl coA dehydrogenase	+21	TAATTAAA	Repression	0.795	0.644
*vnz_06390*	TetR-family regulator	+28	TAATTAAA	Repression	0.947	2.457
*vnz_08815*	Cell wall amidase	−69	TAAATAAA	Activation	2.56	21.732
*vnz_12615*	Membrane protein	-4	TACATAAA	Repression	ND^*b*^	ND
*vnz_18430* (*htrA3*)	Quality control protease	−62	TATATAAA	Activation	9.131	8.134
*vnz_19340* (*htrB*)	Quality control protease	−17	CATATAAA	Repression	0.053	0.492
*vnz_19335* (*cssR*)	Response regulator	−17	CATATAAA	Repression	0.255	0.477
*vnz_19330* (*cssS*)	Sensor kinase	−17	CATATAAA	Repression	0.207	0.210
*vnz_29845*	NAD(P)/FAD-dependent oxidoreductase	−28	CAAATAAA	Repression	0.707	0.191
*vnz_33990*	Beta mannosidase	−21	TAAGTAAA	Repression	0.747	1.036
*vnz_33995*	Sugar-binding protein for ABC transport	+1	TAAGTAAA	Repression	0.977	0.107

^*a*^ The binding site positions are given relative to the transcription start sites (TSS). The DNA sequences of the CutR binding sites are shown, two of which (*htrA3* and *vnz_08815*) were verified *in vitro* using ReDCaT SPR (Fig. 3). Protein abundance data are from TMT proteomics experiments performed on cultures of the *S. venezuelae* wild-type (WT) and *∆cutRS* strains grown on YPD agar for 2 days and 9 days. Note that *htrB-cssRS* likely form an operon under the control of a single CutR-dependent promoter.

^*b*^ ND, not detected.

### CutR is both a transcriptional activator and repressor

Having identified CutR binding sites at its target promoters (Table 1), we cross-referenced them with publicly available *S. venezuelae* transcription start site (TSS) data from http://streptomyces.org.uk (17). The CutR binding site at the *htrB* promoter is located 17 bp upstream of the TSS, while at the *htrA3* promoter, it lies 62 bp upstream (Table 1; Fig. 3A). The position of a regulator binding site relative to the TSS often determines whether it functions as an activator or repressor (18). Binding 17 bp upstream of the *htrB* TSS likely overlaps the core promoter region, interfering with RNA polymerase binding and resulting in transcriptional repression (19, 20). In contrast, binding further upstream, such as at the *htrA3* promoter, may facilitate transcriptional activation via interaction between CutR and RNA polymerase bound at the −35 site (20–22).

**Fig 4:**
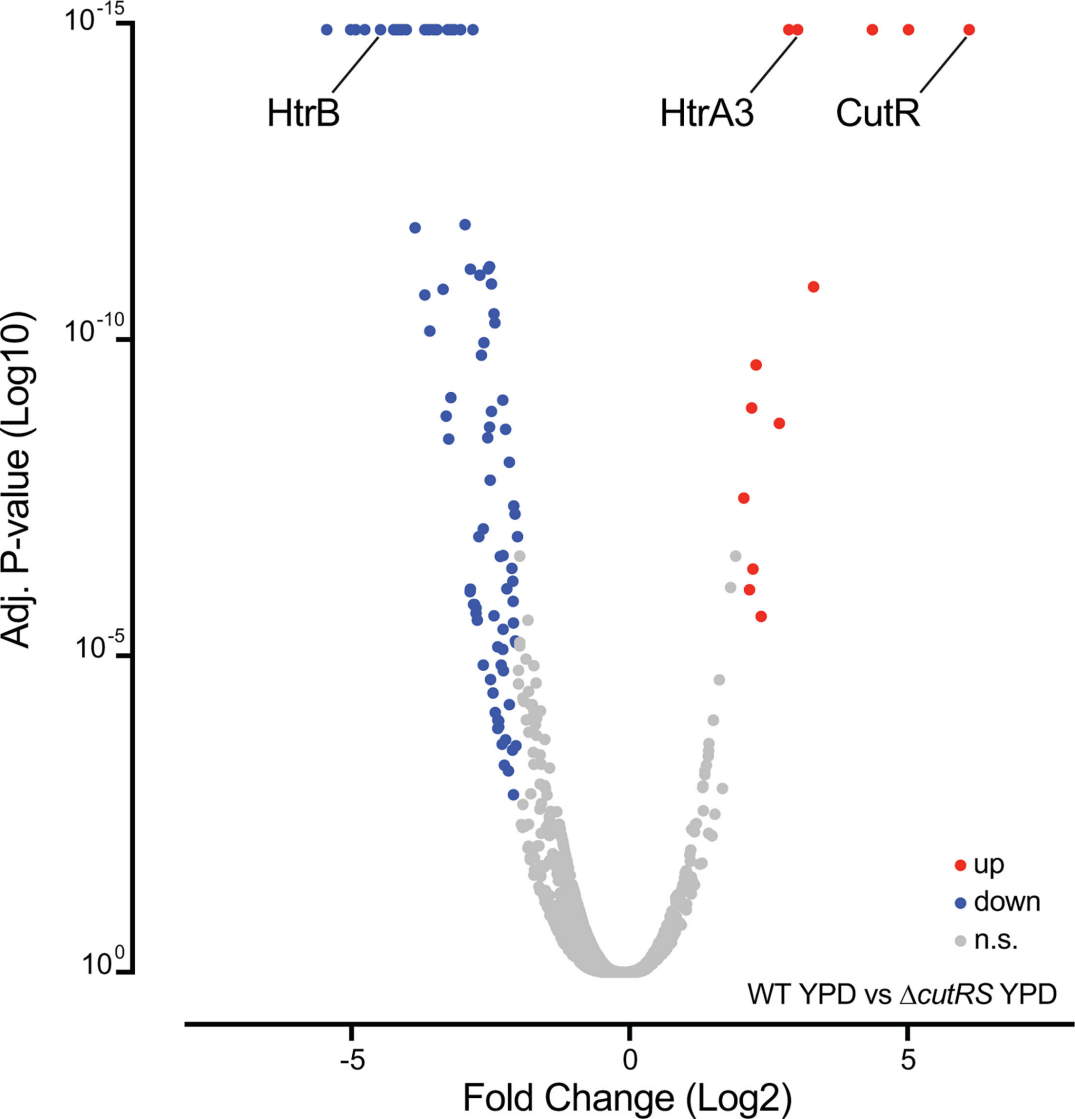
The deletion of *cutRS* causes a global shift in the proteome. Volcano plot of the fold change (log2) protein abundance detected by TMT proteomics against the calculated adjusted *P*-value (log10) for *S. venezuelae* wild-type (wild-type) and Δ*cutRS* strains grown on YPD agar. The abundance of CutRS in the wild-type samples appears exaggerated due to data processing against the Δ*cutRS* samples and does not represent normal levels.

To assess the effect of *cutRS* deletion on CutR target gene products, we used TMT quantitative proteomics to compare the wild-type and Δ*cutRS* strains grown for 2 days and 9 days on YPD agar. Of the 11 predicted CutR-dependent proteins, 8 were detected (Table 1). These data show that CutR activates expression of HtrA3 (9-fold increase in the wild-type strain compared to the Δ*cutRS* mutant) and the putative cell wall amidase Vnz_08815 (21-fold increase), consistent with the location of its binding sites upstream of their promoters (Fig. 4; Table 1). In contrast, CutR represses the production of HtrB (19-fold increase in the Δ*cutRS* strain relative to the wild-type), as well as CssR (3.9-fold increase) and CssS (4.8-fold increase), which are likely encoded in an operon under the control of the *htrB* promoter. CutR also represses the putative oxidoreductase Vnz_29845 (fivefold increase) and the sugar-binding protein Vnz_33995 (ninefold increase), consistent with CutR binding in a position that occludes RNA polymerase binding (Table 1). Levels of the TetR-family regulator Vnz_06390 increased 2.5-fold in the wild-type relative to Δ*cutRS* after 9 days growth on YPD agar despite the CutR binding site being downstream of the transcript start site (Table 1). The levels of the remaining detected target gene products were not significantly affected by *cutRS* deletion. This may be because these genes are not directly regulated by CutR under the tested growth conditions, because CutR binding does not alter transcription in all cases, or because additional layers of regulation are involved (Table 1).

### CutS activity is controlled by two conserved cysteines in its extracellular sensor domain

The conservation of *htrA3* and *htrB* as CutR targets in the distantly related species *S. coelicolor* and *S. venezuelae* supports the hypothesis that CutRS plays a role in the protein secretion stress response. This further implies that CutRS complements the function of CssRS, which activates the expression of *htrA1*, *htrA2,* and *htrB*, but not *htrA3* (23). We thus set out to investigate how the extracellular sensor domain, which is predicted to be between the two transmembrane helices of CutS, might detect defects in protein folding on the outside of the cell. We first aligned the extracellular sensor domains of >100 CutS proteins taken from complete, published *Streptomyces* genome sequences to look for conserved residues or motifs. This analysis identified two cysteine residues that are conserved across every CutS sensor domain (Fig. S1). Cysteines are important in extracellular protein folding in actinobacteria (24) and, consistent with this, the analysis of all proteins encoded in *S. venezuelae* with a Sec signal sequence revealed that 74% contain two or more cysteines that likely form disulfide bonds to help them fold. We thus hypothesized that CutS monitors disulfide bond formation in Sec-translocated proteins. Given the conservation of the two cysteine residues, we reasoned that this could be via the making or breaking of a disulfide bond in the CutS extracellular sensor domain.

**Fig 5:**
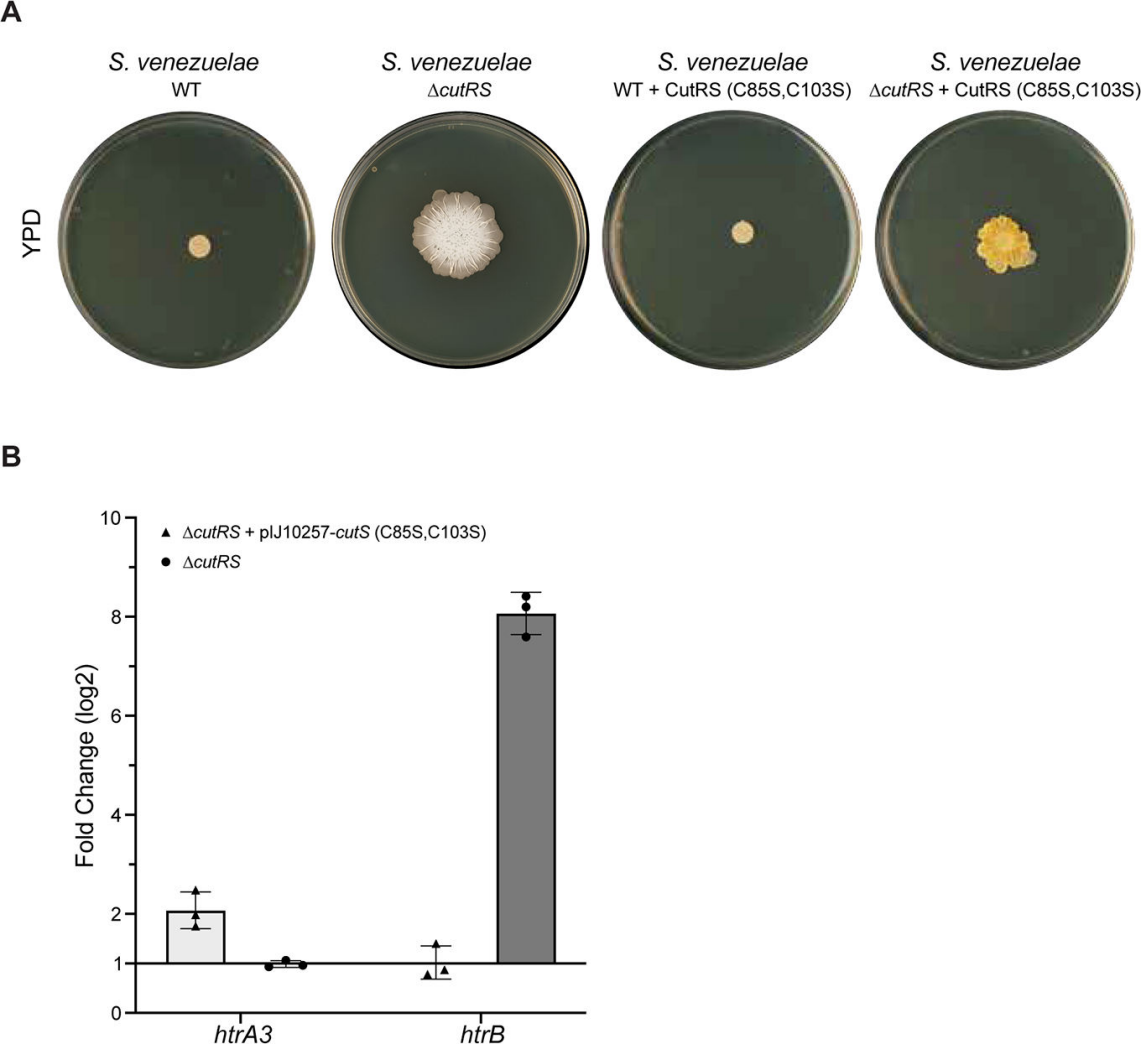
The conserved extracellular cysteine residues control the activity of CutS. (**A**) The Δ*cutRS* mutant can be partially complemented by *in-trans* expression of an operon encoding wild-type CutR and a CutS protein in which the extracellular cysteines are altered to serine (CutS(C85S,C103S)). (**B**) Quantitative RT-PCR analyzing the abundance of *htrA3* and *htrB* in *S. venezuelae* Δ*cutRS* + pIJ10257–*cutRS* (C85S,C103S) (light gray bars) and *S. venezuelae* Δ*cutRS* (dark gray bars). The transcripts were normalized using the constitutively expressed *hrdB* and compared to wild-type expression so that each bar represents the normalized expression change compared to the wild-type strain. Error bars represent standard deviation across replicates.

Modeling of the *S. venezuelae* CutS extracellular sensor domain using AlphaFold indicated that these cysteine residues likely sit within 5 Å of each other, the distance required to form disulfide bonds (Fig. S2). To test this, we designed a CutS variant where both cysteine residues were replaced with serine (CutS(C85S,C103S)) and introduced it into the *S. venezuelae* wild-type and ∆*cutRS* strains along with the wild-type *cutR* gene. This restored a wild-type growth phenotype to the ∆*cutRS* mutant (Fig. 5A), which suggests the CutS(C85S,C103S) variant is active. However, previous work on the VanRS TCS demonstrated that it is possible for loss of an SK to render its cognate RR constitutively active (25). To discount this possibility, we introduced individual copies of either *cutS* or *cutR* under the control of the ermE* promoter into the ∆*cutRS* mutant, but neither restored the wild-type phenotype, confirming that CutS is required for CutR activity. To further investigate the activity of the CutS(C85S,C103S) variant, we used quantitative reverse transcription PCR (qRT-PCR) to measure the expression of the CutR target genes *htrA3* and *htrB* in the wild-type, ∆*cutRS,* and CutS(C85S,C103S) strains (Fig. 5B). The expression of *htrA3* was higher in the strain harboring the CutS(C85S,C103S) variant compared to the wild-type strain, while the expression of *htrB* was reduced (Fig. 5B), which is consistent with CutS(C85S,C103S) activating CutR, which then directly activates the transcription of *htrA3* and represses *htrB*. Thus, we conclude that replacing the two extracellular sensor domain cysteines with serine residues results in a constitutively active CutRS system, which supports the hypothesis that an inability to form disulfide bonds outside the cell activates CutRS and triggers the secretion stress response.

### The wild-type phenotype is recovered by the deletion of *cssRS* in the ∆*cutRS* background

**Fig 6:**
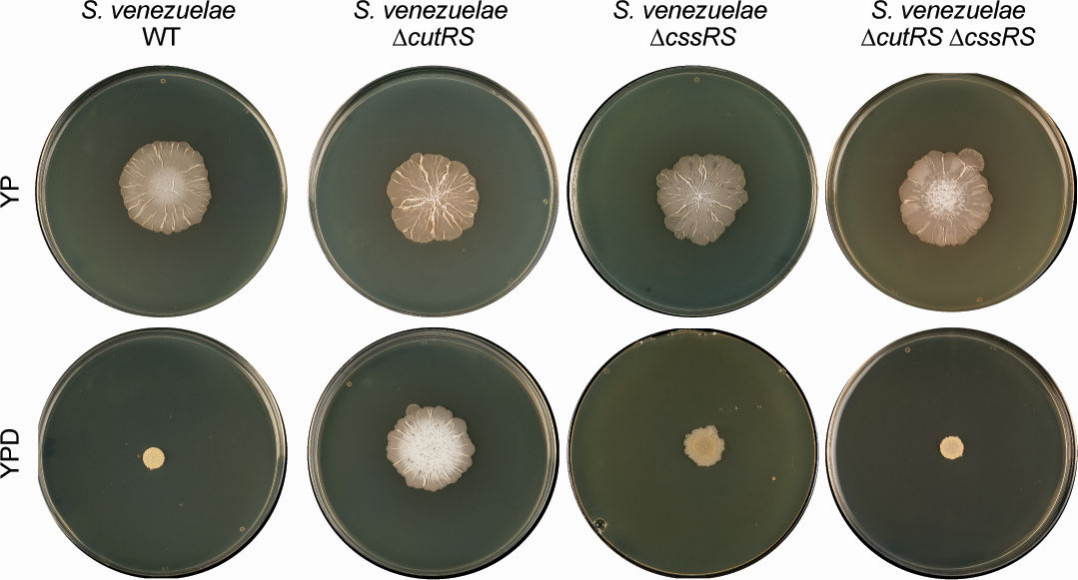
The *S. venezuelae* wild-type phenotype is recovered by deletion of *cssRS* in the Δ*cutRS* background. *S. venezuelae* wild-type, Δ*cutRS*, Δ*cssRS,* and Δ*cutRS-ΔcssRS* strains grown on YP (− glucose) and YPD (+ glucose) agar for 10 days at 30°C.

This work and previous reports suggest that the secretion stress response in *Streptomyces* species is controlled by the CssRS and CutRS two-component systems, which together control the expression of *htrA1*, *htrA2*, *htrA3,* and *htrB*, which encode the four conserved HtrA-family foldases in *Streptomyces* species (11, 23). Although the CssR regulon has not yet been defined, electrophoretic mobility shift assays alongside qRT-PCR were used previously in *S. lividans* (a very close relative of *S. coelicolor*) to show that CssR directly activates expression of *htrA1*, *htrA2,* and *htrB*, but not *htrA3* (23). Instead, the expression of *htrA3* is directly activated by CutR, which also represses the expression of *htrB* in *S. coelicolor* and *S. venezuelae* (11). We attempted to replicate the ∆*cutRS* phenotype by deleting *htrA3* and overexpressing *htrB* in the same background (Fig. S3), but this did not result in the phenotypic change on YPD that we observed with ∆*cutRS*. We thus reasoned that the CssRS system might be compensating for the loss of CutRS. To further investigate the interplay of CssRS and CutRS, we made single and double ∆*cssRS* and ∆*cutRS* mutants of *S. venezuelae* and observed their phenotypes on YP and YPD agar. In the absence of d-glucose, the wild-type, ∆*cssRS*, ∆*cutRS,* and ∆*cssRS-*∆*cutRS* strains all displayed the same phenotype (Fig. 6). On YPD agar, growth is reduced in the wild-type strain by the addition of d-glucose to the medium, but the ∆*cutRS* mutant consumes the glucose and reverts to a phenotype like that observed on YP agar (Fig. 1 and 2). Deletion of *cssRS* in the wild-type background has no obvious effect on growth on either YP or YPD agar, but deletion of *cssRS* in the ∆*cutRS* mutant restores the wild-type phenotype to the ∆*cutRS* mutant growing on YPD agar, suggesting that the altered growth of the ∆*cutRS* mutant on YPD is dependent on CssRS (Fig. 6). Proteomics analysis of the wild-type and ∆*cutRS* strains revealed that the levels of CssRS are increased fivefold in the ∆*cutRS* mutant, and as previously discussed, HtrB levels (activated by CssR and repressed by CutR) are increased 20-fold in the absence of *cutRS*. The *htrB* and *cssRS* genes are located adjacent to each other and likely form an operon under the direct control of both CutR (negative) and CssR (positive). Overproduction of HtrB could be due to the loss of CutR repression in the mutant strain combined with the overproduction of CssRS, with CssR further activating *htrB* expression. A future challenge will be to define the CssR regulon and determine how deletion of *cssRS* complements ∆*cutRS*.

### Extracellular disulfide sensing could be conserved across bacteria

**Fig 7:**
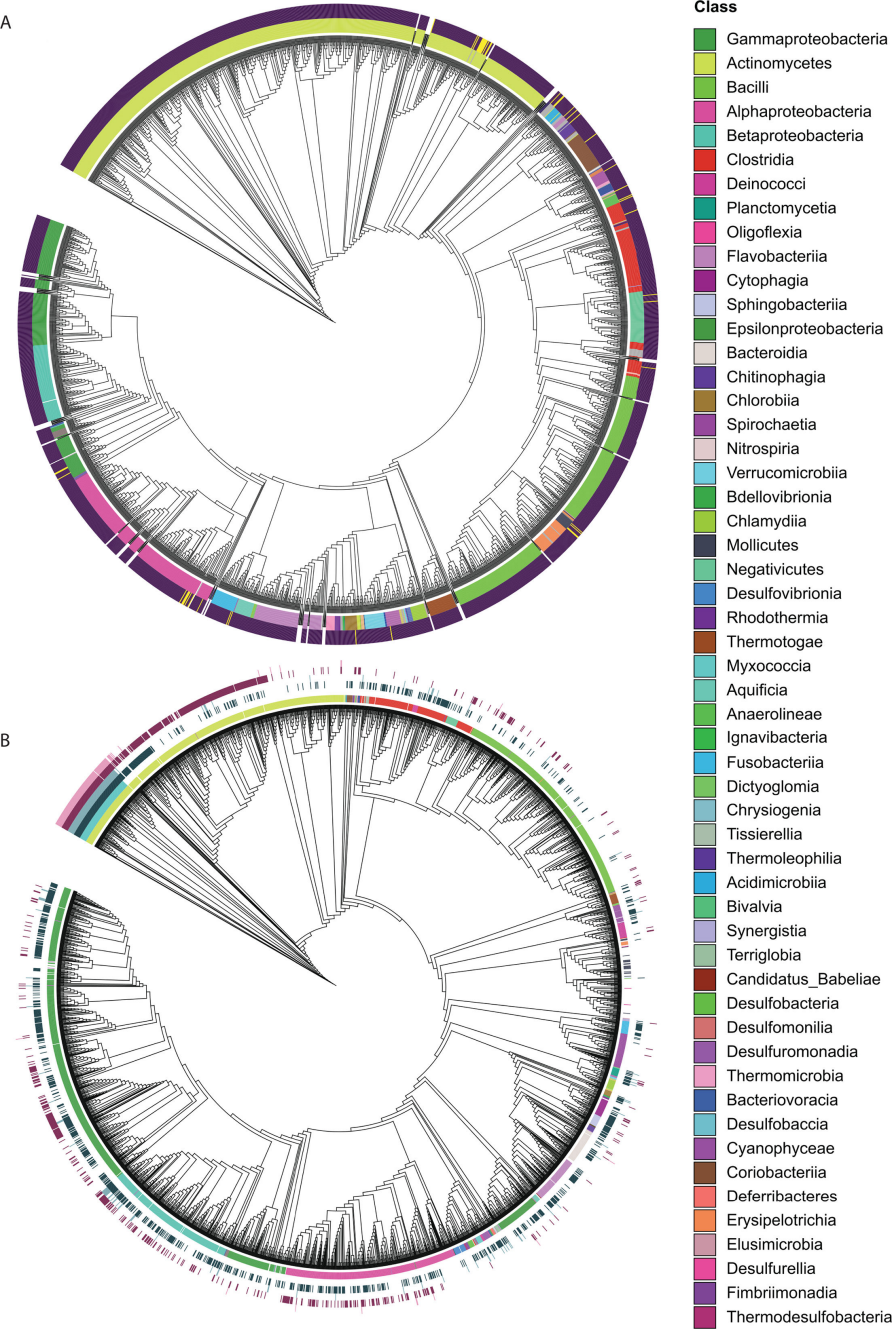
Phylogenetic and cysteine residue analysis of bacterial sensor kinases. (**A**) Maximum-likelihood phylogenetic tree of 348,633 predicted SKs from 12,799 high-quality bacterial genomes. The outer ring is color-coded by bacterial class, as indicated in the legend. The heatmap overlay in the inner ring shows whether that genome contains at least one sensor kinase with two or more cysteine residues in its extracellular domains, as predicted by DeepTMHMM. Dark purple indicates yes, and yellow indicates no. (**B**) Maximum-likelihood phylogenetic tree based on RpoB protein sequences from 2,940 bacterial reference genomes. The outer rings represent the following: bacterial class, as indicated in the legend; genomes in the *Streptomyces* genus (light green); CutS reciprocal BLAST best hits (RBBH) (dark blue); CutS homologs with two or more cysteine residues in their extracellular domains, as predicted by DeepTMHMM (light blue); CssS RBBH (dark pink); CssS homologs with two or more cysteine residues in their extracellular domains, as predicted by DeepTMHMM (light pink). Trees were constructed using MAFFT for sequence alignment of RpoB protein sequences and FASTTREE for maximum-likelihood inference and visualized in iTOL.

There are 15 conserved two-component systems in *Streptomyces* species, and the sensor kinases of all 15 are predicted to be transmembrane. Seven of these (47%) are predicted to have two transmembrane domains surrounding an extracellular domain, thus forming the canonical sensor domain (9). Analysis of these 15 conserved two-component systems (9, 26, 27) also revealed that only CutS and CssS have two conserved extracellular cysteines in their sensor domains (Fig. S2 and S4). Given that these cysteines are implicated in regulating CutS activity, we hypothesize that a similar mechanism may apply to CssS and potentially other bacterial SKs. To assess the prevalence of this feature across the bacterial kingdom, we analyzed 12,799 high-quality bacterial genomes, comprising all reference genomes at scaffold level or higher available in the NCBI database at the time of analysis, and identified 348,633 proteins predicted to function as SKs. We then applied DeepTMHMM (28) to these sequences. We predicted the transmembrane helices for all the SKs and then used this information to identify the putative sensor domains of these proteins and quantify the number of cysteine residues present in these extracellular domains. We observed that 98.9% of all the bacterial strains examined encode at least one SK with two or more cysteines in their predicted extracellular sensor domains (Fig. 7A). The other 1.1% largely belong to a new order of auxotrophic actinobacteria tentatively named *Candidatus* Nanopelagicales that were isolated from Lake Zurich and have streamlined genomes between 1.2 and 1.4 Mbp (29).

Next, we examined the conservation of the CutS and CssS SKs beyond the genus *Streptomyces*. Reciprocal BLAST searches in 2,936 bacterial reference genomes revealed that 39% encoded a CutS homolog and 27% encoded a CssS homolog. Of the 2,936 genomes examined, 53% encode at least one of these two SKs and 12% encode both CutS and CssS. However, further inspection of the amino acid sequences of these orthologues revealed that only 6.9% of CutS and 4.8% of CssS homologs contain two or more cysteines in their extracellular domains. Phylogenetic analysis using a maximum-likelihood tree based on RpoB revealed a broad distribution of these homologs across bacterial taxa (Fig. 7B). The presence of both CutS and CutR with two extracellular cysteines was universally conserved across all tested *Streptomyces* strains, with only three additional genomes, all from actinobacteria, also containing both. This may reflect the fact that actinobacteria are more likely to use disulfide bond formation as a mechanism to fold their secreted proteins (24). Taken together, our findings suggest that extracellular redox sensing via conserved cysteine residues in the sensor domains of SKs may represent a broadly important mechanism across the bacterial kingdom. While CutS and CssS appear to be largely restricted to the genus *Streptomyces*, this sensing function could be carried out by a diverse array of SKs in other bacterial lineages.

## DISCUSSION

In this study, we demonstrate that loss of the highly conserved CutRS TCS in *Streptomyces venezuelae* leads to dramatic changes in growth, glucose metabolism, and antibiotic production. To investigate the underlying mechanisms, we first defined the *S. venezuelae* CutR regulon. This revealed two conserved CutR targets*, htrA3* and *htrB*, shared between *S. venezuelae* and the distantly related *S. coelicolor*. These genes encode members of the HtrA family of proteases. In *S. venezuelae*, CutR activates *htrA3* and *vnz_08815*, a gene predicted to encode a cell wall amidase of unknown function. In a previous study, ChIP-seq identified 85 CutR target genes in *S. coelicolor* (11) compared to only 10 CutR binding sites identified in *S. venezuelae* in this work. Both ChIP-seq experiments were carried out on solid agar with and without glucose, although different growth media were used, namely Difco Nutrient Agar (DNA) for *S. coelicolor* and YP agar for *S. venezuelae*. To check that no binding sites were missed in *S. venezuelae,* we determined the CutR recognition sequence using ReDCaT SPR and searched the genome. The only sites identified were those listed in Table 1, so we conclude that CutR has a much smaller regulon in *S. venezuelae* than *S. coelicolor*. The reasons for this are not yet known.

To try and understand the effects of *cutRS* deletion on *S. venezuelae* growth and glucose consumption, we analyzed the proteomics data, comparing wild-type to mutant (Fig. 4; Tables S3 and S4). These data indicate significant upregulation of several genes that are annotated as peptidoglycan hydrolase and transglycosylase genes in the *S. venezuelae* ∆*cutRS* strain, which may be indicative of increased peptidoglycan turnover required for more rapid growth. These effects are indirect, however. In contrast, CutR directly represses the production of HtrB, as well as CssR and CssS, which likely form an operon with *htrB* under the control of the *htrB* promoter. CutR also directly represses production of the putative oxidoreductase Vnz_29845 (fivefold increase) and ABC transporter sugar-binding protein Vnz_33995 (ninefold increase), which may be linked to the observed glucose phenotype. Examination of the proteomics data shows no significant effects on the abundance of the glucose transporter GlcP or the glucose kinase GlcK in the ∆*cutRS* mutant. However, the IIc subunits Vnz_13030 and Vnz_13035 of the phosphotransferase system (PTS) are upregulated 125- and 15-fold, respectively, in the ∆*cutRS* strain. These subunits are responsible for the linked transport and phosphorylation of sugars and may be responsible for the import and phosphorylation of glucose. Note, however, that carbon catabolite repression is reported to be independent of PTS (15).

CutR only binds to its target gene promoters *in vivo* in the presence of glucose, and the ∆*cutRS* mutant rapidly consumes glucose and forms 10 times more biomass than the wild-type strain on glucose-containing YPD agar. In addition to this, growth of the ∆*cutRS* mutant on glucose triggers the overproduction of antibiotics in both *S. coelicolor* and *S. venezuelae*, somehow bypassing the usual glucose-dependent carbon catabolite repression of antibiotic biosynthesis observed in these species. The link between glucose and the protein secretion stress response mediated by CutRS remains unclear, but glucose is a non-preferred carbon source for *Streptomyces* bacteria. It is possible that growth on d-glucose triggers increased protein secretion to scavenge for other carbon sources and that this results in extracellular protein misfolding and triggers the secretion stress response via activation of CutS. Loss of CutRS forces the bacteria to consume the d-glucose, leading to rapid growth, increased biomass, and the overproduction of antibiotics.

Using ReDCaT SPR, we identified a consensus CutR binding motif (TAWATAAA) in the promoters of *htrA3* and *vnz_08815*, which was also found in all the CutR-enriched promoter regions in the ChIP-seq experiment, except for the *cutRS* operon promoter. We further showed that the position of CutR binding relative to TSSs typically correlates with transcriptional outcome—either activation or repression. Given that CutRS coordinates the expression of two of the four conserved *htrA*-like genes in *S. venezuelae* and *S. coelicolor*, we conclude that CutRS plays a role in the secretion stress response, likely acting in parallel with the CssRS TCS.

While the CssR regulon remains undefined, previous work shows that CssR activates *htrA1*, *htrA2*, and *htrB* while having no effect on *htrA3* (23). CssRS also activates *htrA* and *htrB* in *Bacillus subtilis* (30). Thus, in *Streptomyces* species, CutRS and CssRS appear to act in opposition: they regulate distinct subsets of *htrA*-family genes and exert opposing effects on *htrB* expression. HtrA proteins support extracellular protein folding and degrade irreparably misfolded proteins in both Gram-negative and Gram-positive bacteria (31). CssRS, conserved in Gram-positive species, is homologous to the envelope stress-sensing system CpxAR in *Escherichia coli*. Our bioinformatic analysis shows that while CutRS and CssRS are widely conserved in *Streptomyces*, most other actinobacteria encode one or the other, and homologs are also present across other bacterial phyla (Fig. 7).

Proper folding of secreted proteins requires disulfide bond formation, catalyzed by DsbA-family proteins located on the extracellular face of the cytoplasmic membrane (32). The discovery that the sensor domain of CutS contains two conserved cysteines suggests a redox-based sensing mechanism. Indeed, mutating these residues to serines (C85S,C103S) led to constitutive activation of CutR, with elevated *htrA3* expression and enhanced repression of *htrB*. This supports a model in which CutS monitors disulfide bond formation as a proxy for protein folding quality in the extracytoplasmic space.

Intriguingly, the extracellular domain of CssS also contains two conserved cysteines, and CutS and CssS are the only conserved *Streptomyces* SKs with this feature. This suggests that CssS may similarly sense disulfide bond status, which we intend to explore in future work. Moreover, *cssRS* is overexpressed in the ∆*cutRS* mutant, and deleting *cssRS* in this background restores wild-type growth, further supporting antagonistic functions for these two systems. Additional work is needed to define the CssR regulon and elucidate how CutRS and CssRS jointly regulate the HtrA proteases.

**Fig 8:**
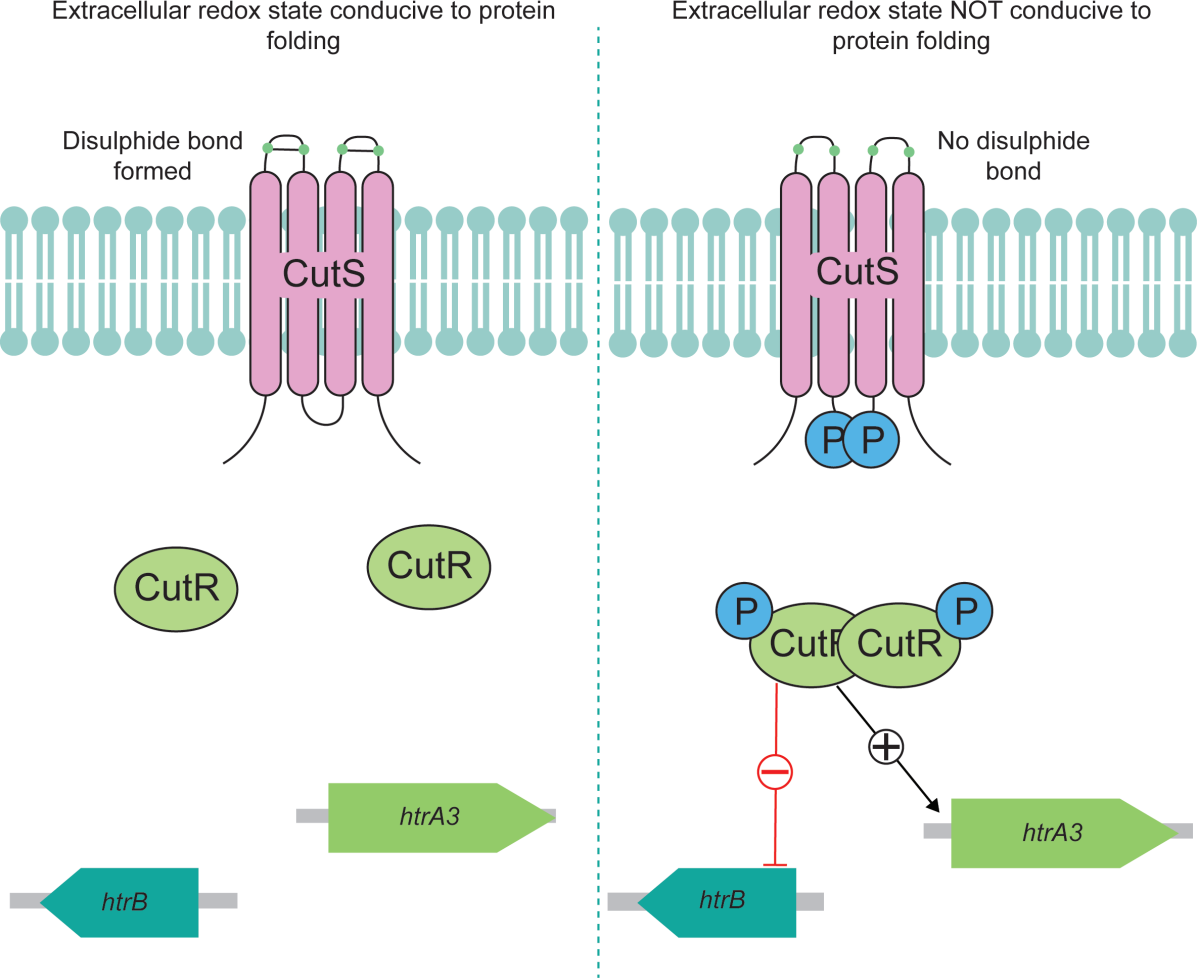
A proposed model for the extracellular redox-sensing activity of the CutRS system in *Streptomyces* spp. When the extracellular space is conducive to protein folding (left side), the cysteine residues in the extracellular sensor domain of the SK CutS (pink) form a disulfide bond. In this state, the CutRS system is inactive. When the extracellular space is not conducive to protein folding (right side), the disulfide bond in the extracellular sensor domain of CutS is unable to form. In this state, the SK CutS is activated, autophosphorylates, and transfers this phosphate to the cognate RR CutR (light green). Phosphorylated CutR homodimerizes and binds DNA, repressing the transcription of *htrB* and activating *htrA3.* These genes encode dual-function chaperone/proteases that can act to restore the cell to homeostasis.

To our knowledge, this is the first proposal of a sensing model for a bacterial secretion or envelope stress response system. *Streptomyces* species are common in soil and the plant rhizosphere and endosphere (33), where protein secretion plays a vital role in breaking down complex organic material for nutrient acquisition. These environments are also oxygen-variable. CutS could sense oxygen availability via disulfide bond formation (Fig. 8). Under anaerobic or microaerobic conditions, disulfide bond formation is likely to be impaired, and this would lead to an accumulation of misfolded extracellular proteins. This would also activate the CutRS system and promote the production of HtrA3 to rescue or degrade misfolded proteins outside the cell.

Oxygen sensing via disulfide bond status has precedence. In *Shewanella* species, the MtrABC porin-cytochrome complex facilitates extracellular electron transfer under anoxic conditions (34). The periplasmic MtrC subunit contains a conserved CX₈C motif that forms a redox-active disulfide only in the presence of oxygen, allowing the cell to modulate reactive oxygen species and electron flux (35). Similarly, in *Pseudomonas aeruginosa*, the periplasmic protein YfiR contains four redox-active cysteines, two of which form a disulfide under oxidative conditions. YfiR modulates the activity of the diguanylate cyclase YfiN in response to oxygen availability (36).

The widespread conservation of sensor kinases with extracellular cysteines across diverse bacterial phyla suggests that redox and oxygen sensing outside the cell could be a fundamental feature of bacterial stress response systems. Future research into these pathways may reveal new regulatory strategies for secretion stress, redox adaptation, and virulence in a broad range of bacterial species.

## MATERIALS AND METHODS

### Growth media and supplements

Protocols for culturing and genetically manipulating *Streptomyces* species are all freely available from http://actinobase.org (37). Strains were grown in the following media (per liter volume): YP agar (10 g yeast extract, 20 g bacto-peptone, 20 g agar); YPD (YP + d-glucose) agar (YP agar with the addition of 100 mL of a 40% filter-sterilized d-glucose); 2×YT (16 g tryptone, 10 g yeast extract, 5 g NaCl); SFM agar (20 g soy flour, 20 g mannitol, 20 g agar); lysogeny broth (LB) (10 g tryptone, 5 g yeast extract, 10 g NaCl); and LB soft agar (LB with the addition of 20 g agar). When appropriate, the media were supplemented with antibiotics at the following concentrations: hygromycin (50 µg/mL), apramycin (50 µg/mL), and nalidixic acid (25 µg/mL). NaCl was excluded from LB when hygromycin was used for selection. Note that the wild-type and ∆*cutRS* cultures shown in Fig. 5 and 6 are the same.

### Strains, plasmids, and primers

The bacterial strains, plasmids, and primers used or generated in this study are listed in Tables S1 and S2, respectively.

### Strain generation

Deletion mutants were complemented using the native gene and promoter cloned into the phage integrative vector pSS170 (37) using Gibson Assembly. Overexpression strains were generated by cloning the gene(s) of interest into pIJ10257 (37, 38) using Gibson Assembly. CRISPR-mediated gene deletions were carried out using pCRISPomyces2 as per the protocol described by Cobb et al. (39). In all cases, the resulting construct was then used to transform *E. coli* ET12567/pUZ8002 (40, 41) and conjugated into the mutant strains as follows. Single colonies of ET12567/pUZ8002 containing the complementation construct were inoculated into 10 mL LB with appropriate selection and incubated overnight at 37°C with shaking at 220 rpm. The overnight culture was sub-cultured 1:100 in 50 mL LB with appropriate selection and grown to OD_600_ ~0.6. The cultures were washed with 10 mL ice-cold LB twice to remove the antibiotic and finally resuspended in 1 mL ice-cold LB. Five hundred microliters of the *E. coli* cell suspension was mixed with 20 µL of *S. venezuelae* spores by inversion and then pelleted by centrifuging at 15,871 × *g* for 2 min. The supernatant was removed, and the pellet was resuspended in 150 µL residual supernatant. Serial 10-fold dilutions were plated on SFM and incubated at room temperature for 16–20 hours, overlaid with 1 mL sterile distilled H_2_O containing appropriate selection and then incubated at 30°C for 3–7 days until colonies appeared. Colonies were re-streaked on SFM agar with appropriate selection at least once before being plated for spore preparation.

### Generation of *S. venezuelae* strain M1702

Deletion of the chloramphenicol BGC and the jadomycin BGC was achieved using the meganuclease system (42). The upstream flank for the chloramphenicol BGC was amplified with oligonucleotides NH112ChlPCR1F and NH115ChlPCR2R, whereas the upstream flanking of the jadomycin cluster was amplified with NH120JadPCR1F and NH123JadPCR2R. The upstream flanking sequences were then cloned into the BgII and HindIII sites of pIJ12738 (42). The downstream flanking sequence of the chloramphenicol BGC was amplified with oligonucleotides NH116ChlPCR3F and NH119ChlPCR4R, whereas the upstream flanking sequence of the jadomycin BGC was amplified with NH124JadPCR3F and NH127JadPCR4R. The downstream flanking sequences were combined with the corresponding upstream flanking sequences in pIJ12738 by cloning into the HindIII and SpeI sites. These pIJ12738 derivatives containing the flanking sequencing for each cluster followed by an I-SceI meganuclease cut site were then mobilized via conjugation through *E. coli* ET12567/pUZ8002, and single-crossover recombinants were selected for with apramycin. We then conjugated pIJ12742 containing the meganuclease into the single-crossover recombinants. Expression of the meganuclease results in a double-strand break, which can be resolved by a second crossover event. Subsequent double crossovers were screened by PCR to determine if they were gene-cluster deletion mutants or if they had reverted to wild type. Gene-cluster deletion mutants were incubated at 37°C for two generations to promote loss of pIJ12742. The resulting strains were Δ*cml* (M1700) and Δ*jad* (M1701). M1700 was used as the starting material to introduce the jadomycin BGC deletion derivative of pIJ12738, and single-crossover recombinants were used to introduce the meganuclease (on pIJ12742) followed by screening for loss of the jadomycin BGC and removal of the plasmid, generating the final double mutant of Δ*cml*Δ*jad* (M1702).

### Antimicrobial bioassays

Five microliters of spores of the relevant *S. venezuelae* strains were inoculated onto the center of a YP or YPD agar plate and incubated for 5 days at 30°C. The *B. subtilis* indicator strain was grown in 10 mL LB at 37°C with 220 rpm shaking the night prior to the assay. Following overnight incubation, the indicator strains were sub-cultured 1:100 into 50 mL LB and grown to OD_600_ ~0.6 and then diluted 1:10 into 50°C soft LB agar. Five milliliters of this inoculated soft agar was then pipetted over the *S. venezuelae-*containing plate, ensuring complete coverage. The plates were air-dried and subsequently incubated at 37°C overnight before imaging.

### Biomass and glucose quantification assays

Five microliters of spores of the relevant *S. venezuelae* strains were inoculated on cellophane-covered YP or YPD agar plates for 5 days at 30°C. Following incubation, the cellophane disks were removed, and the mycelial mass was measured using an analytical balance. For glucose extraction, the underlying agar was chopped into small pieces using a sterile scalpel and transferred to a 250 mL conical flask. Twenty milliliters of sterile distilled H_2_O was added, and samples were incubated for 2 hours at 25°C with shaking at 220 rpm. The resulting liquid was aspirated from the flask and diluted 1:1,000 with sterile distilled H_2_O before being assayed using the Glucose (GO) Assay Kit (Sigma-Aldrich) following the manufacturer’s instructions.

### ChIP-seq

Five microliters of spores of the relevant *S. venezuelae* strains were inoculated onto cellophane-covered YP or YPD agar plates in triplicate and grown for 5 days at 30°C. To cross-link proteins to DNA, the cellophane disks were removed and submerged in 10 mL of fresh 1% (vol/vol) formaldehyde solution at room temperature for 20 min. Following a 5 min incubation in 10 mL 0.5 M glycine solution, the mycelium was harvested and washed twice with 25 mL ice-cold PBS (pH 7.4) before being flash-frozen in liquid nitrogen and stored at −80°C. For lysis, the pellets were resuspended in 2 mL lysis buffer (10 mM Tris-HCl, pH 8.0, 50 mM NaCl, 10 mg/mL lysozyme, EDTA-free protease inhibitor) before 750 µL was transferred to a 2 mL microcentrifuge, and samples were incubated at 37°C for 30 min. For fragmentation, 750 µL IP buffer (100 mM Tris-HCl, pH 8.0, 250 mM NaCl, 0.5% vol/vol Triton X-100, 0.1% SDS, EDTA-free protease inhibitor) was added, and samples were sonicated on ice at 50 Hz for 20 cycles of 10 s with at least 1 min of rest between each cycle. Twenty-five microliters of the crude lysate was combined with 75 µL TE buffer (10 mM Tris-HCl, pH 8.0, 1 mM EDTA) and extracted with 100 µL phenol:chloroform. Two microliters of 1 mg/mL RNase A was combined with 25 µL of the resulting extract, incubated at 37°C for 30 min, and run on a 1% agarose gel to confirm a smear centered at 500 bp. The remaining crude lysate was centrifuged for 15 min at 15,871 × *g* at 4°C, and the supernatant was saved. A total of 750 µL of α-FLAG M2 magnetic beads (Sigma-Aldrich) were washed in 3.75 mL 0.5% IP buffer (IP buffer: 150 mM NaCl, 1% Triton X-100, 50 mM Tris-HCl [pH 8.0], 2 mM EDTA, and Roche cOmplete, Mini, EDTA-free Protease Inhibitor Cocktail tablet(s)) to prepare them for binding. The bead slurry was then mixed with 40 µL clarified lysate and incubated overnight on a vertical rotator at 4°C. The lysate was removed, and the beads were washed four times with 500 µL 0.5 IP buffer with 10 min vertical rotation at 4°C each cycle. Washed beads were incubated in 100 µL elution buffer (50 mM Tris-HCl, pH 7.6, 10 mM EDTA, 1% SDS) overnight at 65°C. The eluant was removed and saved before an additional 50 µL elution buffer was added and incubated at 65°C for a further 5 min. Once removed, 3 µL of 10 mg/mL proteinase K was added to the combined eluants and incubated at 55°C for 2 hours. DNA was extracted with 200 µL phenol:chloroform and purified using a QIAquick PCR Purification Kit (QIAGEN) following the manufacturer’s instructions. DNA was eluted in 50 µL Buffer EB (10 mM Tris-HCl, pH 8.5) and 47 µL snap frozen in liquid nitrogen and stored at −80°C. The remaining 3 µL of sample was used to determine the DNA concentration and quality. The Qubit Fluorometer 2.0 with the high-sensitivity kit was used for DNA concentration, while DNA purity was quantified using a Nanodrop 2000 UV-Vis Spectrophotometer. The stored DNA samples were then shipped to Novogene on dry ice for sequencing using the NovaSeq 6000 Sequencing System (Illumina). Raw sequencing data were received as FASTQ files from Novogene and processed as described previously (11). Raw files were aligned to the *S. venezuelae* NRRL B-65442 genome and enrichment normalized to a moving window of 30 nucleotides compared to a region of the surrounding 3,000 nucleotides with the window moving in steps of 15 nucleotides.

### Protein overexpression and purification

Protein preparation for ReDCaT SPR was performed as follows. N-terminally 6×His-tagged CutR was expressed from the plasmid pET28a in *E. coli* Rosetta (BL21 DE3) pLysS competent cells. Overnight culture (40 mL) was used to inoculate 4 L of LB with selective antibiotics. Cultures were incubated at 37°C with shaking at 220 rpm until OD_600_ ~0.6, before isopropyl-β-d-1-thiogalactopyranoside was added to a final concentration of 1 mM. Cultures were incubated for a further 4 hours, then the cells were pelleted by centrifugation and stored at −80°C. Cell pellets were defrosted on ice for 30 min before being resuspended in 25 mL lysis buffer (20 mM Tris-HCl, pH 8.0, 75 mM NaCl, 0.1% Triton X-100, 10 mg/mL lysozyme, EDTA-free protease inhibitor) and incubated at room temperature for a further 30 min. Suspensions were sonicated on ice eight times at 50 Hz for 30 s per cycle, with 1 min off between each sonication cycle. Cell debris was removed by centrifugation at 20,600 × *g* for 30 min at 4°C and the supernatant filtered through a sterile 0.22 µm filter (Sartorius). Clarified lysate was loaded onto a 1 mL HisTrap HP column (Cytiva) pre-equilibrated with Buffer A (50 mM Tris-HCl pH 8.0, 200 mM NaCl, 5% glycerol). The loaded column was washed with increasing concentrations of imidazole (step 1: 10 mM; step 2: 20 mM; step 3: 30 mM), before the protein was eluted using 500 mM imidazole, all in the same buffer. Desired protein fractions were pooled and loaded onto a preparative-grade HiLoad 16/600 Superdex 200 pg gel filtration column (GE Healthcare) pre-equilibrated with gel filtration buffer (50 mM Tris-HCl, pH 8.0, 200 mM NaCl, 10% glycerol, filtered). Desired fractions were identified and analyzed for purity via SDS-PAGE before being pooled. Aliquots were flash-frozen in liquid nitrogen and stored at −80°C.

### ReDCaT SPR

Protein-DNA binding site scanning and footprinting was performed using ReDCaT SPR as previously described (16). Preliminary scanning was performed using promoter regions of interest divided into a series of overlapping double-stranded oligonucleotide probes (Table S2). The total length of each fragment was 40 bp including 15 bp overlaps. To create the double-stranded probes, each fragment was reverse-complemented and a ReDCaT biotinylated single-stranded linker added to the 3′ end as an overhang. For each fragment, DNA oligos (IDT) were resuspended to 100 µM in sterile distilled H_2_O, and 55 µL forward oligo was mixed with 45 µL reverse oligo. Oligos were annealed at 95°C for 5 min followed by ramping to 4°C at 0.1°C/s. The annealed DNA fragments were then diluted to a final concentration of 1 µM in HBS-EP+ buffer (150 mM NaCl, 3 mM EDTA, 0.05% vol/vol surfactant P20, 10 mM HEPES, pH 7.4). Positive hits from site scanning were selected for footprinting. Two base pair truncations were made from the 3′ end of the selected fragments and similarly from the 5′ end of the inverted fragment. All ReDCaT SPR experiments were performed using a single Sensor Chip SA (GE Healthcare) on a Biacore 8K SPR system (Cytiva) as follows. For each channel, FC_1_ was designated as the reference (FC_REF_) and FC_2_ as the test cell (FC_TEST_). DNA capture at 400 RUs was achieved in FC_TEST_ with a contact time of 60 s at a flow rate of 10 µL/min. Protein analyte (purified CutR; 2 µM or 1 µM) was then added over both FCs for 60 s at a flow rate of 50 µL/min followed by a buffer-only dissociation of 60 s. Regeneration of the chip was achieved using Regeneration buffer (1 M NaCl, 50 mM NaOH) injected for 60 s at a flow rate of 10 µL/min over both FCs. All experiments were run in duplicate, and data analysis was performed using the Biacore Insight Evaluation software (Cytiva) and Microsoft Excel.

### Tandem mass tagging proteomic analysis

Five microliters of spores of the relevant *S. venezuelae* strains were inoculated on cellophane-covered agar plates as triplicate spots in duplicate. After 2 or 9 day incubation at 30°C, the mycelium was scraped off the cellophanes into a 15 mL Falcon tube and resuspended in 10 mL cell lysis buffer (50 mM TEAB buffer, pH 8.0, 150 mM NaCl, 2% SDS, EDTA-free protease inhibitor, PhosSTOP phosphatase inhibitor). The suspension was disrupted via French press three times before being boiled at 100°C for 10 min. Samples were sonicated at 50 Hz four times for 20 s per cycle and then pelleted at 3,220 × *g* for 30 min. Protein concentration was determined using the BCA assay (Thermo Fisher Scientific), and 1 mg of protein from each sample was transferred to a fresh 15 mL Falcon tube. The proteins were precipitated from the supernatant with chloroform-methanol (43) and washed with acetone.

The air-dried pellets were dissolved in 50 µL–100 µL of 2.5% sodium deoxycholate (SDC; Merck). After quantification by BCA assay, 100 µg of protein was reduced, alkylated, and digested with trypsin in the presence of 0.2 M EPPS buffer (Merck) and 2.5% SDC according to standard procedures. After digestion, the SDC was precipitated by adjusting to 0.2% trifluoracetic acid (TFA), and the clarified supernatant was subjected to C18 solid-phase extraction (SPE; OMIX tips; Agilent). TMT labeling was performed using a Thermo TMT10plex kit (lot TL274393) according to the manufacturer’s instructions with slight modifications; the dried peptides were dissolved in 90 µL of 0.2 M EPPS buffer/10% acetonitrile, and 200 µg TMT10plex reagent dissolved in 22 µL of acetonitrile was added. Samples were assigned to the TMT channels in an order avoiding channel leakage between different samples, if possible, as detailed by Brenes et al. (44). After labeling, aliquots of 1.7 µL from each sample were combined and analyzed on the mass spectrometer (detailed below) to check labeling efficiency and estimate total sample abundances. The main sample aliquots were then combined correspondingly and desalted using a 50 mg C18 Sep-Pak cartridge (Waters). The eluted peptides were dissolved in 500 µL of 25 mM NH_4_HCO_3_ and fractionated by high-pH reversed-phase HPLC. Using an ACQUITY Arc Bio System (Waters), the samples were loaded to a Kinetex 5 µm EVO C18 100 Å LC Column 250 × 4.6 mm (Phenomenex). Fractionation was performed with the following gradient of solvents A (water), B (acetonitrile), and C (25 mM NH_4_HCO_3_) at a flow rate of 1 mL/min: solvent C was kept at 10% throughout the gradient; solvent B: 0–5 min: 5%, 5–10 min: 5%–13% curve 5, 10–70 min: 13%–40%, 70–75 min: 40%–50%, 75%–80%: 50%–80%; followed by 5 min at 80% B and re-equilibration to 5% for 24 min. Fractions of 1 mL were collected, dried down, and concatenated to produce 20 final fractions for MS analysis. Aliquots of 30 µL–40 µL were removed and stored at −80°C for global proteome quantification. Aliquots of all concatenated fractions were analyzed by nanoLC-MS/MS on an Orbitrap Fusion Tribrid mass spectrometer coupled to an UltiMate 3000 RSLCnano LC system (Thermo Fisher Scientific). The samples were loaded onto a trap column (nanoEase M/Z Symmetry C18 Trap Column, Waters) with 0.1% TFA at 15 µL/min for 3 min. The trap column was then switched in-line with the analytical column (nanoEase M/Z column, HSS C18 T3, 1.8 µm, 100 Å, 250 mm × 0.75 µm, Waters) for separation using the following gradient of solvents A (water, 0.1% formic acid) and B (80% acetonitrile, 0.1% formic acid) at a flow rate of 0.25 µL/min; for global protein analysis: 0–4 min 3% B (parallel to trapping); 4–17 min increase B curve 4 to 14%; 17–80 min linear increase B to 35%; 80–102 min linear increase B to 55% followed by a ramp to 99% B and re-equilibration to 3% B for 23 min; for enriched phosphopeptides: 0–4 min 3% B (parallel to trapping); 4–11 min increase B curve 4 to 12%; 11–52 min linear increase B to 43%; 52–61 min linear increase B to 55%, 61–64 min linear increase B to 65%, followed by a ramp to 99% B and re-equilibration to 3% B for 23 min. Data were acquired with the following parameters in positive ion mode: MS_1_/OT: resolution 120K, profile mode, mass range m/z 400–1,800, AGC target 2e5, max inject time 50 ms; MS_2_/IT: data-dependent analysis with the following parameters: top 10 in IT Turbo mode, centroid mode, quadrupole isolation window 1.6 Da, charge states 2–5, threshold 1.9e4, CE = 35, AGC target 2e4, max inject time 70 ms, dynamic exclusion 1 count/7 s with ± 7 ppm, multistage activation on with neutral loss 97.98 Da (for phosphopeptides only); MS_3_ synchronous precursor selection (SPS): 10 SPS precursors, isolation window 1.6 Da, HCD fragmentation with CE = 65, Orbitrap resolution 50K, AGC target 1.5e5, max inject time 175 ms.

The acquired raw data were processed and quantified in Proteome Discoverer (PD) 3.1 (Thermo Fisher Scientific); all mentioned tools of the following workflow are nodes of the proprietary PD software. The *Streptomyces venezuelae* (http://strepdb.streptomyces.org.uk/, 7,420 entries) protein FASTA database was imported into PD, adding a reversed sequence database for decoy searches; a database for common contaminants (maxquant.org, 245 entries) was also included. For global protein quantification, the database search was performed using the incorporated search engine CHIMERYS (MSAID, Munich, Germany). The processing workflow also included spectrum selection, the TopN Peak Filter with 20 peaks per 100 Da, and the reporter ion quantification by most confident centroid (20 ppm). The inferys_3.0.0. fragmentation prediction model was used with fragment tolerance of 0.5 Da, enzyme trypsin with one missed cleavage, variable modification oxidation (M), and fixed modifications carbamidomethyl (C) and TMT10plex on N-terminus and K. Identifications were calculated for false discovery rate (FDR) 0.01 (strict) and 0.05 (relaxed). The results were exported into a Microsoft Excel table including data for normalized and unnormalized abundances, ratios for the specified conditions, the corresponding *P*-values and adjusted *P*-values, number of unique peptides, q-values, PEP-values, identification scores from both search engines; FDR confidence filtered for high confidence (strict FDR 0.01) only. Further filtering included removal of contaminants and single unique peptide matches.

### Chemical extraction of chloramphenicol from solid media

Two 5 µL aliquots of spore stock were used to inoculate cellophane-covered 30 mL agar plates with *Streptomyces* spp. before incubation at 30°C for 2 days. Subsequently, the cellophane was removed. Agar was cut into 1 cm^3^ pieces using a sterile razor blade and transferred to a 100 mL Duran bottle. Thirty milliliters of HPLC-grade ethyl acetate was added to the agar, briefly shaken, and left at room temperature for 1 hour. The top 25 mL of the ethyl acetate was then transferred to a sterile 50 mL Falcon tube, and the sample was dried under vacuum. The sample was then resuspended in 500 µL HPLC-grade methanol and transferred to a 1.5 mL microcentrifuge tube. Insoluble particulates were removed via centrifugation at 15,871 × *g* for 10 min, with the uppermost 200 µL of solution transferred to a 2 mL HPLC vial containing a 200 µL glass insert for downstream chloramphenicol quantification.

### Chloramphenicol quantification by high-performance liquid chromatography

Analytical HPLC was performed on a 1290 Infinity II LC System (Agilent). A chloramphenicol standard curve was prepared in triplicate with serial dilutions of chloramphenicol in methanol from 1,000 to 0.01 µg/mL. Chloramphenicol standards and extracted samples were chromatographed over a Kinetex 2.6 µm XB-C18 110 Å, 100 × 4.6 mm column (Phenomenex) eluting with a linear gradient: mobile phase A, 0.1% (vol/vol) trifluoroacetic acid; mobile phase B, acetonitrile; 0 min, 10% B; 1 min, 10% B; 11 min, 100% B; 13 min, 100% B; 13.2 min, 10% B; 15 min, 10% B; flow rate 1 mL/min; injection volume 10 µL. UV absorbance was measured at 278 nm, and the peak area at this wavelength was measured for analysis. The minimum concentration of chloramphenicol that could accurately be detected under these conditions was 0.5 µg/mL.

### qRT-PCR

To perform qRT-PCR, *S. venezuelae* wild-type, ∆*cutRS,* Δ*htrA3*, and ∆*cutRS* + pIJ10257-*cutRS*(C85S,C103S) strains were grown on top of sterilized cellophane discs on YP or YPD agar at 30°C for 8–10 days. Colonies were removed from the cellophanes and kept in liquid nitrogen while being crushed using a sterile pestle and mortar on dry ice. Crushed samples were resuspended in 1 mL RLT Buffer (Qiagen) supplemented with 1% 2-mercaptoethanol. This suspension was added to a QIA-shredder column (Qiagen) with the flowthrough transferred to a new 1.5 mL microfuge tube (leaving the pellet). Then, 700 µL acidic phenol:chloroform was added, and the sample was incubated at room temperature for 3 min and subsequently centrifuged at 15,871 × *g* for 20 min. The resulting upper phase was mixed with 0.5 volumes 95% ethanol. This was then applied to an RNeasy Mini spin column (Qiagen) and processed following the manufacturer’s protocol. The eluant was treated with the Turbo-DNase kit (Invitrogen) followed by an RNeasy mini clean-up kit (Qiagen), both used according to the manufacturer’s instructions. The samples were then flash-frozen in liquid nitrogen for storage at −80°C. RNA was quantified with Nanodrop and Qubit Fluorometer, and ~1 microgram was converted to cDNA using the LunaScript RT SuperMix Kit (NEB) as per the manufacturer’s instructions, including No-RT control reactions. Primers for each transcript were optimized using serial dilutions of template DNA and checked for efficiency by generating standard curves and calculating as follows: E = (10^slope^ − 1)^-1^. Primer sets with an efficiency between 90 and 110% and within 5% of each other were selected. qRT-PCR was run using the QuantStudio1 (Applied Biosystems) with Luna Universal qPCR Master Mix according to the manufacturer’s guidelines for a 20 µL reaction with 2 µL template cDNA and a final concentration of 0.25 mM of each primer. Controls with no reverse transcriptase were run to ensure the absence of contaminating gDNA. ΔC_T_ values were normalized to the *hrdB* gene, which encodes the housekeeping RNA polymerase sigma factor.

### Phylogenetic analysis

Genomes for use in phylogenetic analysis were obtained from the NCBI database (45); in all cases, only complete and “reference” RefSeq genomes were selected to avoid duplication. The accession numbers for genomes used to generate Fig. 7A and 6B are given in Supplementary Information 1.1 and 1.2, respectively. Protein sequence alignments were carried out using MAFFT version 7 (mafft --memsavetree --retree 1 –treeout) (46, 47). Aligned sequences were used to produce a maximum-likelihood tree using FASTTREE (FastTree -jtt alignment_file > output_tree) (48). The resulting trees were annotated in Interactive Tree Of Life v.7 (49). Homologs of SvCutS and SvCssS were identified by reciprocal BLAST search, where reciprocal BLAST best hits were used in further analysis.

### Protein sequence alignments

Protein sequences for alignment were obtained via reciprocal blast searches using *S. venezuelae* GCF_001886595.1 CutS and CssS as the query sequences. The sequences obtained for CutS and CssS homologs are available in Supplemental Information 1.7 and 1.8 , respectively. Sequences were aligned with Clustal Omega (50) and visualized in Jalview (51).

## Data Availability

The ChIP-seq data generated in this study have been deposited in the GEO database under the accession code GSE225370. The proteomics data generated in this study have been deposited in the ProteomeXchange database with identifier PXD051851. The genome sequence accession numbers and protein identifiers used to generate the data in Fig. 7A and B available as Supplementary Information 1 and 2, respectively. Protein identifiers used to generate Fig. S2 and Fig. S4 are available as supplemental material.

## SUPPLEMENTAL MATERIAL

Figure S1 mbio.02991-25-s0001.pdf CutS alignment.

Figure S2 mbio.02991-25-s0002.eps CutS model.

Figure S3 mbio.02991-25-s0003.pdf HtrA3 phenotype.

Figure S4 mbio.02991-25-s0004.eps CssS alignment.

Supplemental text mbio.02991-25-s0005.docx NCBI RefSeq assembly genome accession numbers for the genomes used to generate Fig. 7A.

Table S1 mbio.02991-25-s0006.docx Bacterial strains and plasmids used in this study.

Table S2 mbio.02991-25-s0007.docx Oligonucleotide sequences used in this study.

Table S3 mbio.02991-25-s0008.docx Day 2 timepoint TMT-proteomics data.

Table S4 mbio.02991-25-s0009.docx Day 9 timepoint TMT-proteomics data.

ASM does not own the copyrights to Supplemental Material that may be linked to, or accessed through, an article. The authors have granted ASM a non-exclusive, world-wide license to publish the Supplemental Material files. Please contact the corresponding author directly for reuse.
