# Supplementary material for: The conserved two-component systems CutRS and CssRS control the protein secretion stress response in Streptomyces
Source: mBio. 2025 Dec 15;17(1):e02991-25. doi: 10.1128/mbio.02991-25 (PMC12802291; doi:10.1128/mbio.02991-25)
Supplement: Figure S1 — CutS alignment. [file mbio.02991-25-s0001.pdf]

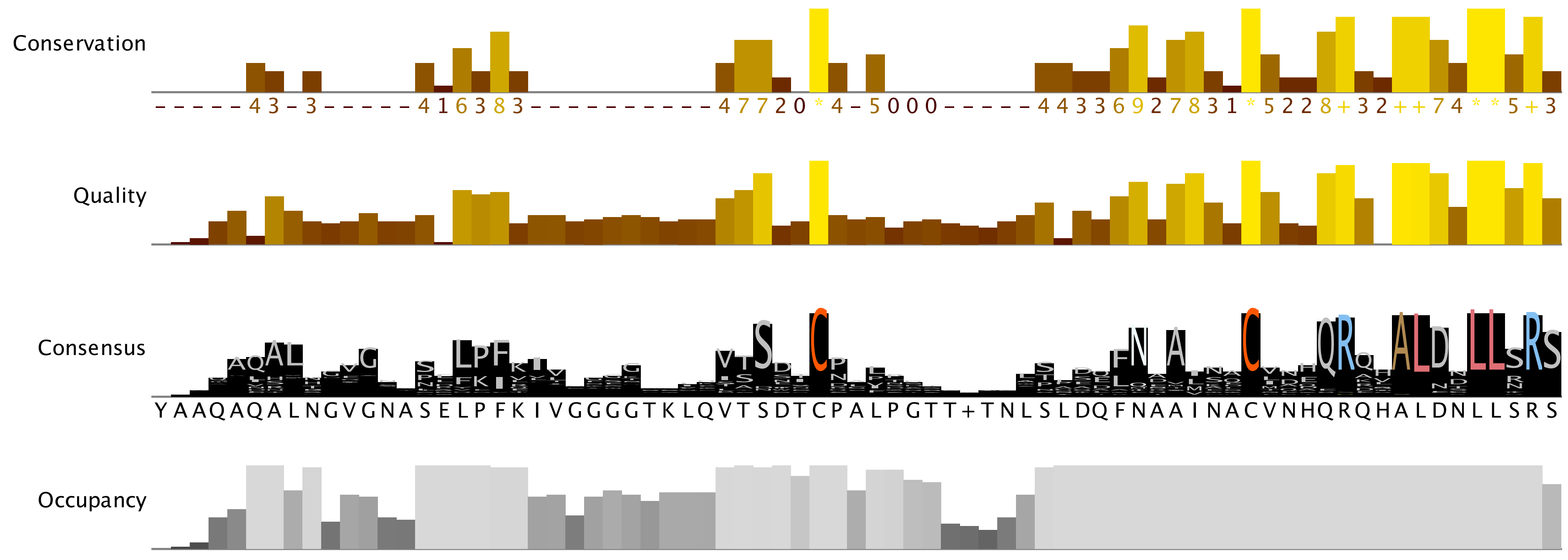

Supplementary Figure 1. | Residue conservation in the extracellular sensor domain of CutS. Clustal OMEGA alignment of the transmembrane domains, as predicted by DeepTMHMM, of 152 CutS homologues from *Streptomyces* spp., visualised in Jalview. The figure shows alignment conservation, quality, consensus, and occupancy across a section of the extracellular sensor domain, highlighting two highly conserved cysteine residues. The 'Conservation' track indicates how conserved each position is, with scores from 0 (variable) to 11 (fully conserved) shown beneath the bars. 'Quality' reflects the degree of conservative substitutions at each position based on the BLOSUM62 matrix. The 'Consensus' line shows the most common residue at each position; an asterisk (*) denotes 100% conservation, while a plus sign (+) indicates conserved physicochemical properties. 'Occupancy' represents the proportion of sequences without a gap at each position. Sequences used to generate this figure are provided in Supplementary Information 1.3.
