# Supplementary material for: The conserved two-component systems CutRS and CssRS control the protein secretion stress response in Streptomyces
Source: mBio. 2025 Dec 15;17(1):e02991-25. doi: 10.1128/mbio.02991-25 (PMC12802291; doi:10.1128/mbio.02991-25)
Supplement: Figure S3 — HtrA3 phenotype. [file mbio.02991-25-s0003.pdf]

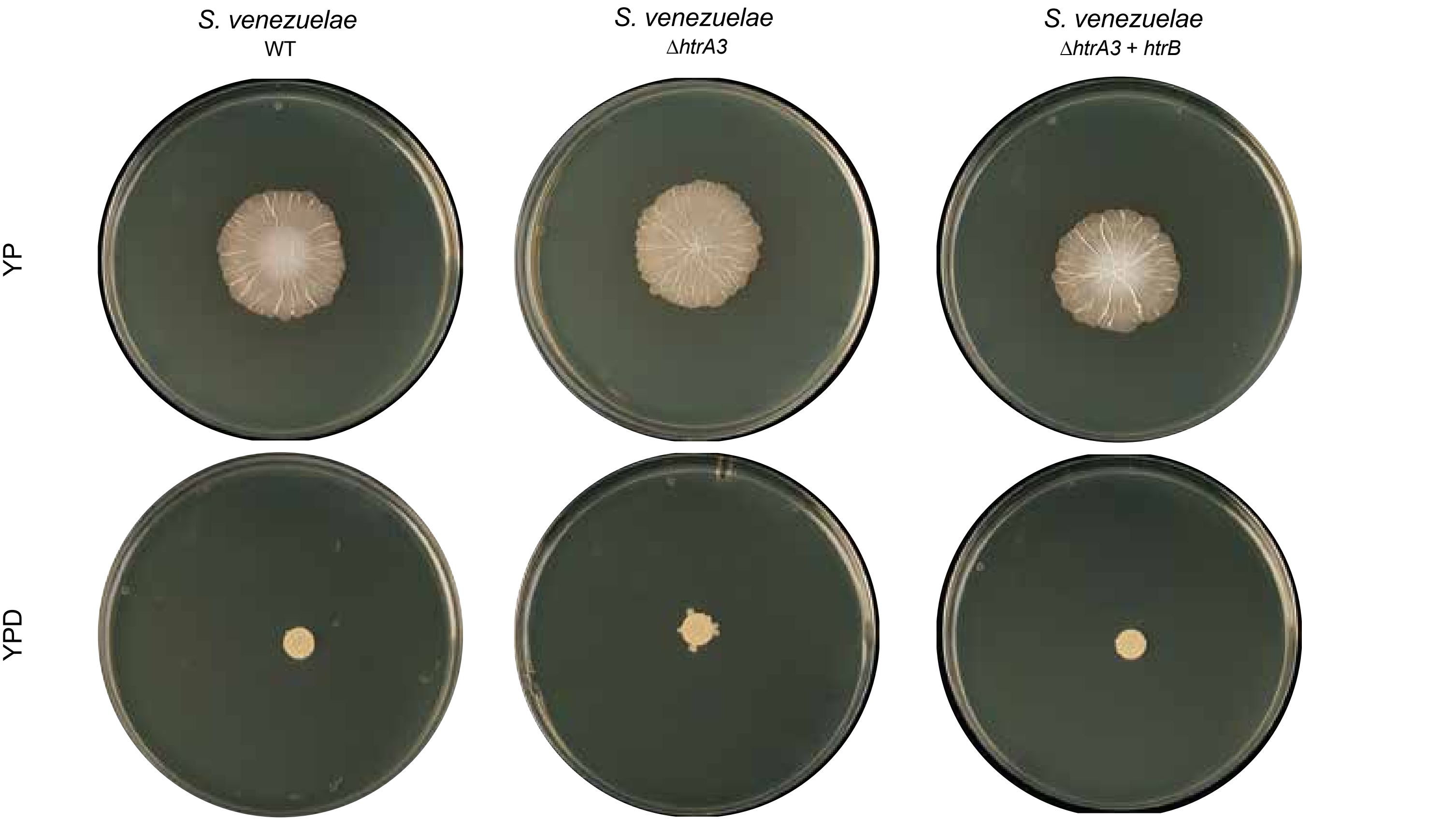

Supplementary Figure 3. | Overexpression of htrB in the *S. venezuelae ΔhtrA3* background is unable to reproduce the *ΔcutRS* phenotype. A. *S. venezuelae* wild-type, *ΔhtrA3* and *ΔhtrA3* with *htrB* overexpression colonies grown on YP (- glucose) and YPD (+ glucose) for 10 days at 30°C.
