# Supplementary material for: The conserved two-component systems CutRS and CssRS control the protein secretion stress response in Streptomyces
Source: mBio. 2025 Dec 15;17(1):e02991-25. doi: 10.1128/mbio.02991-25 (PMC12802291; doi:10.1128/mbio.02991-25)
Supplement: Supplemental text — NCBI RefSeq assembly genome accession numbers for the genomes used to generate Fig. 7A. [file mbio.02991-25-s0005.docx]

**Supplementary Information 1.1.** NCBI RefSeq assembly genome accession numbers for the genomes used to generate Figure 7A

GCA_002213425.1, GCA_025234665.1, GCA_000170895.1, GCA_004295665.1, GCA_000836725.1 GCA_003287285.1, GCA_015461805.1, GCA_014879295.1, GCA_016908115.1, GCA_014656215.1 GCA_014905135.1, GCA_001887285.1, GCA_028561565.1, GCA_004793545.1, GCA_002980595.1 GCA_009728465.1, GCA_000248055.2, GCA_014518315.1, GCA_003008555.2, GCA_000021545.1 GCA_902143375.2, GCA_000181875.2, GCA_007860015.1, GCA_900638485.1, GCA_002927045.1 GCA_014648935.1, GCA_007004545.3, GCA_000284415.2, GCA_004339465.1, GCA_900111795.1 GCA_001437465.1, GCA_003096875.1, GCA_014645235.1, GCA_004793975.1, GCA_000647975.1 GCA_005887635.2, GCA_002101675.1, GCA_018127725.1, GCA_012927345.1, GCA_003576475.1 GCA_009729555.1, GCA_016641735.1, GCA_016889925.1, GCA_001187435.1, GCA_004402625.1 GCA_001718555.1, GCA_012933555.1, GCA_003937805.1, GCA_014196795.1, GCA_009711205.1 GCA_003015165.1, GCA_900187255.1, GCA_011839805.1, GCA_001467895.1, GCA_004342885.1 GCA_007829885.1, GCA_013306565.1, GCA_016904945.1, GCA_900148505.1, GCA_022229015.1 GCA_000355675.1, GCA_001298445.1, GCA_009720725.1, GCA_024171895.1, GCA_002899695.1 GCA_009718825.1, GCA_002921145.1, GCA_013409505.1, GCA_900142685.1, GCA_000223985.2 GCA_003368535.1, GCA_008974285.1, GCA_000735425.1, GCA_003994235.2, GCA_000715015.1 GCA_002117085.1, GCA_010448835.1, GCA_000317575.1, GCA_004363035.1, GCA_003729985.1 GCA_002080125.1, GCA_018403085.1, GCA_002003665.1, GCA_003730065.1, GCA_900188045.1 GCA_023374055.1, GCA_014651375.1, GCA_002251775.1, GCA_000961515.1, GCA_902459775.1 GCA_000227685.3, GCA_006539585.1, GCA_900108015.1, GCA_014641455.1, GCA_001434335.1 GCA_004803895.1, GCA_000166275.1, GCA_000231365.2, GCA_015244675.1, GCA_020687245.1 GCA_001651875.1, GCA_021391315.1, GCA_002272015.1, GCA_900112575.1, GCA_001189035.1 GCA_003286915.1, GCA_002583405.1, GCA_002742285.1, GCA_011812965.1, GCA_004803695.1 GCA_018494065.1, GCA_014323405.1, GCA_000709415.1, GCA_000264455.2, GCA_007786215.1 GCA_004745525.1, GCA_007829035.1, GCA_000819905.1, GCA_012395835.1, GCA_003201285.1 GCA_900103425.1, GCA_023238325.1, GCA_012641465.1, GCA_010587385.1, GCA_900091505.1 GCA_020164485.1, GCA_014197035.1, GCA_017298635.1, GCA_007904085.1, GCA_018363015.1 GCA_900638015.1, GCA_004137685.1, GCA_014640315.1, GCA_900113035.1, GCA_017498585.1 GCA_014646335.1, GCA_028321995.1, GCA_009724245.1, GCA_003611455.1, GCA_900129405.1 GCA_004799285.1, GCA_022179405.1, GCA_002101705.1, GCA_009811555.1, GCA_029026145.1 GCA_017875645.1, GCA_014646375.1, GCA_003258595.1, GCA_000008185.1, GCA_001421015.2 GCA_016134045.1, GCA_014200045.1, GCA_003115815.1, GCA_017894385.1, GCA_022493915.1 GCA_900609045.1, GCA_900457375.1, GCA_900116545.1, GCA_900187365.1, GCA_004025845.1 GCA_003721515.1, GCA_009664975.1, GCA_007990635.1, GCA_003045775.1, GCA_937425535.1 GCA_004799345.1, GCA_004284595.1, GCA_016862435.1, GCA_008923265.1, GCA_016814695.1 GCA_001895085.1, GCA_002804165.1, GCA_003614435.1, GCA_003999335.1, GCA_003337435.1 GCA_009827295.1, GCA_900129915.1, GCA_013408435.1, GCA_001974985.1, GCA_014652395.1 GCA_001043915.1, GCA_005144635.2, GCA_021613355.1, GCA_003664585.1, GCA_013294265.1 GCA_000092825.1, GCA_019039255.1, GCA_000023325.1, GCA_000191145.1, GCA_002285905.1 GCA_003595195.1, GCA_000024285.1, GCA_014042345.1, GCA_014652515.1, GCA_003544895.1 GCA_003148565.1, GCA_016863515.1, GCA_028858425.1, GCA_900104265.1, GCA_009696375.1 GCA_013409575.1, GCA_900105445.1, GCA_003815915.1, GCA_001042675.1, GCA_004363955.1 GCA_008330805.1, GCA_017894365.1, GCA_002893765.1, GCA_018127985.1, GCA_900475855.1 GCA_019856495.1, GCA_003260125.1, GCA_000341895.1, GCA_004661485.1, GCA_019024325.1 GCA_004614195.1, GCA_014653195.1, GCA_002393505.1, GCA_015731765.1, GCA_006438845.1 GCA_001920325.1, GCA_005049045.1, GCA_013155425.1, GCA_014337235.1, GCA_001742285.1 GCA_013408095.1, GCA_001434405.1, GCA_011462075.1, GCA_900100765.1, GCA_900128985.1 GCA_000160535.1, GCA_011065485.1, GCA_017309995.1, GCA_019711655.1, GCA_006494535.1 GCA_000019725.1, GCA_003428865.1, GCA_000765945.1, GCA_004363775.1, GCA_014931715.1 GCA_022699665.1, GCA_003797885.1, GCA_006711645.1, GCA_013340285.1, GCA_001619895.1 GCA_009754945.1, GCA_000956575.1, GCA_003063455.1, GCA_000011965.2, GCA_010729665.1 GCA_004309735.1, GCA_002848365.2, GCA_900111785.1, GCA_019891175.1, GCA_023983615.1 GCA_900090295.1, GCA_003355515.1, GCA_000196355.1, GCA_003351765.1, GCA_002954445.1 GCA_001677435.1, GCA_016812185.1, GCA_900105315.1, GCA_001693775.2, GCA_025822605.1 GCA_020694005.1, GCA_001643015.1, GCA_002887555.1, GCA_014109745.1, GCA_900289205.1 GCA_900107675.1, GCA_018075365.1, GCA_007279655.1, GCA_017939705.1, GCA_000018865.1 GCA_014395225.1, GCA_900113675.1, GCA_008364555.1, GCA_013408145.1, GCA_900699765.1 GCA_002282915.1, GCA_002355295.1, GCA_013267435.1, GCA_016908415.1, GCA_000194605.1 GCA_002863865.1, GCA_000709915.1, GCA_003627965.1, GCA_018335475.1, GCA_003987255.1 GCA_002564025.1, GCA_014284255.1, GCA_004402975.1, GCA_900638215.1, GCA_900637025.1 GCA_018777385.1, GCA_014205085.1, GCA_000233695.3, GCA_004770475.1, GCA_021129235.1 GCA_014201905.1, GCA_022230955.1, GCA_002860125.1, GCA_004217095.1, GCA_001598055.1 GCA_011927825.1, GCA_004331915.1, GCA_003072485.1, GCA_900101095.1, GCA_013391475.1 GCA_003591515.1, GCA_002797575.1, GCA_023715655.1, GCA_014054985.1, GCA_003584645.1 GCA_005771565.1, GCA_900102145.1, GCA_001723285.1, GCA_900129485.1, GCA_025823105.1 GCA_900114055.1, GCA_000306885.1, GCA_005048225.1, GCA_014054965.1, GCA_017353515.1 GCA_000219045.1, GCA_025961205.1, GCA_022179245.1, GCA_003856865.1, GCA_021311115.1 GCA_008245065.1, GCA_900110165.1, GCA_009914215.1, GCA_001940005.1, GCA_021044825.1 GCA_027941775.1, GCA_013114825.1, GCA_001187595.1, GCA_009617755.1, GCA_000023905.1 GCA_003813775.1, GCA_005145005.1, GCA_013155125.1, GCA_000973085.1, GCA_011682075.1 GCA_003594885.1, GCA_004768525.1, GCA_000576555.1, GCA_002355155.1, GCA_004343255.1 GCA_017493175.3, GCA_001436375.1, GCA_007004645.1, GCA_000023565.1, GCA_014982715.1 GCA_002954715.1, GCA_900478135.1, GCA_010915725.1, GCA_004217115.1, GCA_002086125.1 GCA_019797925.1, GCA_900129555.1, GCA_003028295.1, GCA_900107685.1, GCA_000021945.1 GCA_902860155.1, GCA_014650575.1, GCA_009727065.1, GCA_023243115.1, GCA_003570935.1 GCA_003350525.1, GCA_013618625.1, GCA_014647975.1, GCA_905367715.1, GCA_023091965.1 GCA_006538525.1, GCA_018555385.1, GCA_000213255.1, GCA_002096055.1, GCA_018064205.1 GCA_014230055.1, GCA_900110885.1, GCA_005049235.1, GCA_001456255.1, GCA_002310835.1 GCA_011516835.1, GCA_000284115.1, GCA_008041945.1, GCA_016906185.1, GCA_900116765.1 GCA_003350455.1, GCA_020866845.1, GCA_900452865.1, GCA_003033925.1, GCA_002082195.1 GCA_000766865.1, GCA_012926525.1, GCA_028023875.1, GCA_000265075.1, GCA_014635565.1 GCA_003385115.1, GCA_000024805.1, GCA_003710065.1, GCA_900103215.1, GCA_000761485.1 GCA_028982105.1, GCA_002245625.1, GCA_002744735.1, GCA_009183655.1, GCA_006770285.1 GCA_003626755.1, GCA_009696315.1, GCA_003364265.1, GCA_014385405.1, GCA_900110265.1 GCA_004745635.1, GCA_019754295.1, GCA_012273655.1, GCA_014641695.1, GCA_011761605.1 GCA_000196855.1, GCA_004327275.1, GCA_004369205.1, GCA_016811235.1, GCA_009739655.1 GCA_018283675.1, GCA_015863265.1, GCA_014218335.1, GCA_013374815.1, GCA_900116135.1 GCA_900167945.1, GCA_020521235.1, GCA_011764445.1, GCA_023195895.1, GCA_900115715.1 GCA_000498575.2, GCA_002207865.1, GCA_003515685.1, GCA_015674855.1, GCA_012926505.1 GCA_001466665.1, GCA_004801295.1, GCA_000192575.1, GCA_009931375.1, GCA_000166055.1 GCA_001277175.1, GCA_012927355.1, GCA_000967425.1, GCA_003966335.1, GCA_007830115.1 GCA_900156415.1, GCA_900182725.1, GCA_014643835.1, GCA_014764405.1, GCA_000069785.1 GCA_004103715.1, GCA_000696345.2, GCA_020283705.1, GCA_021168825.1, GCA_007830175.1 GCA_900166965.1, GCA_001420645.1, GCA_016583825.1, GCA_001558415.2, GCA_013408405.1 GCA_010731295.1, GCA_000013885.1, GCA_900187395.1, GCA_004342905.1, GCA_014653945.1 GCA_001434985.1, GCA_001922385.1, GCA_004362695.1, GCA_000196795.1, GCA_001404935.1 GCA_025244745.1, GCA_900696085.2, GCA_021726475.1, GCA_003814705.1, GCA_008931845.1 GCA_900129025.1, GCA_900100345.1, GCA_013410405.1, GCA_007713715.1, GCA_007197755.1 GCA_020080045.1, GCA_003344785.1, GCA_900094625.1, GCA_001729805.1, GCA_003435485.1 GCA_004402705.1, GCA_000153125.2, GCA_027626975.1, GCA_000258405.1, GCA_024171725.1 GCA_004681965.1, GCA_019295435.1, GCA_014202415.1, GCA_008932115.1, GCA_900110135.1 GCA_001886735.1, GCA_001263395.1, GCA_003885045.1, GCA_900101415.1, GCA_022968135.1 GCA_003096195.1, GCA_009789655.1, GCA_002982075.1, GCA_018304805.1, GCA_019668465.1 GCA_018476885.1, GCA_001866055.1, GCA_017603605.1, GCA_900119185.1, GCA_901542405.1 GCA_005889745.1, GCA_013284055.1, GCA_000148645.1, GCA_017915135.1, GCA_004332295.1 GCA_001543245.1, GCA_010726505.1, GCA_004346195.1, GCA_001857965.1, GCA_000787715.1 GCA_001618865.1, GCA_002860725.1, GCA_900172275.1, GCA_025561465.1, GCA_020164555.1 GCA_014489595.1, GCA_014773275.1, GCA_003386865.1, GCA_900090285.1, GCA_900019265.2 GCA_001689125.2, GCA_008124835.1, GCA_004323735.1, GCA_014217765.1, GCA_014196955.1 GCA_001746755.1, GCA_014639155.1, GCA_900103775.1, GCA_002845745.1, GCA_000577275.2 GCA_001434135.1, GCA_013266735.1, GCA_029532055.1, GCA_001697225.1, GCA_010686755.1 GCA_016862775.1, GCA_016307205.1, GCA_016862035.1, GCA_000297025.1, GCA_011602365.1 GCA_000474745.1, GCA_010731795.1, GCA_003576345.1, GCA_020531915.1, GCA_003814695.1 GCA_017874355.1, GCA_900108045.1, GCA_014884995.1, GCA_009936215.1, GCA_014639535.1 GCA_008806375.1, GCA_900112705.1, GCA_009763245.1, GCA_900103285.1, GCA_000264295.1 GCA_024171815.1, GCA_002086115.1, GCA_001885945.1, GCA_000283915.1, GCA_016918545.1 GCA_008329645.1, GCA_000346505.1, GCA_002355475.1, GCA_000402035.1, GCA_008632335.1 GCA_021029445.1, GCA_008084905.1, GCA_020911865.1, GCA_025215075.1, GCA_013488225.1 GCA_027498395.1, GCA_011302055.1, GCA_000466965.1, GCA_017945865.1, GCA_009832765.1 GCA_007830185.1, GCA_019047805.1, GCA_000812875.1, GCA_016908355.1, GCA_016412875.1 GCA_004208515.1, GCA_900637515.1, GCA_014648155.1, GCA_000306235.2, GCA_003254545.1 GCA_009688945.1, GCA_001575195.1, GCA_018388445.1, GCA_009932515.1, GCA_008801715.1 GCA_023223645.1, GCA_014196525.1, GCA_000152725.1, GCA_007741535.1, GCA_900106595.1 GCA_014284085.1, GCA_001406295.1, GCA_900143075.1, GCA_900188325.1, GCA_003148495.1 GCA_900635955.1, GCA_022048975.1, GCA_010994755.2, GCA_001553005.1, GCA_004402485.1 GCA_009831115.1, GCA_001050115.1, GCA_003143535.1, GCA_016786255.1, GCA_001719065.1 GCA_019448115.1, GCA_003386205.1, GCA_003403015.1, GCA_000756815.1, GCA_000756615.1 GCA_002797915.1, GCA_003173735.1, GCA_000317515.1, GCA_024753145.1, GCA_000756655.1 GCA_004011095.1, GCA_013179735.1, GCA_016862395.1, GCA_003987095.1, GCA_024655835.1 GCA_004361835.1, GCA_019655015.1, GCA_000169155.1, GCA_000332495.2, GCA_016807805.1 GCA_000022905.1, GCA_900167465.1, GCA_002288185.1, GCA_004771005.1, GCA_003516145.1 GCA_900104945.1, GCA_000010405.1, GCA_003337555.1, GCA_001045435.1, GCA_900460315.1 GCA_003173715.1, GCA_023656515.1, GCA_006717145.1, GCA_007990505.1, GCA_003350925.1 GCA_009827495.1, GCA_001624335.1, GCA_004801285.1, GCA_014705945.2, GCA_014202075.1 GCA_015158295.1, GCA_000298235.1, GCA_009827655.1, GCA_001298525.1, GCA_004011835.1 GCA_022398355.1, GCA_000934565.1, GCA_008039555.1, GCA_016919165.1, GCA_003935895.2 GCA_001544575.2, GCA_001678905.1, GCA_004116265.1, GCA_003054025.1, GCA_002879615.1 GCA_025950235.1, GCA_900111735.1, GCA_000350105.1, GCA_902859915.1, GCA_900111075.1 GCA_028728065.1, GCA_000235405.3, GCA_012927745.1, GCA_009827455.1, GCA_003143695.2 GCA_001458195.1, GCA_900115165.1, GCA_900103005.1, GCA_016904675.1, GCA_000172555.1 GCA_004341235.1, GCA_002930645.1, GCA_003843835.1, GCA_018390645.1, GCA_003129465.1 GCA_901553735.1, GCA_001886615.1, GCA_900108535.1, GCA_001643235.1, GCA_022026155.1 GCA_009817885.1, GCA_000961095.1, GCA_001753165.1, GCA_900102695.1, GCA_001499615.1 GCA_003987275.1, GCA_000240185.2, GCA_018403665.1, GCA_003201715.1, GCA_014295035.1 GCA_006874605.1, GCA_001885995.1, GCA_024734075.1, GCA_003967535.1, GCA_025567525.1 GCA_918814755.1, GCA_018128965.1, GCA_014640555.1, GCA_019857225.1, GCA_009604385.1 GCA_001690755.1, GCA_001889005.1, GCA_019312585.1, GCA_009759665.1, GCA_014705605.1 GCA_026342395.1, GCA_900090305.1, GCA_000876225.1, GCA_000012485.1, GCA_015999265.1 GCA_016862215.1, GCA_000264315.1, GCA_017875555.1, GCA_004208635.1, GCA_001434835.1 GCA_000159015.1, GCA_013416325.1, GCA_014650895.1, GCA_900215605.1, GCA_004005865.1 GCA_014635585.1, GCA_022811645.1, GCA_003054005.1, GCA_910594985.1, GCA_000390185.1 GCA_013386885.1, GCA_001263175.1, GCA_004214795.1, GCA_019351435.1, GCA_019063885.1 GCA_000166415.1, GCA_009649075.1, GCA_002833365.1, GCA_000959505.1, GCA_023499985.1 GCA_002101815.1, GCA_019145375.1, GCA_019511525.1, GCA_900112895.1, GCA_004339625.1 GCA_013372225.1, GCA_009857655.1, GCA_001411475.1, GCA_900172375.1, GCA_004551575.1 GCA_000314895.2, GCA_007991295.1, GCA_014651915.1, GCA_014337175.1, GCA_003240565.2 GCA_012849555.1, GCA_903994045.1, GCA_003336205.1, GCA_029318685.1, GCA_023893635.1 GCA_004514435.1, GCA_009827305.1, GCA_014303955.1, GCA_900182685.1, GCA_001685435.3 GCA_003634235.1, GCA_000010525.1, GCA_014837105.1, GCA_003260095.1, GCA_000145035.1 GCA_009377195.2, GCA_000241055.1, GCA_900636505.1, GCA_000952155.1, GCA_022760755.1 GCA_000025965.1, GCA_008868535.1, GCA_003003005.1, GCA_002081995.1, GCA_003269065.1 GCA_014172405.1, GCA_003457605.1, GCA_900172265.1, GCA_900104035.1, GCA_027853235.1 GCA_001855565.1, GCA_016026395.1, GCA_007829335.1, GCA_009362815.1, GCA_000310185.1 GCA_009827445.1, GCA_000025905.1, GCA_016906305.1, GCA_010726085.1, GCA_004306555.1 GCA_003634315.1, GCA_000393995.1, GCA_024055635.1, GCA_003606285.1, GCA_018127765.1 GCA_011008945.1, GCA_014642635.1, GCA_001499835.1, GCA_014529935.1, GCA_004078635.1 GCA_010671565.1, GCA_003597125.1, GCA_003664325.1, GCA_017834145.1, GCA_001968985.1 GCA_002356335.1, GCA_016724805.1, GCA_014534645.1, GCA_900205755.1, GCA_007988805.1 GCA_013340845.1, GCA_014596975.1, GCA_001436705.1, GCA_012927275.1, GCA_001991075.2 GCA_002939865.1, GCA_002157875.1, GCA_001857945.1, GCA_000007905.1, GCA_000196455.1 GCA_900105385.1, GCA_004340805.1, GCA_001641285.1, GCA_023380205.1, GCA_014202645.1 GCA_009734445.1, GCA_027922425.1, GCA_019904175.1, GCA_000269865.1, GCA_005747095.1 GCA_900015215.1, GCA_002009335.2, GCA_003751635.1, GCA_900101825.1, GCA_900115825.1 GCA_001294335.1, GCA_000021685.1, GCA_001855615.1, GCA_018599195.1, GCA_900185615.1 GCA_001553625.1, GCA_004115155.1, GCA_014197795.1, GCA_004328535.1, GCA_003860075.1 GCA_004054105.1, GCA_900167305.1, GCA_017884005.1, GCA_022267555.1, GCA_014982855.1 GCA_024806925.1, GCA_001705175.1, GCA_000014505.1, GCA_002868715.1, GCA_000025725.1 GCA_002300555.2, GCA_006716645.1, GCA_014837045.1, GCA_002895005.1, GCA_020162115.1 GCA_020687825.1, GCA_003432065.1, GCA_002563715.1, GCA_009834205.1, GCA_000755145.1 GCA_018919145.1, GCA_001010355.1, GCA_003605755.1, GCA_002250945.2, GCA_007994225.1 GCA_005771635.1, GCA_002761755.1, GCA_018531195.1, GCA_002086635.1, GCA_022267515.1 GCA_904425415.1, GCA_900100175.1, GCA_000407605.1, GCA_021043605.1, GCA_014058425.1 GCA_012221945.1, GCA_018394375.1, GCA_003182475.1, GCA_004363245.1, GCA_018555685.1 GCA_004519295.1, GCA_005938385.1, GCA_001855385.1, GCA_009725985.1, GCA_001605725.1 GCA_009828065.1, GCA_003970795.1, GCA_010119935.1, GCA_003096355.1, GCA_003245365.1 GCA_007830615.1, GCA_002591785.1, GCA_005377625.1, GCA_004354045.1, GCA_001955655.1 GCA_003571485.1, GCA_000022525.1, GCA_019880585.1, GCA_010500845.1, GCA_007795095.1 GCA_900176035.1, GCA_000213655.1, GCA_003112675.1, GCA_900452715.1, GCA_905220995.1 GCA_000208385.1, GCA_009557235.1, GCA_900129925.1, GCA_900102905.1, GCA_001998325.1 GCA_009755785.1, GCA_001544555.2, GCA_003315795.1, GCA_015752125.1, GCA_016127755.1 GCA_000012725.1, GCA_011067745.1, GCA_023283885.1, GCA_011290685.1, GCA_003258335.1 GCA_900312995.1, GCA_012972705.2, GCA_014651075.1, GCA_003545815.1, GCA_003097575.1 GCA_900157385.1, GCA_900454465.1, GCA_900089775.1, GCA_005402965.1, GCA_021369635.1 GCA_023299185.1, GCA_014191495.1, GCA_004346685.1, GCA_018139545.1, GCA_014653275.1 GCA_902859625.1, GCA_900461525.1, GCA_019168305.1, GCA_004535825.1, GCA_009830285.1 GCA_002879715.1, GCA_001700435.1, GCA_004364645.1, GCA_001298555.1, GCA_006334995.2 GCA_013282215.1, GCA_000185145.2, GCA_903652925.1, GCA_003014735.1, GCA_000768345.1 GCA_010093345.1, GCA_013867715.1, GCA_004181775.1, GCA_003987515.1, GCA_013372165.1 GCA_003667905.1, GCA_003584125.1, GCA_009649955.1, GCA_016127475.1, GCA_018128265.1 GCA_900093605.1, GCA_900141995.1, GCA_007109405.1, GCA_007859955.1, GCA_001408515.1 GCA_000190755.3, GCA_900114675.1, GCA_011044775.1, GCA_023035875.1, GCA_006715085.1 GCA_010669145.1, GCA_021228795.1, GCA_004137245.1, GCA_003254275.1, GCA_003011925.2 GCA_004358185.1, GCA_005877035.1, GCA_004016505.1, GCA_005144425.2, GCA_014644495.1 GCA_001046655.1, GCA_001047375.1, GCA_016653115.1, GCA_000447675.1, GCA_008932075.1 GCA_004328865.1, GCA_000473995.1, GCA_009996845.1, GCA_022489035.1, GCA_019670005.1 GCA_014203805.1, GCA_017873095.1, GCA_008365275.1, GCA_002262875.1, GCA_001270025.1 GCA_905120465.1, GCA_003268815.1, GCA_001879105.1, GCA_013760845.1, GCA_014237865.1 GCA_002251835.1, GCA_004014775.2, GCA_002288285.1, GCA_900100625.1, GCA_010730195.1 GCA_020521295.1, GCA_018861705.1, GCA_900109785.1, GCA_024749365.1, GCA_000021825.1 GCA_917563915.1, GCA_000359745.1, GCA_000315365.1, GCA_014697095.1, GCA_014650735.1 GCA_001723385.1, GCA_900091625.1, GCA_002252475.1, GCA_019343195.1, GCA_020885855.1 GCA_025995175.1, GCA_000695095.2, GCA_011761495.1, GCA_020459125.1, GCA_020539505.1 GCA_900110915.1, GCA_002741015.1, GCA_004794175.1, GCA_000238395.4, GCA_016908645.1 GCA_014652795.1, GCA_003172895.1, GCA_017352135.1, GCA_023195885.1, GCA_900115345.1 GCA_016028735.1, GCA_914590485.1, GCA_013744875.1, GCA_001888185.1, GCA_004770745.1 GCA_019703875.1, GCA_900637165.1, GCA_900007165.2, GCA_004310325.1, GCA_000284515.1 GCA_020792675.1, GCA_012849055.1, GCA_000834295.1, GCA_016908015.1, GCA_001022135.1 GCA_000224675.1, GCA_014201115.1, GCA_019457945.1, GCA_003026455.1, GCA_001998965.1 GCA_001768675.1, GCA_902702935.1, GCA_001045465.1, GCA_028561275.1, GCA_900215245.1 GCA_003337735.1, GCA_024205225.1, GCA_000143845.1, GCA_014638845.1, GCA_009674495.1 GCA_001399455.2, GCA_003160695.1, GCA_008727875.1, GCA_000949865.1, GCA_021044775.1 GCA_001584325.1, GCA_024029595.1, GCA_002013915.1, GCA_024125445.1, GCA_002086795.1 GCA_008274805.1, GCA_004104355.1, GCA_017894345.1, GCA_000342045.1, GCA_014192155.1 GCA_024172095.1, GCA_004121055.1, GCA_001701045.1, GCA_024160205.1, GCA_014698375.1 GCA_000758725.1, GCA_900141915.1, GCA_000091325.1, GCA_016427565.1, GCA_002706795.1 GCA_003797185.1, GCA_003076595.1, GCA_016028295.1, GCA_001816165.1, GCA_002968995.1 GCA_002632615.1, GCA_015461835.1, GCA_900128865.1, GCA_002897375.1, GCA_002861485.1 GCA_024171995.1, GCA_007829995.1, GCA_900102195.1, GCA_000242915.2, GCA_900105935.1 GCA_001886225.1, GCA_022674245.1, GCA_003583985.1, GCA_011189505.1, GCA_001444445.1 GCA_014637955.1, GCA_011808225.1, GCA_007993035.1, GCA_002288825.1, GCA_000153425.1 GCA_001611555.1, GCA_004307995.1, GCA_900453875.1, GCA_003752405.1, GCA_014243445.1 GCA_014652715.1, GCA_003590945.1, GCA_009377205.1, GCA_902459655.1, GCA_011067105.1 GCA_006438965.1, GCA_014192375.1, GCA_024006985.1, GCA_014705925.2, GCA_017939745.1 GCA_001718335.1, GCA_006517755.1, GCA_900102035.1, GCA_004216695.1, GCA_001971745.1 GCA_000754995.1, GCA_003665435.1, GCA_010731875.1, GCA_013467635.1, GCA_018398935.1 GCA_004769235.1, GCA_000739045.1, GCA_900106035.1, GCA_004769295.1, GCA_016724885.1 GCA_003116135.1, GCA_900101985.1, GCA_001720135.1, GCA_900105375.1, GCA_003851125.1 GCA_014467015.1, GCA_014650755.1, GCA_900142255.1, GCA_900604295.1, GCA_022678645.1 GCA_900163835.1, GCA_018596335.1, GCA_005864225.1, GCA_900115805.1, GCA_900142375.1 GCA_001294575.1, GCA_004053875.1, GCA_014204325.1, GCA_002776695.1, GCA_007786475.1 GCA_000834455.1, GCA_000768355.1, GCA_000768705.1, GCA_009669325.1, GCA_000177535.2 GCA_002813205.1, GCA_000161615.1, GCA_002976435.1, GCA_900445245.1, GCA_003751385.1 GCA_004327225.1, GCA_008152325.1, GCA_022699385.1, GCA_002077945.1, GCA_004681125.1 GCA_003346755.1, GCA_004340665.1, GCA_900109455.1, GCA_009299485.1, GCA_011758745.1 GCA_024971755.1, GCA_017948305.1, GCA_900099915.1, GCA_900461125.1, GCA_003687485.1 GCA_004346865.1, GCA_001027025.1, GCA_022180365.1, GCA_004745825.1, GCA_001636015.1 GCA_916098265.1, GCA_001707835.1, GCA_001746855.1, GCA_022809125.1, GCA_015594845.1 GCA_000331715.1, GCA_016863295.1, GCA_900101285.1, GCA_002206635.1, GCA_001640115.1 GCA_014656115.1, GCA_003226565.1, GCA_019084045.1, GCA_008386695.1, GCA_001465255.1 GCA_011762095.1, GCA_014269025.2, GCA_000024905.1, GCA_008632615.1, GCA_022200785.1 GCA_001832905.1, GCA_019175365.1, GCA_000803215.1, GCA_003583925.1, GCA_004802515.1 GCA_900637075.1, GCA_019931735.1, GCA_004786035.1, GCA_021215455.1, GCA_027922365.1 GCA_900445235.1, GCA_004339595.1, GCA_016865215.1, GCA_002847445.1, GCA_000829235.1 GCA_016495725.1, GCA_004135055.1, GCA_013179805.1, GCA_002563695.1, GCA_002811765.1 GCA_006493595.1, GCA_000263675.2, GCA_000736415.1, GCA_026344005.1, GCA_002192415.1 GCA_008634015.1, GCA_900142205.1, GCA_900100295.1, GCA_015074805.1, GCA_001936235.1 GCA_017874075.1, GCA_020790155.1, GCA_008831075.1, GCA_004331415.1, GCA_003403035.1 GCA_004366595.1, GCA_010728325.1, GCA_016583745.1, GCA_020341435.1, GCA_900089835.1 GCA_018304825.1, GCA_025822765.1, GCA_010725485.1, GCA_014836875.1, GCA_001267955.1 GCA_900142885.1, GCA_900172285.1, GCA_009696615.1, GCA_001458215.1, GCA_003985155.1 GCA_900107235.1, GCA_900142955.1, GCA_027587225.1, GCA_016698645.1, GCA_000092505.1 GCA_002744715.1, GCA_900497695.1, GCA_013488205.1, GCA_006385705.1, GCA_001702265.1 GCA_900113435.1, GCA_002286955.1, GCA_020616335.1, GCA_014202115.1, GCA_023487995.1 GCA_028435365.1, GCA_000968535.1, GCA_000768685.1, GCA_900188395.1, GCA_000189255.2 GCA_900176045.1, GCA_014384895.1, GCA_019308285.1, GCA_008330085.1, GCA_006228125.1 GCA_000236665.1, GCA_900100755.1, GCA_900215655.1, GCA_000348905.1, GCA_000265425.1 GCA_003065605.1, GCA_013307225.1, GCA_003730015.1, GCA_004327985.1, GCA_000284335.1 GCA_014643175.1, GCA_001718535.1, GCA_000320365.1, GCA_012113595.1, GCA_014205135.1 GCA_003860585.1, GCA_004794105.1, GCA_002374835.1, GCA_011043685.1, GCA_000816185.1 GCA_004138395.1, GCA_000204255.1, GCA_014306105.1, GCA_012849215.1, GCA_001027925.1 GCA_009696265.1, GCA_900094945.1, GCA_009793355.1, GCA_002277935.1, GCA_021190955.1 GCA_005885635.1, GCA_900091415.1, GCA_002087085.1, GCA_901538385.1, GCA_014698995.1 GCA_017939625.1, GCA_014384765.1, GCA_007859635.1, GCA_016623605.1, GCA_019008365.1 GCA_009176325.1, GCA_023038235.1, GCA_002119845.1, GCA_900115585.1, GCA_015207485.1 GCA_003991895.1, GCA_003385955.1, GCA_002290025.1, GCA_019916085.1, GCA_021277265.1 GCA_000828125.2, GCA_010587025.1, GCA_900184815.1, GCA_000014865.1, GCA_014174395.1 GCA_900104965.1, GCA_003315635.1, GCA_001535985.1, GCA_000576425.1, GCA_016132545.1 GCA_000222605.2, GCA_003688405.1, GCA_021403115.1, GCA_000016845.1, GCA_002557795.1 GCA_025318105.1, GCA_001436115.1, GCA_014201615.1, GCA_009913435.1, GCA_017313765.1 GCA_008933115.1, GCA_900176375.1, GCA_000758705.1, GCA_006539865.1, GCA_014635145.1 GCA_014698305.1, GCA_900176405.1, GCA_024055725.1, GCA_003007675.1, GCA_001017175.1 GCA_004216855.1, GCA_003860565.1, GCA_019203985.1, GCA_015645465.1, GCA_022749495.1 GCA_014654785.1, GCA_014636335.1, GCA_012648005.1, GCA_013388295.1, GCA_001437695.1 GCA_014836545.1, GCA_003003795.1, GCA_014253065.1, GCA_001485475.2, GCA_000801145.1 GCA_001686985.1, GCA_018278905.1, GCA_900110815.1, GCA_004122105.1, GCA_008124875.1 GCA_001611155.1, GCA_008370245.1, GCA_003933735.1, GCA_007994185.1, GCA_016925655.1 GCA_900115105.1, GCA_001455085.1, GCA_004343075.1, GCA_003122385.1, GCA_004329815.1 GCA_007989425.1, GCA_900454435.1, GCA_002211225.1, GCA_004343195.1, GCA_002927275.1 GCA_020889785.1, GCA_015679325.1, GCA_000473765.1, GCA_014873145.1, GCA_000757385.1 GCA_000012525.1, GCA_000224985.1, GCA_002237775.1, GCA_019141525.1, GCA_900114925.1 GCA_003208175.1, GCA_002021235.1, GCA_014337195.1, GCA_007859915.1, GCA_006716865.1 GCA_014650595.1, GCA_002014825.1, GCA_004216895.1, GCA_004310425.1, GCA_022558445.1 GCA_004319545.1, GCA_900114795.1, GCA_900239975.1, GCA_013409935.1, GCA_000953855.3 GCA_013408565.1, GCA_014522205.1, GCA_000967245.1, GCA_017315345.1, GCA_001735765.2 GCA_000341355.1, GCA_900100775.1, GCA_005116475.1, GCA_011516875.1, GCA_000009945.1 GCA_014649035.1, GCA_004359005.1, GCA_003999585.1, GCA_008694105.1, GCA_003217215.1 GCA_001684975.1, GCA_002142475.1, GCA_011059105.1, GCA_001975955.2, GCA_014199945.1 GCA_014195655.1, GCA_900142765.1, GCA_900167435.1, GCA_001654835.1, GCA_014651755.1 GCA_000006985.1, GCA_015159745.1, GCA_020521275.1, GCA_004137795.1, GCA_001434055.1 GCA_003003265.1, GCA_003363015.1, GCA_015482585.1, GCA_900459485.1, GCA_902153245.3 GCA_011604665.1, GCA_018885085.1, GCA_900215315.1, GCA_900109705.1, GCA_902729405.1 GCA_019511345.1, GCA_014701115.1, GCA_003233695.1, GCA_001632775.1, GCA_008365295.1 GCA_900104365.1, GCA_900104915.1, GCA_025567605.1, GCA_900116065.1, GCA_900128925.1 GCA_902459565.1, GCA_900445255.1, GCA_900445875.1, GCA_024582835.1, GCA_001467055.1 GCA_001584225.1, GCA_014596945.1, GCA_003814815.1, GCA_003386275.1, GCA_000208405.1 GCA_000244955.1, GCA_000511385.1, GCA_000325705.1, GCA_001298465.1, GCA_002860805.1 GCA_002959895.1, GCA_009754905.2, GCA_900096585.1, GCA_900107145.1, GCA_900302475.1 GCA_010731615.1, GCA_002208805.2, GCA_019457915.1, GCA_009696065.1, GCA_001856685.1 GCA_000980815.1, GCA_000525915.1, GCA_014646355.1, GCA_014635705.1, GCA_002056725.1 GCA_009687845.1, GCA_020554845.1, GCA_004364345.1, GCA_900107405.1, GCA_001400735.1 GCA_001687665.2, GCA_001483865.1, GCA_013266695.1, GCA_014195545.1, GCA_003046185.1 GCA_021646685.1, GCA_019923725.1, GCA_900489485.1, GCA_000817955.1, GCA_014205895.1 GCA_900142295.1, GCA_900111555.1, GCA_900163565.1, GCA_024134545.1, GCA_021049305.1 GCA_009866805.1, GCA_000348685.1, GCA_002157855.1, GCA_004366735.1, GCA_004284785.1 GCA_003351175.1, GCA_023746535.1, GCA_004912155.1, GCA_900128905.1, GCA_008698235.1 GCA_009908315.1, GCA_002150005.2, GCA_900174585.1, GCA_002940045.1, GCA_017814275.1 GCA_901538265.1, GCA_000146675.1, GCA_002994635.1, GCA_014069315.1, GCA_014203155.1 GCA_003855155.1, GCA_004342175.1, GCA_020329495.1, GCA_004339025.1, GCA_900156655.1 GCA_014644275.1, GCA_000478195.2, GCA_002027705.1, GCA_014385095.1, GCA_900114645.1 GCA_003626895.1, GCA_016860545.1, GCA_004361915.1, GCA_003703835.1, GCA_004349225.1 GCA_018972165.1, GCA_004135625.1, GCA_014174215.1, GCA_004337445.1, GCA_017654485.1 GCA_003337485.1, GCA_004912145.1, GCA_016758195.1, GCA_020541885.1, GCA_006337165.1 GCA_000550805.1, GCA_001467615.1, GCA_900116575.1, GCA_002834225.1, GCA_022179125.1 GCA_018069755.1, GCA_900454815.1, GCA_000023545.1, GCA_013374215.1, GCA_023283765.1 GCA_003858455.1, GCA_008691045.1, GCA_014656275.1, GCA_009649735.1, GCA_000600005.1 GCA_004349355.1, GCA_008365385.1, GCA_022539405.1, GCA_000318215.2, GCA_014323705.1 GCA_001278705.1, GCA_016722785.1, GCA_000024385.1, GCA_004421025.1, GCA_000746025.2 GCA_010686705.1, GCA_003385775.1, GCA_007989725.1, GCA_900114825.1, GCA_000155735.2 GCA_018687955.1, GCA_003634455.1, GCA_024808875.1, GCA_004519335.1, GCA_018777395.1 GCA_003696285.1, GCA_009731575.1, GCA_000300275.1, GCA_000763575.1, GCA_001514055.1 GCA_900113045.1, GCA_009932395.1, GCA_004340905.1, GCA_021183645.1, GCA_003311965.1 GCA_003076475.1, GCA_019037185.1, GCA_003151135.1, GCA_004348415.1, GCA_014885015.1 GCA_018789675.1, GCA_001656035.1, GCA_007830515.1, GCA_004770105.1, GCA_002847605.1 GCA_001866665.1, GCA_001718955.1, GCA_020831405.1, GCA_001523705.1, GCA_024499525.1 GCA_014268485.2, GCA_014199295.1, GCA_002798015.1, GCA_007362775.1, GCA_003855395.1 GCA_902498735.1, GCA_000974765.1, GCA_003096835.1, GCA_009362845.1, GCA_002797535.1 GCA_900105105.1, GCA_003384725.1, GCA_019139895.1, GCA_900112165.1, GCA_001306415.1 GCA_006770265.1, GCA_000808575.1, GCA_014322845.1, GCA_000603945.1, GCA_003057965.1 GCA_004122095.1, GCA_007859735.1, GCA_014650995.1, GCA_003703475.1, GCA_016031615.1 GCA_003121925.1, GCA_008123425.1, GCA_026343945.1, GCA_019061205.1, GCA_003288115.1 GCA_001050475.1, GCA_010731775.1, GCA_002906155.1, GCA_001458275.1, GCA_016907495.1 GCA_012641365.1, GCA_002000365.1, GCA_006539345.1, GCA_001651865.1, GCA_000403375.2 GCA_013372105.1, GCA_020564935.1, GCA_006538565.1, GCA_000445105.1, GCA_000749845.1 GCA_014193425.1, GCA_900142415.1, GCA_014306135.1, GCA_008728835.1, GCA_014637085.1 GCA_005860795.2, GCA_900101385.1, GCA_025822555.1, GCA_001274935.1, GCA_003851725.1 GCA_900177425.1, GCA_003865375.1, GCA_900107255.1, GCA_009695815.1, GCA_009193305.1 GCA_000195975.1, GCA_001306035.1, GCA_026343995.1, GCA_000055785.1, GCA_002115755.1 GCA_006716095.1, GCA_014784055.1, GCA_022631375.1, GCA_016834455.1, GCA_016583645.1 GCA_005871085.1, GCA_000146065.1, GCA_000143965.1, GCA_025060755.1, GCA_900537995.1 GCA_000179555.1, GCA_003264475.1, GCA_000240225.2, GCA_020859625.1, GCA_013449735.1 GCA_900323515.1, GCA_907177275.1, GCA_023733635.1, GCA_001436295.1, GCA_900094545.1 GCA_009696145.1, GCA_900167165.1, GCA_003201975.1, GCA_002549795.1, GCA_006542645.1 GCA_002812225.1, GCA_003857055.1, GCA_900186025.1, GCA_003957295.1, GCA_014650815.1 GCA_001650025.1, GCA_010731655.1, GCA_900142235.1, GCA_000710775.1, GCA_013978595.1 GCA_002954605.1, GCA_004348445.1, GCA_016862855.1, GCA_017676345.1, GCA_004792415.1 GCA_014642835.1, GCA_000295935.2, GCA_021491355.1, GCA_001553605.1, GCA_002102185.1 GCA_009650135.1, GCA_900115175.1, GCA_900090265.1, GCA_000313175.2, GCA_000590925.1 GCA_024626525.1, GCA_002952315.1, GCA_019929665.1, GCA_006912135.1, GCA_000007205.1 GCA_009906315.1, GCA_007989805.1, GCA_004791695.1, GCA_015751765.1, GCA_900105115.1 GCA_018582665.1, GCA_900116515.1, GCA_004614185.1, GCA_019991125.1, GCA_014635905.1 GCA_004366355.1, GCA_000763135.2, GCA_001976145.1, GCA_003385395.1, GCA_007989895.1 GCA_009909235.1, GCA_016772315.1, GCA_028768405.1, GCA_000025485.1, GCA_001543125.1 GCA_014650335.1, GCA_900102135.1, GCA_001767675.1, GCA_006065315.1, GCA_000023705.1 GCA_007990365.1, GCA_014836505.1, GCA_900105425.1, GCA_005938655.1, GCA_003583725.1 GCA_903642095.2, GCA_007859305.1, GCA_009811595.1, GCA_000993785.3, GCA_900142755.1 GCA_004135365.1, GCA_003964805.1, GCA_000146345.1, GCA_003591035.1, GCA_026343615.1 GCA_003054045.1, GCA_003605385.2, GCA_001990805.3, GCA_000012945.1, GCA_003818135.1 GCA_009740285.1, GCA_003957745.1, GCA_003202235.1, GCA_024707545.1, GCA_011745665.1 GCA_000163895.2, GCA_001403795.1, GCA_002085925.2, GCA_013385175.1, GCA_007970665.1 GCA_021043325.1, GCA_014637725.1, GCA_022175585.1, GCA_014647315.1, GCA_006715095.1 GCA_015690355.1, GCA_003001955.1, GCA_018882205.1, GCA_001008345.1, GCA_003259615.1 GCA_019264345.1, GCA_004522065.1, GCA_025566025.1, GCA_001701025.1, GCA_900156505.1 GCA_000724775.3, GCA_021168665.1, GCA_020171285.1, GCA_003034225.1, GCA_001904725.1 GCA_000021705.1, GCA_900231165.1, GCA_009754915.1, GCA_001534645.1, GCA_013366925.1 GCA_008692035.1, GCA_000812025.2, GCA_002259605.1, GCA_003336665.1, GCA_000010605.1 GCA_027214225.1, GCA_007713705.1, GCA_004801125.1, GCA_018919385.1, GCA_011392075.1 GCA_004771275.1, GCA_014636775.1, GCA_004005485.1, GCA_020164535.1, GCA_003634015.1 GCA_014192105.1, GCA_902500215.1, GCA_000015485.1, GCA_000214175.1, GCA_013371495.1 GCA_001021045.1, GCA_014842875.1, GCA_000006925.2, GCA_003063295.1, GCA_008085455.1 GCA_009933255.1, GCA_008831155.1, GCA_020025155.1, GCA_016522065.1, GCA_002252505.1 GCA_004682055.1, GCA_017573505.1, GCA_900460155.1, GCA_004563965.1, GCA_015291705.1 GCA_014169735.1, GCA_003977685.1, GCA_000169415.1, GCA_006716205.1, GCA_008297955.1 GCA_002846615.1, GCA_024346585.1, GCA_004362535.1, GCA_001756855.1, GCA_003990045.1 GCA_006265165.1, GCA_003350405.1, GCA_010820565.1, GCA_000770585.1, GCA_019655335.1 GCA_003583935.1, GCA_005280195.1, GCA_000024205.1, GCA_011064285.1, GCA_004765605.1 GCA_007050985.1, GCA_006351005.1, GCA_007713685.1, GCA_900142455.1, GCA_014651715.1 GCA_001025155.1, GCA_027570065.1, GCA_002950575.1, GCA_018448965.1, GCA_900476375.1 GCA_013694245.1, GCA_000262305.1, GCA_000498655.1, GCA_009735685.1, GCA_021532655.1 GCA_900637665.1, GCA_009078285.1, GCA_024297065.1, GCA_007992415.1, GCA_003021615.1 GCA_003097495.1, GCA_001400515.1, GCA_000265505.1, GCA_003129485.1, GCA_000178095.1 GCA_900109185.1, GCA_003857035.1, GCA_003326475.1, GCA_019052655.1, GCA_004153445.1 GCA_006151975.2, GCA_003696315.1, GCA_013248975.1, GCA_020165855.1, GCA_000196535.1 GCA_900168155.1, GCA_020682665.1, GCA_010667615.1, GCA_017813335.1, GCA_011455695.1 GCA_002924365.1, GCA_000394055.1, GCA_001723605.1, GCA_001192835.1, GCA_900566065.1 GCA_900108175.1, GCA_008386585.1, GCA_024160185.1, GCA_008369935.1, GCA_900156265.1 GCA_012241385.1, GCA_000159995.1, GCA_026343855.1, GCA_014196765.1, GCA_003710805.1 GCA_017167965.1, GCA_000073005.1, GCA_015751755.1, GCA_003999255.1, GCA_000027145.1 GCA_000737865.1, GCA_010119545.1, GCA_003952005.1, GCA_013201825.1, GCA_010667575.1 GCA_000376605.1, GCA_003574215.1, GCA_001996185.1, GCA_004349145.1, GCA_014530645.1 GCA_003259955.1, GCA_900104735.1, GCA_014647915.1, GCA_010729895.1, GCA_012222965.1 GCA_003046585.1, GCA_007752345.1, GCA_009939295.1, GCA_017354945.1, GCA_014646255.1 GCA_003177055.1, GCA_002201475.1, GCA_002844575.1, GCA_010723225.1, GCA_000196615.1 GCA_003596335.1, GCA_006151805.1, GCA_003312425.1, GCA_014197785.1, GCA_003337715.1 GCA_022179085.1, GCA_006364455.1, GCA_003609995.1, GCA_021596945.1, GCA_900176425.1 GCA_003966655.1, GCA_014640395.1, GCA_003674065.1, GCA_002287885.2, GCA_004355225.1 GCA_025527015.1, GCA_010731575.1, GCA_000023285.1, GCA_002358085.1, GCA_008124615.1 GCA_011455875.1, GCA_007991755.1, GCA_007991695.1, GCA_900101585.1, GCA_020621385.1 GCA_019038595.1, GCA_001543175.1, GCA_014306455.1, GCA_001518015.1, GCA_014643615.1 GCA_017569925.1, GCA_013359825.1, GCA_905142465.1, GCA_025917275.1, GCA_003019295.1 GCA_008803015.1, GCA_018917555.1, GCA_000758165.1, GCA_001434255.1, GCA_018271995.1 GCA_019042115.1, GCA_014779795.1, GCA_900113335.1, GCA_002797555.1, GCA_023520795.1 GCA_004217565.1, GCA_007559285.1, GCA_013358205.1, GCA_023650915.1, GCA_020966695.1 GCA_003101015.1, GCA_000202835.1, GCA_900188005.1, GCA_900167375.1, GCA_020532645.1 GCA_001676875.1, GCA_002443295.1, GCA_000829055.1, GCA_024205965.1, GCA_000816845.1 GCA_000980985.1, GCA_001562415.1, GCA_002204835.1, GCA_001373515.1, GCA_002095475.1 GCA_001305675.1, GCA_001889125.1, GCA_023618015.1, GCA_016127455.1, GCA_014641615.1 GCA_003813965.1, GCA_014197255.1, GCA_015265435.1, GCA_900188295.1, GCA_900127505.1 GCA_002998435.1, GCA_003021645.1, GCA_018603515.1, GCA_900113405.1, GCA_900129095.1 GCA_004564235.1, GCA_012844455.2, GCA_001936115.1, GCA_002259705.1, GCA_002222655.1 GCA_016894205.1, GCA_014642675.1, GCA_021568805.1, GCA_013133795.1, GCA_003815775.1 GCA_017347605.1, GCA_009932435.1, GCA_003634935.1, GCA_000821065.2, GCA_010500835.1 GCA_009827325.1, GCA_003971195.1, GCA_001678755.1, GCA_013697085.1, GCA_900129685.1 GCA_003355455.1, GCA_002162375.1, GCA_018704065.1, GCA_010915705.1, GCA_004801655.1 GCA_019977655.1, GCA_027853965.1, GCA_000333035.1, GCA_000021725.1, GCA_023349185.1 GCA_005406225.1, GCA_900258455.1, GCA_002915195.1, GCA_013377855.1, GCA_900114005.1 GCA_900188235.1, GCA_001956695.1, GCA_900112255.1, GCA_900112925.1, GCA_007559425.1 GCA_003628755.1, GCA_008710035.1, GCA_011758565.1, GCA_900496975.1, GCA_003955665.1 GCA_000009365.1, GCA_016909125.1, GCA_003664125.1, GCA_008807855.1, GCA_015070855.1 GCA_007990995.1, GCA_013393325.1, GCA_900141925.1, GCA_004331485.1, GCA_020532705.1 GCA_000750955.1, GCA_014195635.1, GCA_002285515.1, GCA_002117405.1, GCA_014199625.1 GCA_000471025.2, GCA_002286965.1, GCA_904830935.1, GCA_027570035.1, GCA_009014635.1 GCA_003516165.1, GCA_003963555.1, GCA_018409485.1, GCA_008364625.1, GCA_011762125.1 GCA_028657945.1, GCA_003966625.1, GCA_027921925.1, GCA_022637435.1, GCA_024160875.1 GCA_009377185.1, GCA_000219805.1, GCA_000724605.1, GCA_014190875.1, GCA_001007995.1 GCA_000007505.1, GCA_900110225.1, GCA_016653415.1, GCA_007752935.1, GCA_003862465.1 GCA_021556455.1, GCA_018919215.1, GCA_003201815.1, GCA_002964965.1, GCA_012037625.1 GCA_008806995.1, GCA_017349075.1, GCA_010731735.1, GCA_006239215.1, GCA_016865425.1 GCA_013087605.1, GCA_900142085.1, GCA_011044155.1, GCA_000332975.1, GCA_021654335.1 GCA_001544015.1, GCA_020917325.1, GCA_004362215.1, GCA_009757775.1, GCA_001186365.1 GCA_006385805.1, GCA_022179305.1, GCA_023223525.1, GCA_003590875.1, GCA_023555375.1 GCA_027922305.1, GCA_004357985.1, GCA_000218625.1, GCA_001941345.1, GCA_000224005.3 GCA_016921115.1, GCA_003602095.1, GCA_900114895.1, GCA_016804345.1, GCA_003696215.1 GCA_028570795.1, GCA_000022745.1, GCA_007572485.1, GCA_900116935.1, GCA_002554705.1 GCA_000550785.1, GCA_004135975.1, GCA_902713415.1, GCA_006716135.1, GCA_008710705.1 GCA_900187375.1, GCA_001112925.1, GCA_018398275.1, GCA_009708075.1, GCA_003581615.1 GCA_004336975.1, GCA_902459725.1, GCA_008085905.1, GCA_006539825.1, GCA_001687585.2 GCA_008705135.1, GCA_900101845.1, GCA_010723675.1, GCA_000768555.3, GCA_013267695.1 GCA_002117445.1, GCA_009727155.1, GCA_001083805.1, GCA_000513095.1, GCA_900112605.1 GCA_002209445.1, GCA_008065115.1, GCA_014230465.1, GCA_029383185.1, GCA_900188225.1 GCA_000968055.1, GCA_001434515.1, GCA_003339525.1, GCA_003403135.1, GCA_000026745.1 GCA_002813775.1, GCA_015477355.1, GCA_001544835.2, GCA_900111315.1, GCA_000024865.1 GCA_022533465.1, GCA_900100375.1, GCA_020166415.1, GCA_015352425.1, GCA_011516735.1 GCA_021049245.1, GCA_002278015.2, GCA_022637495.1, GCA_013282625.1, GCA_023169965.1 GCA_900475975.1, GCA_004364635.1, GCA_003590775.1, GCA_028737255.1, GCA_002868735.1 GCA_900638055.1, GCA_016613475.2, GCA_019343455.1, GCA_013327855.1, GCA_003952725.1 GCA_013409065.1, GCA_900104045.1, GCA_017901135.1, GCA_008370835.2, GCA_002897135.1 GCA_900114325.1, GCA_007988965.1, GCA_003312915.1, GCA_003967515.1, GCA_016890085.1 GCA_006716485.1, GCA_900176595.1, GCA_003258945.1, GCA_021654115.1, GCA_014358015.1 GCA_018986915.1, GCA_020162295.1, GCA_900115665.1, GCA_002302415.1, GCA_002335465.1 GCA_009856525.1, GCA_011174675.1, GCA_013423785.1, GCA_013179575.1, GCA_019753765.1 GCA_014199435.1, GCA_014698505.1, GCA_018343535.1, GCA_003054195.1, GCA_007991195.1 GCA_900167115.1, GCA_000525995.1, GCA_900129265.1, GCA_021228235.1, GCA_000166335.1 GCA_024748065.1, GCA_010500865.1, GCA_019083965.1, GCA_013761005.1, GCA_026241935.1 GCA_004564275.1, GCA_900108055.1, GCA_020872015.1, GCA_008974325.1, GCA_006538745.1 GCA_003254585.1, GCA_001995255.1, GCA_014236795.1, GCA_004006375.1, GCA_014196625.1 GCA_001623895.1, GCA_000164695.2, GCA_900188125.1, GCA_003019315.1, GCA_017565745.1 GCA_009192775.1, GCA_014638095.1, GCA_003330785.1, GCA_007754155.1, GCA_005786655.1 GCA_000195635.1, GCA_017652985.1, GCA_001641005.1, GCA_000367205.1, GCA_020783375.1 GCA_000601485.1, GCA_003202285.1, GCA_001886435.1, GCA_003515075.1, GCA_013415105.1 GCA_002943565.1, GCA_007280415.1, GCA_013378015.1, GCA_004365815.1, GCA_002356315.1 GCA_017876535.1, GCA_900115755.1, GCA_003710255.1, GCA_014873875.1, GCA_900105155.1 GCA_003688415.1, GCA_008728085.1, GCA_003387575.1, GCA_000016165.1, GCA_009925085.1 GCA_014656585.1, GCA_000025185.1, GCA_900130065.1, GCA_003254175.1, GCA_013370205.1 GCA_001373395.1, GCA_007830735.1, GCA_900092025.1, GCA_025234795.1, GCA_002846575.1 GCA_009184905.1, GCA_001458375.1, GCA_016863315.1, GCA_011758765.1, GCA_003675895.1 GCA_003863365.1, GCA_019890715.1, GCA_002736145.1, GCA_016862375.1, GCA_000266945.1 GCA_018739865.1, GCA_003202155.1, GCA_900096925.1, GCA_016909495.1, GCA_016649425.1 GCA_002006345.1, GCA_002192535.1, GCA_900096885.1, GCA_021398715.1, GCA_001750105.1 GCA_002917885.1, GCA_001274785.1, GCA_004123295.1, GCA_007829875.1, GCA_014109725.1 GCA_012184385.1, GCA_016027095.1, GCA_000009045.1, GCA_008710095.1, GCA_000174015.1 GCA_018128205.1, GCA_008039575.1, GCA_000021325.1, GCA_004135285.1, GCA_000770675.1 GCA_014287675.1, GCA_900113025.1, GCA_001483145.1, GCA_002021945.1, GCA_021462285.1 GCA_900230245.1, GCA_001751255.1, GCA_001050515.1, GCA_026337845.1, GCA_028330815.1 GCA_024622975.1, GCA_028532485.1, GCA_002216775.1, GCA_000158095.2, GCA_014645355.1 GCA_900094565.1, GCA_003730115.1, GCA_016028775.1, GCA_003346515.1, GCA_001642085.1 GCA_000218895.1, GCA_013294115.1, GCA_018271975.1, GCA_900104825.1, GCA_014595995.2 GCA_014647355.1, GCA_006539885.1, GCA_004358765.1, GCA_005885675.1, GCA_001653755.1 GCA_009301415.1, GCA_001645705.1, GCA_002849895.1, GCA_900111925.1, GCA_024721115.1 GCA_000772105.1, GCA_001641085.1, GCA_014137855.1, GCA_013488025.1, GCA_900141875.1 GCA_000521805.1, GCA_004362715.1, GCA_002995785.1, GCA_002076895.1, GCA_013408985.1 GCA_900141855.1, GCA_000007765.2, GCA_900111295.1, GCA_002813755.1, GCA_010723305.1 GCA_003150675.1, GCA_006493105.1, GCA_002564005.1, GCA_008275125.1, GCA_002563955.1 GCA_016584085.1, GCA_025985205.1, GCA_000970795.1, GCA_025706525.1, GCA_019669985.1 GCA_003050755.1, GCA_001730285.1, GCA_900143005.1, GCA_008121405.1, GCA_007991375.1 GCA_014337255.1, GCA_003612165.1, GCA_016862315.1, GCA_016595415.1, GCA_900187005.1 GCA_001939045.1, GCA_007990525.1, GCA_022227625.1, GCA_007751715.1, GCA_002871975.1 GCA_009299505.1, GCA_003970735.1, GCA_017724035.1, GCA_001721185.1, GCA_901482605.1 GCA_017813245.1, GCA_002014855.1, GCA_018436245.1, GCA_004078655.1, GCA_008579125.1 GCA_011045835.1, GCA_000757725.1, GCA_014650655.1, GCA_011761475.1, GCA_013371475.1 GCA_001742225.1, GCA_028868935.1, GCA_900177775.1, GCA_017309675.2, GCA_900129225.1 GCA_000743945.1, GCA_007990205.1, GCA_000018025.1, GCA_002208825.2, GCA_001750285.1 GCA_014845115.1, GCA_001283065.1, GCA_018380615.1, GCA_014640815.1, GCA_000023785.1 GCA_000227705.3, GCA_023195815.2, GCA_018128425.1, GCA_003176875.1, GCA_011044175.1 GCA_000756715.2, GCA_000525875.1, GCA_002995795.1, GCA_900143645.1, GCA_002874775.1 GCA_007992455.1, GCA_017313335.1, GCA_001317355.1, GCA_015529805.1, GCA_017487345.1 GCA_900114155.1, GCA_005144565.1, GCA_003368325.1, GCA_006542335.1, GCA_002286975.1 GCA_900095885.1, GCA_018390635.1, GCA_009831105.1, GCA_003063475.1, GCA_016595215.1 GCA_024206795.1, GCA_023078335.1, GCA_010722995.1, GCA_002554795.1, GCA_000210915.2 GCA_001437205.1, GCA_900091575.1, GCA_900111675.1, GCA_013409125.2, GCA_013761015.1 GCA_014203235.1, GCA_010500815.1, GCA_011392125.1, GCA_011090385.1, GCA_014672835.1 GCA_014384995.1, GCA_900221005.1, GCA_014397005.1, GCA_900566075.1, GCA_900102315.1 GCA_002763775.1, GCA_010975035.1, GCA_900104475.1, GCA_013347125.1, GCA_014836935.1 GCA_001029435.1, GCA_900478115.1, GCA_001434455.1, GCA_014174355.1, GCA_013201605.1 GCA_013421525.1, GCA_003932295.1, GCA_900163755.1, GCA_002568625.1, GCA_900096965.1 GCA_900168065.1, GCA_016595505.1, GCA_900176415.1, GCA_014164785.1, GCA_000165715.3 GCA_014204835.1, GCA_900095155.1, GCA_016907535.1, GCA_003966875.1, GCA_003330825.1 GCA_900142145.1, GCA_021010575.1, GCA_009834985.1, GCA_900102425.1, GCA_001440545.1 GCA_004365635.1, GCA_003002115.1, GCA_902859705.1, GCA_014863355.1, GCA_001028625.1 GCA_900453395.1, GCA_902459705.1, GCA_000187125.1, GCA_001435195.1, GCA_028421465.1 GCA_017255295.1, GCA_004364325.1, GCA_905250085.1, GCA_900105725.1, GCA_024463555.1 GCA_900111245.1, GCA_005864065.1, GCA_007990975.1, GCA_003470435.1, GCA_004834005.1 GCA_021183725.1, GCA_003610465.1, GCA_002943425.1, GCA_000023405.1, GCA_028553725.1 GCA_019492185.1, GCA_002442935.1, GCA_003313465.1, GCA_017358165.1, GCA_001457055.1 GCA_000025885.1, GCA_004295645.1, GCA_015694465.1, GCA_005877885.1, GCA_009362735.1 GCA_014196925.1, GCA_900215145.1, GCA_016901055.1, GCA_003176895.1, GCA_003575225.1 GCA_018362775.1, GCA_005217835.1, GCA_000224925.2, GCA_015209745.1, GCA_008329985.2 GCA_001913155.1, GCA_002234915.1, GCA_900168195.1, GCA_900167065.1, GCA_016908295.1 GCA_014250475.1, GCA_004115975.1, GCA_003987185.1, GCA_000253115.1, GCA_000067165.1 GCA_003591235.1, GCA_900143095.1, GCA_004340875.1, GCA_005233845.1, GCA_003987215.1 GCA_024362265.1, GCA_902859775.1, GCA_005670695.1, GCA_900107845.1, GCA_016862735.1 GCA_013394205.1, GCA_028009925.1, GCA_013085345.1, GCA_900141845.1, GCA_016027455.1 GCA_000177375.1, GCA_000756505.1, GCA_021532375.1, GCA_003076455.1, GCA_002160635.1 GCA_006716265.1, GCA_024648825.1, GCA_013394005.1, GCA_005924265.1, GCA_007971225.1 GCA_902498995.1, GCA_000224085.1, GCA_003403295.1, GCA_001768715.1, GCA_002234615.1 GCA_000772535.1, GCA_900129665.1, GCA_002310475.1, GCA_001995215.1, GCA_016411825.1 GCA_013377905.1, GCA_014282275.1, GCA_003610015.1, GCA_004295345.1, GCA_014305095.1 GCA_014639655.1, GCA_016862235.1, GCA_022760175.1, GCA_000013325.1, GCA_003633725.1 GCA_013340305.1, GCA_900116235.1, GCA_015234765.1, GCA_002843235.3, GCA_003933745.1 GCA_007746075.1, GCA_900156285.1, GCA_001541345.2, GCA_004342685.1, GCA_011046535.1 GCA_001584605.1, GCA_007559235.1, GCA_003387475.1, GCA_014204985.1, GCA_016918035.1 GCA_900112595.1, GCA_009931695.1, GCA_000013645.1, GCA_015182315.1, GCA_010669285.1 GCA_003574345.1, GCA_009720735.1, GCA_002879885.1, GCA_005402645.1, GCA_000406945.1 GCA_028553785.1, GCA_002998535.1, GCA_002746455.1, GCA_004684205.1, GCA_014836555.1 GCA_007830035.1, GCA_000732925.1, GCA_002796605.1, GCA_015265455.1, GCA_014635345.1 GCA_003012735.1, GCA_003594935.2, GCA_000828915.1, GCA_000156995.2, GCA_008704795.1 GCA_900155985.1, GCA_006716425.1, GCA_019947135.1, GCA_028550555.1, GCA_003977615.1 GCA_900143515.1, GCA_009648575.1, GCA_003987395.1, GCA_014201715.1, GCA_000735695.2 GCA_025567005.1, GCA_009498695.1, GCA_014269225.2, GCA_003609645.1, GCA_007859215.1 GCA_007992535.1, GCA_000400635.2, GCA_014196805.1, GCA_022267535.1, GCA_003003285.1 GCA_014174155.1, GCA_008244685.1, GCA_003071405.1, GCA_900107015.1, GCA_014205885.1 GCA_025822245.1, GCA_001955695.1, GCA_900167025.1, GCA_007829955.1, GCA_015453285.1 GCA_014639255.1, GCA_002811955.1, GCA_900103835.1, GCA_000143145.1, GCA_020687725.1 GCA_900451195.1, GCA_014651695.1, GCA_013408415.1, GCA_023169945.1, GCA_001187785.1 GCA_019456655.1, GCA_000224335.2, GCA_001941485.1, GCA_000986785.1, GCA_002798305.1 GCA_002794255.1, GCA_010365285.1, GCA_024072275.1, GCA_006538985.1, GCA_017378515.1 GCA_002934005.1, GCA_000186245.1, GCA_010669125.1, GCA_900445995.1, GCA_003611475.1 GCA_023336985.1, GCA_021044935.1, GCA_003363435.1, GCA_900107965.1, GCA_012676905.1 GCA_009828925.2, GCA_008704205.1, GCA_900108595.1, GCA_014640435.1, GCA_004402515.1 GCA_012927085.1, GCA_000967545.1, GCA_010500615.1, GCA_024055575.1, GCA_024622505.1 GCA_011189575.1, GCA_003589745.1, GCA_910593825.1, GCA_000785105.2, GCA_003955755.1 GCA_002174125.1, GCA_014653215.1, GCA_018275325.1, GCA_022221585.1, GCA_900129125.1 GCA_000262445.1, GCA_014635085.1, GCA_000948985.2, GCA_000768695.1, GCA_900107115.1 GCA_001652275.1, GCA_000024505.1, GCA_008704495.1, GCA_012911005.2, GCA_000313915.1 GCA_018333255.1, GCA_004341825.1, GCA_900112085.1, GCA_007994985.1, GCA_017821535.1 GCA_001262015.1, GCA_004365655.1, GCA_014652355.1, GCA_000466985.1, GCA_000014725.1 GCA_018024495.1, GCA_900215355.1, GCA_026344055.1, GCA_004571135.1, GCA_900109165.1 GCA_900142655.1, GCA_022179225.1, GCA_002220285.1, GCA_013520865.1, GCA_000802385.1 GCA_007786455.1, GCA_900109575.1, GCA_000153345.1, GCA_001439985.1, GCA_000183745.1 GCA_009807005.1, GCA_000190595.1, GCA_002196515.1, GCA_018598585.1, GCA_019679475.1 GCA_016595675.1, GCA_029076465.1, GCA_005144585.1, GCA_009755605.1, GCA_004803815.1 GCA_009363855.1, GCA_001658025.2, GCA_006716445.1, GCA_000334455.1, GCA_003546685.2 GCA_000751215.2, GCA_003671975.1, GCA_900101865.1, GCA_000317695.1, GCA_014174255.1 GCA_000832905.1, GCA_018467115.1, GCA_002211445.1, GCA_009695765.1, GCA_014635025.1 GCA_011059145.1, GCA_012769535.1, GCA_900103935.1, GCA_900110835.1, GCA_027922205.1 GCA_015708085.1, GCA_000812965.1, GCA_004343035.1, GCA_026240095.1, GCA_000299895.1 GCA_000022085.1, GCA_001955735.1, GCA_022267325.1, GCA_008035925.1, GCA_016127995.1 GCA_900109225.1, GCA_003312875.1, GCA_013177695.1, GCA_014205155.1, GCA_003355155.1 GCA_014284155.1, GCA_017377715.1, GCA_000188015.3, GCA_001514205.1, GCA_003858655.1 GCA_016904155.1, GCA_003350475.1, GCA_003674095.1, GCA_028609865.1, GCA_003966955.1 GCA_000157355.2, GCA_022569295.1, GCA_006152115.1, GCA_008727755.1, GCA_001889445.1 GCA_005765165.1, GCA_003966225.1, GCA_000021045.1, GCA_002949635.1, GCA_000473245.1 GCA_002860745.1, GCA_013358405.1, GCA_000973625.1, GCA_000714815.1, GCA_014898655.1 GCA_002952055.1, GCA_004769665.1, GCA_900111545.1, GCA_009796285.1, GCA_014196195.1 GCA_001941445.1, GCA_000246855.1, GCA_000152145.1, GCA_021404405.1, GCA_002197665.1 GCA_003226345.1, GCA_013364315.1, GCA_023556335.1, GCA_901764975.1, GCA_014649915.1 GCA_008369015.1, GCA_002844395.1, GCA_014230085.1, GCA_023701485.1, GCA_002220865.2 GCA_900142665.1, GCA_018919205.1, GCA_000243695.3, GCA_009827575.1, GCA_022760805.1 GCA_015679185.1, GCA_023061175.1, GCA_900100555.1, GCA_021378375.1, GCA_014773225.1 GCA_025821665.1, GCA_000498475.1, GCA_007991515.1, GCA_014199255.1, GCA_019397265.1 GCA_003172915.1, GCA_014651135.1, GCA_003116815.1, GCA_900172215.1, GCA_010499265.1 GCA_003024515.2, GCA_016862615.1, GCA_008843165.1, GCA_027983475.1, GCA_900110105.1 GCA_002117105.1, GCA_900167155.1, GCA_016865235.1, GCA_016908785.1, GCA_900103665.1 GCA_021166475.1, GCA_000525855.1, GCA_008107635.1, GCA_022819245.1, GCA_014305215.1 GCA_900156665.1, GCA_009931115.1, GCA_003660425.1, GCA_024172085.1, GCA_000019785.1 GCA_018861865.1, GCA_000723225.2, GCA_900109885.1, GCA_002902845.1, GCA_022538055.1 GCA_001048695.1, GCA_002554425.1, GCA_026015925.1, GCA_001458695.1, GCA_027158565.1 GCA_000260115.1, GCA_001027285.1, GCA_900106975.1, GCA_014541205.1, GCA_000661915.1 GCA_003336675.1, GCA_001917445.1, GCA_002102065.1, GCA_000829715.2, GCA_017498075.1 GCA_002811505.1, GCA_016900655.1, GCA_017357285.1, GCA_003852045.1, GCA_900188485.1 GCA_010727945.1, GCA_013179555.1, GCA_013003965.1, GCA_004364315.1, GCA_003864255.1 GCA_002101775.1, GCA_900215515.1, GCA_004339575.1, GCA_016863115.1, GCA_013181415.1 GCA_900115625.1, GCA_017792025.1, GCA_900454495.1, GCA_012524165.2, GCA_014837035.1 GCA_900102725.1, GCA_019039035.1, GCA_900105955.1, GCA_001514305.1, GCA_014384905.1 GCA_900112455.1, GCA_009696165.1, GCA_008806755.1, GCA_900105565.1, GCA_003628445.1 GCA_007992115.1, GCA_004008295.1, GCA_029028125.1, GCA_002086915.1, GCA_900129565.1 GCA_018332655.1, GCA_900115255.1, GCA_000967465.2, GCA_900113905.1, GCA_002355735.1 GCA_900108475.1, GCA_020342335.1, GCA_000722875.1, GCA_021300655.1, GCA_002899895.2 GCA_022870905.1, GCA_002901445.1, GCA_014489415.1, GCA_010730745.1, GCA_016757795.1 GCA_001440395.1, GCA_001434895.1, GCA_000144405.1, GCA_014205175.1, GCA_001465795.2 GCA_004124315.2, GCA_014650515.1, GCA_020215645.1, GCA_000349325.1, GCA_003253705.1 GCA_003660105.1, GCA_023656545.1, GCA_001440385.1, GCA_004118265.1, GCA_000006945.2 GCA_009720525.1, GCA_900637845.1, GCA_022179345.1, GCA_014645855.1, GCA_014698965.1 GCA_000020525.1, GCA_004342445.1, GCA_001005905.1, GCA_000307165.1, GCA_900101085.1 GCA_900115145.1, GCA_900129135.1, GCA_006439335.1, GCA_900452755.1, GCA_011683955.1 GCA_002266855.1, GCA_000243135.3, GCA_002895605.1, GCA_023887685.1, GCA_020412445.1 GCA_025725725.1, GCA_000146185.1, GCA_013170725.1, GCA_003071345.1, GCA_009792355.1 GCA_001584145.1, GCA_013036045.1, GCA_008831485.1, GCA_014207495.1, GCA_018324685.1 GCA_001752425.1, GCA_900115605.1, GCA_002266435.2, GCA_900491935.1, GCA_013328205.1 GCA_002844375.1, GCA_001277235.1, GCA_900106695.1, GCA_007860175.1, GCA_003634045.1 GCA_004353915.1, GCA_014192215.1, GCA_000512205.2, GCA_000318195.2, GCA_007747015.1 GCA_021172165.1, GCA_016908375.1, GCA_003182485.1, GCA_011397855.1, GCA_009708035.1 GCA_025384885.1, GCA_004328075.1, GCA_001049755.1, GCA_004028015.1, GCA_018292165.1 GCA_015160875.1, GCA_900114635.1, GCA_004340465.1, GCA_014193895.1, GCA_014467235.1 GCA_003934165.1, GCA_900182665.1, GCA_003444685.1, GCA_014196495.1, GCA_016862115.1 GCA_024764775.1, GCA_000388115.1, GCA_002207765.1, GCA_900445305.1, GCA_009648975.1 GCA_003050705.1, GCA_010499255.1, GCA_014873855.1, GCA_000262545.1, GCA_016584055.1 GCA_002879935.1, GCA_013408715.1, GCA_004916895.1, GCA_004682045.1, GCA_014653175.1 GCA_900091525.1, GCA_001945605.1, GCA_001619725.1, GCA_000005845.2, GCA_900198195.1 GCA_009903685.1, GCA_017302975.1, GCA_016918565.1, GCA_001990485.1, GCA_017353455.1 GCA_011405655.1, GCA_023612255.1, GCA_003254155.1, GCA_004346185.1, GCA_015910445.1 GCA_024979255.1, GCA_004363045.1, GCA_014055025.1, GCA_007971025.1, GCA_014692875.1 GCA_003351345.1, GCA_014203395.1, GCA_008693605.1, GCA_003668555.1, GCA_022014595.1 GCA_001277255.1, GCA_003182415.1, GCA_014384805.1, GCA_001878675.1, GCA_900638615.1 GCA_003612795.1, GCA_003938205.1, GCA_018221465.1, GCA_013265555.1, GCA_014638745.1 GCA_900452545.1, GCA_019226825.1, GCA_000298255.1, GCA_001768695.1, GCA_000331025.1 GCA_006385685.1, GCA_019857205.1, GCA_013387465.1, GCA_019711535.1, GCA_001027185.1 GCA_004346225.1, GCA_003364175.1, GCA_000245735.2, GCA_004785625.2, GCA_902459695.1 GCA_022135835.1, GCA_900107165.1, GCA_015069875.1, GCA_900182595.1, GCA_018597345.1 GCA_002257705.1, GCA_018866305.1, GCA_009882915.1, GCA_000205025.1, GCA_025567095.1 GCA_003201855.1, GCA_003314435.2, GCA_002368115.1, GCA_021276285.1, GCA_000715205.1 GCA_000612505.1, GCA_017301775.1, GCA_900177815.1, GCA_001468135.1, GCA_003584015.1 GCA_900113665.1, GCA_014653115.1, GCA_007004725.1, GCA_000521785.1, GCA_014199215.1 GCA_018333335.1, GCA_900094935.1, GCA_000525635.1, GCA_003002875.1, GCA_003595175.1 GCA_001971585.1, GCA_001021085.1, GCA_900102855.1, GCA_000158475.2, GCA_900108955.1 GCA_019145435.1, GCA_016862075.1, GCA_001050675.1, GCA_014204715.1, GCA_014268815.2 GCA_001652585.1, GCA_003515705.1, GCA_014836665.1, GCA_002865995.1, GCA_018256865.1 GCA_014195585.1, GCA_017426725.1, GCA_003008595.1, GCA_000298195.1, GCA_021538615.1 GCA_000143725.1, GCA_001662775.1, GCA_900089595.1, GCA_021028635.1, GCA_002902965.1 GCA_900096955.1, GCA_014322885.1, GCA_014640155.1, GCA_001693415.1, GCA_011089895.1 GCA_014490555.1, GCA_001026695.1, GCA_009646095.1, GCA_003751625.1, GCA_021300615.1 GCA_900142985.1, GCA_900102745.1, GCA_000970465.2, GCA_014650055.1, GCA_023156235.1 GCA_014202975.1, GCA_003003235.1, GCA_900099815.1, GCA_022557255.1, GCA_000934605.2 GCA_900149755.1, GCA_026935685.1, GCA_008580665.1, GCA_002866825.1, GCA_003851555.1 GCA_900454705.1, GCA_003959475.1, GCA_902810445.1, GCA_000214825.1, GCA_000771745.2 GCA_000732535.1, GCA_000577895.1, GCA_020805785.1, GCA_900111615.1, GCA_009025875.1 GCA_900100005.1, GCA_018736085.1, GCA_000973105.1, GCA_000214275.3, GCA_012563545.1 GCA_002953735.1, GCA_022601675.1, GCA_900103975.1, GCA_013366375.1, GCA_900142725.1 GCA_001758195.1, GCA_014243395.1, GCA_018798885.1, GCA_019263705.2, GCA_024753255.1 GCA_900475035.1, GCA_000493735.1, GCA_022179585.1, GCA_000813825.1, GCA_000218855.1 GCA_014656355.1, GCA_004362515.1, GCA_003340385.1, GCA_000439775.1, GCA_020532725.1 GCA_019614655.1, GCA_001043025.1, GCA_013019105.1, GCA_003024195.1, GCA_001945525.1 GCA_900108215.1, GCA_016612505.1, GCA_000186265.1, GCA_000469325.1, GCA_004015185.1 GCA_014191775.1, GCA_016595435.1, GCA_013359935.1, GCA_013283835.1, GCA_018881835.1 GCA_000344805.1, GCA_014837235.1, GCA_012184415.1, GCA_023149135.1, GCA_000439255.1 GCA_900218025.1, GCA_902729325.1, GCA_014635725.1, GCA_003285015.1, GCA_006094375.1 GCA_007993815.1, GCA_003337355.1, GCA_014199325.1, GCA_002736205.1, GCA_008824185.1 GCA_014692525.1, GCA_013349145.1, GCA_001590685.1, GCA_000018145.1, GCA_007164725.1 GCA_013030075.1, GCA_002288065.1, GCA_004770635.1, GCA_000203895.1, GCA_002441695.1 GCA_002216005.1, GCA_001306115.1, GCA_900230195.1, GCA_900129755.1, GCA_001936615.1 GCA_001017435.1, GCA_011742165.1, GCA_014641195.1, GCA_011766325.1, GCA_017742995.1 GCA_902859635.1, GCA_004349055.1, GCA_020991375.1, GCA_017592555.1, GCA_003004765.2 GCA_900538275.1, GCA_019957155.1, GCA_020171805.1, GCA_019891355.1, GCA_023516595.1 GCA_021246445.1, GCA_003788635.1, GCA_004022585.1, GCA_008930655.1, GCA_008831085.1 GCA_900108455.1, GCA_007741475.1, GCA_016901035.1, GCA_015277775.1, GCA_001436755.1 GCA_021532625.1, GCA_023555415.1, GCA_018736065.1, GCA_900100355.1, GCA_003815995.1 GCA_900143245.1, GCA_000699505.1, GCA_014698035.1, GCA_000598065.1, GCA_003130405.1 GCA_000236035.1, GCA_000469155.1, GCA_012927205.1, GCA_902459745.1, GCA_900111865.1 GCA_016591975.1, GCA_002841295.1, GCA_002797815.1, GCA_002412335.2, GCA_004341245.1 GCA_004361795.1, GCA_018968705.1, GCA_018622995.1, GCA_023736155.1, GCA_000153485.2 GCA_000011305.1, GCA_006740765.1, GCA_000803645.1, GCA_001597285.1, GCA_003788965.1 GCA_900114725.1, GCA_003382275.1, GCA_011762155.1, GCA_001586155.1, GCA_003024675.1 GCA_000023745.1, GCA_003130585.1, GCA_900113525.1, GCA_001653335.1, GCA_016863015.1 GCA_900683625.1, GCA_002812985.1, GCA_014654935.1, GCA_900103785.1, GCA_000013425.1 GCA_000465575.1, GCA_028401765.1, GCA_009258225.1, GCA_003534205.1, GCA_000014005.1 GCA_001435395.1, GCA_014643915.1, GCA_014635845.1, GCA_004364935.1, GCA_002895525.1 GCA_024722315.1, GCA_000513115.1, GCA_014637345.1, GCA_024055795.1, GCA_015319165.1 GCA_900637895.1, GCA_011881425.1, GCA_000953635.1, GCA_003182095.1, GCA_003977605.1 GCA_002028325.1, GCA_005233835.1, GCA_900105355.1, GCA_024343775.1, GCA_000015585.1 GCA_000307105.1, GCA_003253745.1, GCA_002120225.1, GCA_011764565.1, GCA_009930795.1 GCA_900323885.1, GCA_000785495.1, GCA_001730315.1, GCA_900107765.1, GCA_007827045.1 GCA_017052585.1, GCA_014652675.1, GCA_001077815.2, GCA_000330725.2, GCA_002276885.1 GCA_002288145.1, GCA_000739435.1, GCA_017068455.1, GCA_014648335.1, GCA_900129745.1 GCA_000007025.1, GCA_000023225.1, GCA_001442805.1, GCA_006715045.1, GCA_000014185.1 GCA_007992715.1, GCA_026344035.1, GCA_028561335.1, GCA_009429125.1, GCA_001695575.1 GCA_003096635.1, GCA_006716695.1, GCA_001890925.1, GCA_024499545.1, GCA_018500185.1 GCA_000284635.1, GCA_000212735.1, GCA_009909155.2, GCA_001484725.1, GCA_001039475.1 GCA_001663855.1, GCA_900220995.1, GCA_027498475.1, GCA_000297415.1, GCA_001687365.1 GCA_001654925.1, GCA_900111575.1, GCA_014641595.1, GCA_006569205.1, GCA_020099395.1 GCA_003054055.1, GCA_023156385.1, GCA_010727325.1, GCA_002563925.1, GCA_007995095.1 GCA_001938985.1, GCA_027474505.1, GCA_900218035.1, GCA_005402885.1, GCA_000260985.3 GCA_003076635.1, GCA_004150225.1, GCA_000829465.1, GCA_003351045.1, GCA_900114765.1 GCA_005484965.1, GCA_003173035.1, GCA_014640295.1, GCA_015351335.1, GCA_014489845.1 GCA_009826895.1, GCA_014489495.1, GCA_018128545.1, GCA_003336425.1, GCA_000092225.1 GCA_900105825.1, GCA_021065005.1, GCA_900129455.1, GCA_008326305.1, GCA_007827555.1 GCA_002936985.1, GCA_013127755.1, GCA_020511155.1, GCA_002257665.1, GCA_015277515.1 GCA_000723465.1, GCA_016909175.1, GCA_001904615.1, GCA_025660475.1, GCA_012427845.1 GCA_009176665.1, GCA_004354365.1, GCA_002094855.1, GCA_002119765.1, GCA_004354085.1 GCA_014195435.1, GCA_900187045.1, GCA_014041935.1, GCA_018139565.1, GCA_007997225.1 GCA_006716235.1, GCA_000221985.1, GCA_005862235.1, GCA_000238255.4, GCA_002982135.1 GCA_020735445.1, GCA_007991275.1, GCA_019049655.1, GCA_020687805.1, GCA_016743795.2 GCA_007830065.1, GCA_014396385.1, GCA_016908195.1, GCA_008329945.1, GCA_002073495.2 GCA_024662015.1, GCA_007859715.1, GCA_003688855.1, GCA_900141735.1, GCA_000154825.1 GCA_014650175.1, GCA_004328555.1, GCA_000389675.2, GCA_000186225.1, GCA_003933235.1 GCA_002291425.1, GCA_014207795.1, GCA_004571195.1, GCA_002224365.1, GCA_003651245.1 GCA_019203965.1, GCA_006538185.1, GCA_019793465.1, GCA_011604685.1, GCA_001280945.1 GCA_011066545.1, GCA_007859765.1, GCA_900101165.1, GCA_016907955.1, GCA_016907545.1 GCA_001685395.1, GCA_009789235.1, GCA_003634705.1, GCA_014652935.1, GCA_008801925.2 GCA_019931005.1, GCA_004328965.1, GCA_013314975.1, GCA_003864415.1, GCA_004322855.1 GCA_014284125.1, GCA_016599875.1, GCA_015160855.1, GCA_900114975.1, GCA_009498035.1 GCA_012927265.1, GCA_018881755.1, GCA_000750005.1, GCA_900091465.1, GCA_001751365.1 GCA_000024545.1, GCA_014643775.1, GCA_003053725.1, GCA_022179385.1, GCA_900105925.1 GCA_025946765.1, GCA_003391255.1, GCA_900110615.1, GCA_004364555.1, GCA_000484535.1 GCA_003208215.1, GCA_003070885.1, GCA_905220835.1, GCA_900188425.1, GCA_910593845.1 GCA_014206945.1, GCA_900114425.1, GCA_002939475.1, GCA_004348985.1, GCA_003337415.1 GCA_000255295.1, GCA_900638445.1, GCA_002214395.1, GCA_900100315.1, GCA_000282115.1 GCA_010671605.1, GCA_000091465.1, GCA_016592595.1, GCA_014203835.1, GCA_015482775.1 GCA_002260845.1, GCA_023161965.1, GCA_900113055.1, GCA_000270245.1, GCA_019511665.1 GCA_004103615.1, GCA_022538115.1, GCA_003568585.1, GCA_000014785.1, GCA_900176005.1 GCA_003217325.1, GCA_014202225.1, GCA_008124865.1, GCA_001431485.1, GCA_004216755.1 GCA_024703995.1, GCA_022354425.1, GCA_020542785.1, GCA_001029105.3, GCA_014196595.1 GCA_021474405.1, GCA_003173015.1, GCA_000222305.1, GCA_025660415.1, GCA_003443995.1 GCA_001832885.1, GCA_000612865.1, GCA_014287435.1, GCA_002257545.1, GCA_014397245.1 GCA_000009805.1, GCA_015751785.1, GCA_002075795.1, GCA_009939225.1, GCA_019336435.1 GCA_004354915.1, GCA_013388375.1, GCA_012910885.1, GCA_025144365.1, GCA_003076135.1 GCA_021568695.1, GCA_020092905.1, GCA_001455205.1, GCA_004792515.1, GCA_903994055.1 GCA_012396155.1, GCA_005780245.1, GCA_000517305.1, GCA_001703515.1, GCA_025742995.1 GCA_021033825.1, GCA_019711515.1, GCA_016656965.1, GCA_004358025.1, GCA_018363075.1 GCA_000427335.1, GCA_001855575.1, GCA_018409545.1, GCA_003970385.1, GCA_013328345.1 GCA_003345355.1, GCA_004745595.1, GCA_001021025.1, GCA_001951175.1, GCA_900099785.1 GCA_000091545.1, GCA_003966975.1, GCA_900109925.1, GCA_000413895.1, GCA_016937655.1 GCA_004363075.1, GCA_028737205.1, GCA_000815225.1, GCA_016127955.1, GCA_003688285.1 GCA_000012965.1, GCA_023497905.1, GCA_002307495.1, GCA_014635325.1, GCA_004353905.1 GCA_003863335.1, GCA_019710635.1, GCA_000165735.1, GCA_900112405.1, GCA_016862175.1 GCA_001436255.1, GCA_900104805.1, GCA_003019655.1, GCA_001444405.1, GCA_014137945.1 GCA_000697965.2, GCA_015326295.1, GCA_900129245.1, GCA_003254355.1, GCA_014196125.1 GCA_000143085.1, GCA_004135645.1, GCA_015666175.1, GCA_014171495.1, GCA_013460135.1 GCA_014203355.1, GCA_005771435.1, GCA_023499215.1, GCA_002081795.1, GCA_014211955.1 GCA_014696875.1, GCA_014646895.1, GCA_013410375.1, GCA_014337215.1, GCA_003254115.1 GCA_002020875.1, GCA_001298545.1, GCA_024158125.1, GCA_013002705.1, GCA_014489615.1 GCA_000330885.1, GCA_000231445.2, GCA_900141785.1, GCA_003612015.1, GCA_000010205.1 GCA_900119105.1, GCA_010378095.1, GCA_000722995.1, GCA_900445015.1, GCA_022865105.1 GCA_003046475.1, GCA_014268595.2, GCA_000024325.1, GCA_009184865.1, GCA_004792695.1 GCA_002006355.2, GCA_019464535.1, GCA_004137355.1, GCA_014673215.1, GCA_003751945.1 GCA_008698145.1, GCA_016907695.1, GCA_000394035.1, GCA_009696465.1, GCA_000146165.2 GCA_018069865.1, GCA_900103325.1, GCA_000025645.1, GCA_016132445.1, GCA_002831565.1 GCA_003590835.1, GCA_003114835.3, GCA_005885815.1, GCA_900199215.1, GCA_009208775.1 GCA_000759055.1, GCA_027587195.1, GCA_003002915.1, GCA_025567085.1, GCA_010686685.1 GCA_000186385.1, GCA_002159895.1, GCA_008033215.1, GCA_002101395.1, GCA_008369065.1 GCA_004310665.1, GCA_019331715.1, GCA_002797875.1, GCA_900141985.1, GCA_025139665.1 GCA_022179065.1, GCA_000196435.1, GCA_014982515.1, GCA_900105045.1, GCA_900187295.1 GCA_012927125.1, GCA_021044425.1, GCA_014644515.1, GCA_017526105.1, GCA_004006175.1 GCA_003052585.1, GCA_014656055.1, GCA_004116335.1, GCA_014699135.1, GCA_001152565.1 GCA_000242455.3, GCA_014652555.1, GCA_023509475.1, GCA_003030905.1, GCA_006007885.1 GCA_021032705.1, GCA_002802915.1, GCA_900129285.1, GCA_024259865.1, GCA_016907435.1 GCA_003595215.1, GCA_000160455.2, GCA_900100695.1, GCA_003994395.1, GCA_006385175.1 GCA_009586235.1, GCA_017874035.1, GCA_011008855.1, GCA_000012345.1, GCA_001907205.1 GCA_900067125.1, GCA_011947285.1, GCA_900176255.2, GCA_014075995.1, GCA_019857185.1 GCA_014489475.1, GCA_013303125.1, GCA_002026305.1, GCA_000020685.1, GCA_020523965.1 GCA_003024155.1, GCA_003609575.1, GCA_000238215.1, GCA_003294055.1, GCA_900177245.1 GCA_900103485.1, GCA_004022265.1, GCA_001435445.1, GCA_000179635.2, GCA_001547995.1 GCA_014640115.1, GCA_900094955.1, GCA_003610535.1, GCA_900176335.1, GCA_921293875.1 GCA_014773265.1, GCA_004769615.1, GCA_900109725.1, GCA_016757275.1, GCA_001434935.1 GCA_900460525.1, GCA_003226895.2, GCA_009600605.1, GCA_000190635.1, GCA_900105805.1 GCA_020782245.1, GCA_002407185.1, GCA_001460935.1, GCA_002078095.1, GCA_019061145.1 GCA_014138045.1, GCA_008364955.1, GCA_019930545.1, GCA_012913625.1, GCA_900215415.1 GCA_002020355.1, GCA_001870205.1, GCA_022669045.1, GCA_000019505.1, GCA_000093085.1 GCA_008369105.1, GCA_001996005.1, GCA_010731535.1, GCA_900496965.1, GCA_013166575.1 GCA_900119845.1, GCA_016722965.1, GCA_016765655.2, GCA_023227765.1, GCA_018289135.1 GCA_900142215.1, GCA_014138425.1, GCA_003600795.1, GCA_001465545.3, GCA_021655555.1 GCA_014174405.1, GCA_000194135.1, GCA_001660815.1, GCA_905397435.1, GCA_015707995.1 GCA_005280215.1, GCA_001678495.1, GCA_004404055.1, GCA_014196635.1, GCA_009674485.1 GCA_016900595.1, GCA_014156615.1, GCA_003003095.1, GCA_021272385.1, GCA_900109565.1 GCA_000582515.1, GCA_900476215.1, GCA_019218285.1, GCA_020149575.1, GCA_001748225.1 GCA_004403345.1, GCA_004684055.1, GCA_000613045.3, GCA_003952265.1, GCA_002220755.1 GCA_002896855.1, GCA_014257025.1, GCA_003545875.1, GCA_018324385.1, GCA_014836395.1 GCA_014651795.1, GCA_000023145.1, GCA_014705715.1, GCA_002993325.1, GCA_002355955.1 GCA_900454225.1, GCA_004924335.1, GCA_014635625.1, GCA_914271435.1, GCA_003385495.1 GCA_024169165.1, GCA_000315015.1, GCA_001558935.2, GCA_004005855.1, GCA_000299915.1 GCA_019890615.1, GCA_014645315.1, GCA_003606345.3, GCA_004851605.1, GCA_002073255.2 GCA_003788585.1, GCA_011927835.1, GCA_019665765.1, GCA_016862695.1, GCA_900451005.1 GCA_000024565.1, GCA_900444995.1, GCA_000444995.1, GCA_002849615.1, GCA_003012055.1 GCA_009914055.1, GCA_003581585.1, GCA_013416015.1, GCA_006120725.1, GCA_011044995.1 GCA_001953875.1, GCA_004214895.1, GCA_000236685.1, GCA_019375915.1, GCA_002139935.1 GCA_003337305.1, GCA_004795975.1, GCA_005048155.1, GCA_004803635.1, GCA_002835735.1 GCA_000184705.1, GCA_900113645.1, GCA_004768445.1, GCA_000012805.1, GCA_004291315.1 GCA_019272935.1, GCA_900099695.1, GCA_900143105.1, GCA_902506355.1, GCA_016767175.1 GCA_002082585.1, GCA_003626515.1, GCA_004803795.1, GCA_000218235.1, GCA_900107125.1 GCA_021739025.1, GCA_013408335.1, GCA_008630495.1, GCA_017876625.1, GCA_022229005.1 GCA_007991175.1, GCA_900099965.1, GCA_014836905.1, GCA_011022235.1, GCA_016722765.1 GCA_009547075.1, GCA_002982015.1, GCA_006716815.1, GCA_019599335.1, GCA_900107645.1 GCA_900461105.1, GCA_016583985.1, GCA_900111195.1, GCA_008274655.1, GCA_900475675.1 GCA_004349085.1, GCA_001940285.1, GCA_009646335.1, GCA_016909185.1, GCA_019334125.1 GCA_018362975.1, GCA_008368715.1, GCA_001183985.1, GCA_004363235.1, GCA_013385145.1 GCA_900452925.1, GCA_017592545.1, GCA_011762185.1, GCA_011762045.1, GCA_002795425.1 GCA_004339805.1, GCA_001579945.1, GCA_014892575.1, GCA_002504285.1, GCA_017869155.1 GCA_004028275.1, GCA_014145335.1, GCA_000521505.1, GCA_001411805.1, GCA_001544915.2 GCA_000018225.1, GCA_014836765.1, GCA_000754095.2, GCA_900188055.1, GCA_016900115.1 GCA_003367905.1, GCA_001908725.1, GCA_023472875.1, GCA_900177685.1, GCA_902860115.1 GCA_019457905.1, GCA_019693255.1, GCA_009909185.1, GCA_014202275.1, GCA_003355495.1 GCA_001752395.1, GCA_003952345.1, GCA_000338055.1, GCA_003977595.1, GCA_005484945.1 GCA_000009985.1, GCA_003259265.1, GCA_002286935.1, GCA_005860805.1, GCA_024496245.1 GCA_014191605.1, GCA_018255875.1, GCA_900108425.1, GCA_017742215.1, GCA_000671395.1 GCA_007856155.1, GCA_005518285.1, GCA_014652095.1, GCA_016806835.2, GCA_001277995.1 GCA_003337215.1, GCA_000019665.1, GCA_003253995.1, GCA_009617595.1, GCA_022374935.2 GCA_003386195.1, GCA_900130015.1, GCA_000317535.1, GCA_017353255.1, GCA_000284155.1 GCA_016654015.1, GCA_011742555.1, GCA_000520015.2, GCA_900110035.1, GCA_003475485.1 GCA_002899825.2, GCA_008704445.1, GCA_007625165.1, GCA_000175375.1, GCA_011290485.1 GCA_003610915.1, GCA_014651855.1, GCA_002158865.1, GCA_000599865.1, GCA_015689475.1 GCA_000741785.1, GCA_014643755.1, GCA_008830225.1, GCA_017309605.1, GCA_025289935.1 GCA_002086165.1, GCA_003014575.1, GCA_001881495.1, GCA_009498735.1, GCA_004801395.1 GCA_003966145.1, GCA_014643635.1, GCA_000590885.1, GCA_001273775.1, GCA_001050435.1 GCA_022008395.1, GCA_001514145.1, GCA_000021005.1, GCA_001461035.1, GCA_004349135.1 GCA_025994195.1, GCA_020149915.1, GCA_000020945.1, GCA_001644565.1, GCA_004348825.1 GCA_011601345.1, GCA_000023265.1, GCA_001026905.1, GCA_023955735.1, GCA_002614725.1 GCA_016765775.1, GCA_019656455.1, GCA_000612965.1, GCA_000015725.1, GCA_001854525.1 GCA_019880555.1, GCA_023008165.1, GCA_900129345.1, GCA_000015505.1, GCA_014636275.1 GCA_014656035.1, GCA_014042355.1, GCA_900106775.1, GCA_900111115.1, GCA_000369005.1 GCA_014287895.1, GCA_014645495.1, GCA_000342105.1, GCA_001884065.1, GCA_900105185.1 GCA_003244315.1, GCA_018310345.1, GCA_009884975.1, GCA_015694725.1, GCA_003258295.1 GCA_003070865.1, GCA_900609065.1, GCA_900105475.1, GCA_018282095.1, GCA_014229345.1 GCA_014199205.1, GCA_005144885.1, GCA_016583625.1, GCA_013155395.1, GCA_003233845.1 GCA_017163705.1, GCA_004101845.1, GCA_015169395.1, GCA_004745955.1, GCA_002250055.1 GCA_023283565.1, GCA_001696605.3, GCA_900230255.1, GCA_014638995.1, GCA_000317835.1 GCA_006476605.1, GCA_014837295.1, GCA_020447175.2, GCA_016801985.1, GCA_008698085.1 GCA_017743015.1, GCA_003173615.1, GCA_004792685.1, GCA_014652275.1, GCA_000159635.1 GCA_007748015.1, GCA_019218265.1, GCA_003130705.1, GCA_014201885.1, GCA_004345565.1 GCA_003752505.1, GCA_004006535.1, GCA_001636295.1, GCA_014640615.1, GCA_016908875.1 GCA_007828725.1, GCA_014207985.1, GCA_018437225.1, GCA_024294965.1, GCA_002205635.1 GCA_900112045.1, GCA_002835805.1, GCA_005887615.1, GCA_016757735.1, GCA_008923205.2 GCA_003363135.1, GCA_019645815.1, GCA_004349065.1, GCA_014705705.1, GCA_001975705.1 GCA_900115655.1, GCA_002706745.1, GCA_013377785.1, GCA_016901735.1, GCA_001653565.1 GCA_000189295.2, GCA_001583435.1, GCA_002005485.1, GCA_900113845.1, GCA_003571725.1 GCA_019778565.1, GCA_001611795.1, GCA_004402215.1, GCA_014646055.1, GCA_900104185.1 GCA_002849795.1, GCA_009762775.1, GCA_003606365.2, GCA_900459175.1, GCA_003112735.1 GCA_002926065.1, GCA_015689455.1, GCA_002285285.1, GCA_016056295.1, GCA_000986795.1 GCA_020084865.1, GCA_018968685.1, GCA_022511585.1, GCA_003051865.1, GCA_003097515.1 GCA_001190745.1, GCA_902859875.1, GCA_007559435.1, GCA_004365765.1, GCA_014202215.1 GCA_001625325.1, GCA_014636255.1, GCA_900188085.1, GCA_011516865.1, GCA_001437075.1 GCA_014297575.1, GCA_002157205.1, GCA_016918095.1, GCA_003065405.1, GCA_900188375.1 GCA_000255115.3, GCA_007859815.1, GCA_019645855.1, GCA_013363995.1, GCA_000477435.1 GCA_003001935.1, GCA_004339565.1, GCA_000741535.1, GCA_012030695.1, GCA_026460885.1 GCA_022509905.1, GCA_014635065.1, GCA_014385005.1, GCA_900177715.1, GCA_014204945.1 GCA_016103465.1, GCA_015207655.1, GCA_001700315.1, GCA_007830095.1, GCA_900454935.1 GCA_900187945.1, GCA_014306155.1, GCA_004365915.1, GCA_014284275.1, GCA_000474275.2 GCA_003610635.1, GCA_002973595.1, GCA_002023715.1, GCA_009696575.1, GCA_000258175.1 GCA_004563945.1, GCA_900631945.1, GCA_001544495.2, GCA_003150935.1, GCA_018394055.1 GCA_004770055.1, GCA_029026745.1, GCA_900230225.1, GCA_014648835.1, GCA_014646915.1 GCA_027886705.1, GCA_009730395.1, GCA_000350545.1, GCA_014651455.1, GCA_000280925.3 GCA_004137345.1, GCA_900107885.1, GCA_001999225.1, GCA_002028545.1, GCA_000147055.1 GCA_004965515.1, GCA_014596935.1, GCA_026275365.1, GCA_000176035.2, GCA_005217615.1 GCA_001717955.1, GCA_900167915.1, GCA_018069925.1, GCA_001481395.1, GCA_009184845.1 GCA_024704525.1, GCA_002983865.1, GCA_012911565.1, GCA_009728485.1, GCA_002849655.1 GCA_019844095.1, GCA_001577525.1, GCA_003208435.1, GCA_000154465.1, GCA_014191245.1 GCA_003994485.1, GCA_014639435.1, GCA_004310345.1, GCA_023684385.1, GCA_001399755.1 GCA_014837165.1, GCA_003236295.1, GCA_014672695.1, GCA_014648315.1, GCA_003173595.1 GCA_000215645.1, GCA_001485145.1, GCA_014199195.1, GCA_000250875.1, GCA_018129025.1 GCA_004341915.1, GCA_000009905.1, GCA_000017865.1, GCA_000807855.2, GCA_000160835.1 GCA_014651895.1, GCA_004402595.1, GCA_008123515.1, GCA_000211495.1, GCA_014268805.2 GCA_024171865.1, GCA_014230145.1, GCA_003353065.1, GCA_003112775.1, GCA_900148835.1 GCA_000346295.1, GCA_014197435.1, GCA_005931095.1, GCA_000015025.1, GCA_000264945.2 GCA_009755355.1, GCA_014650435.1, GCA_900114385.1, GCA_900460995.1, GCA_003284585.1 GCA_004135345.1, GCA_019391655.1, GCA_900113285.1, GCA_014641715.1, GCA_016587375.1 GCA_900129295.1, GCA_011290505.1, GCA_004634245.1, GCA_003722335.1, GCA_903886475.1 GCA_900094585.1, GCA_000189775.3, GCA_001375655.1, GCA_003594815.1, GCA_002189065.1 GCA_019431455.1, GCA_003952025.1, GCA_002332425.1, GCA_000333015.1, GCA_003217475.1 GCA_014656395.1, GCA_025234735.1, GCA_025567485.1, GCA_900109145.1, GCA_900128965.1 GCA_000196035.1, GCA_012222825.1, GCA_900060185.1, GCA_001907295.1, GCA_020810675.1 GCA_014206875.1, GCA_001886875.1, GCA_014982975.1, GCA_003315295.1, GCA_000195955.2 GCA_014651875.1, GCA_000177635.2, GCA_009617855.1, GCA_016889065.1, GCA_004337505.1 GCA_000442315.1, GCA_008373755.1, GCA_007859945.1, GCA_011682145.1, GCA_007280575.1 GCA_003311635.1, GCA_008014345.1, GCA_003634275.1, GCA_002217735.1, GCA_003112435.1 GCA_002934665.1, GCA_900097965.1, GCA_000816495.1, GCA_002356115.1, GCA_003863965.1 GCA_011399095.1, GCA_003576455.2, GCA_014651015.1, GCA_900170035.1, GCA_003055045.1 GCA_022179685.1, GCA_002927635.1, GCA_003122085.1, GCA_002102265.1, GCA_000785435.2 GCA_010729305.1, GCA_008931805.1, GCA_000195295.1, GCA_014212315.1, GCA_025209975.1 GCA_900106055.1, GCA_014836595.1, GCA_001524625.2, GCA_900096895.1, GCA_021728415.1 GCA_008386505.1, GCA_000233775.1, GCA_002811925.1, GCA_011927665.1, GCA_000183135.1 GCA_014200015.1, GCA_003709565.1, GCA_018332875.1, GCA_004353895.1, GCA_017498565.1 GCA_000964225.1, GCA_004343305.1, GCA_013409745.1, GCA_014649115.1, GCA_006717075.1 GCA_007714185.1, GCA_020525665.1, GCA_003369575.1, GCA_001702155.1, GCA_900087655.1 GCA_016863135.1, GCA_001434695.1, GCA_018598225.1, GCA_014397115.1, GCA_900105785.1 GCA_900176275.1, GCA_900142675.1, GCA_002020135.1, GCA_009830125.1, GCA_001908105.1 GCA_000016745.1, GCA_004328625.1, GCA_008122505.1, GCA_900110145.1, GCA_900114945.1 GCA_003814885.1, GCA_004786015.1, GCA_004323185.1, GCA_000020485.1, GCA_009758015.1 GCA_900100975.1, GCA_007197735.1, GCA_026072915.1, GCA_014655855.1, GCA_000213975.1 GCA_001023575.1, GCA_016028795.1, GCA_017876315.1, GCA_014653695.1, GCA_009709575.1 GCA_028330895.1, GCA_019209835.1, GCA_015352445.1, GCA_014698835.1, GCA_014263315.1 GCA_000166695.1, GCA_019900845.1, GCA_003850565.1, GCA_002797855.1, GCA_028401405.1 GCA_009857535.1, GCA_000244895.1, GCA_008369445.1, GCA_013377275.1, GCA_014197015.1 GCA_018372105.1, GCA_009789575.1, GCA_012037645.1, GCA_014216335.1, GCA_000941055.1 GCA_014648115.1, GCA_900105055.1, GCA_002351485.1, GCA_001436615.1, GCA_001262605.1 GCA_017876675.1, GCA_022370635.3, GCA_003259335.1, GCA_009789675.1, GCA_007750855.1 GCA_000416985.1, GCA_014196255.1, GCA_001442625.1, GCA_016862875.1, GCA_003265225.1 GCA_004217615.1, GCA_003721225.1, GCA_943590815.1, GCA_002954265.1, GCA_000504525.1 GCA_008364325.2, GCA_020886695.1, GCA_001907195.1, GCA_004359575.1, GCA_014284475.1 GCA_023195925.1, GCA_002903165.1, GCA_023380265.1, GCA_014202825.1, GCA_004364585.1 GCA_000508165.1, GCA_900108025.1, GCA_003011125.1, GCA_015223195.1, GCA_007997305.1 GCA_003606385.1, GCA_016862975.1, GCA_900113825.1, GCA_900603025.1, GCA_014195675.1 GCA_013407955.1, GCA_020736005.1, GCA_013141765.1, GCA_007827295.1, GCA_002117005.1 GCA_902459575.1, GCA_003516125.3, GCA_014694315.1, GCA_014641075.1, GCA_900111365.1 GCA_009931295.1, GCA_003028415.1, GCA_017357045.1, GCA_005889725.1, GCA_000946815.1 GCA_003813885.1, GCA_014193675.1, GCA_900143255.1, GCA_020783335.1, GCA_004342405.1 GCA_008727865.1, GCA_018141425.1, GCA_022478115.1, GCA_007754425.1, GCA_003202165.1 GCA_025384895.1, GCA_000185805.1, GCA_003052265.1, GCA_003610555.1, GCA_001508275.1 GCA_003013675.1, GCA_012767755.2, GCA_007096385.1, GCA_003967355.1, GCA_024346605.1 GCA_014203325.1, GCA_013201155.1, GCA_016728785.1, GCA_004217215.1, GCA_900090235.1 GCA_004217445.1, GCA_013409645.1, GCA_024170735.1, GCA_020546525.1, GCA_000015125.1 GCA_001641755.2, GCA_001039065.1, GCA_014646655.1, GCA_014650795.1, GCA_004569585.1 GCA_003945325.1, GCA_900217795.1, GCA_003337565.1, GCA_900116225.1, GCA_002368295.1 GCA_002302565.1, GCA_019898765.1, GCA_004216665.1, GCA_014649335.1, GCA_014306095.1 GCA_004139715.1, GCA_900112765.1, GCA_008033155.1, GCA_900188165.1, GCA_003419895.1 GCA_900156635.1, GCA_003385895.1, GCA_004126535.1, GCA_904848665.1, GCA_012927165.1 GCA_004363305.1, GCA_001746835.1, GCA_014836325.1, GCA_009906855.1, GCA_014201775.1 GCA_014196505.1, GCA_014284175.1, GCA_000186885.1, GCA_000786575.1, GCA_000196495.1 GCA_014655955.1, GCA_020164585.1, GCA_011759705.1, GCA_001857925.1, GCA_000063585.1 GCA_014656435.1, GCA_020329485.1, GCA_020628455.1, GCA_014117215.1, GCA_002127535.1 GCA_024895295.1, GCA_007748195.1, GCA_009882935.1, GCA_006352065.1, GCA_003369925.1 GCA_020510525.1, GCA_001707825.1, GCA_013004005.1, GCA_011067135.1, GCA_014905115.1 GCA_014145615.1, GCA_019048625.1, GCA_900302485.1, GCA_002937115.1, GCA_024296925.1 GCA_000012925.1, GCA_900111025.1, GCA_006438825.1, GCA_003963535.1, GCA_014652615.1 GCA_018327645.1, GCA_014649875.1, GCA_004684805.1, GCA_002276555.1, GCA_900112685.1 GCA_900103515.1, GCA_002749945.1, GCA_002946835.1, GCA_017876405.1, GCA_005886755.1 GCA_900104525.1, GCA_000196135.1, GCA_000145275.1, GCA_013466425.1, GCA_003815695.1 GCA_003148475.1, GCA_002101785.1, GCA_900156375.1, GCA_000009145.1, GCA_026230175.1 GCA_000312665.1, GCA_003858645.1, GCA_014636235.1, GCA_014297355.1, GCA_000960005.1 GCA_000767165.1, GCA_001766745.1, GCA_001190785.1, GCA_009730315.1, GCA_014635885.1 GCA_019968625.1, GCA_008831305.1, GCA_003268855.1, GCA_003610735.1, GCA_003576595.1 GCA_020735565.1, GCA_003202405.1, GCA_000955965.1, GCA_011040455.1, GCA_002727185.1 GCA_000430995.1, GCA_024749065.1, GCA_015865035.1, GCA_012641515.1, GCA_000522545.2 GCA_001435875.1, GCA_900106015.1, GCA_001522635.2, GCA_900106795.1, GCA_009296175.1 GCA_014042165.1, GCA_002946355.1, GCA_002355535.1, GCA_001663675.1, GCA_001637325.1 GCA_014138495.1, GCA_017874215.1, GCA_008087665.1, GCA_012328925.1, GCA_006716125.1 GCA_000585235.1, GCA_010093235.1, GCA_000318135.1, GCA_009939195.1, GCA_900115055.1 GCA_900108035.1, GCA_023380025.1, GCA_014204865.1, GCA_011682055.1, GCA_003143555.1 GCA_009728145.1, GCA_016862095.1, GCA_007785815.1, GCA_900078705.1, GCA_028550595.1 GCA_914271485.1, GCA_900111235.1, GCA_002104675.1, GCA_014646095.1, GCA_013393365.1 GCA_014699055.1, GCA_009695875.1, GCA_900104885.1, GCA_900094735.1, GCA_000017805.1 GCA_014489535.1, GCA_002215215.1, GCA_900110695.1, GCA_000808015.1, GCA_003148865.1 GCA_026191375.1, GCA_900186995.1, GCA_008180195.1, GCA_004345645.1, GCA_002980495.1 GCA_900101905.1, GCA_000236565.2, GCA_004135385.1, GCA_003054555.1, GCA_001050535.1 GCA_000164905.1, GCA_002943495.1, GCA_000987835.1, GCA_014649415.1, GCA_003387075.1 GCA_900129815.1, GCA_003202435.1, GCA_000196275.1, GCA_010731695.1, GCA_003148645.1 GCA_001302585.1, GCA_000632965.1, GCA_016587435.1, GCA_021432765.1, GCA_004331645.1 GCA_900039485.1, GCA_014636095.1, GCA_023218935.1, GCA_002238045.1, GCA_002368075.1 GCA_000212415.1, GCA_024764965.1, GCA_004768785.1, GCA_900445365.1, GCA_014651815.1 GCA_000443165.1, GCA_004346035.1, GCA_900156345.1, GCA_900220965.1, GCA_016741875.1 GCA_009758415.1, GCA_900459045.1, GCA_001950255.1, GCA_004421005.1, GCA_021391395.1 GCA_002243515.1, GCA_001437665.1, GCA_000468635.1, GCA_001434145.1, GCA_009720865.1 GCA_005146945.1, GCA_011516645.4, GCA_023573565.1, GCA_002149015.1, GCA_900142385.1 GCA_900111395.1, GCA_900104125.1, GCA_003367395.1, GCA_002892185.1, GCA_900100495.1 GCA_000237085.1, GCA_014842915.1, GCA_021026295.1, GCA_020532605.1, GCA_013204855.1 GCA_014205115.1, GCA_028745505.1, GCA_019711355.1, GCA_016428585.1, GCA_009827435.1 GCA_008064665.1, GCA_000160035.2, GCA_014235185.1, GCA_021560075.1, GCA_003096095.1 GCA_900107535.1, GCA_020889705.1, GCA_000284275.1, GCA_003340495.1, GCA_000497245.1 GCA_025567365.1, GCA_009827405.1, GCA_000008445.1, GCA_011299095.1, GCA_004403415.1 GCA_006376685.1, GCA_012273055.1, GCA_900106805.1, GCA_003634525.1, GCA_900113265.1 GCA_009831235.1, GCA_020907985.1, GCA_923077115.1, GCA_000316515.1, GCA_900009265.2 GCA_026340005.1, GCA_014235765.1, GCA_018122625.2, GCA_001866005.1, GCA_006539565.1 GCA_003399445.1, GCA_013201935.1, GCA_003259505.1, GCA_004342165.1, GCA_001950325.1 GCA_900104235.1, GCA_001758465.1, GCA_003003355.1, GCA_900129375.1, GCA_003324525.1 GCA_007991935.1, GCA_900230285.1, GCA_900104515.1, GCA_946903285.1, GCA_900112845.1 GCA_016623495.1, GCA_900104685.1, GCA_900106095.1, GCA_022568835.1, GCA_025564065.1 GCA_003584745.1, GCA_004005845.1, GCA_025143605.1, GCA_000832125.1, GCA_014070455.1 GCA_900167105.1, GCA_018403345.1, GCA_000009765.2, GCA_014647535.1, GCA_003814745.1 GCA_000212375.1, GCA_016863215.1, GCA_006385555.1, GCA_009711465.1, GCA_008180155.1 GCA_001278075.1, GCA_002086285.1, GCA_003149745.1, GCA_001443665.1, GCA_016651435.1 GCA_007035805.1, GCA_000826685.1, GCA_021442325.1, GCA_014654205.1, GCA_003987225.1 GCA_014199525.1, GCA_000214375.1, GCA_025311475.1, GCA_014640905.1, GCA_003340555.1 GCA_021278985.1, GCA_014384705.1, GCA_009823805.1, GCA_001270065.2, GCA_013307205.1 GCA_002846995.1, GCA_002173775.1, GCA_900102505.1, GCA_900156255.1, GCA_016775155.1 GCA_003725295.1, GCA_004363295.1, GCA_013283855.1, GCA_002327105.1, GCA_006715055.1 GCA_003367235.1, GCA_018918345.1, GCA_014229645.1, GCA_008120875.1, GCA_900130035.1 GCA_020991065.1, GCA_013410775.1, GCA_020450145.1, GCA_019797805.1, GCA_019130065.1 GCA_000314975.1, GCA_900478045.1, GCA_903819165.1, GCA_001936175.1, GCA_019670485.1 GCA_001440035.1, GCA_002924625.1, GCA_014736495.1, GCA_013350005.1, GCA_012927325.1 GCA_900115775.1, GCA_016458275.1, GCA_900106955.1, GCA_013346665.1, GCA_014230345.1 GCA_003515105.1, GCA_010500915.1, GCA_008040165.1, GCA_003850045.1, GCA_001186155.3 GCA_001617355.1, GCA_003601975.1, GCA_002157835.1, GCA_013694095.1, GCA_008704425.1 GCA_001543925.1, GCA_016863495.1, GCA_003951095.1, GCA_003007735.2, GCA_001435585.1 GCA_003014535.1, GCA_016522505.1, GCA_001413955.1, GCA_011038645.2, GCA_000499665.2 GCA_900096565.1, GCA_001467975.1, GCA_003320875.1, GCA_001594015.1, GCA_018919395.1 GCA_002284535.1, GCA_001998865.1, GCA_002179915.1, GCA_003955715.1, GCA_002086155.1 GCA_900109075.1, GCA_028607085.1, GCA_002362315.1, GCA_023571765.1, GCA_001593605.1 GCA_017876165.1, GCA_900129365.1, GCA_000368025.1, GCA_000576305.1, GCA_013626205.1 GCA_001439735.1, GCA_002102415.1, GCA_900114415.1, GCA_003149185.1, GCA_900104415.1 GCA_901482695.1, GCA_900169565.1, GCA_014638675.1, GCA_003019675.1, GCA_900445155.1 GCA_025447535.1, GCA_007830265.1, GCA_000508225.1, GCA_020217465.1, GCA_007000325.2 GCA_019431045.1, GCA_003364195.1, GCA_900167455.1, GCA_900177005.1, GCA_000317675.1 GCA_001293525.1, GCA_001467025.1, GCA_900113935.1, GCA_000225955.1, GCA_021738965.1 GCA_018207515.1, GCA_000226625.1, GCA_016862935.1, GCA_004402375.1, GCA_002209385.1 GCA_020595095.1, GCA_020985365.1, GCA_004843545.1, GCA_002838765.1, GCA_015351405.1 GCA_001544755.2, GCA_900091495.1, GCA_012034175.1, GCA_002250565.1, GCA_003002895.1 GCA_017814435.1, GCA_900113865.1, GCA_000816305.1, GCA_001306145.1, GCA_007830125.1 GCA_003236385.1, GCA_001050375.1, GCA_001434705.1, GCA_002374855.1, GCA_900182695.1 GCA_001723355.1, GCA_000498495.1, GCA_006385255.1, GCA_000172975.1, GCA_001444505.1 GCA_900455105.1, GCA_012927045.1, GCA_014644195.1, GCA_001267925.1, GCA_019469265.1 GCA_000023445.1, GCA_900114615.1, GCA_013234275.1, GCA_005239095.1, GCA_002284575.1 GCA_000297115.1, GCA_009758125.1, GCA_003383615.1, GCA_001708405.1, GCA_026723765.1 GCA_009649175.1, GCA_001887715.1, GCA_020782235.1, GCA_020510565.1, GCA_002846395.1 GCA_028982185.1, GCA_010727475.1, GCA_003581605.1, GCA_014874095.1, GCA_016907845.1 GCA_015690345.1, GCA_016919175.1, GCA_001995825.2, GCA_004524855.1, GCA_016592575.1 GCA_011398155.1, GCA_001865575.2, GCA_020735365.1, GCA_012396435.1, GCA_015221875.1 GCA_000963925.1, GCA_010686655.1, GCA_914271545.1, GCA_011038655.2, GCA_003605405.2 GCA_013407765.1, GCA_024809475.1, GCA_000153305.1, GCA_000178875.2, GCA_019890915.1 GCA_024436055.1, GCA_900101895.1, GCA_002906925.1, GCA_900454665.1, GCA_001277345.1 GCA_003986955.1, GCA_000191045.1, GCA_024170285.1, GCA_013389765.1, GCA_000828635.1 GCA_011057975.1, GCA_003408705.1, GCA_004770995.1, GCA_009646115.1, GCA_900102925.1 GCA_900465025.1, GCA_000178115.2, GCA_000247995.2, GCA_023822065.1, GCA_000760695.4 GCA_006349365.1, GCA_014141535.1, GCA_003315135.1, GCA_900637205.1, GCA_027922085.1 GCA_009711095.1, GCA_022869005.1, GCA_024764795.1, GCA_900324035.1, GCA_002197845.1 GCA_018390595.1, GCA_000315075.1, GCA_004124235.1, GCA_014203045.1, GCA_014654365.1 GCA_016863615.1, GCA_014648775.1, GCA_900188405.1, GCA_900103545.1, GCA_020176155.1 GCA_014645135.1, GCA_000011245.1, GCA_004344915.1, GCA_900197875.1, GCA_014192285.1 GCA_013391765.2, GCA_000968375.1, GCA_010726245.1, GCA_900141935.1, GCA_020526085.1 GCA_002005145.1, GCA_001017755.1, GCA_007990265.1, GCA_001543105.1, GCA_019331735.1 GCA_015767775.1, GCA_018417475.1, GCA_001975725.1, GCA_004349195.1, GCA_018223745.1 GCA_009758205.1, GCA_022647425.1, GCA_025148125.1, GCA_001941465.1, GCA_005116465.1 GCA_009739905.1, GCA_004402535.1, GCA_000758665.1, GCA_014650135.1, GCA_021049285.1 GCA_900099645.1, GCA_900129985.1, GCA_007859755.1, GCA_004802635.2, GCA_009735585.1 GCA_014837245.1, GCA_900172225.1, GCA_004339675.1, GCA_000814475.1, GCA_003182595.1 GCA_003851005.1, GCA_014653235.1, GCA_024171765.1, GCA_900115185.1, GCA_900116615.1 GCA_023515995.1, GCA_001908275.1, GCA_008016755.1, GCA_000183545.3, GCA_028596105.1 GCA_004134775.1, GCA_014650715.1, GCA_002234535.1, GCA_014490445.1, GCA_011742875.1 GCA_023347585.1, GCA_000763805.1, GCA_026000375.1, GCA_000015105.1, GCA_900142445.1 GCA_000304215.1, GCA_000144645.1, GCA_900109465.1, GCA_900460135.1, GCA_900114115.1 GCA_018130765.1, GCA_014295435.1, GCA_000468015.1, GCA_900113175.1, GCA_900453865.1 GCA_000024225.1, GCA_024508375.2, GCA_002208765.2, GCA_003601925.1, GCA_902141845.1 GCA_013283645.1, GCA_000310225.1, GCA_015645445.1, GCA_020278605.1, GCA_900129325.1 GCA_026343955.1, GCA_014192475.1, GCA_000953015.1, GCA_002250835.2, GCA_900182605.1 GCA_000277285.1, GCA_009834925.2, GCA_000242595.3, GCA_010119975.1, GCA_001650715.1 GCA_004519545.1, GCA_000023065.1, GCA_004684905.1, GCA_004011825.1, GCA_000016345.1 GCA_004349215.1, GCA_014925355.1, GCA_021278965.1, GCA_014199915.1, GCA_019801785.1 GCA_016902415.1, GCA_900142245.1, GCA_002807015.1, GCA_002514735.1, GCA_001886815.1 GCA_016919645.1, GCA_000836635.1, GCA_000496735.2, GCA_009708005.2, GCA_000196115.1 GCA_003987345.1, GCA_900142715.1, GCA_015207985.1, GCA_019218685.1, GCA_003710245.1 GCA_900099685.1, GCA_000331995.1, GCA_003287735.1, GCA_018332715.1, GCA_002811195.1 GCA_000953715.1, GCA_001005725.1, GCA_019218635.1, GCA_902459735.1, GCA_003095375.1 GCA_017349315.1, GCA_014649495.1, GCA_007990245.1, GCA_014645895.1, GCA_016901255.1 GCA_027921965.1, GCA_014651595.1, GCA_017310015.1, GCA_028829235.1, GCA_900112265.1 GCA_002777255.1, GCA_016937555.1, GCA_014648695.1, GCA_007989085.1, GCA_002464935.1 GCA_013248965.1, GCA_001514535.1, GCA_900221015.1, GCA_002895565.1, GCA_014199415.1 GCA_003144035.1, GCA_009741275.1, GCA_019140855.1, GCA_004403985.1, GCA_003173755.1 GCA_020521255.1, GCA_014652595.1, GCA_021166585.1, GCA_013407855.1, GCA_000166195.3 GCA_017051665.1, GCA_002309535.1, GCA_005047595.1, GCA_900111385.1, GCA_021271025.1 GCA_024809135.1, GCA_000269985.1, GCA_900188095.1, GCA_000230655.3, GCA_009758175.1 GCA_002162115.2, GCA_011777495.1, GCA_011764545.1, GCA_900116645.1, GCA_900217905.1 GCA_013376105.1, GCA_001523725.1, GCA_021379005.1, GCA_900111945.1, GCA_014647755.1 GCA_000147715.3, GCA_001552035.1, GCA_000166355.1, GCA_014333615.1, GCA_001436315.1 GCA_016892705.1, GCA_016405625.1, GCA_900115835.1, GCA_003317055.1, GCA_001536285.1 GCA_000300005.1, GCA_010983895.1, GCA_007830255.1, GCA_003856525.1, GCA_003867015.1 GCA_029215585.1, GCA_024760485.1, GCA_900103035.1, GCA_011044975.1, GCA_019456675.1 GCA_002843355.2, GCA_016907735.1, GCA_003413745.1, GCA_003096015.2, GCA_009295515.1 GCA_008806385.1, GCA_003148905.1, GCA_001545095.1, GCA_900638065.1, GCA_900104555.1 GCA_016834655.1, GCA_008369785.1, GCA_001887595.1, GCA_900606115.1, GCA_003335255.1 GCA_000012325.1, GCA_009827545.1, GCA_900458705.1, GCA_024029775.1, GCA_006538885.1 GCA_013408295.1, GCA_000020625.1, GCA_020905375.1, GCA_014534685.1, GCA_013394065.1 GCA_014639795.1, GCA_016919065.1, GCA_014650155.1, GCA_000227585.1, GCA_027570155.1 GCA_014385165.1, GCA_018343545.2, GCA_024054455.1, GCA_000498975.2, GCA_001562555.1 GCA_900114125.1, GCA_000521725.1, GCA_008710145.1, GCA_015775515.1, GCA_900637975.1 GCA_021739485.1, GCA_002797685.1, GCA_004786095.1, GCA_900186835.1, GCA_015500095.1 GCA_000214215.1, GCA_016628735.2, GCA_013285525.1, GCA_001870735.1, GCA_003150835.1 GCA_004801305.1, GCA_900102055.1, GCA_014656295.1, GCA_017086365.1, GCA_000306255.2 GCA_024360925.2, GCA_900102075.1, GCA_013200955.2, GCA_900109025.1, GCA_006542295.1 GCA_014646155.1, GCA_029268985.1, GCA_014217805.1, GCA_013167215.1, GCA_004104375.1 GCA_006715765.1, GCA_002846365.1, GCA_900142165.1, GCA_003353425.1, GCA_007859855.1 GCA_002529485.1, GCA_900128895.1, GCA_000219125.2, GCA_900111105.1, GCA_900142575.1 GCA_001457555.1, GCA_004912275.1, GCA_008274785.1, GCA_003201875.1, GCA_001017775.3 GCA_019100565.1, GCA_009377345.1, GCA_007421815.1, GCA_011578045.1, GCA_900403705.1 GCA_000412675.1, GCA_014489515.1, GCA_000235665.2, GCA_904061905.1, GCA_001761545.1 GCA_009600895.1, GCA_001761385.1, GCA_001702215.1, GCA_011682045.1, GCA_004353985.1 GCA_008932225.1, GCA_004364975.1, GCA_009904675.1, GCA_003352185.1, GCA_003600355.1 GCA_001717125.1, GCA_027570195.1, GCA_009690845.1, GCA_026210475.1, GCA_003324675.1 GCA_003217575.1, GCA_014205765.1, GCA_000185705.2, GCA_004217555.1, GCA_000195555.1 GCA_002075285.3, GCA_000069965.1, GCA_002287375.1, GCA_900636765.1, GCA_018688255.1 GCA_000417735.2, GCA_003945385.1, GCA_003932015.2, GCA_003334855.1, GCA_001939735.1 GCA_017357225.1, GCA_009827515.1, GCA_023821985.2, GCA_000306675.3, GCA_023897015.1 GCA_000016065.1, GCA_907164545.1, GCA_014654095.1, GCA_023218175.1, GCA_000478885.1 GCA_017830045.1, GCA_003864295.1, GCA_900111965.1, GCA_019711635.1, GCA_016894345.1 GCA_900167535.1, GCA_000422245.1, GCA_000272005.1, GCA_000376665.1, GCA_003633715.1 GCA_014635125.1, GCA_904848625.1, GCA_900115155.1, GCA_000600335.2, GCA_900002405.2 GCA_018383905.1, GCA_010974945.1, GCA_014652415.1, GCA_009601165.1, GCA_004770615.1 GCA_002055515.1, GCA_009697385.1, GCA_900476255.1, GCA_012584515.1, GCA_900102275.1 GCA_019448235.1, GCA_000025345.1, GCA_007741495.1, GCA_014698335.1, GCA_003925875.1 GCA_900167125.1, GCA_018333175.1, GCA_003966155.1, GCA_007954785.1, GCA_000350085.1 GCA_016415705.1, GCA_001189365.1, GCA_000013605.1, GCA_022213125.1, GCA_900452405.1 GCA_001013905.1, GCA_013376475.1, GCA_003289645.1, GCA_007004765.1, GCA_013141825.1 GCA_014077625.1, GCA_008386565.1, GCA_014467055.1, GCA_023283605.1, GCA_019444005.1 GCA_005049105.1, GCA_000737315.1, GCA_020885575.1, GCA_001051995.2, GCA_014141525.1 GCA_900111985.1, GCA_009742705.1, GCA_017581925.1, GCA_014203665.1, GCA_013211575.1 GCA_004137235.1, GCA_008693785.1, GCA_001025035.1, GCA_015354245.1, GCA_016595555.1 GCA_000949295.1, GCA_903994035.1, GCA_003259835.1, GCA_011758645.1, GCA_017348895.1 GCA_002115725.1, GCA_005047355.1, GCA_006716885.1, GCA_017599305.1, GCA_014197145.1 GCA_002240355.1, GCA_001922305.1, GCA_008189685.1, GCA_001940235.1, GCA_001921845.1 GCA_009556455.1, GCA_000346315.1, GCA_004339015.1, GCA_000315055.1, GCA_009905215.1 GCA_022374875.3, GCA_003730135.1, GCA_014697625.1, GCA_001951155.1, GCA_004353865.1 GCA_004295585.1, GCA_002074155.1, GCA_021233355.1, GCA_900167255.1, GCA_003173575.1 GCA_007830545.1, GCA_011761355.1, GCA_020682705.1, GCA_022014715.1, GCA_022171985.1 GCA_007845675.1, GCA_003330865.1, GCA_014646635.1, GCA_000239275.1, GCA_014649635.1 GCA_017883985.1, GCA_008085895.1, GCA_002289455.1, GCA_016728365.1, GCA_003019925.1 GCA_002221505.1, GCA_900445285.1, GCA_014196535.1, GCA_004008975.1, GCA_026127385.1 GCA_008629695.1, GCA_026108055.1, GCA_002632875.1, GCA_900116175.1, GCA_017312485.1 GCA_004011505.1, GCA_000022325.1, GCA_001613545.1, GCA_000185505.1, GCA_024918935.1 GCA_900129955.1, GCA_024169515.1, GCA_007753265.1, GCA_900102545.1, GCA_001995095.2 GCA_900302505.1, GCA_007993795.1, GCA_001594225.2, GCA_000279145.1, GCA_016862815.1 GCA_010726955.1, GCA_003725775.1, GCA_007995085.1, GCA_003952945.1, GCA_005497185.1 GCA_000817255.2, GCA_015476275.1, GCA_009811795.1, GCA_005144905.1, GCA_000162755.2 GCA_018531165.1, GCA_003722295.1, GCA_021236965.1, GCA_900114715.1, GCA_003626575.1 GCA_000807675.2, GCA_003014695.1, GCA_002441855.2, GCA_014643415.1, GCA_000166935.1 GCA_022845715.1, GCA_009295845.1, GCA_002206625.1, GCA_022749515.1, GCA_014196335.1 GCA_003350545.1, GCA_003362805.1, GCA_001691055.1, GCA_900638655.1, GCA_014648475.1 GCA_000517265.1, GCA_000245015.1, GCA_900105975.1, GCA_001941945.1, GCA_020329505.1 GCA_015689195.1, GCA_004571025.1, GCA_000279995.1, GCA_003863415.1, GCA_002217395.1 GCA_003254745.1, GCA_005406205.1, GCA_900099675.1, GCA_000021285.1, GCA_000973725.1 GCA_022846515.1, GCA_000568815.1, GCA_016917755.1, GCA_021030645.1, GCA_001482405.1 GCA_900142075.1, GCA_026011795.1, GCA_020687785.1, GCA_005144715.1, GCA_027286365.1 GCA_000597865.1, GCA_002370525.2, GCA_900112105.1, GCA_000397065.2, GCA_001050015.1 GCA_024584745.1, GCA_905220735.1, GCA_006874425.1, GCA_900106925.1, GCA_017377855.1 GCA_000284295.1, GCA_014196425.1, GCA_000184435.1, GCA_010078385.1, GCA_000770115.2 GCA_016925615.1, GCA_000236785.2, GCA_900114165.1, GCA_014023275.1, GCA_006443685.1 GCA_003966735.1, GCA_022370835.2, GCA_029268915.1, GCA_003201205.1, GCA_000266885.1 GCA_003347095.1, GCA_002245355.1, GCA_016904755.2, GCA_000160115.1, GCA_020859565.1 GCA_000767835.1, GCA_023283485.1, GCA_004801375.1, GCA_013664685.1, GCA_014207565.1 GCA_000333895.2, GCA_018070025.1, GCA_020463795.1, GCA_002204235.2, GCA_003628305.1 GCA_001715975.1, GCA_009908295.1, GCA_900115045.1, GCA_022870945.1, GCA_000143825.1 GCA_000733295.1, GCA_000016985.1, GCA_003797835.1, GCA_900155965.1, GCA_017813235.1 GCA_000024005.1, GCA_014645195.1, GCA_900111875.1, GCA_002104765.1, GCA_025997855.1 GCA_000155555.1, GCA_003072465.1, GCA_004362245.1, GCA_003944795.1, GCA_900109345.1 GCA_025823185.1, GCA_014284195.1, GCA_900101465.1, GCA_009883795.1, GCA_001514125.1 GCA_009789225.1, GCA_010211765.1, GCA_020097095.1, GCA_001509475.1, GCA_014841135.1 GCA_000015305.1, GCA_001280865.1, GCA_000300095.1, GCA_000025705.1, GCA_003970925.1 GCA_003575965.1, GCA_900188185.1, GCA_004359375.1, GCA_900102835.1, GCA_020735865.1 GCA_021390435.3, GCA_000165125.2, GCA_014775655.1, GCA_018139695.1, GCA_014385265.1 GCA_008933955.1, GCA_028414605.1, GCA_002015115.1, GCA_000010505.1, GCA_001787355.1 GCA_002286695.1, GCA_006569185.2, GCA_000152985.1, GCA_009874335.1, GCA_014697105.1 GCA_001931755.2, GCA_900188415.1, GCA_000007125.1, GCA_003058085.1, GCA_021290925.1 GCA_003696235.1, GCA_000685235.1, GCA_001517975.1, GCA_900114255.1, GCA_900638385.1 GCA_028724705.1, GCA_007004135.1, GCA_014269395.1, GCA_001281175.1, GCA_020640915.1 GCA_007923295.1, GCA_901905185.2, GCA_004025525.1, GCA_900182575.1, GCA_004363175.1 GCA_900182705.1, GCA_900129935.1, GCA_002846495.1, GCA_005843985.1, GCA_014647195.1 GCA_013607875.1, GCA_000214665.1, GCA_009604375.1, GCA_009696445.1, GCA_013267375.1 GCA_000024085.1, GCA_009735645.1, GCA_000696675.2, GCA_902501455.1, GCA_018256955.1 GCA_014836755.1, GCA_004365965.1, GCA_016907015.1, GCA_000185445.1, GCA_003544915.1 GCA_023061185.1, GCA_004791675.1, GCA_008121455.1, GCA_003416885.1, GCA_003346865.1 GCA_020169515.1, GCA_003598715.1, GCA_001708955.1, GCA_007954585.1, GCA_012395815.1 GCA_000018325.1, GCA_018448885.1, GCA_003454775.1, GCA_024105665.1, GCA_006438955.1 GCA_000020845.1, GCA_900112335.1, GCA_009938305.1, GCA_900113445.1, GCA_001705425.1 GCA_001618385.1, GCA_014640335.1, GCA_000215085.1, GCA_014062315.1, GCA_004346845.1 GCA_009761395.1, GCA_003028855.1, GCA_001685015.1, GCA_019904155.1, GCA_000217815.1 GCA_003627095.1, GCA_900067155.1, GCA_003046295.1, GCA_002934625.1, GCA_000020005.1 GCA_002954725.1, GCA_014836415.1, GCA_000818095.1, GCA_000300975.2, GCA_001641635.1 GCA_900163655.1, GCA_000761155.1, GCA_013359945.1, GCA_001042635.1, GCA_002243135.1 GCA_003865405.1, GCA_020907275.1, GCA_019552005.1, GCA_004217175.1, GCA_014647515.1 GCA_002911665.1, GCA_001592935.1, GCA_000723165.1, GCA_003034845.1, GCA_025961475.1 GCA_002920895.1, GCA_900102625.1, GCA_013285305.1, GCA_022267315.1, GCA_000764855.1 GCA_026941245.1, GCA_002865605.1, GCA_003071385.1, GCA_000018405.1, GCA_029224645.1 GCA_900101395.1, GCA_001435235.1, GCA_009910845.1, GCA_013377295.1, GCA_900129045.1 GCA_000219355.1, GCA_027533625.1, GCA_016634425.1, GCA_005844085.1, GCA_010091945.1 GCA_002111585.1, GCA_014892245.1, GCA_003340315.1, GCA_001561915.1, GCA_013376155.1 GCA_028331025.1, GCA_900129465.1, GCA_014641495.1, GCA_013425525.1, GCA_025567285.1 GCA_900091555.1, GCA_007954425.1, GCA_014636495.1, GCA_024453815.1, GCA_026410355.1 GCA_014268585.2, GCA_900114285.1, GCA_013867635.1, GCA_003344965.1, GCA_900115015.1 GCA_008923365.1, GCA_002305895.1, GCA_003755125.1, GCA_001996285.1, GCA_001191605.1 GCA_000181575.2, GCA_900182645.1, GCA_023272795.1, GCA_009856605.1, GCA_004342485.1 GCA_000943515.2, GCA_003544835.1, GCA_014650095.1, GCA_024808255.1, GCA_004343705.1 GCA_003259845.1, GCA_900090325.1, GCA_005818865.1, GCA_900107585.1, GCA_019599145.1 GCA_017583045.1, GCA_000177355.1, GCA_003721595.1, GCA_014656545.1, GCA_000807275.1 GCA_000017545.1, GCA_014643735.1, GCA_900129515.1, GCA_018390555.1, GCA_003719825.1 GCA_019931215.1, GCA_003058325.1, GCA_009685195.1, GCA_006547165.1, GCA_004362865.1 GCA_003820355.1, GCA_000155515.2, GCA_019801215.1, GCA_900103275.1, GCA_006539145.1 GCA_014652195.1, GCA_013377535.1, GCA_013408305.1, GCA_014204535.1, GCA_003386665.1 GCA_003429095.1, GCA_008086205.1, GCA_021391495.1, GCA_900103885.1, GCA_014639075.1 GCA_008329715.1, GCA_900110555.1, GCA_001484625.1, GCA_900188335.1, GCA_002196905.1 GCA_003633775.1, GCA_024112395.1, GCA_014174495.1, GCA_016743775.1, GCA_012933545.1 GCA_900108185.1, GCA_022343725.1, GCA_001514035.1, GCA_001006285.1, GCA_003253545.1 GCA_900233005.1, GCA_000983115.1, GCA_014648495.1, GCA_002752675.1, GCA_900167545.1 GCA_004770895.1, GCA_014836675.1, GCA_016741815.1, GCA_003994465.1, GCA_005795905.1 GCA_001457025.1, GCA_013462805.1, GCA_002632825.1, GCA_022410465.1, GCA_001580945.1 GCA_017874625.1, GCA_001050235.2, GCA_003722355.1, GCA_003363265.1, GCA_000512735.1 GCA_021172045.1, GCA_004117055.1, GCA_003182135.1, GCA_000196155.1, GCA_001704115.1 GCA_018224925.1, GCA_014701095.1, GCA_014640095.1, GCA_004135405.1, GCA_010093185.1 GCA_003350535.1, GCA_004564355.1, GCA_003231285.1, GCA_016653465.1, GCA_006149045.1 GCA_014692495.1, GCA_019430945.1, GCA_006716355.1, GCA_900176135.1, GCA_027921945.1 GCA_001941565.1, GCA_900114085.1, GCA_000731675.1, GCA_025566905.1, GCA_003663885.1 GCA_016862255.1, GCA_018288855.1, GCA_016629665.1, GCA_023702635.1, GCA_025567125.1 GCA_004137085.1, GCA_900100565.1, GCA_900110955.1, GCA_014836615.1, GCA_003019255.1 GCA_014635985.1, GCA_001753675.2, GCA_001757105.1, GCA_000513135.1, GCA_016907475.1 GCA_022701195.1, GCA_002407265.1, GCA_900096915.1, GCA_001580045.1, GCA_001298715.2 GCA_017607425.1, GCA_900113005.1, GCA_003544855.1, GCA_003860465.1, GCA_019711595.1 GCA_027622855.1, GCA_016786215.1, GCA_000314855.2, GCA_000496595.1, GCA_014237875.1 GCA_003387165.1, GCA_000265385.1, GCA_009742725.1, GCA_000364325.1, GCA_003864335.1 GCA_016858125.1, GCA_001697185.1, GCA_005938195.1, GCA_002631205.1, GCA_007992435.1 GCA_002288305.1, GCA_900101955.1, GCA_014643655.1, GCA_008974265.1, GCA_000600105.1 GCA_016862295.1, GCA_013410665.1, GCA_000939975.1, GCA_000222485.1, GCA_014982785.1 GCA_900142975.1, GCA_012940005.1, GCA_009765695.1, GCA_015627105.1, GCA_017565765.1 GCA_009671065.1, GCA_021172145.1, GCA_900637915.1, GCA_003123745.1, GCA_000685215.1 GCA_012295525.1, GCA_000016185.1, GCA_013204795.1, GCA_900089755.1, GCA_008704515.1 GCA_000236705.1, GCA_003149085.1, GCA_003205195.1, GCA_006378125.1, GCA_900114535.1 GCA_013204825.1, GCA_900444665.1, GCA_020735465.1, GCA_016865585.1, GCA_001999945.1 GCA_008693185.1, GCA_010077965.1, GCA_900106985.1, GCA_013415115.1, GCA_007858445.1 GCA_018129525.1, GCA_004122165.1, GCA_005405885.1, GCA_900142875.1, GCA_900112295.1 GCA_001420715.1, GCA_000691225.1, GCA_003382365.1, GCA_014202345.1, GCA_900302445.1 GCA_023700755.1, GCA_015476235.1, GCA_001721045.1, GCA_028330985.1, GCA_000250635.1 GCA_014647635.1, GCA_009650215.1, GCA_900115065.1, GCA_013410615.1, GCA_015159595.1 GCA_012911865.1, GCA_002116635.1, GCA_001564455.1, GCA_009192945.1, GCA_900184295.1 GCA_900107925.1, GCA_017368455.1, GCA_001904715.1, GCA_020328055.1, GCA_017338855.1 GCA_001620265.1, GCA_001756615.1, GCA_001434365.1, GCA_001465345.1, GCA_011927695.1 GCA_010179735.1, GCA_014649995.1, GCA_000427095.1, GCA_014873955.1, GCA_002156545.1 GCA_001440415.1, GCA_009684715.1, GCA_022819085.1, GCA_022459015.1, GCA_021650935.1 GCA_022095695.1, GCA_900187235.1, GCA_900176505.1, GCA_018437275.1, GCA_013114835.1 GCA_013410655.1, GCA_900094975.1, GCA_001767595.1, GCA_004405125.1, GCA_900167905.1 GCA_000023105.1, GCA_018390695.1, GCA_014202765.1, GCA_006861775.1, GCA_900142065.1 GCA_001547755.1, GCA_004362145.1, GCA_020829645.1, GCA_013377885.1, GCA_000964795.1 GCA_001580545.1, GCA_016907925.1, GCA_022370755.2, GCA_016865555.1, GCA_001558255.2 GCA_900112585.1, GCA_002795245.1, GCA_000046845.1, GCA_014642875.1, GCA_000191405.1 GCA_009796305.1, GCA_003815535.1, GCA_902459765.1, GCA_020886175.1, GCA_000732945.1 GCA_900102365.1, GCA_003751935.1, GCA_002009235.1, GCA_000018605.1, GCA_004769575.1 GCA_014644415.1, GCA_006965545.2, GCA_008605885.1, GCA_000008725.1, GCA_000147675.2 GCA_002165375.2, GCA_900459355.1, GCA_009901585.1, GCA_000806415.1, GCA_000196675.2 GCA_000020385.1, GCA_005048855.1, GCA_002892925.1, GCA_004770625.1, GCA_006334925.1 GCA_900185015.1, GCA_004402785.1, GCA_014203025.1, GCA_017569185.1, GCA_900142995.1 GCA_014898175.1, GCA_022509925.1, GCA_019734885.1, GCA_000725365.1, GCA_000238375.4 GCA_900156305.1, GCA_003350445.1, GCA_027920505.1, GCA_008271745.1, GCA_010500895.1 GCA_014201825.1, GCA_019721995.1, GCA_002243385.1, GCA_900105645.1, GCA_900107275.1 GCA_016028515.1, GCA_009687885.1, GCA_014651775.1, GCA_013266765.1, GCA_014651835.1 GCA_011009535.1, GCA_016342785.1, GCA_004799325.1, GCA_000241955.1, GCA_003865155.1 GCA_013410215.1, GCA_001922405.1, GCA_003725735.1, GCA_008704855.1, GCA_025532145.1 GCA_005280275.1, GCA_021654455.1, GCA_025567045.1, GCA_014701195.1, GCA_900205605.1 GCA_001542405.1, GCA_000009085.1, GCA_004152935.1, GCA_000340435.3, GCA_013410745.1 GCA_002086735.1, GCA_001281405.1, GCA_010093305.1, GCA_900452415.1, GCA_014202575.1 GCA_009012935.1, GCA_001890385.1, GCA_014852685.1, GCA_900445025.1, GCA_000517405.1 GCA_013265585.1, GCA_017347585.1, GCA_002023665.2, GCA_000214495.2, GCA_003019695.1 GCA_000368565.1, GCA_000815105.2, GCA_007475525.1, GCA_000710755.1, GCA_013343195.3 GCA_011045025.1, GCA_023278125.1, GCA_003688895.1, GCA_023703465.1, GCA_024363565.1 GCA_000153185.1, GCA_014268375.2, GCA_006874765.1, GCA_002812205.1, GCA_014384885.1 GCA_003001855.1, GCA_003581425.1, GCA_021283305.1, GCA_008831035.1, GCA_001558775.1 GCA_002302635.1, GCA_003718735.1, GCA_003201355.1, GCA_017909455.1, GCA_006385595.1 GCA_004331265.1, GCA_006539505.1, GCA_900114035.1, GCA_001514065.1, GCA_900107415.1 GCA_900096905.1, GCA_001029285.1, GCA_013003985.1, GCA_900156335.1, GCA_009759865.1 GCA_900109935.1, GCA_001247745.1, GCA_019891545.1, GCA_011326725.1, GCA_003217235.1 GCA_022807975.1, GCA_002074095.1, GCA_001653715.1, GCA_014931075.1, GCA_000182745.1 GCA_009695635.1, GCA_003010915.2, GCA_003402615.1, GCA_014524565.1, GCA_024273205.1 GCA_007954275.1, GCA_003860525.1, GCA_029355765.1, GCA_020091505.1, GCA_001029445.1 GCA_026236545.1, GCA_003023845.1, GCA_003574295.1, GCA_029168315.1, GCA_000178855.1 GCA_027158465.1, GCA_007197645.1, GCA_900176325.1, GCA_015210005.1, GCA_000015665.1 GCA_001509405.1, GCA_018604565.1, GCA_002879535.2, GCA_000364845.1, GCA_000612685.1 GCA_014982935.1, GCA_016735115.1, GCA_001855275.1, GCA_014199395.1, GCA_000013165.1 GCA_013403315.1, GCA_013460375.1, GCA_004345745.1, GCA_016890225.1, GCA_002631185.1 GCA_014651035.1, GCA_900186885.1, GCA_003725995.1, GCA_001654455.1, GCA_004331385.1 GCA_000222105.4, GCA_003432035.1, GCA_007362295.1, GCA_003385515.1, GCA_010725725.1 GCA_001639065.2, GCA_015694525.1, GCA_000215105.1, GCA_000407285.1, GCA_004134825.1 GCA_021029865.1, GCA_000764535.1, GCA_002240415.1, GCA_001730225.1, GCA_000730385.1 GCA_013282725.1, GCA_014205295.1, GCA_014649775.1, GCA_014489575.1, GCA_001907235.1 GCA_022803015.1, GCA_012295575.1, GCA_003970355.1, GCA_023656565.1, GCA_000260135.1 GCA_003987405.1, GCA_009676365.1, GCA_000158955.1, GCA_000307875.1, GCA_003007785.1 GCA_016653295.1, GCA_014337155.1, GCA_022810315.1, GCA_900115205.1, GCA_000012685.1 GCA_017829975.1, GCA_900113145.1, GCA_017068375.1, GCA_004359515.1, GCA_900460295.1 GCA_900104305.1, GCA_010731835.1, GCA_016918935.1, GCA_014201975.1, GCA_001956985.1 GCA_021556435.1, GCA_018917285.1, GCA_900120065.1, GCA_003129905.1, GCA_900188035.1 GCA_900111445.1, GCA_008704715.1, GCA_014203695.1, GCA_001722345.1, GCA_001434435.1 GCA_900108405.1, GCA_001050275.1, GCA_015461845.1, GCA_003264855.1, GCA_002087235.1 GCA_013140855.1, GCA_000010985.1, GCA_014841105.1, GCA_018139605.1, GCA_026344075.1 GCA_007858365.1, GCA_022898935.1, GCA_000154965.1, GCA_015262605.1, GCA_002128305.1 GCA_017753665.1, GCA_001513975.1, GCA_001886275.1, GCA_004770365.1, GCA_014202705.1 GCA_003253485.1, GCA_006716315.1, GCA_028553745.1, GCA_014202755.1, GCA_003269425.1 GCA_026626665.1, GCA_000963885.1, GCA_001885765.1, GCA_900445045.1, GCA_000741205.1 GCA_014358075.1, GCA_024807225.1, GCA_000348785.1, GCA_003725535.1, GCA_025961225.1 GCA_000785705.2, GCA_003258605.2, GCA_021650955.1, GCA_003004785.1, GCA_000708045.1 GCA_013177295.1, GCA_902703175.1, GCA_018137965.1, GCA_003254525.1, GCA_015245355.1 GCA_001692755.1, GCA_000060345.1, GCA_020079945.1, GCA_014117445.1, GCA_000974785.1 GCA_900107935.1, GCA_900116415.1, GCA_900142185.1, GCA_900104175.1, GCA_001688845.2 GCA_001761325.1, GCA_010287905.1, GCA_008704555.1, GCA_000364225.2, GCA_000949635.1 GCA_021029975.1, GCA_000950575.1, GCA_025567215.1, GCA_017876755.1, GCA_016458305.1 GCA_019575995.1, GCA_022870885.1, GCA_024137025.1, GCA_016862455.1, GCA_025061645.1 GCA_008802365.1, GCA_018972105.1, GCA_003096815.1, GCA_002211785.1, GCA_004135935.1 GCA_014054725.1, GCA_900157305.1, GCA_004570845.1, GCA_002217985.1, GCA_004785935.1 GCA_018599245.1, GCA_002374275.1, GCA_001431585.1, GCA_001687545.1, GCA_011170085.1 GCA_014205075.1, GCA_001682515.1, GCA_008705175.1, GCA_018145655.1, GCA_005233875.1 GCA_028981785.1, GCA_008386635.1, GCA_900188015.1, GCA_902153235.3, GCA_001467925.1 GCA_014646275.1, GCA_000019085.1, GCA_016725865.1, GCA_007341385.1, GCA_006152065.1 GCA_000178975.2, GCA_900102015.1, GCA_001941385.1, GCA_002811875.1, GCA_023806125.1 GCA_009857595.1, GCA_029277985.1, GCA_016056345.1, GCA_000190435.1, GCA_021245985.1 GCA_021049265.1, GCA_003992745.1, GCA_002591855.1, GCA_008727795.1, GCA_900107485.1 GCA_009695585.1, GCA_003026495.1, GCA_019316905.1, GCA_014982725.1, GCA_000965255.1 GCA_023156245.1, GCA_014172375.1, GCA_018905915.1, GCA_009828705.1, GCA_000019165.1 GCA_025631005.1, GCA_000786195.1, GCA_000724625.1, GCA_016861995.1, GCA_008017415.1 GCA_009913915.1, GCA_003350585.1, GCA_007988945.1, GCA_007859675.1, GCA_019443985.1 GCA_017874315.1, GCA_000016545.1, GCA_004723625.1, GCA_900129385.1, GCA_019703855.1 GCA_001853795.1, GCA_014636935.1, GCA_017354925.1, GCA_017255415.1, GCA_006363815.1 GCA_004345615.1, GCA_000190555.1, GCA_002285495.1, GCA_019973475.1, GCA_024128855.1 GCA_003367705.1, GCA_006740045.1, GCA_001747425.1, GCA_019603355.1, GCA_900101495.1 GCA_026427415.1, GCA_002259745.1, GCA_002844555.1, GCA_009295665.1, GCA_014198775.1 GCA_000299335.2, GCA_003003215.1, GCA_020809405.1, GCA_001205715.1, GCA_022846375.1 GCA_020034655.1, GCA_000252445.1, GCA_007993755.1, GCA_007829815.1, GCA_023516435.1 GCA_003014675.1, GCA_003966255.1, GCA_014692505.1, GCA_001402875.1, GCA_001434665.1 GCA_014191985.1, GCA_016599795.1, GCA_002943715.1, GCA_003667725.1, GCA_024125425.1 GCA_001682385.1, GCA_001940525.2, GCA_002841315.1, GCA_023016525.1, GCA_005780185.1 GCA_007988865.1, GCA_017591665.1, GCA_900103535.1, GCA_018531145.1, GCA_007989625.1 GCA_014641635.1, GCA_001192795.1, GCA_014652535.1, GCA_014652855.1, GCA_007997285.1 GCA_013820765.1, GCA_900094605.1, GCA_008086175.1, GCA_002843315.1, GCA_900115115.1 GCA_003074055.1, GCA_003950515.1, GCA_023656415.1, GCA_000226295.1, GCA_014196975.1 GCA_008728855.1, GCA_005048205.1, GCA_018704125.1, GCA_009939005.1, GCA_004117095.1 GCA_017901155.1, GCA_000007805.1, GCA_000024885.1, GCA_900489835.1, GCA_003014715.1 GCA_009688965.1, GCA_013201705.1, GCA_000023245.1, GCA_001652725.1, GCA_018141025.1 GCA_000012665.1, GCA_024198255.1, GCA_900115745.1, GCA_014651175.1, GCA_900141905.1 GCA_001028175.1, GCA_000018945.1, GCA_003364255.1, GCA_007991735.1, GCA_005780165.1 GCA_002381345.1, GCA_016756055.1, GCA_900106005.1, GCA_007992195.1, GCA_015277675.1 GCA_019613795.1, GCA_024198235.1, GCA_905221025.1, GCA_008014775.1, GCA_003590795.1 GCA_014493765.1, GCA_025917315.1, GCA_000230915.2, GCA_000498535.1, GCA_023284025.1 GCA_020216065.1, GCA_003966265.1, GCA_001709345.1, GCA_004217495.1, GCA_000982415.1 GCA_020037025.1, GCA_014947195.2, GCA_013315815.1, GCA_900110965.1, GCA_001431505.1 GCA_001477135.1, GCA_009451885.1, GCA_900108395.1, GCA_011516755.1, GCA_000020965.1 GCA_001632745.1, GCA_000233595.1, GCA_001885585.1, GCA_900112555.1, GCA_020149935.1 GCA_002995805.1, GCA_003367195.1, GCA_019711495.1, GCA_000178835.2, GCA_900618115.1 GCA_004524775.1, GCA_007860125.1, GCA_018917425.1, GCA_005862305.2, GCA_001043955.1 GCA_003201595.1, GCA_000211835.2, GCA_003957255.1, GCA_010729485.1, GCA_014191725.1 GCA_014836955.1, GCA_016728825.1, GCA_017976235.1, GCA_000300915.1, GCA_004341035.1 GCA_007004555.1, GCA_003633685.1, GCA_012910785.2, GCA_009735625.1, GCA_000733725.1 GCA_003261575.2, GCA_001816145.1, GCA_002284565.1, GCA_011927945.1, GCA_000730165.2 GCA_017873395.1, GCA_012033785.1, GCA_001854605.1, GCA_000731795.2, GCA_017874395.1 GCA_001688725.2, GCA_014203215.1, GCA_002934635.1, GCA_014858725.1, GCA_015352455.1 GCA_003611735.1, GCA_000299575.1, GCA_000967495.1, GCA_002871945.2, GCA_900142125.1 GCA_900102915.1, GCA_009695775.1, GCA_025215495.1, GCA_001308265.1, GCA_009730655.1 GCA_017873965.1, GCA_004123145.1, GCA_900101965.1, GCA_016613535.2, GCA_022899935.1 GCA_900141795.1, GCA_002354875.1, GCA_008933165.1, GCA_014836535.1, GCA_014295415.1 GCA_014637795.1, GCA_002160865.1, GCA_003752585.1, GCA_018326305.1, GCA_003260975.1 GCA_001573155.1, GCA_006385785.1, GCA_014174475.1, GCA_015751895.1, GCA_001940455.1 GCA_006716745.1, GCA_000835165.1, GCA_003265965.1, GCA_000241265.2, GCA_001636545.1 GCA_900142015.1, GCA_000956535.1, GCA_000975265.2, GCA_003386165.1, GCA_000019045.1 GCA_002849835.1, GCA_000444875.1, GCA_000959365.1, GCA_020683125.1, GCA_004348345.1 GCA_003953955.1, GCA_013694105.1, GCA_000583855.1, GCA_020278625.1, GCA_002802905.1 GCA_900111455.1, GCA_001044425.1, GCA_008932245.1, GCA_003385555.1, GCA_003987595.1 GCA_009828025.1, GCA_018332695.1, GCA_004339125.1, GCA_900089605.1, GCA_011761305.1 GCA_000723565.1, GCA_000264765.2, GCA_000006605.1, GCA_001597945.1, GCA_902459555.1 GCA_000355905.1, GCA_000940995.1, GCA_003172875.1, GCA_013363755.1, GCA_900102715.1 GCA_016605205.1, GCA_013347325.1, GCA_000284355.1, GCA_004785665.1, GCA_023920085.1 GCA_005280655.1, GCA_009856825.1, GCA_007997295.1, GCA_009734385.1, GCA_026183675.1 GCA_014230355.1, GCA_000471625.1, GCA_014653055.1, GCA_019355955.1, GCA_001438805.1 GCA_001412105.1, GCA_014203095.1, GCA_002834295.1, GCA_900458355.1, GCA_001514235.1 GCA_009913235.1, GCA_003044275.1, GCA_014207655.1, GCA_014645515.1, GCA_018494035.1 GCA_004684345.1, GCA_015689215.1, GCA_017868775.1, GCA_900302465.1, GCA_018406645.1 GCA_003173055.1, GCA_023195755.1, GCA_001465595.2, GCA_015627215.1, GCA_000204155.1 GCA_003665065.1, GCA_900103625.1, GCA_000192435.2, GCA_001567585.1, GCA_001405615.1 GCA_014192015.1, GCA_025914095.1, GCA_020616595.1, GCA_027922065.1, GCA_000970755.1 GCA_007859655.1, GCA_900111155.1, GCA_022230935.1, GCA_020523605.1, GCA_019711555.1 GCA_900187165.1, GCA_014191545.1, GCA_013137895.1, GCA_900167605.1, GCA_003006415.1 GCA_900112865.1, GCA_014656095.1, GCA_007625155.1, GCA_014062345.1, GCA_016820635.1 GCA_002934445.1, GCA_001507325.1, GCA_016605985.1, GCA_001633145.1, GCA_007747445.1 GCA_013004105.1, GCA_014771645.1, GCA_013347285.1, GCA_019042735.1, GCA_010723735.1 GCA_000217655.1, GCA_900110425.1, GCA_019263585.2, GCA_000218545.1, GCA_016863255.1 GCA_013280595.1, GCA_900105165.1, GCA_006968745.1, GCA_904848585.1, GCA_004331455.1 GCA_020510245.1, GCA_000258575.2, GCA_020735705.1, GCA_018437265.1, GCA_028737265.1 GCA_000802245.2, GCA_001687605.2, GCA_000468615.2, GCA_000259275.1, GCA_016765585.1 GCA_018069705.1, GCA_007991655.1, GCA_003798325.1, GCA_902459625.1, GCA_025558825.1 GCA_002251755.1, GCA_004363315.1, GCA_900156895.1, GCA_900116825.1, GCA_017874835.1 GCA_021312615.1, GCA_900091455.1, GCA_000166775.1, GCA_006546775.1, GCA_010211755.1 GCA_900100085.1, GCA_002162355.1, GCA_014621655.1, GCA_023330585.1, GCA_018881715.1 GCA_900240005.1, GCA_014635545.1, GCA_015209725.1, GCA_003388735.1, GCA_024756225.1 GCA_002095315.1, GCA_900660745.1, GCA_014202635.1, GCA_014652875.1, GCA_019428685.1 GCA_004681975.1, GCA_012911925.1, GCA_023922585.1, GCA_002007485.1, GCA_900100115.1 GCA_003050785.1, GCA_002209125.2, GCA_014645835.1, GCA_009834345.1, GCA_007995055.1 GCA_008704535.1, GCA_000742675.1, GCA_000160775.2, GCA_014696625.1, GCA_014651495.1 GCA_003096035.1, GCA_020164595.1, GCA_000092865.1, GCA_003470205.1, GCA_023283845.1 GCA_000952855.1, GCA_002006995.1, GCA_000319575.2, GCA_001753245.1, GCA_021172025.1 GCA_900450725.1, GCA_000243335.1, GCA_013385965.1, GCA_000576595.1, GCA_000157895.2 GCA_004683965.1, GCA_017873675.1, GCA_001050345.1, GCA_001441165.1, GCA_001021065.1 GCA_021183825.1, GCA_900142615.1, GCA_003054265.1, GCA_003989665.1, GCA_002217285.1 GCA_028751525.1, GCA_016862275.1, GCA_000350165.1, GCA_002959855.1, GCA_011927795.1 GCA_001046645.1, GCA_000092405.1, GCA_003001905.1, GCA_025154035.1, GCA_900167975.1 GCA_000152625.1, GCA_000626715.1, GCA_021531915.1, GCA_014270035.1, GCA_017876295.1 GCA_000024125.1, GCA_900101795.1, GCA_011303955.1, GCA_000328625.1, GCA_023702375.1 GCA_021295345.1, GCA_003046625.1, GCA_003254335.1, GCA_013096725.2, GCA_008124785.1 GCA_003315615.1, GCA_003143755.1, GCA_003148665.1, GCA_021390325.1, GCA_003987645.1 GCA_013403245.1, GCA_000737685.1, GCA_003030465.1, GCA_016632335.1, GCA_900095735.1 GCA_014648915.1, GCA_025961315.1, GCA_022024215.1, GCA_020144665.1, GCA_900142225.1 GCA_900188175.1, GCA_009208555.1, GCA_015163715.1, GCA_900110605.1, GCA_001601575.1 GCA_009711185.1, GCA_900103865.1, GCA_009914515.1, GCA_008327825.1, GCA_024224615.1 GCA_003096275.1, GCA_002271915.1, GCA_009627355.1, GCA_016127195.1, GCA_005116505.1 GCA_024281235.1, GCA_000820515.1, GCA_014650355.1, GCA_009831375.1, GCA_023703365.1 GCA_900111175.1, GCA_009755275.1, GCA_016907275.1, GCA_014268745.2, GCA_003345655.1 GCA_026547225.1, GCA_900107105.1, GCA_900167335.1, GCA_014203795.1, GCA_900129215.1 GCA_008124765.1, GCA_014202695.1, GCA_003065425.1, GCA_002093625.1, GCA_004340525.1 GCA_003987135.1, GCA_014332695.1, GCA_019429485.1, GCA_002043005.1, GCA_014644155.1 GCA_002993885.1, GCA_000685255.1, GCA_002954665.1, GCA_006094475.1, GCA_003574135.1 GCA_004353845.1, GCA_000013665.1, GCA_004011115.1, GCA_007845645.1, GCA_000022025.1 GCA_900115815.1, GCA_011761565.1, GCA_004216565.1, GCA_020532555.1, GCA_001968835.1 GCA_022592395.1, GCA_003298775.1, GCA_900106675.1, GCA_015025075.1, GCA_900129195.1 GCA_002220195.1, GCA_024171925.1, GCA_014678925.1, GCA_008802405.1, GCA_016757215.1 GCA_009827395.1, GCA_014218355.1, GCA_008830185.1, GCA_007992015.1, GCA_005403045.1 GCA_021166595.1, GCA_014137975.1, GCA_024171785.1, GCA_024380015.1, GCA_014873755.1 GCA_900176305.1, GCA_017876515.1, GCA_000407225.1, GCA_000740055.1, GCA_019458805.1 GCA_004331955.1, GCA_014490725.1, GCA_028553805.1, GCA_900113195.1, GCA_001558695.1 GCA_900116285.1, GCA_021590045.1, GCA_008039615.1, GCA_000497425.1, GCA_004803915.1 GCA_000173615.1, GCA_024054035.1, GCA_002843055.1, GCA_014202895.1, GCA_001436575.1 GCA_016751895.1, GCA_900168045.1, GCA_024195295.1, GCA_001437755.1, GCA_001439685.1 GCA_003144315.1, GCA_014779765.1, GCA_003591655.1, GCA_900452385.1, GCA_900099735.1 GCA_007748075.1, GCA_001997385.1, GCA_014653785.1, GCA_003991565.1, GCA_900454955.1 GCA_005697565.1, GCA_900176165.1, GCA_003353175.1, GCA_017313085.1, GCA_002177115.1 GCA_000018285.1, GCA_015644585.1, GCA_021545825.1, GCA_900141765.1, GCA_001441615.1 GCA_009380135.2, GCA_900156625.1, GCA_009601025.1, GCA_023715345.1, GCA_002209565.1 GCA_023913775.1, GCA_021172065.1, GCA_007830315.1, GCA_015689335.1, GCA_002760655.1 GCA_022568795.1, GCA_000699585.2, GCA_003315205.1, GCA_900100185.1, GCA_900156605.1 GCA_004134885.2, GCA_000176235.1, GCA_023614315.1, GCA_004291115.2, GCA_015244665.1 GCA_003606325.3, GCA_022786785.1, GCA_003444775.1, GCA_003590145.2, GCA_024701915.1 GCA_003268025.1, GCA_000340885.1, GCA_900101835.1, GCA_012516355.1, GCA_003751605.1 GCA_020147965.1, GCA_004102045.2, GCA_000284075.1, GCA_002367975.1, GCA_000494755.1 GCA_005771405.1, GCA_001634285.1, GCA_004011905.1, GCA_014644315.1, GCA_009497155.1 GCA_016889385.1, GCA_001720395.1, GCA_004217185.1, GCA_001047075.2, GCA_016725245.1 GCA_009659415.1, GCA_006547045.1, GCA_006265225.1, GCA_018138105.1, GCA_012849095.1 GCA_002368095.1, GCA_900107635.1, GCA_900091545.1, GCA_003515985.1, GCA_017565425.1 GCA_016725645.1, GCA_905220825.1, GCA_000019965.1, GCA_008041935.1, GCA_009695995.1 GCA_013371425.1, GCA_008831045.1, GCA_001999965.1, GCA_017303155.1, GCA_008710285.1 GCA_002811945.1, GCA_001642575.1, GCA_014642735.1, GCA_022376835.1, GCA_006575665.1 GCA_002210095.1, GCA_900188245.1, GCA_007828095.1, GCA_900177405.1, GCA_027570165.1 GCA_020866905.1, GCA_005473905.2, GCA_002000125.1, GCA_013409345.1, GCA_004367745.1 GCA_002355975.1, GCA_011304355.1, GCA_000935125.1, GCA_025564085.1, GCA_001761365.1 GCA_018256475.1, GCA_021310995.1, GCA_003259435.1, GCA_900099605.1, GCA_017353175.1 GCA_002029235.1, GCA_011762195.1, GCA_011742925.1, GCA_002902145.1, GCA_004217545.1 GCA_003590815.1, GCA_001485435.1, GCA_900637655.1, GCA_003931915.1, GCA_014333425.1 GCA_009827615.1, GCA_001984705.1, GCA_902860125.1, GCA_003991585.1, GCA_003713065.1 GCA_002018435.1, GCA_003024525.3, GCA_004745505.1, GCA_000317165.1, GCA_018333375.1 GCA_019740355.2, GCA_014836885.1, GCA_900102535.1, GCA_000092905.1, GCA_023717965.1 GCA_003236315.1, GCA_009910675.1, GCA_900129885.1, GCA_014145325.1, GCA_009932475.1 GCA_004769275.1, GCA_000353565.1, GCA_000020025.1, GCA_000469465.1, GCA_004565465.1 GCA_000953135.1, GCA_000770795.1, GCA_009753675.1, GCA_001544455.2, GCA_001639105.2 GCA_000243235.1, GCA_014639875.1, GCA_000161455.1, GCA_000305935.1, GCA_002939785.1 GCA_018274385.1, GCA_003113315.1, GCA_009711085.1, GCA_003367245.1, GCA_001263415.1 GCA_004028155.1, GCA_000829445.1, GCA_013127955.1, GCA_001438315.1, GCA_007995015.1 GCA_006337105.1, GCA_000224065.2, GCA_014642275.1, GCA_001887355.1, GCA_900167185.1 GCA_004362525.1, GCA_004421105.1, GCA_007745175.1, GCA_011761985.1, GCA_900103915.1 GCA_022760365.1, GCA_900116405.1, GCA_900112755.1, GCA_000262405.1, GCA_018459925.1 GCA_001514265.1, GCA_900129785.1, GCA_000023845.1, GCA_023921225.1, GCA_014202615.1 GCA_000404185.1, GCA_009707515.1, GCA_016056275.1, GCA_000160475.1, GCA_014198105.1 GCA_018595685.2, GCA_004309355.1, GCA_003732525.1, GCA_003428625.2, GCA_020531965.1 GCA_014699035.1, GCA_023973165.1, GCA_026168555.1, GCA_900112525.1, GCA_001302265.1 GCA_009767945.1, GCA_016481305.1, GCA_000236925.1, GCA_021168615.1, GCA_017876115.1 GCA_002930615.1, GCA_025152575.1, GCA_017742245.1, GCA_025567565.1, GCA_003815975.1 GCA_900155355.1, GCA_003626645.1, GCA_000367945.1, GCA_012275705.1, GCA_002287065.1 GCA_024171735.1, GCA_015209585.1, GCA_900101185.1, GCA_900100705.1, GCA_024648865.1 GCA_000242335.3, GCA_019645875.1, GCA_018728565.1, GCA_007197885.1, GCA_001676745.1 GCA_900637575.1, GCA_001742185.1, GCA_009857495.1, GCA_019968605.1, GCA_017114825.1 GCA_014192095.1, GCA_016904885.1, GCA_016772415.2, GCA_900475915.1, GCA_000007085.1 GCA_003610785.1, GCA_017313265.1, GCA_014201195.1, GCA_009371985.2, GCA_021129195.1 GCA_900112005.1, GCA_000331535.1, GCA_010731115.1, GCA_001586785.2, GCA_014156695.1 GCA_002841215.1, GCA_027922325.1, GCA_003815015.1, GCA_023371205.1, GCA_000235605.1 GCA_900109115.1, GCA_014203595.1, GCA_900114215.1, GCA_002078015.1, GCA_004339725.1 GCA_900478035.1, GCA_900141835.1, GCA_000021925.1, GCA_001644605.1, GCA_003351225.1 GCA_902806985.1, GCA_001050035.1, GCA_022827545.1, GCA_000830005.1, GCA_004363965.1 GCA_900188355.1, GCA_900102295.1, GCA_000174435.1, GCA_900638685.1, GCA_014385105.1 GCA_014174375.1, GCA_024349785.1, GCA_002849715.1, GCA_900128975.1, GCA_007989025.1 GCA_026735135.1, GCA_000737785.1, GCA_900637055.1, GCA_002023405.1, GCA_008831125.1 GCA_000179915.2, GCA_013155295.1, GCA_023221575.1, GCA_900102225.1, GCA_019203205.1 GCA_013283785.1, GCA_009720675.1, GCA_003314995.1, GCA_016587355.1, GCA_008806595.1 GCA_012035195.1, GCA_010728525.1, GCA_014905095.1, GCA_000144625.1, GCA_003149245.1 GCA_014199935.1, GCA_001431535.1, GCA_019145495.1, GCA_000281175.1, GCA_014647855.1 GCA_014197205.1, GCA_004634195.1, GCA_002896965.1, GCA_004331155.1, GCA_014646075.1 GCA_009498235.1, GCA_003710825.1, GCA_900156705.1, GCA_007752535.1, GCA_002018015.1 GCA_003544935.1, GCA_008124625.1, GCA_000757885.1, GCA_004797125.1, GCA_018139045.1 GCA_001708485.1, GCA_007989765.1, GCA_002224225.1, GCA_008107625.1, GCA_017313275.1 GCA_014042365.1, GCA_002265435.1, GCA_006265175.1, GCA_020731205.1, GCA_006717045.1 GCA_002135195.1, GCA_900101685.1, GCA_001889045.1, GCA_018829635.1, GCA_000143985.1 GCA_000021745.1, GCA_011058155.1, GCA_900101075.1, GCA_004403945.1, GCA_001043175.1 GCA_003675955.1, GCA_003697785.1, GCA_012584455.1, GCA_003990155.1, GCA_900566055.1 GCA_008016795.1, GCA_000210695.1, GCA_000512145.2, GCA_006385135.1, GCA_018398425.1 GCA_013371215.1, GCA_007859325.1, GCA_022012515.1, GCA_900111885.1, GCA_007785775.2 GCA_002008345.1, GCA_014650115.1, GCA_900107215.1, GCA_014287275.1, GCA_012926615.1 GCA_001509495.1, GCA_003148585.1, GCA_900637545.1, GCA_016863275.1, GCA_000165465.1 GCA_014205685.1, GCA_900113515.1, GCA_003096655.1, GCA_900182555.1, GCA_010731815.2 GCA_004353825.1, GCA_001037465.1, GCA_900128525.1, GCA_014652655.1, GCA_000973065.1 GCA_900142325.1, GCA_003254055.1, GCA_013340935.1, GCA_016863575.1, GCA_001889165.1 GCA_006335015.1, GCA_004349315.1, GCA_015142735.1, GCA_014651935.1, GCA_004362495.1 GCA_003311805.1, GCA_017573545.1, GCA_023008245.1, GCA_000829435.1, GCA_905120475.1 GCA_023283805.1, GCA_016617615.1, GCA_003584105.1, GCA_002206385.2, GCA_900090245.1 GCA_902459595.1, GCA_900107725.1, GCA_001434815.1, GCA_001904775.1, GCA_021173085.1 GCA_900460625.1, GCA_900187065.1, GCA_014201735.1, GCA_900099905.1, GCA_018122595.1 GCA_024181585.1, GCA_004365405.1, GCA_001310225.1, GCA_003751345.1, GCA_003316695.1 GCA_003570715.1, GCA_004340975.1, GCA_001412635.1, GCA_917563925.1, GCA_900177385.1 GCA_019207025.1, GCA_003966715.1, GCA_001434295.1, GCA_014466955.1, GCA_002300525.1 GCA_008933155.1, GCA_004016515.1, GCA_018555455.1, GCA_001483765.1, GCA_018390735.1 GCA_900167205.1, GCA_025996015.1, GCA_900638635.1, GCA_900096975.1, GCA_004358205.1 GCA_000019845.1, GCA_019731765.1, GCA_004004515.1, GCA_014873535.1, GCA_900142775.1 GCA_004803575.1, GCA_900106045.1, GCA_001984825.2, GCA_000016285.1, GCA_900108485.1 GCA_900101915.1, GCA_014384985.1, GCA_006459125.1, GCA_009036245.1, GCA_002101555.1 GCA_000233715.3, GCA_020071995.1, GCA_009687865.1, GCA_002887775.1, GCA_900100925.1 GCA_900109245.1, GCA_003664005.1, GCA_001617625.1, GCA_900167405.1, GCA_900638565.1 GCA_001908095.1, GCA_018403185.1, GCA_900105635.1, GCA_900090225.1, GCA_937468385.1 GCA_021234875.1, GCA_022606465.1, GCA_004362405.1, GCA_018101125.2, GCA_003258865.1 GCA_019331775.1, GCA_009649845.1, GCA_001708425.1, GCA_009695655.1, GCA_000284315.1 GCA_002917105.1, GCA_000689175.1, GCA_003721245.1, GCA_900115505.1, GCA_000242255.3 GCA_002335445.1, GCA_004168255.1, GCA_018406605.1, GCA_022637595.1, GCA_000394015.1 GCA_012396315.1, GCA_003015145.1, GCA_921294215.1, GCA_024172705.1, GCA_001551835.1 GCA_003990055.1, GCA_014203125.1, GCA_905220975.1, GCA_002920915.1, GCA_016028635.1 GCA_008056315.1, GCA_000092845.1, GCA_005577435.1, GCA_002843385.1, GCA_014649135.1 GCA_010918895.1, GCA_900108915.1, GCA_014206845.1, GCA_003475105.1, GCA_010993845.2 GCA_009769165.1, GCA_022559585.1, GCA_002005465.1, GCA_004331745.1, GCA_000214155.1 GCA_002217195.1, GCA_900188255.1, GCA_013201665.1, GCA_017874255.1, GCA_900142805.1 GCA_016027815.1, GCA_900112775.1, GCA_000230995.3, GCA_000768335.1, GCA_000723585.1 GCA_002900385.1, GCA_006335005.1, GCA_000980835.1, GCA_900143265.1, GCA_900112225.1 GCA_900458665.1, GCA_023499275.1, GCA_018449405.1, GCA_000344785.1, GCA_001553405.1 GCA_002813445.1, GCA_009668025.1, GCA_900111015.1, GCA_900187215.1, GCA_016908635.1 GCA_009671205.1, GCA_012272835.1, GCA_007828955.1, GCA_020310025.1, GCA_000829395.1 GCA_018408575.1, GCA_014635285.1, GCA_004570865.1, GCA_002007565.1, GCA_002259585.1 GCA_021147765.1, GCA_014205055.1, GCA_900112675.1, GCA_023923245.1, GCA_018476645.1 GCA_014217995.1, GCA_007830055.1, GCA_003944765.1, GCA_020387775.1, GCA_014656415.1 GCA_900111305.1, GCA_000632475.2, GCA_900108275.1, GCA_000445475.1, GCA_900115475.1 GCA_014050225.1, GCA_003054475.1, GCA_000020985.1, GCA_009728935.1, GCA_900188275.1 GCA_014650775.1, GCA_019029495.1, GCA_020546685.1, GCA_001747085.1, GCA_002993335.1 GCA_923081025.1, GCA_001458555.1, GCA_016909075.1, GCA_027622945.1, GCA_009600885.1 GCA_001897425.1, GCA_013378445.1, GCA_025562755.1, GCA_024172185.1, GCA_014218275.1 GCA_004307415.1, GCA_014193375.1, GCA_014647675.1, GCA_021295095.1, GCA_001436135.1 GCA_001020985.1, GCA_002021565.1, GCA_900129725.1, GCA_003185915.1, GCA_001653075.1 GCA_014884965.1, GCA_014644535.1, GCA_000931935.2, GCA_025264685.1, GCA_003182375.1 GCA_900102265.1, GCA_000758765.1, GCA_009914535.1, GCA_000371485.1, GCA_003967555.1 GCA_001659725.1, GCA_000287335.1, GCA_000418365.1, GCA_014638005.1, GCA_003254505.1 GCA_003259445.1, GCA_002441655.1, GCA_003945345.1, GCA_013423485.1, GCA_018224885.1 GCA_014646115.1, GCA_000280055.1, GCA_900102685.1, GCA_900106025.1, GCA_008630535.1 GCA_000585215.1, GCA_900460255.1, GCA_024259515.1, GCA_016724785.1, GCA_020422925.2 GCA_900446125.1, GCA_002849875.1, GCA_017498085.1, GCA_901544385.1, GCA_002285635.2 GCA_004217045.1, GCA_004684935.1, GCA_910593785.1, GCA_001742305.1, GCA_900460335.1 GCA_019139535.1, GCA_011304195.1, GCA_009380165.1, GCA_004564375.1, GCA_013753875.1 GCA_001043975.1, GCA_014836405.1, GCA_023333585.1, GCA_018966525.1, GCA_017498525.1 GCA_014199335.1, GCA_003429665.1, GCA_013366805.1, GCA_018408705.1, GCA_004366575.1 GCA_001437605.1, GCA_017676385.1, GCA_000769915.1, GCA_018128025.1, GCA_002327145.1 GCA_000174415.1, GCA_001884235.1, GCA_000622425.1, GCA_001319845.1, GCA_001189295.1 GCA_018137985.1, GCA_008704395.1, GCA_900066015.1, GCA_004000605.1, GCA_008710165.1 GCA_004295685.1, GCA_016653315.1, GCA_014725695.1, GCA_003546865.1, GCA_002277955.1 GCA_900111495.1, GCA_900185565.1, GCA_000344135.1, GCA_027921865.1, GCA_017161365.1 GCA_002980575.1, GCA_003148385.1, GCA_013177675.1, GCA_012927305.1, GCA_014652575.1 GCA_001730235.1, GCA_019047765.1, GCA_900637105.1, GCA_002871895.1, GCA_002632595.1 GCA_011250645.1, GCA_019039105.1, GCA_900105765.1, GCA_003308995.1, GCA_003346775.1 GCA_005234135.1, GCA_016123485.1, GCA_016623615.1, GCA_003325375.1, GCA_020531905.1 GCA_000755585.2, GCA_000346485.2, GCA_014205635.1, GCA_001436505.1, GCA_014323725.1 GCA_008693965.1, GCA_009496975.1, GCA_014054885.1, GCA_009569385.1, GCA_022179445.1 GCA_010550675.1, GCA_004364865.1, GCA_900452695.1, GCA_003265305.2, GCA_000821325.2 GCA_000972785.3, GCA_001921205.1, GCA_004122115.1, GCA_001543285.1, GCA_003002065.1 GCA_003003695.1, GCA_026343715.1, GCA_003605475.1, GCA_017304535.1, GCA_008370715.1 GCA_900129495.1, GCA_012927405.1, GCA_025999835.1, GCA_900111805.1, GCA_022869165.1 GCA_014397785.1, GCA_900091585.1, GCA_900478165.1, GCA_015831355.1, GCA_008086165.1 GCA_022637515.1, GCA_000021905.1, GCA_014697125.1, GCA_014836445.1, GCA_000015345.1 GCA_004028235.1, GCA_010731895.1, GCA_014653155.1, GCA_016583575.1, GCA_001865995.1 GCA_011516935.1, GCA_000833025.1, GCA_016595495.1, GCA_900094965.1, GCA_014649015.1 GCA_003011885.1, GCA_001437855.1, GCA_900109285.1, GCA_018406465.1, GCA_000559085.2 GCA_004114955.1, GCA_000829515.1, GCA_000017305.1, GCA_025821625.1, GCA_007991215.1 GCA_014652335.1, GCA_000585335.1, GCA_006542685.1, GCA_007748035.1, GCA_004135605.1 GCA_014640195.1, GCA_001438655.1, GCA_900215295.1, GCA_023238245.1, GCA_019056575.1 GCA_004342665.1, GCA_019355975.1, GCA_014195535.1, GCA_000013765.1, GCA_002885975.1 GCA_017872935.1, GCA_003634345.1, GCA_014837055.1, GCA_000019945.1, GCA_014640075.1 GCA_900111745.1, GCA_003668565.1, GCA_009377175.1, GCA_900113375.1, GCA_001989575.1 GCA_018139645.1, GCA_014649655.1, GCA_001281425.1, GCA_026154845.1, GCA_008086115.1 GCA_015644725.1, GCA_000007985.2, GCA_900458545.1, GCA_017353185.1, GCA_001458235.1 GCA_002933875.1, GCA_000487995.2, GCA_004331345.1, GCA_900142005.1, GCA_003863355.1 GCA_003589925.1, GCA_018129065.1, GCA_902860075.1, GCA_013177635.1, GCA_019598945.1 GCA_003633615.1, GCA_000244975.1, GCA_003602235.1, GCA_013249015.1, GCA_014203955.1 GCA_000966975.1, GCA_008298015.1, GCA_026228885.1, GCA_900103475.1, GCA_014649735.1 GCA_000092245.1, GCA_019399205.1, GCA_900107285.1, GCA_015472005.1, GCA_001458295.1 GCA_019395145.1, GCA_009388985.1, GCA_005048265.1, GCA_028981925.1, GCA_000231055.3 GCA_025145565.1, GCA_003586265.1, GCA_002234595.1, GCA_000612035.1, GCA_007746795.1 GCA_006516965.1, GCA_008107645.1, GCA_900450955.1, GCA_021474425.1, GCA_000210095.1 GCA_014196915.1, GCA_003025555.1, GCA_014647895.1, GCA_000170755.1, GCA_015097055.1 GCA_003987355.1, GCA_012275535.1, GCA_003432485.1, GCA_016862995.1, GCA_011040435.1 GCA_003026475.1, GCA_010667655.1, GCA_015163655.1, GCA_900217755.1, GCA_001648155.1 GCA_016306625.1, GCA_900112485.1, GCA_010119385.1, GCA_012871055.1, GCA_902459585.1 GCA_004329705.1, GCA_900110435.1, GCA_014656195.1, GCA_011068405.5, GCA_003289945.1 GCA_900099765.1, GCA_018135955.1, GCA_005877905.1, GCA_003664645.1, GCA_000298295.1 GCA_014646675.1, GCA_004770555.1, GCA_016741855.1, GCA_014705615.1, GCA_900167935.1 GCA_022836935.1, GCA_013283665.1, GCA_001886695.1, GCA_001499925.1, GCA_900143235.1 GCA_011927725.1, GCA_023333595.1, GCA_014201875.1, GCA_022601735.1, GCA_016599835.1 GCA_003865035.1, GCA_014648815.1, GCA_000145235.1, GCA_028609845.1, GCA_003994475.1 GCA_006864425.1, GCA_004919095.1, GCA_010723515.1, GCA_008365315.1, GCA_001544795.2 GCA_002531755.2, GCA_004122035.1, GCA_003367475.1, GCA_000169255.2, GCA_014672715.2 GCA_900682675.2, GCA_007998985.1, GCA_000612055.1, GCA_000300855.1, GCA_003633895.1 GCA_002864915.1, GCA_900110855.1, GCA_000176375.1, GCA_001590835.1, GCA_000008465.1 GCA_900187055.1, GCA_014197135.1, GCA_003797945.1, GCA_001263205.1, GCA_000447205.1 GCA_000828085.3, GCA_021183565.1, GCA_014268785.2, GCA_003122325.1, GCA_001517585.1 GCA_900112035.1, GCA_008244765.1, GCA_001267255.1, GCA_014197915.1, GCA_014656135.1 GCA_018140635.1, GCA_900090105.1, GCA_000158175.1, GCA_023283895.1, GCA_000179015.2 GCA_004357995.1, GCA_008195825.1, GCA_007827425.1, GCA_000368585.1, GCA_000804505.1 GCA_002270055.1, GCA_004770155.1, GCA_900094835.1, GCA_009911795.1, GCA_002786325.1 GCA_019097855.1, GCA_011605535.1, GCA_012295595.1, GCA_002252445.1, GCA_014049385.1 GCA_003044065.1, GCA_001721685.1, GCA_900099615.1, GCA_016906065.1, GCA_003402975.1 GCA_014643535.1, GCA_001606025.1, GCA_019457885.1, GCA_024746855.1, GCA_900258035.1 GCA_023715605.1, GCA_005943945.1, GCA_001437905.1, GCA_000316195.1, GCA_013408745.1 GCA_005889715.1, GCA_001687475.2, GCA_020889625.1, GCA_004137255.1, GCA_022179765.1 GCA_000176855.2, GCA_016128195.1, GCA_014206975.1, GCA_000981765.1, GCA_003813905.1 GCA_900108565.1, GCA_007992695.1, GCA_004217455.1, GCA_002886045.1, GCA_016027415.1 GCA_007954605.1, GCA_001476135.1, GCA_002356035.1, GCA_003935525.1, GCA_014196055.1 GCA_900015005.1, GCA_001687335.1, GCA_900637295.1, GCA_002343915.1, GCA_009664085.1 GCA_900113635.1, GCA_004912165.1, GCA_005116445.1, GCA_012641725.1, GCA_001686925.1 GCA_900637195.1, GCA_900184935.1, GCA_900156225.1, GCA_003339815.1, GCA_003931795.1 GCA_003610805.1, GCA_004005905.1, GCA_900100655.1, GCA_003097535.1, GCA_018403685.1 GCA_003010965.1, GCA_011927635.1, GCA_900289045.1, GCA_900129115.1, GCA_015221995.1 GCA_000661955.1, GCA_002005165.1, GCA_023721795.1, GCA_000194115.1, GCA_009857745.1 GCA_012033735.1, GCA_016888445.1, GCA_004770145.1, GCA_900107465.1, GCA_004349265.1 GCA_011516825.1, GCA_000227665.3, GCA_010078235.1, GCA_023715205.1, GCA_900115855.1 GCA_002934265.1, GCA_900114705.1, GCA_014651515.1, GCA_000022265.1, GCA_001657475.1 GCA_016734805.1, GCA_003867075.1, GCA_003862485.1, GCA_000488275.1, GCA_003172975.1 GCA_026013905.1, GCA_016907875.1, GCA_004004565.1, GCA_015356115.1, GCA_014048465.1 GCA_013402795.1, GCA_900167325.1, GCA_001952065.1, GCA_000317475.1, GCA_022230925.1 GCA_009811755.1, GCA_900103795.1, GCA_017352215.1, GCA_000253035.1, GCA_902459685.1 GCA_022179725.1, GCA_003570705.1, GCA_016907775.1, GCA_001675165.1, GCA_001562425.1 GCA_902499105.1, GCA_009295725.1, GCA_004564325.1, GCA_025558845.1, GCA_009183815.1 GCA_003024475.1, GCA_020166395.1, GCA_000504245.1, GCA_900101615.1, GCA_000062885.1 GCA_014192055.1, GCA_017875235.1, GCA_016862575.1, GCA_003987045.1, GCA_018128325.1 GCA_003459085.1, GCA_024460155.1, GCA_002797675.1, GCA_900108005.1, GCA_014107475.1 GCA_018917865.1, GCA_004363725.1, GCA_025515205.1, GCA_014873765.1, GCA_016907055.1 GCA_019780885.1, GCA_004343155.1, GCA_004349105.1, GCA_024199905.1, GCA_016696765.1 GCA_014648275.1, GCA_022394675.1, GCA_009711415.1, GCA_017872975.1, GCA_018129005.1 GCA_000706765.1, GCA_002915595.1, GCA_019139835.1, GCA_900129355.1, GCA_014199265.1 GCA_004296335.1, GCA_016888335.1, GCA_001545155.1, GCA_900100955.1, GCA_900115085.1 GCA_011761185.1, GCA_001274775.1, GCA_014640975.1, GCA_002263515.1, GCA_900129845.1 GCA_008806325.1, GCA_009002475.1, GCA_004365595.1, GCA_011044475.1, GCA_009649915.1 GCA_001586235.1, GCA_000754805.1, GCA_005117025.1, GCA_009857835.1, GCA_001275135.1 GCA_012911815.1, GCA_003148525.1, GCA_005843925.1, GCA_019552185.1, GCA_014803385.1 GCA_015904235.1, GCA_025660375.1, GCA_003002095.1, GCA_014199985.1, GCA_002216815.1 GCA_002233675.1, GCA_013283795.1, GCA_000024825.1, GCA_027213965.1, GCA_014218705.1 GCA_009380155.1, GCA_006539205.1, GCA_014903745.1, GCA_013410645.1, GCA_900107135.1 GCA_000773975.1, GCA_014763045.1, GCA_016908595.1, GCA_002027205.1, GCA_014191875.1 GCA_008727925.1, GCA_013368775.1, GCA_001586255.1, GCA_013177355.1, GCA_900105945.1 GCA_014138605.1, GCA_009428965.1, GCA_009730055.1, GCA_000511355.1, GCA_014189415.1 GCA_000024985.1, GCA_000185385.1, GCA_009882985.1, GCA_900105585.1, GCA_013410345.1 GCA_018437235.1, GCA_000023825.1, GCA_009371875.1, GCA_000196095.1, GCA_000775615.1 GCA_000025125.1, GCA_002407065.1, GCA_900113695.1, GCA_003751265.1, GCA_014655715.1 GCA_900113065.1, GCA_017357445.1, GCA_004342425.1, GCA_001908025.1, GCA_003858535.1 GCA_003390675.1, GCA_002367995.1, GCA_900089985.1, GCA_000196515.1, GCA_002860345.1 GCA_014191715.1, GCA_000968195.1, GCA_004358695.1, GCA_014023125.1, GCA_004005965.1 GCA_000413935.1, GCA_003051055.1, GCA_900445945.1, GCA_000834395.1, GCA_008370495.1 GCA_004214835.1, GCA_003029735.1, GCA_001641695.1, GCA_001281385.1, GCA_003236175.1 GCA_022870845.1, GCA_002895945.1, GCA_003325355.1, GCA_004362375.1, GCA_014647735.1 GCA_000600975.2, GCA_900142565.1, GCA_025266575.1, GCA_003097635.1, GCA_004346105.1 GCA_003206055.1, GCA_003148745.1, GCA_014651475.1, GCA_019443125.1, GCA_013408555.1 GCA_902497555.1, GCA_002355335.1, GCA_000195315.1, GCA_007991635.1, GCA_001895265.1 GCA_025566885.1, GCA_900187075.1, GCA_002937075.1, GCA_003008575.1, GCA_000517385.1 GCA_000234355.2, GCA_006159205.1, GCA_005280315.1, GCA_900103615.1, GCA_900128885.1 GCA_002214625.2, GCA_018413645.1, GCA_002102025.1, GCA_003626545.1, GCA_016901015.1 GCA_011090165.1, GCA_003076515.1, GCA_000154725.1, GCA_004369225.1, GCA_014650035.1 GCA_004793925.1, GCA_017161445.1, GCA_013149805.1, GCA_023078355.1, GCA_003550065.1 GCA_015137655.1, GCA_000177235.2, GCA_028982115.1, GCA_008271695.1, GCA_018243215.1 GCA_000772265.1, GCA_000689395.1, GCA_012913485.1, GCA_900156495.1, GCA_025152405.1 GCA_900091435.1, GCA_006265115.1, GCA_022834715.1, GCA_019203185.1, GCA_010820555.1 GCA_005771525.1, GCA_000736675.1, GCA_020268605.1, GCA_004138325.1, GCA_012295615.1 GCA_001461805.1, GCA_016862835.1, GCA_019754945.1, GCA_027921985.1, GCA_014946725.1 GCA_014396585.1, GCA_900186985.1, GCA_017874735.1, GCA_004363195.1, GCA_016595465.1 GCA_002843065.1, GCA_014645555.1, GCA_020024005.1, GCA_008571395.1, GCA_003063245.1 GCA_008806775.1, GCA_003633955.1, GCA_025151995.1, GCA_000296815.2, GCA_019795155.1 GCA_027922345.1, GCA_000091565.1, GCA_001941625.1, GCA_900099705.1, GCA_019042245.1 GCA_006716345.1, GCA_003635025.1, GCA_025562715.2, GCA_900168325.1, GCA_009755645.1 GCA_900168025.1, GCA_014199615.1, GCA_000277165.1, GCA_003002055.1, GCA_900113745.1 GCA_009857845.1, GCA_004114535.1, GCA_014274465.1, GCA_011516885.1, GCA_008868685.1 GCA_001643775.1, GCA_000342165.1, GCA_000020465.1, GCA_004341685.1, GCA_000820845.2 GCA_009299385.1, GCA_003332885.2, GCA_019711615.1, GCA_016552165.1, GCA_024172165.1 GCA_016612995.1, GCA_022968395.1, GCA_004798725.1, GCA_022430545.2, GCA_003696265.1 GCA_023646305.1, GCA_900099985.1, GCA_001507645.1, GCA_900101785.1, GCA_004784475.1 GCA_003014655.1, GCA_001662875.1, GCA_009498275.1, GCA_007859865.1, GCA_003602275.1 GCA_900167055.1, GCA_001866515.1, GCA_017052655.1, GCA_009933595.1, GCA_002286915.1 GCA_021648825.1, GCA_018343695.1, GCA_018316795.1, GCA_004342325.1, GCA_002355595.1 GCA_026805325.1, GCA_001431465.1, GCA_019429535.1, GCA_000153705.1, GCA_012933505.1 GCA_012225885.1, GCA_008710135.1, GCA_016809835.2, GCA_001028705.1, GCA_002532075.1 GCA_902143385.2, GCA_004216555.1, GCA_000818015.1, GCA_002795825.1, GCA_001414055.1 GCA_900156155.1, GCA_000445065.1, GCA_016765795.1, GCA_009674665.1, GCA_006376675.1 GCA_014841115.1, GCA_000017265.1, GCA_017876495.1, GCA_004341395.1, GCA_008704575.1 GCA_003626535.1, GCA_008151785.1, GCA_003938655.1, GCA_009882975.1, GCA_001563495.1 GCA_003627055.1, GCA_014652435.1, GCA_007004665.1, GCA_009711345.1, GCA_001886595.1 GCA_000255135.1, GCA_007750395.1, GCA_003219815.1, GCA_001642655.1, GCA_004054195.1 GCA_007741515.1, GCA_900232995.1, GCA_000196475.1, GCA_009857445.1, GCA_900187285.1 GCA_008254045.1, GCA_003795145.1, GCA_003634585.1, GCA_014634985.1, GCA_003002955.1 GCA_000025005.1, GCA_900143695.1, GCA_000297395.2, GCA_023714025.1, GCA_002795405.1 GCA_003693265.1, GCA_002869505.1, GCA_001553565.1, GCA_001747405.1, GCA_014322795.1 GCA_000008625.1, GCA_000403135.1, GCA_007559155.1, GCA_011927785.1, GCA_019704135.1 GCA_014836745.1, GCA_016316925.1, GCA_023838525.1, GCA_024023655.1, GCA_003815875.1 GCA_014748335.1, GCA_000196215.1, GCA_900176365.1, GCA_014898155.1, GCA_013410455.1 GCA_001620305.1, GCA_003122215.1, GCA_005221285.1, GCA_014189455.1, GCA_016908395.1 GCA_003054225.1, GCA_014648075.1, GCA_001436605.1, GCA_027622875.1, GCA_014642915.1 GCA_001046855.1, GCA_900172335.1, GCA_025311515.1, GCA_004349235.1, GCA_900185625.1 GCA_025567065.1, GCA_000300235.2, GCA_014138835.1, GCA_004121315.1, GCA_000024785.1 GCA_900141825.1, GCA_004022565.1, GCA_006715175.1, GCA_026901265.1, GCA_900453045.1 GCA_003606265.1, GCA_002077695.1, GCA_001507385.1, GCA_000176915.2, GCA_014874065.1 GCA_003812285.1, GCA_900176515.1, GCA_016863535.1, GCA_001436595.1, GCA_014048475.1 GCA_900172305.1, GCA_016894445.1, GCA_003550015.1, GCA_900070205.1, GCA_014699005.1 GCA_002355855.1, GCA_022271405.1, GCA_014636175.1, GCA_900115615.1, GCA_021609905.1 GCA_004025965.2, GCA_017565705.1, GCA_020423125.1, GCA_016902245.1, GCA_004216715.1 GCA_021083375.1, GCA_900104905.1, GCA_020150375.1, GCA_021026095.1, GCA_003990915.1 GCA_900099975.1, GCA_002102175.1, GCA_009662475.1, GCA_019753795.1, GCA_900100665.1 GCA_025566865.1, GCA_900302495.1, GCA_001723305.1, GCA_000317495.1, GCA_000512915.1 GCA_002157895.1, GCA_004525745.1, GCA_023653015.1, GCA_907165195.1, GCA_010093135.1 GCA_003664035.1, GCA_004153455.1, GCA_006337085.1, GCA_900129805.1, GCA_014297595.1 GCA_004011175.1, GCA_012516475.1, GCA_009711525.1, GCA_000246755.1, GCA_016583795.1 GCA_900110585.1, GCA_028607105.1, GCA_009696275.1, GCA_003610695.1, GCA_003590855.1 GCA_018491655.1, GCA_014199595.1, GCA_000177215.2, GCA_900129305.1, GCA_001190945.1 GCA_016026615.1, GCA_014701235.1, GCA_009711445.1, GCA_009913655.1, GCA_026339705.1 GCA_009835145.1, GCA_004402695.1, GCA_003337375.1, GCA_003641185.1, GCA_900141885.1 GCA_900445655.1, GCA_002080475.1, GCA_021568905.1, GCA_004355005.1, GCA_002940015.1 GCA_015207255.1, GCA_014836495.1, GCA_900239945.1, GCA_900142395.1, GCA_005890135.1 GCA_009707125.1, GCA_004564075.1, GCA_001708935.1, GCA_001420695.1, GCA_004358165.1 GCA_023155215.1, GCA_900091565.1, GCA_023525255.1, GCA_900105885.1, GCA_900104705.1 GCA_900142895.1, GCA_000261045.2, GCA_014653575.1, GCA_004362855.1, GCA_000734965.1 GCA_001618175.1, GCA_000280865.2, GCA_000831385.1, GCA_010233585.1, GCA_004364175.1 GCA_026344175.1, GCA_002355435.1, GCA_018257325.1, GCA_001982615.1, GCA_013778325.1 GCA_014623485.1, GCA_006364355.1, GCA_001483945.1, GCA_000253015.1, GCA_900105655.1 GCA_900177645.1, GCA_017874545.1, GCA_905397275.1, GCA_900103445.1, GCA_900105255.1 GCA_900109085.1, GCA_900660755.1, GCA_900142275.1, GCA_021272345.1, GCA_918814575.1 GCA_011300215.1, GCA_900172235.1, GCA_003014475.1, GCA_019396925.1, GCA_017354965.1 GCA_000170835.1, GCA_003352005.1, GCA_014203115.1, GCA_900172325.1, GCA_003076755.1 GCA_014646815.1, GCA_019670705.1, GCA_007992495.1, GCA_016907715.1, GCA_011045125.1 GCA_003687175.1, GCA_004340985.1, GCA_900450765.1, GCA_001999305.1, GCA_003114855.1 GCA_003234965.1, GCA_000987775.1, GCA_003335045.1, GCA_900091515.1, GCA_014207595.1 GCA_000832545.1, GCA_900108585.1, GCA_011492945.1, GCA_014651355.1, GCA_002051585.1 GCA_003852895.1, GCA_003217255.1, GCA_009720745.1, GCA_007991315.1, GCA_003885105.1 GCA_007625215.1, GCA_014636295.1, GCA_002163605.1, GCA_002844535.1, GCA_014202905.1 GCA_014284305.1, GCA_001580615.1, GCA_018324205.1, GCA_000309885.1, GCA_016894325.1 GCA_003318295.1, GCA_004116385.1, GCA_003719725.1, GCA_004402015.1, GCA_900167565.1 GCA_000961215.1, GCA_028473685.1, GCA_002993365.1, GCA_014197245.1, GCA_014197445.1 GCA_900417275.1, GCA_004366635.1, GCA_010811875.1, GCA_009877435.1, GCA_016772275.1 GCA_003627135.1, GCA_002393445.1, GCA_000017685.1, GCA_003609595.1, GCA_009674565.1 GCA_017368815.1, GCA_014635825.1, GCA_004363555.1, GCA_001854655.1, GCA_012911935.1 GCA_900447205.1, GCA_009811635.1, GCA_016900875.1, GCA_014200235.1, GCA_003382565.3 GCA_014230315.1, GCA_012910805.1, GCA_025758635.1, GCA_005771675.1, GCA_002080395.1 GCA_014196745.1, GCA_003865365.1, GCA_012516395.1, GCA_923079645.1, GCA_017368795.1 GCA_004363805.1, GCA_009901565.1, GCA_003634695.1, GCA_016741935.1, GCA_000019205.1 GCA_003025455.1, GCA_900129275.1, GCA_003600625.1, GCA_000154385.1, GCA_003957515.1 GCA_003953935.1, GCA_003284895.1, GCA_003058465.1, GCA_000237305.1, GCA_020923495.1 GCA_001750725.1, GCA_002858715.1, GCA_018685455.1, GCA_018389405.1, GCA_015680405.1 GCA_017254835.1, GCA_003595305.1, GCA_004151275.1, GCA_014352875.1, GCA_009873295.1 GCA_002356555.2, GCA_016862795.1, GCA_019669905.1, GCA_001887245.1, GCA_004341165.1 GCA_019933235.1, GCA_900129075.1, GCA_001314325.1, GCA_003148935.1, GCA_000009305.1 GCA_001906925.1, GCA_016908655.1, GCA_001267435.1, GCA_014647175.1, GCA_011397115.1 GCA_014640455.1, GCA_900107035.1, GCA_022606295.1, GCA_000243395.3, GCA_022899355.1 GCA_017873435.1, GCA_003932995.1, GCA_000014965.1, GCA_002076915.1, GCA_014649695.1 GCA_900142365.1, GCA_900055185.1, GCA_014385025.1, GCA_004683865.2, GCA_900102435.1 GCA_004348195.1, GCA_020971565.1, GCA_900107435.1, GCA_004358035.1, GCA_900142625.1 GCA_009937995.1, GCA_005116715.1, GCA_001596815.1, GCA_000294655.1, GCA_003268275.1 GCA_003335215.1, GCA_016900315.1, GCA_000331735.1, GCA_000025945.1, GCA_017815575.1 GCA_002251735.1, GCA_024622045.1, GCA_009909615.1, GCA_000253375.1, GCA_012271785.1 GCA_001412085.1, GCA_003363715.1, GCA_900114045.1, GCA_918378365.1, GCA_900105535.1 GCA_007989385.1, GCA_004770425.1, GCA_000963705.1, GCA_014636315.1, GCA_014642795.1 GCA_900079135.1, GCA_016863555.1, GCA_023701865.1, GCA_006546835.1, GCA_024518835.1 GCA_003096175.1, GCA_003579925.1, GCA_013315265.1, GCA_003025615.1, GCA_016909155.1 GCA_000349205.1, GCA_013267415.1, GCA_900129035.1, GCA_014638275.1, GCA_012979535.1 GCA_013415125.1, GCA_019711415.1, GCA_900114205.1, GCA_011926545.1, GCA_014647655.1 GCA_003628125.1, GCA_000006765.1, GCA_900289195.1, GCA_022953075.1, GCA_009928685.1 GCA_014647435.1, GCA_001020955.1, GCA_014645075.1, GCA_900142055.1, GCA_021654855.1 GCA_000626675.1, GCA_007997015.1, GCA_003074995.2, GCA_003586285.1, GCA_001544515.2 GCA_018130825.1, GCA_900115905.1, GCA_014650255.1, GCA_012034335.1, GCA_016806125.1 GCA_003204205.1, GCA_014138635.1, GCA_000154525.1, GCA_900129015.1, GCA_000166135.1 GCA_003325455.1, GCA_008245125.1, GCA_000315235.1, GCA_026410485.1, GCA_003627075.1 GCA_000024025.1, GCA_010730575.1, GCA_900182655.1, GCA_003634755.1, GCA_007997385.1 GCA_024205945.1, GCA_000473895.1, GCA_000948975.2, GCA_014645695.1, GCA_002007645.1 GCA_014646215.1, GCA_900458255.1, GCA_002849855.1, GCA_900142135.1, GCA_009828115.1 GCA_016862955.1, GCA_015704865.1, GCA_020026925.1, GCA_000215705.1, GCA_022352215.1 GCA_900231835.1, GCA_002802875.1, GCA_004216535.1, GCA_003435335.1, GCA_014489635.1 GCA_001187845.1, GCA_014837145.1, GCA_900455725.1, GCA_000767465.1, GCA_900184895.1 GCA_000156355.1, GCA_005166025.1, GCA_014651615.1, GCA_024159215.1, GCA_014650975.1 GCA_014643255.1, GCA_000696185.1, GCA_002115805.1, GCA_011611525.1, GCA_002797735.1 GCA_003429565.1, GCA_012647205.1, GCA_000214235.1, GCA_009827535.1, GCA_010747415.1 GCA_002441885.1, GCA_900102525.1, GCA_000024965.1, GCA_000725125.1, GCA_018292125.1 GCA_001038625.1, GCA_009720625.1, GCA_013415485.1, GCA_004118675.1, GCA_016583765.1 GCA_003611695.1, GCA_007743815.1, GCA_014635265.1, GCA_900129975.1, GCA_014395785.1 GCA_021043395.1, GCA_000170955.2, GCA_014652835.1, GCA_003094615.1, GCA_007989465.1 GCA_003729955.1, GCA_024753205.1, GCA_009674725.1, GCA_004342065.1, GCA_004770265.1 GCA_001721295.1, GCA_000178455.1, GCA_014648295.1, GCA_002738225.1, GCA_003202035.1 GCA_003003275.1, GCA_003627155.1, GCA_001434395.1, GCA_004348335.1, GCA_016862755.1 GCA_900005615.1, GCA_003438245.1, GCA_003244875.1, GCA_013403565.1, GCA_004551525.1 GCA_019916025.1, GCA_000364285.1, GCA_000682695.1, GCA_003573545.1, GCA_006716765.1 GCA_023347705.1, GCA_902459485.1, GCA_900167145.1, GCA_022230905.1, GCA_002499975.2 GCA_025567415.1, GCA_001675355.1, GCA_011683915.1, GCA_014696435.1, GCA_000195275.1 GCA_014200395.1, GCA_022811665.1, GCA_900101435.1, GCA_001888925.1, GCA_009769755.1 GCA_006540005.1, GCA_900129825.1, GCA_014202435.1, GCA_002238335.1, GCA_017873765.1 GCA_004745645.1, GCA_009695985.1, GCA_004217385.1, GCA_000014705.1, GCA_018424625.1 GCA_024029675.1, GCA_003854965.1, GCA_000815465.1, GCA_000734015.1, GCA_900248245.1 GCA_000022725.1, GCA_020883535.1, GCA_002116735.1, GCA_017329545.1, GCA_010667645.1 GCA_000145215.1, GCA_014779715.1, GCA_900108855.1, GCA_005938215.1, GCA_004025325.1 GCA_001983105.1, GCA_014639235.1, GCA_003344765.1, GCA_002072065.1, GCA_900156645.1 GCA_012979235.1, GCA_020881015.1, GCA_024753245.1, GCA_005280585.1, GCA_016587815.1 GCA_003013475.1, GCA_027570215.1, GCA_024267675.1, GCA_001431415.1, GCA_007625075.1 GCA_002355275.1, GCA_000008385.1, GCA_009828105.1, GCA_000020045.1, GCA_008373765.1 GCA_900119735.1, GCA_007991015.1, GCA_000166395.1, GCA_009177095.1, GCA_016495865.1 GCA_014490575.1, GCA_007828865.1, GCA_024296885.1, GCA_000833355.1, GCA_002532535.1 GCA_001544695.2, GCA_004122735.1, GCA_000376545.2, GCA_009709615.1, GCA_012932215.1 GCA_021282585.1, GCA_024756255.1, GCA_014650915.1, GCA_016482825.1, GCA_003711845.1 GCA_002250655.1, GCA_009877425.1, GCA_009377235.1, GCA_000830905.1, GCA_000765975.1 GCA_900103455.1, GCA_016127855.1, GCA_010671595.1, GCA_023822005.2, GCA_003219795.1 GCA_000231385.3, GCA_900461565.1, GCA_012163135.1, GCA_900101125.1, GCA_023653455.1 GCA_013868055.1, GCA_014204915.1, GCA_000455605.1, GCA_008000835.1, GCA_000020125.1 GCA_025567015.1, GCA_001544475.2, GCA_002217275.1, GCA_027570075.1, GCA_000300335.1 GCA_003096415.1, GCA_000968685.2, GCA_014837015.1, GCA_900104845.1, GCA_001746455.1 GCA_900104535.1, GCA_000155675.2, GCA_003236395.1, GCA_015326725.1, GCA_003991875.1 GCA_000183205.2, GCA_000247565.1, GCA_900095795.1, GCA_013410755.1, GCA_020073815.1 GCA_001435375.1, GCA_003012705.1, GCA_005862185.2, GCA_000767055.1, GCA_028737215.1 GCA_000225525.2, GCA_003231495.1, GCA_001516895.1, GCA_007747795.1, GCA_009600995.1 GCA_019343125.1, GCA_900088825.1, GCA_000468955.1, GCA_014654675.1, GCA_900197575.1 GCA_014207355.1, GCA_003261055.1, GCA_008386485.1, GCA_008014675.1, GCA_003428695.1 GCA_022647665.1, GCA_012910715.1, GCA_007858435.1, GCA_003568825.1, GCA_003315595.1 GCA_001399515.1, GCA_000576575.1, GCA_001008165.2, GCA_009684755.1, GCA_012933655.1 GCA_017837635.1, GCA_014650235.1, GCA_001700325.1, GCA_016908495.1, GCA_900116665.1 GCA_005768555.2, GCA_001307105.1, GCA_003096575.1, GCA_002086545.1, GCA_004368925.1 GCA_000007605.1, GCA_009858245.1, GCA_000757795.1, GCA_014898195.1, GCA_007830635.1 GCA_900100725.1, GCA_900156675.1, GCA_000488215.1, GCA_013141785.1, GCA_000522985.1 GCA_002104335.1, GCA_003970915.1, GCA_014193515.1, GCA_006542355.1, GCA_000756125.1 GCA_000355765.4, GCA_001399675.1, GCA_007559275.1, GCA_009906395.1, GCA_003014755.1 GCA_023284005.1, GCA_001866645.1, GCA_001683395.1, GCA_003052605.1, GCA_010725885.1 GCA_003327535.1, GCA_018129085.1, GCA_016458825.1, GCA_003315245.1, GCA_003054245.1 GCA_900637185.1, GCA_003113265.1, GCA_015139575.1, GCA_003710915.1, GCA_003387535.1 GCA_900108515.1, GCA_003207895.1, GCA_000231405.3, GCA_000092925.1, GCA_022557135.1 GCA_000008865.2, GCA_004005945.1, GCA_002135235.1, GCA_017875465.1, GCA_011764585.1 GCA_000971295.1, GCA_022836845.1, GCA_025950085.1, GCA_016735675.1, GCA_004346165.1 GCA_014203855.1, GCA_001042695.1, GCA_014083925.1, GCA_900156805.1, GCA_004153685.1 GCA_900104485.1, GCA_016775475.1, GCA_900107625.1, GCA_016862475.1, GCA_014636635.1 GCA_005864045.1, GCA_000168295.1, GCA_002217355.1, GCA_007992835.1, GCA_003721215.1 GCA_014534705.1, GCA_019458025.1, GCA_900167445.1, GCA_002858925.1, GCA_016863455.1 GCA_014652375.1, GCA_009883735.1, GCA_900115975.1, GCA_014268935.2, GCA_005885605.1 GCA_013343005.1, GCA_016745505.1, GCA_002744755.1, GCA_006370295.1, GCA_900010725.2 GCA_002813455.1, GCA_003025345.2, GCA_011388215.1, GCA_008329965.1, GCA_004362825.1 GCA_900111515.1, GCA_000016425.1, GCA_024029915.1, GCA_003024235.1, GCA_001012775.1 GCA_020892115.1, GCA_000021805.1, GCA_000764025.1, GCA_001183605.1, GCA_025567195.1 GCA_019219635.1, GCA_007828155.1, GCA_004798685.1, GCA_000770635.1, GCA_000020365.1 GCA_014652475.1, GCA_003987315.1, GCA_003367175.1, GCA_000759445.1, GCA_022179785.1 GCA_000442275.2, GCA_000813125.1, GCA_001278115.1, GCA_019933155.2, GCA_000024725.1 GCA_011189495.1, GCA_900099995.1, GCA_001730365.1, GCA_900143085.1, GCA_000601455.1 GCA_004345735.1, GCA_001465835.2, GCA_000188195.1, GCA_003703815.1, GCA_020447305.2 GCA_001580535.1, GCA_023634645.1, GCA_002847645.1, GCA_900143065.1, GCA_020991125.1 GCA_001695395.1, GCA_016056305.1, GCA_016862055.1, GCA_021184025.1, GCA_020141465.1 GCA_003386075.1, GCA_902459645.1, GCA_016031625.1, GCA_004833285.1, GCA_000769555.1 GCA_000196315.1, GCA_017876545.1, GCA_020735545.1, GCA_002101965.1, GCA_012396585.1 GCA_016653615.1, GCA_002742385.2, GCA_022487425.1, GCA_014639275.1, GCA_016918735.1 GCA_018069875.1, GCA_002245695.1, GCA_005217605.1, GCA_009909145.1, GCA_001953865.1 GCA_009908195.1, GCA_025717515.1, GCA_000010665.1, GCA_019132845.1, GCA_011761635.1 GCA_014138435.1, GCA_004102745.1, GCA_002812485.1, GCA_001298575.1, GCA_002197685.1 GCA_000297055.2, GCA_009760405.1, GCA_900112505.1, GCA_006334525.1, GCA_003325475.1 GCA_013376455.1, GCA_001955715.1, GCA_011927905.1, GCA_014349155.1, GCA_020639365.1 GCA_011440395.1, GCA_005876855.1, GCA_016919245.1, GCA_017498545.1, GCA_000685295.1 GCA_907165215.1, GCA_900101405.1, GCA_001642995.1, GCA_007995155.1, GCA_935825525.1 GCA_900101935.1, GCA_900176145.1, GCA_900110485.1, GCA_020886055.1, GCA_003205575.1 GCA_902813185.1, GCA_022814725.1, GCA_900172295.1, GCA_017751225.1, GCA_003054175.1 GCA_001988955.1, GCA_014652635.1, GCA_001655675.1, GCA_010669205.1, GCA_900461315.1 GCA_001545035.2, GCA_002891435.1, GCA_000328545.1, GCA_009649745.1, GCA_014384745.1 GCA_900107045.1, GCA_003293845.1, GCA_019050325.1, GCA_009733595.1, GCA_021568855.1 GCA_024753215.1, GCA_005884405.1, GCA_004114975.1, GCA_008757455.1, GCA_001720165.1 GCA_016025255.1, GCA_009017255.1, GCA_014138465.1, GCA_016598775.1, GCA_014642075.1 GCA_001447355.1, GCA_004799035.1, GCA_004366795.1, GCA_002216855.1, GCA_014284335.1 GCA_020042285.1, GCA_016918855.1, GCA_022549675.1, GCA_900103815.1, GCA_021608135.1 GCA_002252535.1, GCA_013260645.1, GCA_027570055.1, GCA_900452835.1, GCA_014763015.1 GCA_003172955.1, GCA_025792415.1, GCA_003385845.1, GCA_017638545.1, GCA_000024465.1 GCA_900113965.1, GCA_011299575.1, GCA_018336855.1, GCA_016785945.1, GCA_024171885.1 GCA_014268755.2, GCA_000974365.1, GCA_000013345.1, GCA_024813435.1, GCA_016919345.1 GCA_001438395.1, GCA_019042275.1, GCA_014199685.1, GCA_007993065.1, GCA_900176355.1 GCA_002995755.1, GCA_018138125.1, GCA_000217795.1, GCA_018835585.1, GCA_001518835.1 GCA_009827055.1, GCA_007991455.1, GCA_023559125.1, GCA_001854325.1, GCA_016406105.1 GCA_014837065.1, GCA_003217155.1, GCA_014646755.1, GCA_000478605.2, GCA_018343795.1 GCA_007830155.1, GCA_000235885.1, GCA_014204975.1, GCA_000934545.1, GCA_014649715.1 GCA_001675045.1, GCA_002844235.1, GCA_006538705.1, GCA_001956035.1, GCA_014196835.1 GCA_004336905.1, GCA_000266925.1, GCA_022359095.1, GCA_902141855.1, GCA_001883705.2 GCA_015351395.1, GCA_003846135.1, GCA_003633755.1, GCA_016595155.1, GCA_014647075.1 GCA_013141725.1, GCA_015679265.1, GCA_014195485.1, GCA_014202315.1, GCA_025628765.1 GCA_024299005.1, GCA_900172365.1, GCA_016907515.1, GCA_003664555.1, GCA_900106945.1 GCA_014268655.2, GCA_005144735.1, GCA_016411865.1, GCA_001458775.1, GCA_003124125.1 GCA_004208415.1, GCA_023805255.1, GCA_000521865.1, GCA_900112235.1, GCA_002109495.1 GCA_028023555.1, GCA_011100465.1, GCA_009936155.1, GCA_015167415.1, GCA_002355215.1 GCA_017051705.1, GCA_007846085.1, GCA_000331105.1, GCA_013409425.1, GCA_004116625.1 GCA_000737065.1, GCA_014648415.1, GCA_002327205.1, GCA_003046325.1, GCA_007786445.1 GCA_014652215.1, GCA_004345825.1, GCA_004022465.1, GCA_028596025.1, GCA_012272815.1 GCA_002201795.1, GCA_002251795.1, GCA_000816085.1, GCA_002216145.1, GCA_009725995.1 GCA_003003775.1, GCA_014841215.1, GCA_018454475.1, GCA_004364165.1, GCA_002263495.1 GCA_015694345.1, GCA_900177665.1, GCA_004327335.1, GCA_008297975.1, GCA_006970865.1 GCA_002287965.1, GCA_000685275.1, GCA_006861675.1, GCA_003336225.1, GCA_003315035.1 GCA_019048385.1, GCA_024519315.1, GCA_000169235.1, GCA_004363655.1, GCA_001561955.1 GCA_002262935.1, GCA_014641655.1, GCA_000981585.1, GCA_008802355.1, GCA_001648475.1 GCA_004167665.1, GCA_003217075.1, GCA_014646615.1, GCA_000024765.1, GCA_011761265.1 GCA_016031655.1, GCA_014623465.1, GCA_003369565.1, GCA_007559335.1, GCA_900114315.1 GCA_007992095.1, GCA_000223215.1, GCA_020532675.1, GCA_002841335.1, GCA_900107755.1 GCA_003710965.1, GCA_014647835.1, GCA_018800825.1, GCA_000213235.1, GCA_016025525.2 GCA_002934605.1, GCA_001693385.1, GCA_018982885.2, GCA_917563885.1, GCA_013408685.1 GCA_900110255.1, GCA_004363005.1, GCA_900167525.1, GCA_019890955.1, GCA_002258215.1 GCA_001876955.1, GCA_003952165.1, GCA_007097155.1, GCA_900103845.1, GCA_900638345.1 GCA_014203755.1, GCA_000622365.1, GCA_900109375.1, GCA_002288365.1, GCA_003114815.1 GCA_003258315.1, GCA_013372285.1, GCA_900109685.1, GCA_004766125.1, GCA_004345705.1 GCA_900609055.1, GCA_902499555.1, GCA_000393975.1, GCA_900109295.1, GCA_016765615.1 GCA_014205875.1, GCA_003044035.1, GCA_013309895.1, GCA_900167275.1, GCA_004341445.1 GCA_003086355.2, GCA_006274405.1, GCA_004118375.1, GCA_023712105.1, GCA_018403325.1 GCA_014357575.1, GCA_014202475.1, GCA_002959935.1, GCA_900182615.1, GCA_003335485.1 GCA_900112885.1, GCA_900452395.1, GCA_002259645.1, GCA_900659865.1, GCA_000348925.1 GCA_000802305.1, GCA_003293695.1, GCA_025758395.1, GCA_003024815.1, GCA_000317145.1 GCA_900453675.1, GCA_027922265.1, GCA_000331515.1, GCA_001942625.1, GCA_003858635.1 GCA_003007685.1, GCA_003813985.1, GCA_003351745.1, GCA_003003845.1, GCA_002742165.1 GCA_017377355.1, GCA_000092785.1, GCA_003945365.1, GCA_001435995.1, GCA_016117655.1 GCA_015140235.1, GCA_016746095.1, GCA_027922105.1, GCA_003762925.1, GCA_014268495.2 GCA_014648635.1, GCA_001650695.1, GCA_003217355.1, GCA_014207075.1, GCA_004519315.1 GCA_003703885.1, GCA_004363595.1, GCA_000019905.1, GCA_001629705.2, GCA_009936175.1 GCA_000243115.3, GCA_900095135.1, GCA_025398255.1, GCA_013002715.1, GCA_002082175.1 GCA_007558725.1, GCA_005860765.1, GCA_027944115.1, GCA_002998295.1, GCA_016745235.1 GCA_016428595.1, GCA_003627035.1, GCA_017349295.1, GCA_001458735.1, GCA_022899245.1 GCA_900103735.1, GCA_009664835.1, GCA_004340205.1, GCA_002220155.1, GCA_008692955.1 GCA_006546625.1, GCA_900095025.1, GCA_001307195.1, GCA_008805035.1, GCA_001874525.1 GCA_001982605.1, GCA_900141715.1, GCA_009647605.1, GCA_025145985.1, GCA_003576375.1 GCA_009910725.1, GCA_000967625.1, GCA_002234575.2, GCA_001434475.1, GCA_014202325.1 GCA_900102045.1, GCA_000196555.1, GCA_900454855.1, GCA_016908525.1, GCA_001519075.1 GCA_011045115.1, GCA_900105715.1, GCA_008000935.1, GCA_000026105.1, GCA_003129925.1 GCA_900156725.1, GCA_008630015.1, GCA_011812995.1, GCA_005670685.2, GCA_009831765.1 GCA_900107695.1, GCA_007556605.1, GCA_001007935.1, GCA_004916905.1, GCA_014196295.1 GCA_002933275.1, GCA_014640635.1, GCA_020886775.1, GCA_026341945.1, GCA_010731635.1 GCA_003003055.1, GCA_002846655.1, GCA_023373525.1, GCA_005116735.1, GCA_016735585.2 GCA_017868935.1, GCA_003668875.1, GCA_001565895.1, GCA_022678485.1, GCA_900155925.1 GCA_014696555.1, GCA_002911015.1, GCA_003336705.1, GCA_007431345.1, GCA_024349285.1 GCA_900100165.1, GCA_001431525.1, GCA_003843875.1, GCA_007556705.1, GCA_000812665.2 GCA_026127345.1, GCA_016862155.1, GCA_000022145.1, GCA_003176735.1, GCA_000816575.1 GCA_900167515.1, GCA_002278075.1, GCA_008369605.1, GCA_000204015.1, GCA_003544815.1 GCA_001307545.1, GCA_003612775.1, GCA_900187885.1, GCA_003386575.1, GCA_003945585.1 GCA_014648095.1, GCA_003814915.1, GCA_012863495.1, GCA_000019405.1, GCA_023349125.1 GCA_014305155.1, GCA_003862475.1, GCA_900101995.1, GCA_022524485.1, GCA_000017845.1 GCA_900044055.2, GCA_000011345.1, GCA_900107355.1, GCA_014199445.1, GCA_007745435.1 GCA_003315405.1, GCA_009258045.1, GCA_003716325.1, GCA_900113085.1, GCA_018491765.1 GCA_002980625.1, GCA_011089875.1, GCA_004403305.1, GCA_013372265.1, GCA_003202065.1 GCA_001718635.1, GCA_020076305.1, GCA_024721995.1, GCA_001661075.1, GCA_002847305.1 GCA_024102615.1, GCA_000964365.1, GCA_004103735.1, GCA_018739485.1, GCA_900018365.1 GCA_900560965.1, GCA_900106995.1, GCA_003097295.1, GCA_008000755.1, GCA_027118995.1 GCA_001235865.1, GCA_001543305.1, GCA_003410415.1, GCA_007197555.1, GCA_002858825.1 GCA_900103185.1, GCA_000021645.1, GCA_004912135.1, GCA_007751035.1, GCA_002288525.1 GCA_014649375.1, GCA_000243715.3, GCA_001981305.1, GCA_014635305.1, GCA_001595645.1 GCA_003298795.1, GCA_024054555.1, GCA_019430925.1, GCA_016746085.1, GCA_005405925.1 GCA_020026895.1, GCA_000740965.1, GCA_000367525.2, GCA_000215975.1, GCA_003581645.1 GCA_001637205.1, GCA_013093415.1, GCA_002749615.1, GCA_002860625.1, GCA_019132875.1 GCA_009184705.1, GCA_003337575.1, GCA_018332955.1, GCA_001518795.1, GCA_001482365.1 GCA_002982315.1, GCA_003050615.1, GCA_015222005.1, GCA_002234495.1, GCA_900108365.1 GCA_000967915.1, GCA_900458435.1, GCA_000238295.4, GCA_012911875.1, GCA_014861485.1 GCA_003987065.1, GCA_003575975.1, GCA_014836635.1, GCA_900111375.1, GCA_900111005.1 GCA_012911605.1, GCA_014203605.1, GCA_020905415.1, GCA_014655295.1, GCA_019711335.1 GCA_001645135.1, GCA_001854475.1, GCA_008087625.1, GCA_004358105.1, GCA_004348455.1 GCA_001274945.1, GCA_003386535.1, GCA_014192415.1, GCA_004118285.1, GCA_022953055.1 GCA_001983935.1, GCA_003149535.1, GCA_000393015.1, GCA_002291445.1, GCA_000746925.1 GCA_016908555.1, GCA_010093065.1, GCA_017873135.1, GCA_009601505.1, GCA_020215685.1 GCA_014621695.1, GCA_900101105.1, GCA_025345565.1, GCA_014698475.1, GCA_007748115.1 GCA_900292015.1, GCA_014207745.1, GCA_019104725.1, GCA_018598175.1, GCA_900129875.1 GCA_013868155.1, GCA_008693705.1, GCA_000020305.1, GCA_900107815.1, GCA_900199185.1 GCA_014201585.1, GCA_015034585.1, GCA_004769555.1, GCA_013409365.1, GCA_002764115.1 GCA_000803315.1, GCA_016592615.1, GCA_016728105.1, GCA_017114865.1, GCA_007827695.1 GCA_009833495.1, GCA_003634095.1, GCA_014385195.1, GCA_019141545.1, GCA_004362975.1 GCA_024462265.1, GCA_001444425.1, GCA_012396015.1, GCA_017901175.1, GCA_002068575.1 GCA_007970805.1, GCA_016758175.1, GCA_000165795.1, GCA_018283645.1, GCA_900105005.1 GCA_003097615.1, GCA_000878195.1, GCA_019974115.1, GCA_008867985.1, GCA_014201555.1 GCA_000243155.3, GCA_014843995.1, GCA_900128995.1, GCA_023227745.1, GCA_017893965.1 GCA_000341395.1, GCA_001758785.1, GCA_009909205.1, GCA_019711735.1, GCA_000152885.1 GCA_900188205.1, GCA_021044795.1, GCA_007004065.1, GCA_002915575.1, GCA_003721565.1 GCA_019711565.1, GCA_013294015.1, GCA_001436225.1, GCA_003364205.1, GCA_014645615.1 GCA_000146895.1, GCA_015207035.1, GCA_003628145.1, GCA_014197105.1, GCA_011601315.1 GCA_004795745.1, GCA_900129205.1, GCA_009857475.1, GCA_000243675.3, GCA_026013645.1 GCA_900105555.1, GCA_003732545.1, GCA_017316205.1, GCA_000145255.1, GCA_013618545.1 GCA_001544535.2, GCA_001277195.1, GCA_017377395.1, GCA_000695625.1, GCA_017942085.1 GCA_019704495.1, GCA_014649675.1, GCA_028607025.1, GCA_004134905.2, GCA_900100155.1 GCA_005116455.1, GCA_900107245.1, GCA_003289925.1, GCA_001542415.1, GCA_900111285.1 GCA_003284725.1, GCA_900103065.1, GCA_009428905.1, GCA_002278035.1, GCA_028622095.1 GCA_000199675.1, GCA_900188115.1, GCA_007641255.1, GCA_000018205.1, GCA_000184685.1 GCA_015775975.1, GCA_003987105.1, GCA_009811575.1, GCA_000009785.1, GCA_004331335.1 GCA_014641735.1, GCA_000685315.1, GCA_014874055.1, GCA_003007715.1, GCA_012317185.1 GCA_017874415.1, GCA_004337635.1, GCA_000198775.1, GCA_001904655.1, GCA_016126035.1 GCA_003710935.1, GCA_017498775.1, GCA_007556775.1, GCA_902459805.1, GCA_003574085.1 GCA_029025825.1, GCA_014284235.1, GCA_014653855.1, GCA_017068355.1, GCA_000765825.2 GCA_900499075.1, GCA_011694815.1, GCA_025567495.1, GCA_014648995.1, GCA_003053745.1 GCA_007556585.1, GCA_000174355.1, GCA_003315175.1, GCA_019239235.1, GCA_003323745.1 GCA_007623795.1, GCA_900454535.1, GCA_014646235.1, GCA_018860155.1, GCA_900218015.1 GCA_019748715.1, GCA_022538045.1, GCA_001991015.1, GCA_011326735.1, GCA_015356855.1 GCA_003967075.1, GCA_011045015.1, GCA_008065135.1, GCA_020097475.1, GCA_001305595.1 GCA_014637265.1, GCA_900116555.1, GCA_900109735.1, GCA_007833215.1, GCA_020164495.1 GCA_027563335.1, GCA_003415675.1, GCA_003148625.1, GCA_015594545.1, GCA_012912135.1 GCA_001632805.1, GCA_900114595.1, GCA_016939435.1, GCA_000788395.1, GCA_007830415.1 GCA_017313785.1, GCA_019504385.1, GCA_024029335.1, GCA_008629655.1, GCA_000297075.2 GCA_025060895.1, GCA_021725675.1, GCA_015244705.1, GCA_009811375.1, GCA_001750685.1 GCA_900450505.1, GCA_001936295.1, GCA_900096995.1, GCA_002951835.1, GCA_900187305.1 GCA_002000485.1, GCA_023573625.1, GCA_020443705.1, GCA_003268475.1, GCA_900108115.1 GCA_000359525.1, GCA_900105695.1, GCA_012910905.1, GCA_014203645.1, GCA_900104215.1 GCA_014384795.1, GCA_001436275.1, GCA_002000425.1, GCA_003034915.1, GCA_900536025.1 GCA_007830875.1, GCA_002198095.1, GCA_000178955.2, GCA_002877605.2, GCA_014138385.1 GCA_000187585.1, GCA_029223525.1, GCA_003634105.1, GCA_024622425.1, GCA_002285575.1 GCA_014197055.1, GCA_000974425.1, GCA_016583835.1, GCA_019357495.1, GCA_000725405.1 GCA_000315795.1, GCA_018760735.1, GCA_014295015.1, GCA_015627165.1, GCA_026013525.1 GCA_010667685.1, GCA_028771845.1, GCA_010728155.1, GCA_020861345.1, GCA_000367925.1 GCA_003236335.1, GCA_005860775.1, GCA_902502825.2, GCA_003344445.1, GCA_020042345.1 GCA_012932965.1, GCA_900112355.1, GCA_000183725.1, GCA_019711375.1, GCA_000317105.1 GCA_001904635.1, GCA_002797715.1, GCA_003696365.1, GCA_021168455.1, GCA_008632635.1 GCA_900184945.1, GCA_004348975.1, GCA_009695545.1, GCA_020138775.1, GCA_003096675.1 GCA_900108245.1, GCA_014395425.1, GCA_017963645.1, GCA_009650095.1, GCA_900096945.1 GCA_900459215.1, GCA_013378075.1, GCA_900113315.1, GCA_013376415.1, GCA_017583065.1 GCA_002076835.1, GCA_001045555.1, GCA_012923785.1, GCA_004362665.1, GCA_002954325.1 GCA_003207865.1, GCA_003952225.1, GCA_004339765.1, GCA_003063625.1, GCA_001633025.1 GCA_002998925.1, GCA_000015045.1, GCA_004364125.1, GCA_000714935.1, GCA_900465355.1 GCA_016908955.1, GCA_003337425.1, GCA_003987435.1, GCA_023155275.1, GCA_014836775.1 GCA_003931855.1, GCA_000025605.1, GCA_900101815.1, GCA_000219725.1, GCA_900116635.1 GCA_003346815.1, GCA_001267535.1, GCA_002148965.1, GCA_002860365.1, GCA_013177315.1 GCA_001402675.1, GCA_003345525.1, GCA_023108895.1, GCA_000307855.1, GCA_003014595.1 GCA_000412335.2, GCA_016586355.1, GCA_900230175.1, GCA_011440275.1, GCA_007097365.1 GCA_024804185.1, GCA_900112365.1, GCA_014652915.1, GCA_001648355.1, GCA_011492965.1 GCA_900104745.1, GCA_018729315.1, GCA_900110065.1, GCA_003635045.1, GCA_004103825.1 GCA_022179285.1, GCA_000753735.1, GCA_023006325.1, GCA_000384965.1, GCA_003987175.1 GCA_002173515.1, GCA_003574835.2, GCA_003789055.1, GCA_000576635.1, GCA_023613945.1 GCA_014640255.1, GCA_008011915.1, GCA_014306175.1, GCA_007988825.1, GCA_009730295.1 GCA_003254705.1, GCA_000943555.1, GCA_025446935.1, GCA_011682235.2, GCA_001431295.2 GCA_002843175.1, GCA_000222975.1, GCA_018917195.1, GCA_019297855.1, GCA_005862345.1 GCA_003431825.1, GCA_001434915.1, GCA_000020145.1, GCA_003570885.1, GCA_014145675.1 GCA_002798435.1, GCA_020923435.1, GCA_025397885.1, GCA_012972675.1, GCA_016093295.1 GCA_020310005.1, GCA_000241305.2, GCA_003364335.1, GCA_003012795.1, GCA_002909415.1 GCA_014199665.1, GCA_014201505.1, GCA_003937825.1, GCA_014203715.1, GCA_013364095.1 GCA_001499655.1, GCA_000328705.1, GCA_001431725.1, GCA_025215115.1, GCA_900111225.1 GCA_010731855.1, GCA_014050145.1, GCA_000299355.1, GCA_001975665.1, GCA_001719165.1 GCA_016865445.1, GCA_001460635.1, GCA_021556395.1, GCA_000091405.1, GCA_000445445.1 GCA_000253275.1, GCA_002097535.1, GCA_010731755.1, GCA_019145355.1, GCA_900100785.1 GCA_900100885.1, GCA_016820655.1, GCA_014639315.1, GCA_900142025.1, GCA_014636475.1 GCA_012223425.1, GCA_009695745.1, GCA_000817775.3, GCA_002940085.1, GCA_007556595.1 GCA_006540045.1, GCA_024752335.1, GCA_014635665.1, GCA_900107595.1, GCA_001507595.1 GCA_002268635.1, GCA_004209755.1, GCA_009829925.1, GCA_018219815.1, GCA_000091305.1 GCA_016908325.1, GCA_002564045.1, GCA_010537335.1, GCA_907164845.1, GCA_002933575.1 GCA_007814115.1, GCA_003015125.1, GCA_003385055.1, GCA_014645815.1, GCA_006175995.1 GCA_900142175.1, GCA_018687595.1, GCA_010692725.1, GCA_900115565.1, GCA_003944715.1 GCA_013177735.1, GCA_008245045.1, GCA_900105865.1, GCA_000468495.1, GCA_001494635.1 GCA_007988885.1, GCA_003716585.1, GCA_013141775.1, GCA_020328095.1, GCA_003464225.1 GCA_000025065.1, GCA_002735305.1, GCA_003003255.1, GCA_023231945.1, GCA_001431405.1 GCA_021245805.1, GCA_000153225.1, GCA_001712815.1, GCA_021555155.1, GCA_003640665.1 GCA_001274725.1, GCA_010727605.1, GCA_004404025.1, GCA_010730355.1, GCA_003600245.1 GCA_000737325.2, GCA_900156785.1, GCA_900168005.1, GCA_001275345.1, GCA_014930455.1 GCA_001865765.1, GCA_017348915.1, GCA_008123965.1, GCA_003710985.1, GCA_013087515.1 GCA_003991975.1, GCA_000021565.1, GCA_008086185.1, GCA_003432115.1, GCA_002872015.2 GCA_013166605.1, GCA_000020645.1, GCA_026871175.2, GCA_008630065.1, GCA_008039565.1 GCA_020783315.1, GCA_900187095.1, GCA_022637475.1, GCA_022968825.1, GCA_002760615.1 GCA_000335475.2, GCA_007859195.1, GCA_016728645.1, GCA_000183425.1, GCA_007991615.1 GCA_006636205.1, GCA_900109525.1, GCA_010435965.1, GCA_027922185.1, GCA_003012775.1 GCA_003725415.1, GCA_001655685.1, GCA_900453305.1, GCA_900176525.1, GCA_000019705.1 GCA_013410525.1, GCA_000018665.1, GCA_001941505.1, GCA_004364575.1, GCA_900105505.1 GCA_002795845.1, GCA_013408795.1, GCA_012844305.1, GCA_018138385.1, GCA_900113485.1 GCA_017068395.1, GCA_014699115.1, GCA_002174535.1, GCA_020171505.1, GCA_002346025.1 GCA_907163265.1, GCA_000967305.2, GCA_018734325.1, GCA_011290545.1, GCA_014656375.1 GCA_002068775.1, GCA_002272085.1, GCA_003330725.1, GCA_023208015.1, GCA_003627755.1 GCA_027921845.1, GCA_000342005.1, GCA_020405475.1, GCA_003522965.1, GCA_027915275.1 GCA_017873235.1, GCA_009674535.1, GCA_014644435.1, GCA_021147805.1, GCA_004116025.1 GCA_000021985.1, GCA_021764745.1, GCA_002014965.1, GCA_002802865.1, GCA_026310195.1 GCA_014635685.1, GCA_900207585.1, GCA_000298215.1, GCA_000507245.1, GCA_016801755.1 GCA_014873935.1, GCA_001642325.1, GCA_003026895.1, GCA_000695795.1, GCA_022012555.1 GCA_003795125.1, GCA_000300995.1, GCA_001267885.1, GCA_900475025.1, GCA_013363975.1 GCA_008831385.1, GCA_014841125.1, GCA_003691585.1, GCA_004769745.1, GCA_016907675.1 GCA_029167075.1, GCA_016888945.1, GCA_003002925.1, GCA_000192865.1, GCA_001866075.3 GCA_014649595.1, GCA_014268445.2, GCA_001956775.1, GCA_007860045.1, GCA_900101705.1 GCA_022603395.1, GCA_016785085.1, GCA_003386555.1, GCA_009734405.1, GCA_900105025.1 GCA_000212395.1, GCA_000262755.1, GCA_002072105.2, GCA_006974105.1, GCA_009734425.1 GCA_017497975.1, GCA_024125165.1, GCA_000212695.1, GCA_003353055.1, GCA_016862195.1 GCA_003627815.1, GCA_022836475.1, GCA_900155935.1, GCA_002266285.1, GCA_900167075.1 GCA_003944705.1, GCA_003987565.1, GCA_009674805.1, GCA_013149785.1, GCA_014652315.1 GCA_900090315.1, GCA_000739935.1, GCA_005116665.1, GCA_002797775.1, GCA_001274715.1 GCA_000010305.1, GCA_003570845.1, GCA_900156735.1, GCA_024742095.1, GCA_900104245.1 GCA_018314255.1, GCA_006517255.1, GCA_004299785.2, GCA_024498075.1, GCA_002843035.1 GCA_001404635.1, GCA_002242805.1, GCA_003611585.1, GCA_900129545.1, GCA_002205675.1 GCA_021532615.1, GCA_024159085.1, GCA_003055625.1, GCA_025567445.1, GCA_000190575.1 GCA_900129155.1, GCA_019090985.1, GCA_001406035.1, GCA_006491595.2, GCA_001278365.1 GCA_016860625.1, GCA_002959775.1, GCA_009648935.1, GCA_004403515.1, GCA_007860185.1 GCA_001854695.1, GCA_011382965.1, GCA_003121985.1, GCA_002891295.1, GCA_016638705.1 GCA_900101475.1, GCA_001050135.1, GCA_000876205.1, GCA_900102335.1, GCA_022410535.1 GCA_013004605.1, GCA_021044755.1, GCA_009697165.1, GCA_900217235.1, GCA_020216525.1 GCA_000758685.1, GCA_003583405.1, GCA_002355375.1, GCA_007004655.1, GCA_900111355.1 GCA_900099935.1, GCA_900637495.1, GCA_002117125.1, GCA_014694385.1, GCA_003951975.1 GCA_002102355.1, GCA_900177735.1, GCA_002285715.1, GCA_022690625.1, GCA_003044185.1 GCA_007066085.1, GCA_008693005.1, GCA_018917845.1, GCA_005671395.1, GCA_004331695.1 GCA_019139675.1, GCA_019402665.1, GCA_003751805.1, GCA_000314995.1, GCA_004681155.1 GCA_009601015.1, GCA_004342725.1, GCA_020889685.1, GCA_018332455.1, GCA_004348725.1 GCA_023035295.1, GCA_000737705.1, GCA_003550175.1, GCA_900636685.1, GCA_003051085.1 GCA_003429605.1, GCA_014648135.1, GCA_025263685.1, GCA_002209165.2, GCA_000238275.4 GCA_024793055.1, GCA_021733145.1, GCA_022811605.1, GCA_014646195.1, GCA_024464675.1 GCA_002288545.1, GCA_000988745.3, GCA_003323715.1, GCA_900112975.1, GCA_900459365.1 GCA_017876775.1, GCA_016863655.1, GCA_900100395.1, GCA_014779665.1, GCA_001605895.1 GCA_018388465.1, GCA_000830985.1, GCA_014205015.1, GCA_000517425.1, GCA_017896165.1 GCA_017639205.1, GCA_000814735.1, GCA_013372205.1, GCA_006540085.1, GCA_023653475.1 GCA_002906165.1, GCA_003363485.1, GCA_000416965.1, GCA_006542275.1, GCA_001433995.1 GCA_001314995.1, GCA_014352855.1, GCA_025822865.1, GCA_004331435.1, GCA_010499455.1 GCA_014649535.1, GCA_001729245.1, GCA_009734005.2, GCA_017776525.1, GCA_000307585.2 GCA_900182545.1, GCA_016907655.1, GCA_023573145.1, GCA_018861735.1, GCA_900091615.1 GCA_021733725.1, GCA_004768465.1, GCA_006542395.2, GCA_003364315.1, GCA_020531725.1 GCA_001439225.1, GCA_013408175.1, GCA_024733725.1, GCA_002894165.1, GCA_014640675.1 GCA_028747985.1, GCA_000284615.1, GCA_004358325.1, GCA_002924615.1, GCA_004322755.1 GCA_001305515.1, GCA_003014615.1, GCA_900176465.1, GCA_013365435.1, GCA_900142605.1 GCA_001310085.1, GCA_003555545.1, GCA_001513955.1, GCA_004354015.1, GCA_014673495.1 GCA_009674685.1, GCA_016093275.1, GCA_900110375.1, GCA_000152825.2, GCA_001431315.2 GCA_024198215.1, GCA_029223485.1, GCA_001294205.1, GCA_019891395.1, GCA_000974985.2 GCA_000970775.1, GCA_001932615.1, GCA_002221525.1, GCA_003149515.1, GCA_002762215.1 GCA_008119665.1, GCA_003433515.1, GCA_014863405.1, GCA_023008305.1, GCA_002954545.1 GCA_900156575.1, GCA_002217335.1, GCA_002223275.1, GCA_017309175.1, GCA_022846135.1 GCA_900115865.1, GCA_003688665.1, GCA_016055185.1, GCA_006770325.1, GCA_007559025.1 GCA_002215585.1, GCA_901764995.1, GCA_021654655.1, GCA_027922025.1, GCA_012034385.1 GCA_014648395.1, GCA_014490485.1, GCA_002861965.1, GCA_000067205.1, GCA_018474025.1 GCA_011751765.2, GCA_009735315.1, GCA_008632235.1, GCA_016724865.1, GCA_900114545.1 GCA_002105555.1, GCA_009709675.1, GCA_002646595.1, GCA_000722545.1, GCA_009617915.1 GCA_004324755.1, GCA_001012825.2, GCA_004358895.1, GCA_015070385.1, GCA_021026195.1 GCA_018398355.1, GCA_015265475.1, GCA_004801405.1, GCA_008693045.1, GCA_009711405.1 GCA_004363335.1, GCA_000214595.2, GCA_003015185.1, GCA_000227745.3, GCA_024621935.1 GCA_000359605.1, GCA_002906255.1, GCA_019890635.1, GCA_003119195.2, GCA_018343625.1 GCA_001718895.1, GCA_008386575.1, GCA_001693735.1, GCA_900130005.1, GCA_016576965.1 GCA_007971525.1, GCA_018717645.1, GCA_003583365.1, GCA_011308835.1, GCA_900114875.1 GCA_003610495.1, GCA_019659805.1, GCA_021018745.1, GCA_000622405.1, GCA_900090275.1 GCA_000327045.1, GCA_002269685.1, GCA_001437125.1, GCA_025200715.1, GCA_024055595.1 GCA_900141965.1, GCA_016900955.1, GCA_000277895.2, GCA_020880995.1, GCA_002153515.1 GCA_009827635.1, GCA_003634995.1, GCA_001816125.1, GCA_013364395.1, GCA_003340475.1 GCA_019602855.1, GCA_017874285.1, GCA_002786295.1, GCA_010122465.1, GCA_020911725.1 GCA_024105745.1, GCA_024649945.1, GCA_004342005.1, GCA_900157375.1, GCA_024171945.1 GCA_002082605.1, GCA_900102815.1, GCA_016019885.1, GCA_900142105.1, GCA_002302475.1 GCA_014268615.1, GCA_009856445.1, GCA_016861975.1, GCA_021568315.1, GCA_012932915.1 GCA_012033695.1, GCA_000153105.1, GCA_022376295.1, GCA_010508875.1, GCA_000348945.1 GCA_900215535.1, GCA_010131535.1, GCA_001997295.1, GCA_025264625.1, GCA_002234405.1 GCA_900221025.1, GCA_000165505.1, GCA_001659785.1, GCA_007991355.1, GCA_001703555.1 GCA_014645755.1, GCA_016446285.1, GCA_009761375.1, GCA_016862675.1, GCA_000153205.1 GCA_900115955.1, GCA_009789595.1, GCA_014212055.1, GCA_003025495.1, GCA_900177725.1 GCA_003240585.1, GCA_900177655.1, GCA_015207065.1, GCA_000014765.1, GCA_902728305.1 GCA_008017445.1, GCA_900453685.1, GCA_900100845.1, GCA_014651995.1, GCA_900113955.1 GCA_900250125.1, GCA_015097275.1, GCA_016908315.1, GCA_012396505.1, GCA_027270575.1 GCA_900168055.1, GCA_000385435.1, GCA_027270315.1, GCA_014982805.1, GCA_009910365.1 GCA_003724155.1, GCA_014207905.1, GCA_000091785.1, GCA_006716085.1, GCA_009026825.1 GCA_003612125.1, GCA_009711475.1, GCA_900112695.1, GCA_004803475.1, GCA_003001695.1 GCA_900167085.1, GCA_017876395.1, GCA_001293565.1, GCA_015234135.1, GCA_001936625.1 GCA_023715385.1, GCA_015377145.2, GCA_004341375.1, GCA_003363155.1, GCA_002760665.1 GCA_014201465.1, GCA_008121305.1, GCA_000058485.1, GCA_001593985.1, GCA_900187355.1 GCA_003097655.1, GCA_012689545.1, GCA_000963865.1, GCA_004341205.1, GCA_000022065.1 GCA_001028665.1, GCA_001660485.1, GCA_000974685.2, GCA_014649755.1, GCA_900129085.1 GCA_028010245.1, GCA_000190735.2, GCA_900108065.1, GCA_017254915.1, GCA_000233915.4 GCA_000531125.1, GCA_900459405.1, GCA_900119145.1, GCA_017167985.1, GCA_011617105.1 GCA_020010925.1, GCA_935822925.1, GCA_900103645.1, GCA_001078595.1, GCA_023156285.1 GCA_002252325.1, GCA_010723575.1, GCA_000092885.1, GCA_018256975.1, GCA_017569225.1 GCA_011578285.1, GCA_900453455.1, GCA_000010085.1, GCA_000145945.2, GCA_011682065.1 GCA_004378255.1, GCA_015710995.1, GCA_003185895.1, GCA_010119615.1, GCA_900187315.1 GCA_001399775.1, GCA_900156595.1, GCA_900637635.1, GCA_900186975.1, GCA_900103695.1 GCA_007991775.1, GCA_018141405.1, GCA_021129305.1, GCA_014649895.1, GCA_021404985.1 GCA_013085545.1, GCA_010435915.1, GCA_022179105.1, GCA_900141975.1, GCA_014750655.1 GCA_016599755.1, GCA_019668505.1, GCA_017676365.1, GCA_016811105.1, GCA_000019185.1 GCA_010093225.1, GCA_004208535.1, GCA_007361795.1, GCA_028657195.3, GCA_022512515.1 GCA_902806695.1, GCA_016909015.1, GCA_025753815.1, GCA_019704535.1, GCA_001735525.1 GCA_006516955.1, GCA_000954135.2, GCA_024106375.1, GCA_001687625.2, GCA_011067265.1 GCA_002078315.1, GCA_900220975.1, GCA_013371085.1, GCA_013372045.1, GCA_019145235.1 GCA_002844275.1, GCA_004307015.1, GCA_015476335.1, GCA_003994255.1, GCA_025137635.1 GCA_003987655.1, GCA_013385825.1, GCA_000144605.1, GCA_003626655.1, GCA_003994345.2 GCA_004803505.1, GCA_016900895.1, GCA_004758605.1, GCA_001619695.1, GCA_016907385.1 GCA_022179645.1, GCA_023195775.1, GCA_002966125.1, GCA_000147695.3, GCA_005406215.1 GCA_900114375.1, GCA_017254865.1, GCA_002355415.1, GCA_008807015.1, GCA_007829925.1 GCA_024105735.1, GCA_002861525.1, GCA_003217515.1, GCA_023284065.1, GCA_021532315.1 GCA_004363095.1, GCA_001439025.1, GCA_014643695.1, GCA_000212675.2, GCA_023130475.1 GCA_016584105.1, GCA_009857605.1, GCA_001705075.2, GCA_003290445.1, GCA_001653195.1 GCA_014137995.1, GCA_020995475.1, GCA_007641235.1, GCA_011764425.1, GCA_007988905.1 GCA_900141895.1, GCA_018156225.1, GCA_900156685.1, GCA_003013245.1, GCA_003688495.1 GCA_018863395.1, GCA_000014145.1, GCA_006704145.1, GCA_014636865.1, GCA_026735165.1 GCA_025399795.1, GCA_900141805.1, GCA_927798215.1, GCA_001552785.1, GCA_001435245.1 GCA_002205645.1, GCA_014642405.1, GCA_003944755.1, GCA_001953955.1, GCA_001594005.1 GCA_023634845.1, GCA_003812925.1, GCA_000083545.1, GCA_003362755.1, GCA_007995065.1 GCA_003987495.1, GCA_025147765.1, GCA_014192515.1, GCA_018491735.2, GCA_900110575.1 GCA_000961885.1, GCA_016908935.1, GCA_014647875.1, GCA_023195735.1, GCA_014190805.1 GCA_024029435.1, GCA_025914135.1, GCA_003940805.1, GCA_013386115.1, GCA_020736405.1 GCA_009604405.1, GCA_004208525.1, GCA_008830365.1, GCA_000948395.1, GCA_017498685.1 GCA_003287455.1, GCA_026930265.1, GCA_014712675.1, GCA_001439085.1, GCA_900116005.1 GCA_000146505.1, GCA_021021555.1, GCA_004771075.1, GCA_014204935.1, GCA_010722915.1 GCA_016653255.1, GCA_011008935.1, GCA_009756665.1, GCA_003205515.1, GCA_020819595.1 GCA_900107605.1, GCA_003309065.1, GCA_003385595.1, GCA_013359775.1, GCA_000007625.1 GCA_002995745.1, GCA_900104815.1, GCA_002149925.1, GCA_002290485.1, GCA_001941975.1 GCA_900113605.1, GCA_013359905.1, GCA_008064635.1, GCA_021922925.1, GCA_011927955.1 GCA_014207645.1, GCA_016031635.1, GCA_003940825.1, GCA_004346135.1, GCA_002295105.1 GCA_000215935.3, GCA_006788895.1, GCA_000454025.1, GCA_900163545.1, GCA_003584165.1 GCA_000026185.1, GCA_004341725.1, GCA_014201515.1, GCA_020551945.1, GCA_003610355.1 GCA_014145225.1, GCA_001021495.1, GCA_002632755.1, GCA_014270105.1, GCA_004346745.1 GCA_001544615.2, GCA_003721275.1, GCA_003752155.1, GCA_018141445.1, GCA_900094795.1 GCA_002154225.1, GCA_004367585.1, GCA_001010945.1, GCA_010093445.1, GCA_014218725.1 GCA_900113275.1, GCA_900129945.1, GCA_018919175.1, GCA_900101025.1, GCA_001642805.2 GCA_003071065.1, GCA_003851885.1, GCA_000504585.2, GCA_000753795.1, GCA_002210435.1 GCA_021608225.1, GCA_016908565.1, GCA_000152245.2, GCA_009380215.1, GCA_020297465.1 GCA_005046025.1, GCA_016583905.1, GCA_900636675.1, GCA_900104445.1, GCA_001687565.2 GCA_900102085.1, GCA_014645535.1, GCA_001584615.1, GCA_000691805.2, GCA_000733715.2 GCA_000682675.1, GCA_012029655.1, GCA_014705655.1, GCA_004368965.1, GCA_002608105.1 GCA_014644035.1, GCA_019145475.1, GCA_007994075.1, GCA_000819565.1, GCA_000147335.1 GCA_006265245.1, GCA_014196455.1, GCA_019331695.1, GCA_004005935.1, GCA_027587205.1 GCA_003991135.1, GCA_007846095.1, GCA_001439035.1, GCA_004117075.1, GCA_002770725.1 GCA_003208655.1, GCA_016698665.1, GCA_015686695.1, GCA_007556685.1, GCA_015244785.1 GCA_000013405.1, GCA_001663175.1, GCA_001698205.1, GCA_003053845.1, GCA_002009295.1 GCA_017874425.1, GCA_013450155.1, GCA_007197575.1, GCA_900129055.1, GCA_001436735.1 GCA_014268695.2, GCA_001436555.1, GCA_014640175.1, GCA_000191105.1, GCA_001437405.1 GCA_003194085.1, GCA_900450575.1, GCA_007280785.1, GCA_001405015.1, GCA_027922405.1 GCA_003412455.1, GCA_000407205.1, GCA_002864175.1, GCA_008694225.1, GCA_001667535.1 GCA_001443605.1, GCA_025234715.1, GCA_003014435.1, GCA_014648955.1, GCA_013128195.2 GCA_019166065.1, GCA_004114895.1, GCA_003003375.1, GCA_014836645.1, GCA_020091425.1 GCA_004310285.1, GCA_016595795.1, GCA_029210495.1, GCA_001580025.1, GCA_001277215.2 GCA_016026875.1, GCA_019042215.1, GCA_018863235.1, GCA_000534275.1, GCA_013409785.1 GCA_900109255.1, GCA_900187085.1, GCA_001042715.1, GCA_014641945.1, GCA_022662535.1 GCA_002872255.1, GCA_029003155.1, GCA_011600945.2, GCA_002261065.1, GCA_014644335.1 GCA_000759025.1, GCA_900110925.1, GCA_014137865.1, GCA_003584135.1, GCA_005298075.1 GCA_020622355.1, GCA_004339085.1, GCA_003234935.1, GCA_009184635.1, GCA_025916425.1 GCA_014645655.1, GCA_014646295.1, GCA_009856865.1, GCA_028561705.1, GCA_003148885.1 GCA_011250635.1, GCA_009604425.1, GCA_008923245.1, GCA_002214645.1, GCA_016598615.1 GCA_900460215.1, GCA_007860075.1, GCA_025118245.1, GCA_020216025.1, GCA_017874715.1 GCA_000242635.3, GCA_001877035.1, GCA_000454725.1, GCA_900116805.1, GCA_003269955.1 GCA_000012865.1, GCA_001698185.1, GCA_014195505.1, GCA_003019965.1, GCA_001650635.1 GCA_015999465.1, GCA_000332215.1, GCA_020844025.1, GCA_004920405.1, GCA_001853485.1 GCA_018336155.1, GCA_014652815.1, GCA_017352115.1, GCA_900187035.1, GCA_013201895.1 GCA_006443095.1, GCA_002804065.1, GCA_000741575.1, GCA_016908975.1, GCA_008728195.1 GCA_024612375.1, GCA_002021755.1, GCA_004216775.1, GCA_003263915.2, GCA_000164135.1 GCA_015694735.1, GCA_001683355.1, GCA_014334055.1, GCA_001751245.1, GCA_014647035.1 GCA_014960965.1, GCA_001664385.1, GCA_007992055.1, GCA_003185655.1, GCA_900142095.1 GCA_018343665.1, GCA_900105335.1, GCA_016236975.1, GCA_002102395.1, GCA_003610775.1 GCA_000347775.1, GCA_000297255.1, GCA_009688985.1, GCA_001046875.1, GCA_007860135.1 GCA_003609635.1, GCA_000525775.1, GCA_000300875.1, GCA_921292975.1, GCA_900119825.1 GCA_003024805.1, GCA_003226255.1, GCA_019711315.1, GCA_003812505.1, GCA_003614235.1 GCA_900114935.1, GCA_009371975.2, GCA_015645385.1, GCA_014852565.2, GCA_003002815.1 GCA_002217925.1, GCA_006716565.1, GCA_000093025.1, GCA_012843165.1, GCA_000016645.1 GCA_019798055.1, GCA_900108945.1, GCA_001404615.1, GCA_019139795.1, GCA_020523925.1 GCA_002205845.1, GCA_002891025.1, GCA_006716575.1, GCA_000172135.1, GCA_000158915.1 GCA_004770045.1, GCA_900184705.1, GCA_902859805.1, GCA_003410055.1, GCA_025148285.1 GCA_001886195.1, GCA_001274895.1, GCA_014643455.1, GCA_013410545.1, GCA_014643375.1 GCA_018881545.1, GCA_002943755.1, GCA_013133775.1, GCA_900176285.1, GCA_002310495.1 GCA_003977665.1, GCA_019204025.1, GCA_014836425.1, GCA_023895975.1, GCA_900112315.1 GCA_002374315.1, GCA_902459535.1, GCA_003151025.1, GCA_016907315.1, GCA_022513635.1 GCA_900215205.1, GCA_019739075.1, GCA_000007245.1, GCA_900094885.1, GCA_007989825.1 GCA_006364615.1, GCA_014931175.1, GCA_001123825.1, GCA_002204915.1, GCA_026797595.1 GCA_002797495.1, GCA_025566985.1, GCA_007860095.1, GCA_014836835.1, GCA_004364155.1 GCA_900459315.1, GCA_020523625.1, GCA_008973485.1, GCA_004359545.1, GCA_007992175.1 GCA_003201605.1, GCA_900109545.1, GCA_001298955.1, GCA_002310795.1, GCA_014636215.1 GCA_012933385.1, GCA_001548275.1, GCA_004801905.1, GCA_014764685.1, GCA_014643595.1 GCA_019931675.1, GCA_900475445.1, GCA_001281485.1, GCA_000953655.1, GCA_002841275.1 GCA_003289965.1, GCA_000943565.1, GCA_018398475.1, GCA_019429605.1, GCA_003344925.1 GCA_003129565.1, GCA_016135805.1, GCA_001050215.2, GCA_019331675.1, GCA_014203555.1 GCA_019930585.1, GCA_002021095.1, GCA_023699965.1, GCA_025567405.1, GCA_000018525.1 GCA_014647215.1, GCA_007747655.1, GCA_014395975.1, GCA_000775595.1, GCA_019204045.1 GCA_006716115.1, GCA_020736845.1, GCA_003014485.1, GCA_016862555.1, GCA_900636925.1 GCA_010731715.1, GCA_004361735.1, GCA_007990545.2, GCA_003254625.1, GCA_011045165.1 GCA_024436175.1, GCA_002749495.1, GCA_003096695.1, GCA_005938225.1, GCA_000024605.1 GCA_001885095.1, GCA_002250625.1, GCA_011068285.1, GCA_001854125.1, GCA_009789075.1 GCA_003609605.1, GCA_022848905.1, GCA_004795735.1, GCA_014202505.1, GCA_900110645.1 GCA_019175485.1, GCA_001281525.1, GCA_004151455.1, GCA_900215465.1, GCA_001456355.1 GCA_002872475.1, GCA_004306155.1, GCA_018598185.1, GCA_014649195.1, GCA_003594915.1 GCA_900100015.1, GCA_003721155.4, GCA_026651605.1, GCA_009755585.1, GCA_020387415.1 GCA_001302565.1, GCA_003576245.1, GCA_900100855.1, GCA_001660045.1, GCA_000238915.2 GCA_021404305.1, GCA_007753095.1, GCA_002954765.1, GCA_023517775.1, GCA_003403095.1 GCA_025567175.1, GCA_013761175.1, GCA_020405345.1, GCA_007747215.1, GCA_014637405.1 GCA_004210275.1, GCA_002936955.1, GCA_000712595.1, GCA_006716305.1, GCA_003385765.1 GCA_900174455.1, GCA_003989135.1, GCA_003626695.1, GCA_002813575.1, GCA_010509575.1 GCA_013410355.1, GCA_001544875.2, GCA_001442745.1, GCA_005886105.2, GCA_900637725.1 GCA_004006435.1, GCA_014836985.1, GCA_009866965.1, GCA_002127965.1, GCA_014652135.1 GCA_900102345.1, GCA_003008535.1, GCA_014610845.1, GCA_002056295.1, GCA_002916695.1 GCA_003968655.1, GCA_002797975.1, GCA_024170465.1, GCA_900111595.1, GCA_017487405.1 GCA_019697375.1, GCA_024172125.1, GCA_018454455.1, GCA_016918765.2, GCA_003096075.1 GCA_002797755.1, GCA_022900255.1, GCA_000018685.1, GCA_007855645.1, GCA_009740395.1 GCA_900445535.1, GCA_003609975.1, GCA_003634965.1, GCA_029030725.1, GCA_009834165.1 GCA_003664185.1, GCA_009738105.1, GCA_003417535.1, GCA_023061135.1, GCA_014145395.1 GCA_000940805.1, GCA_009617585.1, GCA_000018885.1, GCA_028606985.1, GCA_900188025.1 GCA_000737765.1, GCA_018139625.1, GCA_018502505.1, GCA_001611955.1, GCA_007993045.1 GCA_003003125.1, GCA_002795305.1, GCA_008040105.1, GCA_006717095.1, GCA_015244565.1 GCA_900115555.1, GCA_011455495.1, GCA_002101585.1, GCA_002288325.1, GCA_007280555.1 GCA_007830455.1, GCA_016905005.1, GCA_003697345.1, GCA_027563315.1, GCA_009908265.2 GCA_025137375.1, GCA_003148845.1, GCA_018286635.1, GCA_014269185.2, GCA_013201645.1 GCA_014199305.1, GCA_018623035.1, GCA_900461585.1, GCA_013141715.1, GCA_011045095.1 GCA_004122145.1, GCA_902859945.1, GCA_003173695.1, GCA_002835605.1, GCA_009721605.1 GCA_004682015.1, GCA_028023675.1, GCA_007830435.1, GCA_900109495.1, GCA_022059885.1 GCA_900187985.1, GCA_017569325.1, GCA_002909375.1, GCA_002115745.1, GCA_003865135.1 GCA_002072955.1, GCA_001027565.1, GCA_008014745.1, GCA_004366375.1, GCA_900092135.1 GCA_900188445.1, GCA_017573465.1, GCA_900167175.1, GCA_900108085.1, GCA_014306625.1 GCA_023168405.1, GCA_003515775.1, GCA_002803535.1, GCA_001050195.2, GCA_000332515.2 GCA_022179325.1, GCA_013340205.1, GCA_900459125.1, GCA_012976305.1, GCA_017349355.1 GCA_001936335.1, GCA_900176535.1, GCA_000442645.1, GCA_013366855.1, GCA_006788875.1 GCA_900111765.1, GCA_016406085.1, GCA_001437055.1, GCA_012790675.1, GCA_020616615.1 GCA_004104075.1, GCA_003402575.1, GCA_900142035.1, GCA_000260965.1, GCA_004216915.1 GCA_009497075.1, GCA_003217295.1, GCA_008838325.1, GCA_021083665.1, GCA_007990935.1 GCA_000226315.1, GCA_900111755.1, GCA_001938935.1, GCA_008153345.1, GCA_003860545.1 GCA_014644455.1, GCA_003545825.1, GCA_002086485.1, GCA_027921805.1, GCA_900156755.1 GCA_003686955.1, GCA_014655595.1, GCA_018736045.1, GCA_900115005.1, GCA_014696825.1 GCA_000175615.1, GCA_018457175.1, GCA_003121895.1, GCA_003336365.1, GCA_002251085.2 GCA_900129235.1, GCA_009768885.1, GCA_021249385.1, GCA_900142695.1, GCA_000298875.1 GCA_001580015.1, GCA_021404465.1, GCA_022788655.1, GCA_000017565.1, GCA_003386795.1 GCA_016728665.1, GCA_018223345.1, GCA_001306135.1, GCA_000511305.1, GCA_002242685.1 GCA_009762795.1, GCA_900167135.1, GCA_003182275.1, GCA_003046545.1, GCA_000211815.1 GCA_007004095.1, GCA_003931535.1, GCA_021365465.1, GCA_016647595.1, GCA_900113015.1 GCA_004766045.1, GCA_016908855.1, GCA_014636995.1, GCA_022811525.1, GCA_000247605.1 GCA_003149475.2, GCA_003996965.1, GCA_900207575.1, GCA_008386465.1, GCA_003814555.1 GCA_009720775.1, GCA_013409205.1, GCA_003433955.1, GCA_014650295.1, GCA_014192275.1 GCA_003205615.1, GCA_003570725.1, GCA_014131755.1, GCA_000772945.1, GCA_028726025.1 GCA_008362905.1, GCA_900277125.1, GCA_000807775.2, GCA_900142435.1, GCA_016613385.1 GCA_003233655.1, GCA_900103965.1, GCA_021852965.1, GCA_013155465.1, GCA_013249065.1 GCA_009739535.1, GCA_000829825.1, GCA_019331805.1, GCA_003314975.1, GCA_900115765.1 GCA_007990305.1, GCA_900187975.1, GCA_003033885.1, GCA_002285125.1, GCA_000970455.1 GCA_014844295.1, GCA_003387375.1, GCA_005937185.1, GCA_012927105.1, GCA_007301515.1 GCA_004801885.1, GCA_007558815.1, GCA_000224455.2, GCA_003668575.1, GCA_011764505.1 GCA_011170065.1, GCA_002954685.1, GCA_003390395.1, GCA_013249045.1, GCA_003605645.1 GCA_000348725.1, GCA_003113895.1, GCA_009834025.1, GCA_004405045.1, GCA_016632365.1 GCA_001999465.1, GCA_002860355.1, GCA_014649055.1, GCA_022811685.1, GCA_003967575.1 GCA_000262245.1, GCA_000827885.1, GCA_003860345.1, GCA_026410325.1, GCA_003011895.2 GCA_003315775.1, GCA_003258365.1, GCA_029457395.1, GCA_005281455.1, GCA_000722635.1 GCA_900129315.1, GCA_001456025.1, GCA_017357825.1, GCA_027922165.1, GCA_001006005.1 GCA_009017275.1, GCA_010211745.1, GCA_001685415.1, GCA_900168015.1, GCA_014650875.1 GCA_900116905.1, GCA_900157365.1, GCA_003002825.1, GCA_000960975.1, GCA_000969445.1 GCA_000775605.1, GCA_001677885.1, GCA_015207075.1, GCA_003339865.1, GCA_900116115.1 GCA_003932735.1, GCA_013467655.1, GCA_002858935.1, GCA_002980525.1, GCA_900538225.1 GCA_010550695.1, GCA_004341885.1, GCA_000636015.1, GCA_016904955.1, GCA_900102945.1 GCA_014652455.1, GCA_900129865.1, GCA_000479045.1, GCA_003576285.1, GCA_003990185.1 GCA_018760365.1, GCA_009833085.1, GCA_014212125.1, GCA_000160055.1, GCA_014646315.1 GCA_000471645.3, GCA_000949475.1, GCA_001580835.1, GCA_000622385.1, GCA_900102875.1 GCA_014196965.1, GCA_001647695.1, GCA_000196875.2, GCA_013374995.1, GCA_002504085.1 GCA_004803465.1, GCA_000218565.1, GCA_023094245.1, GCA_009695865.1, GCA_017948595.1 GCA_019880305.1, GCA_018499875.1, GCA_017874575.1, GCA_901538355.1, GCA_000723205.1 GCA_014645375.1, GCA_022832545.1, GCA_003515065.1, GCA_000709935.2, GCA_000368805.1 GCA_009827995.1, GCA_016599815.1, GCA_003663795.1, GCA_900475885.1, GCA_003937945.1 GCA_022869785.1, GCA_018555435.2, GCA_018333235.1, GCA_000212915.1, GCA_900129175.1 GCA_013201075.1, GCA_016735615.1, GCA_003696345.1, GCA_003046315.1, GCA_017589465.1 GCA_021261325.1, GCA_001562195.1, GCA_900113125.1, GCA_011761945.1, GCA_900188345.1 GCA_014174305.1, GCA_016774385.1, GCA_013003915.1, GCA_007994135.1, GCA_008274215.1 GCA_004016525.1, GCA_025402875.1, GCA_002973535.1, GCA_009906795.1, GCA_000235585.1 GCA_003258705.1, GCA_000724225.2, GCA_003726035.1, GCA_900119165.1, GCA_029525575.1 GCA_003634215.1, GCA_009902005.1, GCA_003611635.1, GCA_900474605.1, GCA_019049675.1 GCA_016918505.1, GCA_018324105.1, GCA_902859815.1, GCA_002754935.1, GCA_016584425.1 GCA_003384605.1, GCA_023502425.1, GCA_001305575.3, GCA_003315515.1, GCA_003641245.1 GCA_008274625.1, GCA_001676725.1, GCA_000070465.1, GCA_021919345.1, GCA_003892345.1 GCA_011398355.1, GCA_003339505.1, GCA_016862515.1, GCA_004217135.1, GCA_006335145.1 GCA_007632255.1, GCA_014650215.1, GCA_017873955.1, GCA_001953355.1, GCA_001644645.1 GCA_016741775.1, GCA_004745865.1, GCA_000747315.1, GCA_014646035.1, GCA_004359355.1 GCA_001767615.1, GCA_018729455.1, GCA_014144865.1, GCA_022385335.1, GCA_003725695.1 GCA_001468005.1, GCA_014385085.1, GCA_004005565.1, GCA_016415625.1, GCA_009831215.1 GCA_900163825.1, GCA_019222765.1, GCA_001270965.1, GCA_000226565.1, GCA_001644015.1 GCA_000195335.1, GCA_016907355.1, GCA_010682105.1, GCA_000179615.1, GCA_000599985.1 GCA_012273615.1, GCA_009697225.1, GCA_002813245.1, GCA_001874645.1, GCA_000024925.1 GCA_000827125.1, GCA_003054285.1, GCA_900102885.1, GCA_016127255.1, GCA_016458235.1 GCA_000258535.2, GCA_002795805.1, GCA_010669305.1, GCA_016124105.1, GCA_016026735.1 GCA_000024345.1, GCA_003667575.1, GCA_003725795.1, GCA_001010285.1, GCA_002082135.1 GCA_023283825.1, GCA_020320395.1, GCA_001758365.1, GCA_002022145.1, GCA_004087915.1 GCA_025567385.1, GCA_900111145.1, GCA_013389365.1, GCA_900116425.1, GCA_014284365.1 GCA_000341345.1, GCA_003057875.1, GCA_900637985.1, GCA_900112645.1, GCA_007744255.1 GCA_003991425.1, GCA_006542665.1, GCA_001007875.1, GCA_013170785.1, GCA_014649395.1 GCA_002288005.1, GCA_012184425.1, GCA_014117265.1, GCA_903994065.1, GCA_001596795.1 GCA_004310685.1, GCA_900109175.1, GCA_019430905.1, GCA_014196615.1, GCA_000802235.1 GCA_013282035.1, GCA_003096955.1, GCA_000092425.1, GCA_000161995.1, GCA_003315415.1 GCA_007830235.1, GCA_021018765.1, GCA_018501225.1, GCA_007845595.1, GCA_014644295.1 GCA_001889025.1, GCA_023277505.1, GCA_014502795.1, GCA_004104115.1, GCA_003429285.1 GCA_000503895.1, GCA_007990775.1, GCA_009711125.1, GCA_001592965.1, GCA_001753085.1 GCA_024807615.1, GCA_003996985.1, GCA_900182565.1, GCA_022845615.1, GCA_000407365.1 GCA_004366715.1, GCA_002531935.1, GCA_021654135.1, GCA_024753285.1, GCA_004104035.1 GCA_003026215.1, GCA_017352235.1, GCA_900091445.1, GCA_014635185.1, GCA_900143275.1 GCA_004331675.1, GCA_004923255.1, GCA_017498485.1, GCA_004331995.1, GCA_900116565.1 GCA_007744675.1, GCA_000963965.1, GCA_000723425.2, GCA_016907235.1, GCA_017751145.1 GCA_020885975.1, GCA_002953195.1, GCA_014638355.1, GCA_015223355.1, GCA_005601135.1 GCA_025133195.1, GCA_016791605.1, GCA_013409565.1, GCA_001578075.1, GCA_014642655.1 GCA_014646575.1, GCA_000224965.2, GCA_004770415.1, GCA_014648255.1, GCA_903989455.1 GCA_009696005.1, GCA_900683595.1, GCA_008710745.1, GCA_022812045.1, GCA_006716185.1 GCA_028201735.1, GCA_900113325.1, GCA_000831645.3, GCA_004785645.1, GCA_900444945.1 GCA_000833575.1, GCA_012163155.1, GCA_001437425.1, GCA_013347265.1, GCA_007954355.1 GCA_014638915.1, GCA_007858975.2, GCA_003008515.1, GCA_900089455.2, GCA_026184115.1 GCA_900129065.1, GCA_900103345.1, GCA_900129585.1, GCA_016742955.1, GCA_902859965.1 GCA_028869115.1, GCA_020424085.1, GCA_002086695.1, GCA_014191055.1, GCA_016623585.1 GCA_003860405.1, GCA_000445125.1, GCA_019793435.1, GCA_028981765.1, GCA_000012985.1 GCA_017876155.1, GCA_019645835.1, GCA_012849375.1, GCA_000013985.1, GCA_900109955.1 GCA_020519665.1, GCA_004346265.1, GCA_004721035.1, GCA_003950325.1, GCA_008710275.1 GCA_000521485.1, GCA_016860525.1, GCA_029478095.1, GCA_018918285.1, GCA_007830355.1 GCA_001612705.2, GCA_000092205.1, GCA_004310865.1, GCA_900103875.1, GCA_000964595.1 GCA_003574965.1, GCA_014649935.1, GCA_014646135.1, GCA_024220175.1, GCA_027922005.1 GCA_001730295.1, GCA_014596575.1, GCA_015097455.1, GCA_005083985.2, GCA_004343345.1 GCA_000801295.1, GCA_013155545.1, GCA_001542625.2, GCA_014648035.1, GCA_006228385.1 GCA_002283365.1, GCA_017876575.1, GCA_022399565.1, GCA_900101875.1, GCA_009901955.1 GCA_023283985.1, GCA_008180465.1, GCA_012295505.1, GCA_000757425.2, GCA_002973605.1 GCA_004339095.1, GCA_023380245.1, GCA_006494475.1, GCA_022008365.1, GCA_001722335.1 GCA_004403065.1, GCA_003667565.1, GCA_014836945.1, GCA_014647255.1, GCA_028736055.1 GCA_007097245.1, GCA_000271305.1, GCA_028981865.1, GCA_001767685.1, GCA_001653975.1 GCA_004343505.1, GCA_004168365.1, GCA_004348435.1, GCA_016803985.1, GCA_001245615.1 GCA_900475405.1, GCA_002069135.1, GCA_001700985.1, GCA_016908515.1, GCA_900156815.1 GCA_002954645.1, GCA_900106645.1, GCA_004364805.1, GCA_011392115.1, GCA_013307245.1 GCA_000214355.1, GCA_022695515.1, GCA_009708175.1, GCA_014385065.1, GCA_007752135.1 GCA_014642175.1, GCA_001887775.1, GCA_900141815.1, GCA_003752415.1, GCA_008923185.1 GCA_018070075.1, GCA_009707865.1, GCA_002157165.1, GCA_016859375.1, GCA_003355475.1 GCA_000455525.1, GCA_024752535.1, GCA_900104895.1, GCA_022341425.1, GCA_023283775.1 GCA_000826835.2, GCA_009753715.1, GCA_000935025.1, GCA_019343495.1, GCA_000164635.1 GCA_003939025.1, GCA_002563675.1, GCA_000732635.1, GCA_900465055.1, GCA_004340685.1 GCA_900177275.1, GCA_004614165.1, GCA_009299475.1, GCA_900454715.1, GCA_014648015.1 GCA_000168975.1, GCA_003386945.1, GCA_002407485.1, GCA_009496005.1, GCA_900097105.1 GCA_016599635.1, GCA_000590795.1, GCA_000265405.1, GCA_000021865.1, GCA_002119425.1 GCA_000317615.1, GCA_003217775.1, GCA_004362805.1, GCA_001513965.1, GCA_003666245.1 GCA_000802225.1, GCA_003967035.1, GCA_007992355.1, GCA_003711265.1, GCA_001856695.1 GCA_008634025.1, GCA_003966125.1, GCA_022884085.1, GCA_900168125.1, GCA_004403185.1 GCA_023195835.1, GCA_016771845.1, GCA_000006685.1, GCA_000496075.1, GCA_000467125.1 GCA_016861545.1, GCA_900110115.1, GCA_900460175.1, GCA_900113875.1, GCA_006716785.1 GCA_001679665.1, GCA_012971725.1, GCA_004342585.1, GCA_024721975.1, GCA_020687205.1 GCA_014647995.1, GCA_021952865.1, GCA_016100695.1, GCA_004322705.1, GCA_900116705.1 GCA_014946825.2, GCA_003955735.1, GCA_000314935.1, GCA_011765605.1, GCA_900156065.1 GCA_900143185.1, GCA_006363895.1, GCA_004514475.2, GCA_026651875.1, GCA_013178945.1 GCA_003985135.1, GCA_022343685.1, GCA_900106765.1, GCA_001703635.1, GCA_001942495.1 GCA_006539325.1, GCA_019443105.1, GCA_023498005.1, GCA_001767695.1, GCA_900156535.1 GCA_900107455.1, GCA_018531375.1, GCA_009757995.1, GCA_012276755.1, GCA_004352915.1 GCA_900102095.1, GCA_000829315.1, GCA_900638585.1, GCA_008629685.1, GCA_014396165.1 GCA_002117565.1, GCA_025566965.1, GCA_900112745.1, GCA_004331465.1, GCA_013201725.1 GCA_005771535.1, GCA_900155945.1, GCA_013283875.1, GCA_021654575.1, GCA_002934545.1 GCA_002356535.2, GCA_003097315.1, GCA_900100255.1, GCA_003385415.1, GCA_014640655.1 GCA_005938105.1, GCA_012317585.1, GCA_018916625.1, GCA_010728925.1, GCA_900129905.1 GCA_014650955.1, GCA_025211425.1, GCA_013304825.1, GCA_000392435.1, GCA_014190775.1 GCA_914484815.1, GCA_014529945.1, GCA_009827315.1, GCA_003046775.1, GCA_004005815.1 GCA_003950315.1, GCA_011393025.1, GCA_900105815.1, GCA_020171465.1, GCA_004769195.1 GCA_006742785.1, GCA_027920805.1, GCA_900217815.1, GCA_000013705.1, GCA_900104455.1 GCA_001717565.1, GCA_014397255.1, GCA_007991855.1, GCA_012911495.1, GCA_020990565.1 GCA_002975235.1, GCA_002017865.1, GCA_018398395.1, GCA_001051235.2, GCA_014641575.1 GCA_013087625.1, GCA_903819435.1, GCA_018390535.1, GCA_020829495.1, GCA_023283945.1 GCA_018476905.1, GCA_019930665.1, GCA_003585975.1, GCA_003855095.1, GCA_020097615.1 GCA_021412585.1, GCA_001553115.1, GCA_001571775.1, GCA_003634555.1, GCA_020341675.1 GCA_009769205.1, GCA_900060165.1, GCA_001545075.2, GCA_022179185.1, GCA_009831665.3 GCA_014698735.1, GCA_014650555.1, GCA_009762325.1, GCA_011947195.1, GCA_025567345.1 GCA_006517625.1, GCA_004684875.1, GCA_000024845.1, GCA_022701275.1, GCA_002995845.1 GCA_000160695.1, GCA_003932715.1, GCA_014645795.1, GCA_003596405.1, GCA_016126715.1 GCA_016925535.1, GCA_900101745.1, GCA_011207455.1, GCA_014652235.1, GCA_900114965.1 GCA_015234745.1, GCA_025000125.1, GCA_000512295.1, GCA_002257585.1, GCA_014649515.1 GCA_021216675.1, GCA_900111405.1, GCA_014648975.1, GCA_001263755.1, GCA_011759375.1 GCA_014803405.1, GCA_002940065.1, GCA_002019225.1, GCA_024614185.1, GCA_009649705.1 GCA_022870865.1, GCA_000015285.1, GCA_008329925.1, GCA_014651095.1, GCA_018139685.1 GCA_001953175.1, GCA_003122365.1, GCA_024666385.1, GCA_900113805.1, GCA_014773245.1 GCA_009931595.1, GCA_013336795.1, GCA_019145205.1, GCA_020861475.1, GCA_020181435.1 GCA_006861795.1, GCA_014642855.1, GCA_000023865.1, GCA_007991335.1, GCA_000020505.1 GCA_014268885.2, GCA_000949255.1, GCA_900094555.1, GCA_003010935.1, GCA_006152145.1 GCA_900475835.1, GCA_014647015.1, GCA_011759585.1, GCA_016064815.1, GCA_002005305.1 GCA_012939995.1, GCA_013425765.1, GCA_000154785.2, GCA_001280595.1, GCA_014652695.1 GCA_001293165.1, GCA_002093115.1, GCA_003148775.1, GCA_003008425.1, GCA_020883495.1 GCA_014836845.1, GCA_002573965.1, GCA_025144665.1, GCA_022427145.1, GCA_016026695.1 GCA_013377195.1, GCA_000183405.1, GCA_002243645.1, GCA_008124715.1, GCA_004403395.1 GCA_900110875.1, GCA_001858005.1, GCA_009720825.1, GCA_008807375.1, GCA_014201355.1 GCA_001618845.1, GCA_000015565.1, GCA_016907365.1, GCA_025149125.1, GCA_004803875.1 GCA_001742425.1, GCA_013300845.1, GCA_011516765.1, GCA_008693645.1, GCA_009695915.1 GCA_900089955.1, GCA_026547205.1, GCA_001684335.1, GCA_016899755.1, GCA_013373955.1 GCA_003574095.1, GCA_026427595.1, GCA_008124775.1, GCA_017312725.1, GCA_000682755.1 GCA_014200805.1, GCA_000024405.1, GCA_002943415.1, GCA_900141865.1, GCA_014192335.1 GCA_000763515.1, GCA_010726645.1, GCA_000181895.2, GCA_000818395.1, GCA_013357945.1 GCA_027921765.1, GCA_900105065.1, GCA_008629675.1, GCA_014651055.1, GCA_003072065.1 GCA_008868005.1, GCA_003012915.1, GCA_016862635.1, GCA_017874595.1, GCA_000014885.1 GCA_020552705.1, GCA_009711365.1, GCA_009768975.1, GCA_001921215.1, GCA_014268455.2 GCA_003752125.1, GCA_007859935.1, GCA_006715785.1, GCA_001078055.1, GCA_009755225.1 GCA_011927765.1, GCA_900638245.1, GCA_019351405.1, GCA_002994045.1, GCA_001886715.1 GCA_000969395.1, GCA_003116835.1, GCA_002101335.1, GCA_006094275.1, GCA_007830335.1 GCA_003060865.1, GCA_001995985.1, GCA_900130055.1, GCA_000712295.1, GCA_900129575.1 GCA_018531185.1, GCA_900100195.1, GCA_011762025.1, GCA_904830955.1, GCA_900113945.1 GCA_016595525.1, GCA_002504165.1, GCA_010731595.1, GCA_017254845.1, GCA_017921895.1 GCA_026005375.1, GCA_003386095.1, GCA_900637555.1, GCA_014207705.1, GCA_001296145.1 GCA_000022565.1, GCA_000012305.1, GCA_000020725.1, GCA_003933245.1, GCA_900105485.1 GCA_022627575.1, GCA_000787375.1, GCA_000817785.2, GCA_009741375.1, GCA_000013025.1 GCA_002289575.1, GCA_000215955.3, GCA_013106755.1, GCA_019884785.1, GCA_014647595.1 GCA_001507665.1, GCA_023823875.1, GCA_004168035.1, GCA_013912435.1, GCA_003323735.1 GCA_021026375.1, GCA_003611465.1, GCA_017347565.1, GCA_000023465.1, GCA_007992215.1 GCA_000265295.1, GCA_014892695.1, GCA_003096995.1, GCA_009938015.1, GCA_003605435.1 GCA_001870225.1, GCA_019084545.1, GCA_008107585.1, GCA_025311495.1, GCA_004379295.1 GCA_018324255.1, GCA_013266755.1, GCA_014648515.1, GCA_004402235.1, GCA_900100455.1 GCA_900089585.1, GCA_017377745.1, GCA_019285775.1, GCA_018882155.1, GCA_014201655.1 GCA_900475315.1, GCA_021271205.1, GCA_000613185.1, GCA_016587455.1, GCA_900105995.1 GCA_019880385.1, GCA_014653435.1, GCA_016238465.1, GCA_000255555.2, GCA_008630635.1 GCA_011682175.1, GCA_007747995.1, GCA_900110025.1, GCA_004011865.1, GCA_003751225.1 GCA_024397795.1, GCA_003003155.1, GCA_900013535.1, GCA_900101735.1, GCA_900112515.1 GCA_001761465.1, GCA_002259755.1, GCA_008374075.1, GCA_003100395.1, GCA_000376705.1 GCA_013347305.1, GCA_001702115.1, GCA_001998825.1, GCA_000011385.1, GCA_022678625.1 GCA_014649795.1, GCA_002020805.1, GCA_002204815.1, GCA_003259375.1, GCA_001025195.1 GCA_006783905.1, GCA_017315755.1, GCA_004745575.1, GCA_002222595.2, GCA_900100075.1 GCA_001596155.1, GCA_001005215.1, GCA_025998535.1, GCA_008247605.1, GCA_016238445.1 GCA_001307805.2, GCA_000805375.1, GCA_000969415.1, GCA_016653355.1, GCA_002706425.1 GCA_003651225.1, GCA_003862435.1, GCA_014384785.1, GCA_019049695.1, GCA_003123725.1 GCA_007991495.1, GCA_002014765.1, GCA_000015245.1, GCA_009901525.1, GCA_016918785.1 GCA_000828475.1, GCA_007829565.1, GCA_004359195.1, GCA_014207535.1, GCA_019334315.1 GCA_004358675.1, GCA_003143515.1, GCA_008974185.1, GCA_020748465.1, GCA_000565485.1 GCA_000154705.2, GCA_021728535.1, GCA_017052465.1, GCA_017498065.1, GCA_013141695.1 GCA_014217255.1, GCA_002871685.1, GCA_014836335.1, GCA_003966915.1, GCA_009650015.1 GCA_023516615.1, GCA_902859895.1, GCA_018316655.1, GCA_014223975.1, GCA_014651675.1 GCA_013046825.1, GCA_016908145.1, GCA_900187205.1, GCA_018127825.1, GCA_000018105.1 GCA_000317125.1, GCA_009674825.1, GCA_001654855.1, GCA_900631955.1, GCA_019049625.1 GCA_003254475.1, GCA_003987015.1, GCA_023380225.1, GCA_001672295.1, GCA_024362845.1 GCA_003966895.1, GCA_021556615.1, GCA_900187145.1, GCA_009858255.1, GCA_028553765.1 GCA_013601105.1, GCA_021117385.1, GCA_003386235.1, GCA_021432085.1, GCA_004337475.1 GCA_004362745.1, GCA_019797845.1, GCA_023145695.1, GCA_003966755.1, GCA_006539285.1 GCA_000511935.2, GCA_003987475.1, GCA_021117405.1, GCA_002155145.1, GCA_008704935.1 GCA_027214045.1, GCA_000814825.1, GCA_014873945.1, GCA_004004555.2, GCA_002259525.1 GCA_003585765.1, GCA_016755875.1, GCA_900129965.1, GCA_014644555.1, GCA_001664265.1 GCA_009711055.1, GCA_007713735.2, GCA_900114265.1, GCA_004214935.1, GCA_900100735.1 GCA_907164555.1, GCA_004348685.1, GCA_000968175.1, GCA_014489725.1, GCA_015223105.1 GCA_004337705.1, GCA_900114625.1, GCA_009904105.1, GCA_006965445.1, GCA_007954525.1 GCA_011762225.1, GCA_007992075.1, GCA_014646935.1, GCA_002127545.1, GCA_900143285.1 GCA_006517175.1, GCA_001735805.1, GCA_000025305.1, GCA_900177375.1, GCA_014652175.1 GCA_900156105.1, GCA_001723295.1, GCA_018455725.1, GCA_000497445.1, GCA_002088235.1 GCA_003201955.1, GCA_001046895.1, GCA_010730055.1, GCA_008124885.1, GCA_900637955.1 GCA_900129445.1, GCA_015207425.1, GCA_016908255.1, GCA_011806565.1, GCA_006740705.1 GCA_000169175.1, GCA_900172355.1, GCA_009193355.1, GCA_011174775.1, GCA_000331185.2 GCA_900182585.1, GCA_004331735.1, GCA_017638925.1, GCA_000731315.1, GCA_013283745.1 GCA_002814075.1, GCA_023061285.1, GCA_020329435.1, GCA_009735555.1, GCA_001936255.1 GCA_002160355.1, GCA_021462825.1, GCA_020447205.1, GCA_001050295.1, GCA_000023125.1 GCA_016424485.1, GCA_004345005.1, GCA_009296165.1, GCA_002095535.1, GCA_003583765.1 GCA_020171525.1, GCA_012641475.1, GCA_014192395.1, GCA_005217585.1, GCA_011045075.1 GCA_025567465.1, GCA_016881405.1, GCA_001623425.1, GCA_000691605.1, GCA_003752465.1 GCA_010728725.1, GCA_003595235.1, GCA_000200595.1, GCA_902859845.1, GCA_018141485.1 GCA_003838225.1, GCA_900115305.1, GCA_025790165.1, GCA_900105665.1, GCA_009711225.1 GCA_018448785.1, GCA_022179525.1, GCA_009827595.1, GCA_025567315.1, GCA_900167575.1 GCA_003965815.1, GCA_004342915.1, GCA_019623905.1, GCA_900112245.1, GCA_014696315.1 GCA_014773365.1, GCA_011466855.1, GCA_014524625.1, GCA_900000175.1, GCA_900450885.1 GCA_900187015.1, GCA_000184345.2, GCA_900114235.1, GCA_001753205.1, GCA_000647675.1 GCA_000739695.1, GCA_002302495.1, GCA_013166515.1, GCA_001435935.1, GCA_900215625.1 GCA_012910705.2, GCA_026642255.1, GCA_014648575.1, GCA_003668795.1, GCA_900115225.1 GCA_000008325.1, GCA_000321415.2, GCA_020026915.1, GCA_900104135.1, GCA_900101885.1 GCA_001570425.1, GCA_003012745.1, GCA_900103135.1, GCA_028748065.1, GCA_900188145.1 GCA_014650615.1, GCA_900113135.1, GCA_900111425.1, GCA_014635245.1, GCA_900115035.1 GCA_025567305.1, GCA_013372125.1, GCA_900111685.1, GCA_003986975.1, GCA_000764165.1 GCA_000743575.1, GCA_001611975.1, GCA_900106565.1, GCA_010560025.1, GCA_014204795.1 GCA_000833995.1, GCA_901421005.1, GCA_000237825.1, GCA_004348545.1, GCA_016860605.1 GCA_000828615.1, GCA_004102945.1, GCA_014202935.1, GCA_900099775.1, GCA_001704615.3 GCA_900188155.1, GCA_016522075.1, GCA_014204755.1, GCA_015773195.1, GCA_018145875.1 GCA_003028575.1, GCA_014961145.1, GCA_014648875.1, GCA_014268275.3, GCA_003350505.1 GCA_014651115.1, GCA_015234585.1, GCA_004134865.1, GCA_900638135.1, GCA_902859855.1 GCA_900445005.1, GCA_004168285.1, GCA_014203705.1, GCA_900103955.1, GCA_900142735.1 GCA_002075885.1, GCA_010681825.2, GCA_002356295.1, GCA_900110735.1, GCA_001306175.1 GCA_020480685.1, GCA_025567165.1, GCA_008932285.1, GCA_003259935.1, GCA_900109365.1 GCA_016728725.1, GCA_900103225.1, GCA_017301615.1, GCA_000153465.1, GCA_019429525.1 GCA_008876665.1, GCA_024198175.1, GCA_900100795.1, GCA_006363915.1, GCA_003627955.1 GCA_000012885.1, GCA_014646015.1, GCA_005221305.1, GCA_016907895.1, GCA_018326425.1 GCA_022601685.1, GCA_000025225.2, GCA_001651805.1, GCA_023284045.1, GCA_007991475.1 GCA_023017165.1, GCA_021044685.1, GCA_003194565.2, GCA_003600895.1, GCA_016924235.1 GCA_009831415.1, GCA_016863475.1, GCA_012276695.1, GCA_900110245.1, GCA_013366395.1 GCA_900167475.1, GCA_022354085.1, GCA_003812345.1, GCA_003515945.1, GCA_003752005.1 GCA_003687725.1, GCA_900177745.1, GCA_006385165.1, GCA_003795375.1, GCA_900114775.1 GCA_014637175.1, GCA_900109135.1, GCA_014650395.1, GCA_009830105.1, GCA_001909055.1 GCA_014899185.1, GCA_011040385.1, GCA_003730295.1, GCA_014397415.1, GCA_028355655.1 GCA_004770765.1, GCA_007097145.1, GCA_018919265.1, GCA_004342625.1, GCA_014202035.1 GCA_003259525.1, GCA_002245655.1, GCA_900110775.1, GCA_001046635.1, GCA_900091475.1 GCA_013408925.1, GCA_001655245.1, GCA_017357245.1, GCA_001741865.1, GCA_001543325.1 GCA_900103985.1, GCA_014652035.1, GCA_003634775.1, GCA_003634925.1, GCA_000026085.1 GCA_014649855.1, GCA_023713375.1, GCA_900112345.1, GCA_900115195.1, GCA_000740785.1 GCA_002204745.1, GCA_001889605.1, GCA_014174165.1, GCA_000828895.1, GCA_002240035.1 GCA_003194385.1, GCA_900110345.1, GCA_003814005.1, GCA_026650945.1, GCA_014200295.1 GCA_016461705.1, GCA_900452515.1, GCA_900220985.1, GCA_001707755.1, GCA_003013695.1 GCA_012933365.1, GCA_000775285.1, GCA_002841355.1, GCA_003343305.1, GCA_900102465.1 GCA_014198875.1, GCA_003387615.1, GCA_014163495.1, GCA_000143685.1, GCA_001940565.1 GCA_019711435.1, GCA_003259745.1, GCA_002761235.1, GCA_003545925.1, GCA_000025265.1 GCA_001584205.1, GCA_003253775.1, GCA_009696045.1, GCA_004375095.1, GCA_003987145.1 GCA_900167095.1, GCA_025154055.1, GCA_007097285.1, GCA_014207695.1, GCA_021568795.1 GCA_006636215.1, GCA_001514215.1, GCA_003837865.1, GCA_000092125.1, GCA_003204135.1 GCA_011305415.1, GCA_000247715.1, GCA_010729105.1, GCA_900109035.1, GCA_900167365.1 GCA_011382985.1, GCA_009915155.1, GCA_004366815.1, GCA_014646515.1, GCA_011090185.1 GCA_003612055.1, GCA_013409625.1, GCA_014203895.1, GCA_000296835.1, GCA_014196345.1 GCA_002727065.1, GCA_023824315.1, GCA_002951935.1, GCA_000147355.1, GCA_000956465.1 GCA_000800395.1, GCA_021556475.1, GCA_014174275.1, GCA_002086515.1, GCA_001975785.1 GCA_015099595.1, GCA_013520985.1, GCA_003628505.1, GCA_000175195.1, GCA_004009905.1 GCA_003412465.1, GCA_019375475.1, GCA_004564725.1, GCA_012395255.1, GCA_022014495.1 GCA_000980885.2, GCA_003688555.1, GCA_000022005.1, GCA_003344865.1, GCA_900187875.1 GCA_001434555.1, GCA_006861825.1, GCA_013409045.1, GCA_900111135.1, GCA_025567135.1 GCA_020091585.1, GCA_016773205.1, GCA_900108225.1, GCA_001981525.1, GCA_000346065.1 GCA_016919705.1, GCA_003072125.1, GCA_025215155.1, GCA_014268505.2, GCA_006539925.1 GCA_026339895.1, GCA_007858475.1, GCA_003989795.1, GCA_001017655.1, GCA_000442255.1 GCA_021295315.1, GCA_003600645.1, GCA_014651535.1, GCA_003970675.1, GCA_900215335.1 GCA_003675325.1, GCA_001553955.1, GCA_016907915.1, GCA_013166595.1, GCA_003254015.1 GCA_002305855.1, GCA_002216795.1, GCA_013085255.1, GCA_000016785.1, GCA_000186345.1 GCA_002993285.1, GCA_003467385.1, GCA_014236655.1, GCA_014650695.1, GCA_008274695.1 GCA_000183665.1, GCA_000196075.1, GCA_900175965.1, GCA_001278055.1, GCA_001889105.1 GCA_003013395.1, GCA_002906475.1, GCA_000019525.1, GCA_001547735.1, GCA_020002245.1 GCA_004912195.1, GCA_004403465.1, GCA_007988845.1, GCA_012910955.1, GCA_900115595.1 GCA_900445265.1, GCA_017815135.1, GCA_003989825.1, GCA_021343995.1, GCA_021398215.1 GCA_000387765.1, GCA_009363415.1, GCA_012103215.1, GCA_024171955.1, GCA_004216575.1 GCA_000175215.2, GCA_017832095.1, GCA_000513295.1, GCA_000024365.1, GCA_020731525.1 GCA_900156525.1, GCA_902859935.1, GCA_014982745.1, GCA_016584445.1, GCA_021165915.1 GCA_004116975.1, GCA_020463755.1, GCA_024436035.1, GCA_900636885.1, GCA_000409775.1 GCA_002243665.1, GCA_002741055.1, GCA_001747105.1, GCA_003429105.1, GCA_002269385.1 GCA_018333155.1, GCA_001905345.1, GCA_000296695.1, GCA_900659885.1, GCA_000144695.1 GCA_016127275.1, GCA_019890725.1, GCA_024628825.1, GCA_014647055.1, GCA_900102215.1 GCA_000495435.3, GCA_900091425.1, GCA_001468025.1, GCA_019331725.1, GCA_000192745.1 GCA_900104725.1, GCA_019891535.1, GCA_006704125.1, GCA_002858675.1, GCA_019139815.1 GCA_003226325.1, GCA_007558865.1, GCA_004769775.1, GCA_014652775.1, GCA_002287505.1 GCA_006385675.1, GCA_004785795.1, GCA_021412605.1, GCA_021168575.1, GCA_017948675.1 GCA_027625995.1, GCA_000756665.1, GCA_900183975.1, GCA_020618775.1, GCA_014262665.1 GCA_003815615.1, GCA_018206105.1, GCA_014692515.1, GCA_003327285.1, GCA_014635865.1 GCA_002895925.1, GCA_008370345.1, GCA_006007945.1, GCA_002259795.1

**Supplementary Information 1.2.** NCBI RefSeq assembly genome accession numbers for the genomes used to generate Figure 7B

GCF_000022305.1, GCF_003711265.1, GCF_045346035.1, GCF_000764555.1, GCF_003966755.1 GCF_000517405.1, GCF_002813775.1, GCF_022845755.1, GCF_004295125.1, GCF_029919255.1 GCF_000164865.1, GCF_001647695.1, GCF_016592615.1, GCF_000007745.1, GCF_000196515.1 GCF_029030725.1, GCF_936269705.1, GCF_025152405.1, GCF_044591705.1, GCF_000287335.1 GCF_009363895.1, GCF_964275155.1, GCF_964063155.1, GCF_964036635.1, GCF_963970025.1 GCF_963853645.1, GCF_963243745.1, GCF_963243495.1, GCF_963227125.1, GCF_961514395.1 GCF_949794035.1, GCF_949790605.1, GCF_949774925.1, GCF_949769195.1, GCF_946995915.1 GCF_946903285.1, GCF_946888465.1, GCF_940677205.1, GCF_937425535.1, GCF_918378365.1 GCF_914590485.1, GCF_905367715.1, GCF_905331265.2, GCF_904859905.1, GCF_904848165.1 GCF_904830935.1, GCF_903886475.1, GCF_902813185.1, GCF_902810445.1, GCF_902459485.1 GCF_901538355.1, GCF_901538265.1, GCF_901482695.1, GCF_901482605.1, GCF_900683745.1 GCF_900683625.1, GCF_900682675.2, GCF_900660755.1, GCF_900660745.1, GCF_900660685.1 GCF_900660615.1, GCF_900638685.1, GCF_900638655.1, GCF_900638635.1, GCF_900638615.1 GCF_900638485.1, GCF_900638385.1, GCF_900638355.1, GCF_900638305.1, GCF_900638245.1 GCF_900638215.1, GCF_900638135.1, GCF_900638015.1, GCF_900637975.1, GCF_900637955.1 GCF_900637915.1, GCF_900637905.1, GCF_900637895.1, GCF_900637855.1, GCF_900637795.1 GCF_900637755.1, GCF_900637725.1, GCF_900637665.1, GCF_900637655.1, GCF_900637635.1 GCF_900637615.1, GCF_900637575.1, GCF_900637555.1, GCF_900637545.1, GCF_900637515.1 GCF_900637475.1, GCF_900637325.1, GCF_900637305.1, GCF_900637295.1, GCF_900637265.1 GCF_900637235.1, GCF_900637205.1, GCF_900637195.1, GCF_900637185.1, GCF_900637165.1 GCF_900637105.1, GCF_900637075.1, GCF_900637055.1, GCF_900637025.1, GCF_900636985.1 GCF_900636925.1, GCF_900636915.1, GCF_900636885.1, GCF_900636765.1, GCF_900636745.1 GCF_900636575.1, GCF_900636475.1, GCF_900636445.1, GCF_900635955.1, GCF_900635775.1 GCF_900603025.1, GCF_900560965.1, GCF_900537995.1, GCF_900478165.1, GCF_900478135.1 GCF_900478115.1, GCF_900478045.1, GCF_900478035.1, GCF_900477945.1, GCF_900476255.1 GCF_900476215.1, GCF_900476055.1, GCF_900476045.1, GCF_900476035.1, GCF_900476005.1 GCF_900475975.1, GCF_900475945.1, GCF_900475915.1, GCF_900475905.1, GCF_900475885.1 GCF_900475855.1, GCF_900475835.1, GCF_900475675.1, GCF_900475625.1, GCF_900475595.1 GCF_900475555.1, GCF_900475505.1, GCF_900475445.1, GCF_900475415.1, GCF_900475375.1 GCF_900475315.1, GCF_900475285.1, GCF_900475035.1, GCF_900475025.1, GCF_900474615.1 GCF_900474605.1, GCF_900465055.1, GCF_900327255.1, GCF_900324475.1, GCF_900289045.1 GCF_900217235.1, GCF_900198195.1, GCF_900187355.1, GCF_900187315.1, GCF_900187305.1 GCF_900187295.1, GCF_900187285.1, GCF_900187255.1, GCF_900187235.1, GCF_900187215.1 GCF_900187205.1, GCF_900187185.1, GCF_900187175.1, GCF_900187165.1, GCF_900187125.1 GCF_900187105.1, GCF_900187095.1, GCF_900187085.1, GCF_900187075.1, GCF_900187065.1 GCF_900187055.1, GCF_900187045.1, GCF_900187035.1, GCF_900187015.1, GCF_900187005.1 GCF_900186995.1, GCF_900186985.1, GCF_900186965.1, GCF_900186885.1, GCF_900186865.1 GCF_900186835.1, GCF_900184295.1, GCF_900183975.1, GCF_900157305.1, GCF_900149385.2 GCF_900116935.1, GCF_900096765.1, GCF_900095155.1, GCF_900087655.1, GCF_900070355.1 GCF_900048035.1, GCF_900005615.1, GCF_046058945.1, GCF_045833485.1, GCF_045784305.1 GCF_045784255.1, GCF_045689975.1, GCF_045689615.1, GCF_045276965.1, GCF_045209695.1 GCF_045208505.1, GCF_045207185.1, GCF_045162825.1, GCF_045161845.1, GCF_045161815.1 GCF_045160875.1, GCF_045057155.1, GCF_045041415.1, GCF_045040895.1, GCF_045039635.1 GCF_045039555.1, GCF_045002895.1, GCF_044999035.1, GCF_044987285.1, GCF_044872765.1 GCF_044797865.1, GCF_044795995.1, GCF_044790185.1, GCF_044790165.1, GCF_044597075.1 GCF_044501185.1, GCF_044360475.1, GCF_044358485.1, GCF_044095835.1, GCF_044051715.1 GCF_043632995.1, GCF_043587585.1, GCF_043575855.1, GCF_043228865.1, GCF_043186315.1 GCF_042926695.1, GCF_042854845.1, GCF_042852765.1, GCF_042846945.1, GCF_042751225.1 GCF_042691665.1, GCF_042466515.1, GCF_041897805.1, GCF_041893865.1, GCF_041892135.1 GCF_041549525.1, GCF_041519315.1, GCF_041519305.1, GCF_041504375.1, GCF_041475515.1 GCF_041464075.1, GCF_041429785.1, GCF_041428895.1, GCF_041428845.1, GCF_041428715.1 GCF_041428605.1, GCF_041295575.1, GCF_041228765.1, GCF_041227765.1, GCF_041222865.1 GCF_041021905.1, GCF_040959045.1, GCF_040869025.1, GCF_040822035.1, GCF_040790665.1 GCF_040746085.1, GCF_040739365.1, GCF_040687965.1, GCF_040529065.1, GCF_040438935.1 GCF_040409965.1, GCF_040267685.1, GCF_040267515.1, GCF_040256395.1, GCF_040235295.1 GCF_040215475.1, GCF_040105105.1, GCF_039789245.1, GCF_039724765.1, GCF_039654145.1 GCF_039636765.1, GCF_039634495.1, GCF_039555305.1, GCF_039555295.1, GCF_039519175.1 GCF_038784545.1, GCF_038725785.1, GCF_038723655.1, GCF_038500265.1, GCF_038452265.1 GCF_038396635.1, GCF_038368255.1, GCF_038086725.1, GCF_038024855.1, GCF_038024125.1 GCF_038024105.1, GCF_037935975.1, GCF_037892375.1, GCF_037818705.1, GCF_037811135.1 GCF_037478105.1, GCF_037478055.1, GCF_037478035.1, GCF_037477945.1, GCF_037414505.1 GCF_037333485.1, GCF_037290055.1, GCF_037201985.1, GCF_037126205.1, GCF_037113525.1 GCF_037099765.1, GCF_037081835.1, GCF_037076345.1, GCF_037060685.1, GCF_037055245.1 GCF_037044555.1, GCF_037023865.1, GCF_036894225.1, GCF_036885195.1, GCF_036884255.1 GCF_036864995.1, GCF_036840915.1, GCF_036812735.1, GCF_036700025.1, GCF_036700015.1 GCF_036689625.1, GCF_036689615.1, GCF_036585405.1, GCF_036511535.1, GCF_036492835.1 GCF_036492635.1, GCF_036492425.1, GCF_036492245.1, GCF_036488675.1, GCF_036352035.1 GCF_036346855.1, GCF_036345975.1, GCF_036345035.1, GCF_036327715.1, GCF_036323495.1 GCF_036323475.1, GCF_036322735.1, GCF_036320655.1, GCF_036320555.1, GCF_036287475.1 GCF_036250755.1, GCF_036250655.1, GCF_036227585.1, GCF_036208045.1, GCF_036204065.1 GCF_036189845.1, GCF_036178885.1, GCF_036177905.1, GCF_036172715.1, GCF_036170895.1 GCF_036142455.1, GCF_036073155.1, GCF_036051655.1, GCF_036041405.1, GCF_036035005.1 GCF_036014075.1, GCF_036010405.1, GCF_035990065.1, GCF_035957715.1, GCF_035930445.1 GCF_035918135.1, GCF_035918035.1, GCF_035917655.1, GCF_035917595.1, GCF_035917475.1 GCF_035905475.1, GCF_035747985.1, GCF_035231985.1, GCF_034627025.1, GCF_034555895.2 GCF_034554815.1, GCF_034508895.1, GCF_034479635.1, GCF_034479615.1, GCF_034479555.1 GCF_034479515.1, GCF_034479305.1, GCF_034478925.1, GCF_034424665.1, GCF_034424645.1 GCF_034424565.1, GCF_034424545.1, GCF_034424315.1, GCF_034424215.1, GCF_034421895.1 GCF_034376045.1, GCF_034375205.1, GCF_034356035.1, GCF_034355335.1, GCF_034298135.1 GCF_034262375.1, GCF_034259205.1, GCF_034055775.1, GCF_034055115.1, GCF_034049535.1 GCF_034047095.1, GCF_034011435.1, GCF_033969665.1, GCF_033956065.1, GCF_033955345.1 GCF_033954835.1, GCF_033807855.1, GCF_033723955.1, GCF_033547155.1, GCF_033547095.1 GCF_033547035.1, GCF_033406955.1, GCF_033126985.1, GCF_033109485.1, GCF_033106145.1 GCF_033097365.1, GCF_033096465.1, GCF_032924845.1, GCF_032923285.1, GCF_032922765.1 GCF_032879645.1, GCF_032850805.1, GCF_032810065.1, GCF_032806925.1, GCF_032698475.1 GCF_032164335.1, GCF_032108445.1, GCF_031593255.1, GCF_031348265.1, GCF_031297465.1 GCF_031202285.1, GCF_031199375.1, GCF_031191545.1, GCF_031172035.1, GCF_030980095.1 GCF_030864025.1, GCF_030863865.1, GCF_030719275.1, GCF_030718845.1, GCF_030644225.1 GCF_030644205.1, GCF_030585465.1, GCF_030553095.1, GCF_030518655.1, GCF_030517105.1 GCF_030506325.1, GCF_030506305.1, GCF_030505655.1, GCF_030445085.1, GCF_030440595.1 GCF_030440575.1, GCF_030440555.1, GCF_030440535.1, GCF_030440365.1, GCF_030440315.2 GCF_030439775.1, GCF_030439705.1, GCF_030439285.1, GCF_030438475.1, GCF_030438365.1 GCF_030438115.1, GCF_030437945.1, GCF_030437705.1, GCF_030436445.1, GCF_030436185.1 GCF_030414215.1, GCF_030412785.1, GCF_030408855.1, GCF_030408815.1, GCF_030408795.1 GCF_030408755.1, GCF_030408715.1, GCF_030408675.1, GCF_030408595.1, GCF_030408575.1 GCF_030408435.1, GCF_030408415.1, GCF_030408395.1, GCF_030408375.1, GCF_030406665.1 GCF_030376725.1, GCF_030370465.1, GCF_030369635.1, GCF_030344935.1, GCF_030323865.1 GCF_030297255.1, GCF_030296935.1, GCF_030296755.1, GCF_030296615.1, GCF_030296595.1 GCF_030296575.1, GCF_030295955.1, GCF_030295935.1, GCF_030295765.1, GCF_030295685.1 GCF_030295285.1, GCF_030294945.1, GCF_030294405.1, GCF_030291735.1, GCF_030291635.1 GCF_030285665.1, GCF_030272285.1, GCF_030253495.1, GCF_030246965.1, GCF_030144325.1 GCF_030123245.1, GCF_030122845.1, GCF_030064585.1, GCF_030060435.1, GCF_030053895.1 GCF_030035425.1, GCF_030016155.2, GCF_029982035.1, GCF_029961225.1, GCF_029909795.1 GCF_029892065.1, GCF_029892045.1, GCF_029889745.1, GCF_029866925.1, GCF_029854435.1 GCF_029854215.1, GCF_029853775.1, GCF_029774255.1, GCF_029774155.1, GCF_029714545.1 GCF_029714365.1, GCF_029714185.1, GCF_029632925.1, GCF_029625475.1, GCF_029625435.1 GCF_029625335.1, GCF_029541605.1, GCF_029457455.1, GCF_029457395.1, GCF_029318685.1 GCF_029223865.1, GCF_029215875.1, GCF_029094545.1, GCF_029028125.1, GCF_029026745.1 GCF_029026145.1, GCF_029025825.1, GCF_029024945.1, GCF_029023685.1, GCF_029011865.1 GCF_028891345.1, GCF_028885395.1, GCF_028868935.1, GCF_028768405.1, GCF_028751525.1 GCF_028751155.1, GCF_028751035.1, GCF_028747985.1, GCF_028743515.2, GCF_028743495.1 GCF_028736055.1, GCF_028735875.1, GCF_028657945.1, GCF_028622335.1, GCF_028622095.1 GCF_028609885.1, GCF_028609825.1, GCF_028609805.1, GCF_028596125.1, GCF_028596105.1 GCF_028596025.1, GCF_028553805.1, GCF_028553785.1, GCF_028553765.1, GCF_028553745.1 GCF_028553725.1, GCF_028532485.1, GCF_028473685.1, GCF_028421465.1, GCF_028355655.1 GCF_027947595.1, GCF_027947575.1, GCF_027946175.1, GCF_027942015.1, GCF_027925445.1 GCF_027924785.1, GCF_027923905.1, GCF_027920505.1, GCF_027886705.1, GCF_027626975.1 GCF_027625995.1, GCF_027571405.1, GCF_027497495.1, GCF_027497475.1, GCF_027474845.2 GCF_027474505.1, GCF_027286365.1, GCF_027270315.1, GCF_027118995.1, GCF_026914285.1 GCF_026723765.1, GCF_026651875.1, GCF_026650945.1, GCF_026572315.1, GCF_026427415.1 GCF_026248825.1, GCF_026104315.1, GCF_026013905.1, GCF_026013645.1, GCF_026000375.1 GCF_025999835.1, GCF_025998455.1, GCF_025997855.1, GCF_025985205.1, GCF_025908395.1 GCF_025758395.1, GCF_025758125.1, GCF_025722975.1, GCF_025643595.1, GCF_025583665.1 GCF_025562755.1, GCF_025561465.1, GCF_025560845.1, GCF_025558845.1, GCF_025558825.1 GCF_025558325.1, GCF_025452235.1, GCF_025311515.1, GCF_025311495.1, GCF_025311475.1 GCF_025264705.1, GCF_025264685.1, GCF_025264625.1, GCF_025231465.1, GCF_025215495.1 GCF_025200905.1, GCF_025200885.1, GCF_025200715.1, GCF_025200655.1, GCF_025152575.1 GCF_025151995.1, GCF_025151385.1, GCF_025151045.1, GCF_025150245.1, GCF_025150085.1 GCF_025149915.1, GCF_025149785.1, GCF_025149625.1, GCF_025149465.1, GCF_025149285.1 GCF_025149125.1, GCF_025148965.1, GCF_025148785.1, GCF_025148635.1, GCF_025148445.1 GCF_025148285.1, GCF_025148125.1, GCF_025147765.1, GCF_025146565.1, GCF_025145845.1 GCF_025144995.1, GCF_025144665.1, GCF_025144545.1, GCF_025139665.1, GCF_025137635.1 GCF_025137375.1, GCF_025133285.1, GCF_025118245.1, GCF_024971755.1, GCF_024927925.1 GCF_024925425.1, GCF_024918935.1, GCF_024752535.1, GCF_024746855.1, GCF_024734405.1 GCF_024638035.1, GCF_024628825.1, GCF_024584745.1, GCF_024584605.1, GCF_024584585.1 GCF_024453835.1, GCF_024453815.1, GCF_024401155.1, GCF_024397795.1, GCF_024362345.1 GCF_024362265.1, GCF_024349785.1, GCF_024347655.1, GCF_024347635.1, GCF_024347595.1 GCF_024347575.1, GCF_024347535.1, GCF_024347495.1, GCF_024347475.1, GCF_024347415.1 GCF_024347355.1, GCF_024347315.1, GCF_024347295.1, GCF_024347275.1, GCF_024347255.1 GCF_024347115.1, GCF_024347095.1, GCF_024347075.1, GCF_024347055.1, GCF_024347015.1 GCF_024346975.1, GCF_024346955.1, GCF_024346935.1, GCF_024346875.1, GCF_024346835.1 GCF_024346795.1, GCF_024346755.1, GCF_024346715.1, GCF_024346675.1, GCF_024299005.1 GCF_024297005.1, GCF_024224615.1, GCF_024206795.1, GCF_024205945.1, GCF_024181585.1 GCF_024134545.1, GCF_023935825.1, GCF_023923245.1, GCF_023921225.1, GCF_023920225.1 GCF_023920085.1, GCF_023733635.1, GCF_023700225.1, GCF_023699965.1, GCF_023653455.2 GCF_023650915.1, GCF_023573145.1, GCF_023520795.1, GCF_023518055.1, GCF_023498005.1 GCF_023373785.1, GCF_023347705.1, GCF_023299185.1, GCF_023278185.1, GCF_023278125.1 GCF_023277505.1, GCF_023238325.1, GCF_023208015.1, GCF_023195815.2, GCF_023195735.1 GCF_023035795.1, GCF_023035295.1, GCF_023023125.1, GCF_023008305.1, GCF_022984195.1 GCF_022879815.1, GCF_022879615.1, GCF_022871045.2, GCF_022871025.1, GCF_022870945.1 GCF_022870865.1, GCF_022870845.1, GCF_022869645.1, GCF_022869165.1, GCF_022846375.1 GCF_022846135.1, GCF_022845615.1, GCF_022836895.1, GCF_022827545.1, GCF_022812045.1 GCF_022811605.1, GCF_022811565.1, GCF_022809775.1, GCF_022788655.1, GCF_022749515.1 GCF_022749495.1, GCF_022701015.1, GCF_022674245.1, GCF_022647325.1, GCF_022637515.1 GCF_022637495.1, GCF_022592395.1, GCF_022558445.1, GCF_022533465.1, GCF_022493915.1 GCF_022453685.1, GCF_022430545.2, GCF_022430525.1, GCF_022394675.1, GCF_022385335.1 GCF_022374895.2, GCF_022374875.2, GCF_022371215.1, GCF_022370835.2, GCF_022370755.2 GCF_022370635.2, GCF_022370415.1, GCF_022369495.1, GCF_022354085.1, GCF_022343725.1 GCF_022179545.1, GCF_022175585.2, GCF_022170785.1, GCF_022059885.1, GCF_022024335.1 GCF_022024215.1, GCF_022014715.1, GCF_021919345.1, GCF_021733145.1, GCF_021654795.1 GCF_021654775.1, GCF_021650975.2, GCF_021650935.1, GCF_021545825.1, GCF_021496385.1 GCF_021462285.1, GCF_021441905.1, GCF_021432085.1, GCF_021391495.1, GCF_021391315.1 GCF_021378605.1, GCF_021365465.1, GCF_021278985.1, GCF_021249385.1, GCF_021228795.1 GCF_021216655.1, GCF_021184025.1, GCF_021183565.1, GCF_021172025.1, GCF_021165935.1 GCF_021049305.1, GCF_021044685.1, GCF_020991125.1, GCF_020991025.1, GCF_020917325.1 GCF_020911725.1, GCF_020906275.1, GCF_020889625.1, GCF_020886775.1, GCF_020886175.1 GCF_020883495.1, GCF_020872015.1, GCF_020810675.1, GCF_020783575.1, GCF_020783375.1 GCF_020783335.1, GCF_020783315.1, GCF_020736045.1, GCF_020736005.1, GCF_020735985.1 GCF_020735925.1, GCF_020735545.1, GCF_020735445.1, GCF_020683125.1, GCF_020546685.1 GCF_020542785.1, GCF_020541305.1, GCF_020541265.1, GCF_020510565.1, GCF_020510525.1 GCF_020510245.1, GCF_020423125.1, GCF_020422925.2, GCF_020406815.2, GCF_020310025.1 GCF_020310005.1, GCF_020215645.1, GCF_020181435.1, GCF_020162295.1, GCF_020162115.1 GCF_020150375.1, GCF_020149575.1, GCF_020099395.1, GCF_020099335.1, GCF_020099275.1 GCF_020097475.1, GCF_020097375.1, GCF_020097295.1, GCF_020091505.1, GCF_020091425.1 GCF_020080045.1, GCF_020079945.1, GCF_019977755.1, GCF_019977655.1, GCF_019933235.1 GCF_019931045.1, GCF_019931005.1, GCF_019930925.1, GCF_019930785.1, GCF_019930665.1 GCF_019930545.1, GCF_019916025.1, GCF_019904175.1, GCF_019891395.1, GCF_019890955.1 GCF_019884785.1, GCF_019856495.1, GCF_019844095.1, GCF_019797925.1, GCF_019797805.1 GCF_019774635.1, GCF_019739075.1, GCF_019704535.1, GCF_019670485.1, GCF_019670105.1 GCF_019669905.1, GCF_019668505.1, GCF_019645875.1, GCF_019645855.1, GCF_019645835.1 GCF_019645815.1, GCF_019603355.1, GCF_019602855.1, GCF_019575995.1, GCF_019552005.1 GCF_019456675.1, GCF_019456655.1, GCF_019453995.1, GCF_019397265.1, GCF_019396925.1 GCF_019395145.1, GCF_019357495.1, GCF_019343495.1, GCF_019343125.1, GCF_019334125.1 GCF_019285775.1, GCF_019272935.1, GCF_019222765.1, GCF_019175485.1, GCF_019141545.1 GCF_019090985.1, GCF_019048645.1, GCF_019048385.1, GCF_019048245.1, GCF_019048165.1 GCF_019048125.1, GCF_019048105.1, GCF_019048065.1, GCF_019047805.1, GCF_019047465.1 GCF_019046945.1, GCF_018972165.1, GCF_018885085.1, GCF_018802645.1, GCF_018802605.1 GCF_018739485.1, GCF_018736065.1, GCF_018736045.1, GCF_018734325.1, GCF_018604565.1 GCF_018604145.1, GCF_018582665.1, GCF_018502505.1, GCF_018459925.1, GCF_018409545.1 GCF_018398935.1, GCF_018394375.1, GCF_018394055.1, GCF_018389705.1, GCF_018343775.1 GCF_018336855.1, GCF_018326425.1, GCF_018324685.1, GCF_018324255.1, GCF_018324205.1 GCF_018324105.1, GCF_018314255.1, GCF_018292205.1, GCF_018292165.1, GCF_018292125.1 GCF_018289135.1, GCF_018279895.1, GCF_018279705.1, GCF_018278905.1, GCF_018223745.1 GCF_018141425.1, GCF_018139125.1, GCF_018135955.1, GCF_018128425.1, GCF_018128265.1 GCF_018128205.1, GCF_018128125.1, GCF_018127985.1, GCF_018127825.1, GCF_018127785.1 GCF_018127765.1, GCF_018127725.1, GCF_018075365.1, GCF_018069625.1, GCF_018064205.1 GCF_017948405.1, GCF_017948325.1, GCF_017901175.1, GCF_017894385.1, GCF_017894365.1 GCF_017894345.1, GCF_017894325.1, GCF_017893965.1, GCF_017815575.1, GCF_017753665.1 GCF_017751225.1, GCF_017743015.1, GCF_017742995.1, GCF_017724035.1, GCF_017639205.1 GCF_017607425.1, GCF_017569325.2, GCF_017526105.1, GCF_017498685.1, GCF_017498585.1 GCF_017488845.2, GCF_017377395.1, GCF_017377355.1, GCF_017357445.1, GCF_017357245.1 GCF_017357225.1, GCF_017352235.1, GCF_017352135.1, GCF_017348895.1, GCF_017329545.1 GCF_017310015.1, GCF_017309605.1, GCF_017301775.1, GCF_017167985.1, GCF_017161265.1 GCF_017068375.1, GCF_017068355.1, GCF_016939435.1, GCF_016917755.1, GCF_016906185.1 GCF_016906065.1, GCF_016894385.1, GCF_016894325.1, GCF_016890085.1, GCF_016889465.1 GCF_016889425.1, GCF_016889385.1, GCF_016888945.1, GCF_016865425.1, GCF_016864595.1 GCF_016861545.1, GCF_016858125.1, GCF_016834455.1, GCF_016804345.1, GCF_016804005.1 GCF_016801755.1, GCF_016767175.1, GCF_016757795.1, GCF_016747875.1, GCF_016728825.1 GCF_016728785.1, GCF_016728665.1, GCF_016728365.1, GCF_016728105.1, GCF_016726985.1 GCF_016726485.1, GCF_016726365.1, GCF_016725645.1, GCF_016725245.1, GCF_016725005.1 GCF_016724865.1, GCF_016724805.1, GCF_016724785.1, GCF_016698705.1, GCF_016647595.1 GCF_016605985.1, GCF_016599815.1, GCF_016599795.1, GCF_016599635.1, GCF_016598775.1 GCF_016592595.1, GCF_016592575.1, GCF_016584445.1, GCF_016576965.1, GCF_016406325.1 GCF_016250455.1, GCF_016128195.1, GCF_016128095.1, GCF_016127995.1, GCF_016127955.1 GCF_016127855.1, GCF_016127715.1, GCF_016127495.1, GCF_016127475.1, GCF_016127455.1 GCF_016127355.1, GCF_016127275.1, GCF_016127255.1, GCF_016127215.1, GCF_016127195.1 GCF_016028855.1, GCF_016028795.1, GCF_016028775.1, GCF_016028735.1, GCF_016028295.1 GCF_016028275.1, GCF_016027855.1, GCF_016027415.1, GCF_016027375.1, GCF_016027095.1 GCF_016026755.1, GCF_016026735.1, GCF_016026695.1, GCF_016026635.1, GCF_016026615.1 GCF_016026575.1, GCF_016026395.1, GCF_015999465.1, GCF_015710995.1, GCF_015689495.1 GCF_015689395.1, GCF_015689195.1, GCF_015624505.1, GCF_015476275.1, GCF_015476235.1 GCF_015377145.2, GCF_015326725.1, GCF_015326295.1, GCF_015291705.1, GCF_015244315.1 GCF_015160875.1, GCF_015139575.1, GCF_015099595.1, GCF_015074805.1, GCF_014961145.1 GCF_014905135.1, GCF_014898195.1, GCF_014892695.1, GCF_014879295.1, GCF_014844295.1 GCF_014843995.1, GCF_014789145.1, GCF_014779555.2, GCF_014770185.1, GCF_014725695.1 GCF_014705945.2, GCF_014705925.2, GCF_014701115.1, GCF_014701095.1, GCF_014697215.1 GCF_014697095.1, GCF_014681765.1, GCF_014680085.1, GCF_014673495.1, GCF_014672695.1 GCF_014656585.1, GCF_014623465.1, GCF_014495845.1, GCF_014489535.1, GCF_014489515.1 GCF_014466955.1, GCF_014397415.1, GCF_014397255.1, GCF_014397115.1, GCF_014396385.1 GCF_014396165.1, GCF_014395785.1, GCF_014395425.1, GCF_014395225.1, GCF_014269225.2 GCF_014269025.2, GCF_014268695.2, GCF_014236795.1, GCF_014235185.1, GCF_014218335.1 GCF_014218275.1, GCF_014217765.1, GCF_014216335.1, GCF_014211955.1, GCF_014191545.1 GCF_014171495.1, GCF_014131795.1, GCF_014131755.1, GCF_014109845.1, GCF_014076555.1 GCF_014070455.1, GCF_014070435.1, GCF_014058685.1, GCF_014055025.1, GCF_014055005.1 GCF_014054985.1, GCF_014054965.1, GCF_014054885.1, GCF_014054725.1, GCF_013752735.1 GCF_013488225.1, GCF_013488205.1, GCF_013488025.1, GCF_013423865.1, GCF_013402795.1 GCF_013394065.1, GCF_013394005.1, GCF_013389765.1, GCF_013388375.1, GCF_013388295.1 GCF_013377995.1, GCF_013377295.1, GCF_013377235.1, GCF_013377195.1, GCF_013376455.1 GCF_013374215.1, GCF_013372285.1, GCF_013372265.1, GCF_013372245.1, GCF_013372225.1 GCF_013372205.1, GCF_013372165.1, GCF_013372125.1, GCF_013372045.1, GCF_013368775.1 GCF_013364315.1, GCF_013364095.1, GCF_013347325.1, GCF_013347305.1, GCF_013347285.1 GCF_013347265.1, GCF_013343195.2, GCF_013342985.1, GCF_013340845.1, GCF_013318015.2 GCF_013285525.1, GCF_013285305.1, GCF_013282725.1, GCF_013282215.1, GCF_013267695.1 GCF_013267435.1, GCF_013267415.1, GCF_013267395.1, GCF_013201935.1, GCF_013201725.1 GCF_013201665.1, GCF_013177655.1, GCF_013127755.1, GCF_013085545.1, GCF_013046825.1 GCF_013030075.1, GCF_013009555.1, GCF_013004005.1, GCF_012971725.1, GCF_012913625.1 GCF_012648005.1, GCF_012647205.1, GCF_012584515.1, GCF_012516395.1, GCF_012295615.1 GCF_012295595.1, GCF_012276695.1, GCF_012225885.1, GCF_012222965.1, GCF_012222825.1 GCF_012029655.1, GCF_011801455.1, GCF_011765605.1, GCF_011764565.1, GCF_011617105.1 GCF_011611525.1, GCF_011600945.2, GCF_011466855.1, GCF_011455875.1, GCF_011455495.1 GCF_011399095.1, GCF_011397855.1, GCF_011067105.1, GCF_011066545.1, GCF_011058775.1 GCF_011046975.1, GCF_011046895.1, GCF_011046555.1, GCF_011046245.1, GCF_011045835.1 GCF_011044475.1, GCF_011040435.1, GCF_010918895.1, GCF_010731835.1, GCF_010731815.2 GCF_010731795.1, GCF_010731775.1, GCF_010731755.1, GCF_010731735.1, GCF_010731715.1 GCF_010731695.1, GCF_010731655.1, GCF_010731635.1, GCF_010731615.1, GCF_010731595.1 GCF_010731575.1, GCF_010731535.1, GCF_010731295.1, GCF_010731115.1, GCF_010730955.1 GCF_010730745.1, GCF_010730575.1, GCF_010730355.1, GCF_010730195.1, GCF_010730055.1 GCF_010729895.1, GCF_010729665.1, GCF_010729485.1, GCF_010729305.1, GCF_010729105.1 GCF_010728925.1, GCF_010728725.1, GCF_010728525.1, GCF_010728325.1, GCF_010728155.1 GCF_010727945.1, GCF_010727605.1, GCF_010727475.1, GCF_010727325.1, GCF_010727125.1 GCF_010726955.1, GCF_010726765.1, GCF_010726645.1, GCF_010726245.1, GCF_010726085.1 GCF_010725885.1, GCF_010725725.1, GCF_010725485.1, GCF_010669305.1, GCF_010669245.1 GCF_010669225.1, GCF_010669205.1, GCF_010509575.1, GCF_010509075.1, GCF_010508875.1 GCF_010450915.1, GCF_010450895.1, GCF_010450875.1, GCF_009938225.1, GCF_009938015.1 GCF_009936175.1, GCF_009936155.1, GCF_009933595.1, GCF_009931595.1, GCF_009931295.1 GCF_009931115.1, GCF_009930795.1, GCF_009914475.1, GCF_009914215.2, GCF_009913655.1 GCF_009911755.1, GCF_009905255.1, GCF_009901525.1, GCF_009884975.1, GCF_009883735.1 GCF_009873295.1, GCF_009856625.1, GCF_009856605.1, GCF_009832765.1, GCF_009831375.1 GCF_009796305.1, GCF_009789595.1, GCF_009789575.1, GCF_009769205.1, GCF_009769165.1 GCF_009755645.1, GCF_009755585.1, GCF_009739535.1, GCF_009734005.1, GCF_009731575.1 GCF_009730595.1, GCF_009730395.1, GCF_009730295.1, GCF_009730055.1, GCF_009708955.1 GCF_009688985.1, GCF_009688965.1, GCF_009688945.1, GCF_009684715.1, GCF_009676365.1 GCF_009664085.1, GCF_009662475.1, GCF_009650135.1, GCF_009649955.1, GCF_009649915.1 GCF_009648935.1, GCF_009646115.1, GCF_009586235.1, GCF_009498035.1, GCF_009429125.1 GCF_009428965.1, GCF_009363155.1, GCF_009363135.1, GCF_009363115.1, GCF_009363095.1 GCF_009362255.1, GCF_009301415.1, GCF_009299385.1, GCF_009184705.1, GCF_009183365.2 GCF_009035845.1, GCF_009025875.1, GCF_008932225.1, GCF_008932115.1, GCF_008931805.1 GCF_008876665.1, GCF_008831485.1, GCF_008831385.1, GCF_008824185.1, GCF_008807015.1 GCF_008806995.1, GCF_008803015.1, GCF_008801925.2, GCF_008728195.1, GCF_008705175.1 GCF_008705135.1, GCF_008704995.1, GCF_008704935.1, GCF_008704855.1, GCF_008704795.1 GCF_008704715.1, GCF_008704555.1, GCF_008704535.1, GCF_008704515.1, GCF_008704495.1 GCF_008704445.1, GCF_008704395.1, GCF_008694105.1, GCF_008693965.1, GCF_008693705.1 GCF_008693645.1, GCF_008639345.1, GCF_008639165.1, GCF_008630635.1, GCF_008370835.2 GCF_008370715.1, GCF_008369725.1, GCF_008369605.1, GCF_008330085.1, GCF_008329945.1 GCF_008329925.1, GCF_008327825.1, GCF_008298035.1, GCF_008274825.1, GCF_008247605.1 GCF_008245045.1, GCF_008245025.1, GCF_008245005.1, GCF_008244765.1, GCF_008153345.1 GCF_008151785.1, GCF_008087665.1, GCF_008087625.1, GCF_008086545.1, GCF_008065135.1 GCF_008033155.1, GCF_008033135.1, GCF_007990635.1, GCF_007990545.2, GCF_007990525.1 GCF_007990505.1, GCF_007990365.1, GCF_007989425.1, GCF_007971685.1, GCF_007971525.1 GCF_007971385.1, GCF_007971025.1, GCF_007970805.1, GCF_007970665.1, GCF_007970465.1 GCF_007954785.1, GCF_007954605.1, GCF_007954525.1, GCF_007954425.1, GCF_007904085.1 GCF_007859655.1, GCF_007858975.2, GCF_007814525.1, GCF_007795095.1, GCF_007641255.1 GCF_007567505.1, GCF_007475525.1, GCF_007362295.1, GCF_007361795.1, GCF_007035805.1 GCF_006874765.1, GCF_006740045.1, GCF_006738645.1, GCF_006716135.1, GCF_006711645.1 GCF_006542355.1, GCF_006542335.1, GCF_006494775.1, GCF_006459125.1, GCF_006364355.1 GCF_006337125.1, GCF_006151905.1, GCF_006094455.1, GCF_006094395.1, GCF_006094375.1 GCF_006094275.1, GCF_006007945.1, GCF_005952805.1, GCF_005931095.1, GCF_005890135.1 GCF_005886755.1, GCF_005886435.1, GCF_005877035.1, GCF_005845365.1, GCF_005843985.1 GCF_005671335.1, GCF_005670685.2, GCF_005519465.1, GCF_005484965.1, GCF_005377625.1 GCF_005280655.1, GCF_005280315.1, GCF_005222225.1, GCF_005221305.1, GCF_005221285.1 GCF_005144905.1, GCF_005144425.2, GCF_004924335.1, GCF_004843545.1, GCF_004803895.1 GCF_004803835.1, GCF_004803815.1, GCF_004803795.1, GCF_004802635.2, GCF_004792415.1 GCF_004786015.1, GCF_004771075.1, GCF_004768745.1, GCF_004768525.1, GCF_004571195.1 GCF_004564075.1, GCF_004526345.1, GCF_004524775.2, GCF_004519515.1, GCF_004421025.1 GCF_004421005.1, GCF_004367745.1, GCF_004367585.1, GCF_004358345.1, GCF_004353865.1 GCF_004348195.1, GCF_004337635.1, GCF_004328625.1, GCF_004328555.1, GCF_004322755.1 GCF_004295665.1, GCF_004214815.1, GCF_004214795.1, GCF_004135975.1, GCF_004135935.1 GCF_004117095.1, GCF_004117055.1, GCF_004116975.1, GCF_004101845.1, GCF_004087915.1 GCF_004063735.1, GCF_004028275.1, GCF_004011905.1, GCF_004011115.1, GCF_004011095.1 GCF_004008975.1, GCF_004006435.1, GCF_003999355.1, GCF_003999335.1, GCF_003999255.1 GCF_003991875.1, GCF_003967075.1, GCF_003966975.1, GCF_003966735.1, GCF_003966655.1 GCF_003966625.1, GCF_003955735.1, GCF_003952265.1, GCF_003945385.1, GCF_003945365.1 GCF_003932995.1, GCF_003932715.1, GCF_003932015.1, GCF_003860585.1, GCF_003860565.1 GCF_003860345.1, GCF_003855395.1, GCF_003852045.1, GCF_003851555.1, GCF_003850565.1 GCF_003815995.1, GCF_003815975.1, GCF_003815915.1, GCF_003815875.1, GCF_003815775.1 GCF_003815695.1, GCF_003812505.1, GCF_003798325.1, GCF_003795145.1, GCF_003795125.1 GCF_003732525.1, GCF_003722315.1, GCF_003722295.1, GCF_003721155.3, GCF_003719195.1 GCF_003711105.1, GCF_003687415.2, GCF_003671975.1, GCF_003667885.1, GCF_003660105.1 GCF_003641185.1, GCF_003628755.1, GCF_003614235.1, GCF_003610015.1, GCF_003606285.1 GCF_003606265.1, GCF_003595235.1, GCF_003589745.1, GCF_003585765.1, GCF_003584745.1 GCF_003574835.2, GCF_003574215.1, GCF_003571725.1, GCF_003568825.1, GCF_003544935.1 GCF_003544915.1, GCF_003544855.1, GCF_003544835.1, GCF_003544815.1, GCF_003534205.1 GCF_003516145.1, GCF_003516125.1, GCF_003515985.1, GCF_003515105.1, GCF_003491405.1 GCF_003444775.1, GCF_003443655.1, GCF_003433515.1, GCF_003428925.1, GCF_003403135.1 GCF_003391255.1, GCF_003367705.1, GCF_003355515.1, GCF_003355475.1, GCF_003351565.1 GCF_003346815.1, GCF_003346775.1, GCF_003346755.1, GCF_003343305.1, GCF_003343245.1 GCF_003330865.1, GCF_003330825.1, GCF_003330785.1, GCF_003323815.1, GCF_003290385.1 GCF_003288115.1, GCF_003265225.1, GCF_003258335.1, GCF_003253775.1, GCF_003233655.1 GCF_003177055.1, GCF_003176835.1, GCF_003173695.1, GCF_003172995.1, GCF_003151025.1 GCF_003150935.1, GCF_003149495.1, GCF_003143515.1, GCF_003101015.1, GCF_003097575.1 GCF_003096175.1, GCF_003096095.1, GCF_003096075.1, GCF_003074995.2, GCF_003071405.1 GCF_003065365.1, GCF_003049785.1, GCF_003049605.2, GCF_003030465.1, GCF_003019925.1 GCF_003019695.1, GCF_003019675.1, GCF_003019655.1, GCF_003019315.1, GCF_003019295.1 GCF_003019255.1, GCF_003015125.1, GCF_003013675.1, GCF_003012915.1, GCF_003010915.2 GCF_003010495.1, GCF_002998925.1, GCF_002998535.1, GCF_002998435.1, GCF_002998295.1 GCF_002993285.1, GCF_002983865.1, GCF_002982115.1, GCF_002953735.1, GCF_002953195.1 GCF_002952315.1, GCF_002951935.1, GCF_002951835.1, GCF_002950575.1, GCF_002950395.1 GCF_002949635.1, GCF_002906475.1, GCF_002902965.1, GCF_002902925.1, GCF_002896855.1 GCF_002892185.1, GCF_002887615.1, GCF_002887555.1, GCF_002872415.1, GCF_002871995.2 GCF_002849875.1, GCF_002849835.1, GCF_002849795.1, GCF_002849715.1, GCF_002848365.2 GCF_002847845.2, GCF_002847445.1, GCF_002813755.1, GCF_002812705.1, GCF_002804245.1 GCF_002804205.1, GCF_002804005.1, GCF_002803845.1, GCF_002795865.1, GCF_002770595.1 GCF_002762215.1, GCF_002761235.1, GCF_002749675.1, GCF_002749615.1, GCF_002749495.1 GCF_002736065.1, GCF_002706795.1, GCF_002706745.1, GCF_002706425.1, GCF_002688605.1 GCF_002591335.1, GCF_002568625.1, GCF_002549795.1, GCF_002504085.1, GCF_002442935.1 GCF_002441935.1, GCF_002407485.1, GCF_002393505.1, GCF_002393445.1, GCF_002370525.2 GCF_002370195.2, GCF_002356555.2, GCF_002356315.1, GCF_002355975.1, GCF_002355855.1 GCF_002355775.1, GCF_002355595.1, GCF_002355535.1, GCF_002355475.1, GCF_002355215.1 GCF_002354875.1, GCF_002327205.1, GCF_002327145.1, GCF_002310835.1, GCF_002310795.1 GCF_002305895.1, GCF_002305855.1, GCF_002302565.1, GCF_002302495.1, GCF_002302475.1 GCF_002302415.1, GCF_002291445.1, GCF_002291425.1, GCF_002290025.1, GCF_002288525.1 GCF_002288285.1, GCF_002287965.1, GCF_002285935.1, GCF_002285715.1, GCF_002285635.2 GCF_002285575.1, GCF_002285515.1, GCF_002285495.1, GCF_002278135.3, GCF_002278035.1 GCF_002278015.2, GCF_002277935.1, GCF_002269385.1, GCF_002263515.1, GCF_002263495.1 GCF_002257705.2, GCF_002257695.2, GCF_002257585.1, GCF_002250055.1, GCF_002243515.1 GCF_002240415.1, GCF_002240355.1, GCF_002238335.1, GCF_002237575.1, GCF_002234495.1 GCF_002224645.1, GCF_002224365.1, GCF_002224265.1, GCF_002222655.1, GCF_002222595.2 GCF_002221505.1, GCF_002220285.1, GCF_002220155.1, GCF_002218045.2, GCF_002215585.1 GCF_002215215.1, GCF_002214645.1, GCF_002214625.1, GCF_002214395.1, GCF_002211785.1 GCF_002209125.2, GCF_002208825.2, GCF_002208805.2, GCF_002201795.1, GCF_002192415.1 GCF_002173775.1, GCF_002173515.1, GCF_002163585.1, GCF_002162375.1, GCF_002158865.1 GCF_002157855.1, GCF_002157835.1, GCF_002157205.1, GCF_002157165.1, GCF_002155265.1 GCF_002155145.1, GCF_002142475.1, GCF_002139935.1, GCF_002127965.1, GCF_002119765.1 GCF_002117445.1, GCF_002117105.1, GCF_002117085.1, GCF_002104335.1, GCF_002101335.1 GCF_002097715.1, GCF_002097535.1, GCF_002082195.1, GCF_002081995.1, GCF_002080475.1 GCF_002080395.1, GCF_002080125.1, GCF_002075795.1, GCF_002074155.1, GCF_002073495.2 GCF_002073255.2, GCF_002072065.1, GCF_002067135.1, GCF_002056795.1, GCF_002056725.1 GCF_002043005.1, GCF_002028405.1, GCF_002028325.1, GCF_002023665.2, GCF_002021755.1 GCF_002009335.2, GCF_002009295.1, GCF_002006355.2, GCF_002005485.1, GCF_002005465.1 GCF_002005405.1, GCF_002005305.1, GCF_001999945.1, GCF_001999225.1, GCF_001998865.1 GCF_001991075.2, GCF_001988955.1, GCF_001984445.1, GCF_001975955.2, GCF_001975705.1 GCF_001975665.1, GCF_001974985.1, GCF_001969385.1, GCF_001955735.1, GCF_001953955.1 GCF_001951175.1, GCF_001951155.1, GCF_001951095.1, GCF_001941945.1, GCF_001941825.1 GCF_001941585.1, GCF_001941565.1, GCF_001941465.1, GCF_001941425.1, GCF_001941345.1 GCF_001936235.1, GCF_001936175.1, GCF_001922385.1, GCF_001922305.1, GCF_001908725.1 GCF_001908275.1, GCF_001895265.1, GCF_001889445.1, GCF_001889105.1, GCF_001888925.1 GCF_001887245.1, GCF_001886815.1, GCF_001886695.1, GCF_001886435.1, GCF_001878675.1 GCF_001874645.1, GCF_001870205.1, GCF_001865575.2, GCF_001858005.1, GCF_001857925.1 GCF_001856645.1, GCF_001855275.1, GCF_001854325.1, GCF_001854225.1, GCF_001787355.1 GCF_001761545.1, GCF_001753205.1, GCF_001750725.1, GCF_001747425.1, GCF_001747405.1 GCF_001742225.1, GCF_001742185.1, GCF_001735765.2, GCF_001723605.1, GCF_001721685.1 GCF_001721185.1, GCF_001720485.1, GCF_001719165.1, GCF_001718895.1, GCF_001718555.1 GCF_001718535.1, GCF_001717525.2, GCF_001715535.1, GCF_001712815.1, GCF_001708485.1 GCF_001708425.1, GCF_001708405.1, GCF_001705175.1, GCF_001705075.2, GCF_001703515.1 GCF_001702215.1, GCF_001702155.1, GCF_001702115.1, GCF_001701045.1, GCF_001700985.1 GCF_001698225.1, GCF_001698205.1, GCF_001693515.2, GCF_001693385.1, GCF_001693335.1 GCF_001687665.2, GCF_001687625.2, GCF_001687605.2, GCF_001687585.2, GCF_001687565.2 GCF_001687545.1, GCF_001686985.1, GCF_001685435.2, GCF_001685355.1, GCF_001682385.1 GCF_001676725.1, GCF_001676705.1, GCF_001664385.1, GCF_001663855.1, GCF_001663675.1 GCF_001663175.1, GCF_001663155.1, GCF_001660485.1, GCF_001660045.1, GCF_001659785.1 GCF_001658025.2, GCF_001654515.1, GCF_001654455.1, GCF_001652465.1, GCF_001644705.1 GCF_001644605.1, GCF_001644565.1, GCF_001643775.1, GCF_001642675.1, GCF_001642655.1 GCF_001641285.1, GCF_001641005.1, GCF_001636015.1, GCF_001634285.1, GCF_001633165.1 GCF_001618845.1, GCF_001618685.1, GCF_001617625.1, GCF_001611675.1, GCF_001611155.1 GCF_001610955.1, GCF_001606025.1, GCF_001605725.1, GCF_001597285.1, GCF_001590685.1 GCF_001587155.1, GCF_001586235.1, GCF_001586155.1, GCF_001584225.1, GCF_001584205.1 GCF_001584145.1, GCF_001579945.1, GCF_001577305.1, GCF_001562195.1, GCF_001561955.1 GCF_001559015.1, GCF_001558775.1, GCF_001558415.2, GCF_001558255.2, GCF_001553955.1 GCF_001553625.1, GCF_001553605.1, GCF_001553565.1, GCF_001552035.1, GCF_001547995.1 GCF_001545155.1, GCF_001543305.1, GCF_001543285.1, GCF_001543245.1, GCF_001543205.1 GCF_001543175.1, GCF_001543145.1, GCF_001543105.1, GCF_001542625.1, GCF_001535545.1 GCF_001518835.1, GCF_001513745.1, GCF_001509405.1, GCF_001499615.1, GCF_001483865.1 GCF_001482365.1, GCF_001465835.2, GCF_001465795.2, GCF_001465595.2, GCF_001465545.3 GCF_001465255.1, GCF_001460635.1, GCF_001457635.1, GCF_001457555.1, GCF_001457455.1 GCF_001456255.1, GCF_001455205.1, GCF_001444445.1, GCF_001444425.1, GCF_001444405.1 GCF_001442805.1, GCF_001442745.1, GCF_001441165.1, GCF_001431725.1, GCF_001414055.1 GCF_001411805.1, GCF_001402875.1, GCF_001399775.1, GCF_001399515.1, GCF_001314995.1 GCF_001314325.1, GCF_001310085.1, GCF_001305675.1, GCF_001305595.1, GCF_001305575.3 GCF_001302585.1, GCF_001298465.1, GCF_001296145.1, GCF_001281485.1, GCF_001281385.1 GCF_001281085.1, GCF_001281025.1, GCF_001277995.1, GCF_001277255.1, GCF_001277235.1 GCF_001277215.2, GCF_001277195.1, GCF_001277175.1, GCF_001275365.1, GCF_001273775.1 GCF_001267925.1, GCF_001267885.1, GCF_001267435.1, GCF_001267155.1, GCF_001262715.1 GCF_001262015.1, GCF_001191605.1, GCF_001190945.1, GCF_001190745.1, GCF_001189295.1 GCF_001187595.1, GCF_001051995.2, GCF_001050475.1, GCF_001050435.1, GCF_001050135.1 GCF_001050115.1, GCF_001043175.1, GCF_001042715.1, GCF_001042695.1, GCF_001042675.1 GCF_001042655.1, GCF_001042635.1, GCF_001042595.1, GCF_001040945.1, GCF_001038625.1 GCF_001029105.3, GCF_001028665.1, GCF_001028625.1, GCF_001027285.1, GCF_001025215.1 GCF_001025195.1, GCF_001025175.1, GCF_001025155.1, GCF_001021085.1, GCF_001021065.1 GCF_001021045.1, GCF_001021025.1, GCF_001020985.1, GCF_001020955.1, GCF_001017775.3 GCF_001017435.1, GCF_001010285.1, GCF_001008165.2, GCF_001007935.1, GCF_001006005.1 GCF_000993785.3, GCF_000988745.2, GCF_000987835.1, GCF_000981765.1, GCF_000981585.1 GCF_000980835.1, GCF_000980815.1, GCF_000975265.2, GCF_000974685.2, GCF_000973725.1 GCF_000973705.1, GCF_000973105.1, GCF_000973085.1, GCF_000972785.3, GCF_000968535.2 GCF_000968375.1, GCF_000968195.1, GCF_000967915.1, GCF_000967425.1, GCF_000967305.2 GCF_000963865.1, GCF_000961515.1, GCF_000961215.1, GCF_000961095.1, GCF_000960995.1 GCF_000959545.1, GCF_000959365.1, GCF_000954135.1, GCF_000953715.1, GCF_000953655.1 GCF_000953635.1, GCF_000953135.1, GCF_000948985.2, GCF_000948975.2, GCF_000943515.2 GCF_000934605.2, GCF_000934565.1, GCF_000835165.1, GCF_000834455.1, GCF_000833575.1 GCF_000833025.1, GCF_000832905.1, GCF_000831645.3, GCF_000831485.1, GCF_000830005.1 GCF_000829395.1, GCF_000828635.1, GCF_000828615.1, GCF_000828515.1, GCF_000827125.1 GCF_000821185.2, GCF_000819565.1, GCF_000818015.1, GCF_000817955.1, GCF_000816845.1 GCF_000816185.1, GCF_000816085.1, GCF_000815225.1, GCF_000815105.2, GCF_000807675.2 GCF_000807275.1, GCF_000800395.1, GCF_000785495.1, GCF_000785105.2, GCF_000783935.2 GCF_000772105.1, GCF_000767685.1, GCF_000767465.1, GCF_000767055.1, GCF_000763575.1 GCF_000763515.1, GCF_000761155.1, GCF_000758725.1, GCF_000758705.1, GCF_000758685.1 GCF_000758665.1, GCF_000757795.1, GCF_000756615.1, GCF_000755585.2, GCF_000747315.1 GCF_000743945.1, GCF_000742835.1, GCF_000740965.1, GCF_000739435.1, GCF_000739085.1 GCF_000737865.1, GCF_000737325.1, GCF_000736415.1, GCF_000734015.1, GCF_000733715.2 GCF_000732945.1, GCF_000732925.1, GCF_000731315.1, GCF_000730385.1, GCF_000725405.1 GCF_000725365.1, GCF_000724775.3, GCF_000724625.1, GCF_000724605.1, GCF_000723425.2 GCF_000723365.1, GCF_000709415.1, GCF_000699505.1, GCF_000697965.2, GCF_000696675.2 GCF_000696345.2, GCF_000632985.1, GCF_000632845.1, GCF_000619905.2, GCF_000612505.1 GCF_000600105.1, GCF_000600005.1, GCF_000599985.1, GCF_000597865.1, GCF_000583855.1 GCF_000582515.1, GCF_000577895.1, GCF_000576555.1, GCF_000568815.1, GCF_000565175.1 GCF_000550805.1, GCF_000550785.1, GCF_000522985.1, GCF_000521725.1, GCF_000521505.1 GCF_000513475.1, GCF_000512915.1, GCF_000512735.1, GCF_000512205.2, GCF_000511355.1 GCF_000504585.2, GCF_000504105.1, GCF_000504085.1, GCF_000503895.1, GCF_000500935.1 GCF_000499665.2, GCF_000498655.1, GCF_000494755.1, GCF_000493735.1, GCF_000484535.1 GCF_000479335.1, GCF_000477435.1, GCF_000471025.2, GCF_000463345.2, GCF_000455605.1 GCF_000447675.1, GCF_000445445.2, GCF_000442645.1, GCF_000439435.1, GCF_000418365.1 GCF_000412675.1, GCF_000402035.1, GCF_000400935.1, GCF_000400635.2, GCF_000376545.2 GCF_000367205.1, GCF_000355765.4, GCF_000355695.1, GCF_000348725.1, GCF_000344805.1 GCF_000344785.1, GCF_000341395.1, GCF_000341355.1, GCF_000341345.1, GCF_000340885.1 GCF_000340435.2, GCF_000336465.1, GCF_000332115.1, GCF_000331995.1, GCF_000331735.1 GCF_000331715.1, GCF_000331185.2, GCF_000330885.1, GCF_000328705.1, GCF_000328625.1 GCF_000327045.1, GCF_000325705.1, GCF_000321415.2, GCF_000319575.2, GCF_000319385.1 GCF_000317835.1, GCF_000317695.1, GCF_000317675.1, GCF_000317615.1, GCF_000317575.1 GCF_000317515.1, GCF_000317495.1, GCF_000317475.1, GCF_000317305.3, GCF_000317125.1 GCF_000317105.1, GCF_000316515.1, GCF_000313175.2, GCF_000309885.1, GCF_000307585.2 GCF_000307165.1, GCF_000307105.1, GCF_000306675.2, GCF_000305935.1, GCF_000300455.3 GCF_000300235.2, GCF_000300095.1, GCF_000300005.1, GCF_000299355.1, GCF_000299335.2 GCF_000298875.1, GCF_000297075.2, GCF_000297055.2, GCF_000284635.1, GCF_000284615.1 GCF_000284515.1, GCF_000284415.1, GCF_000284335.1, GCF_000284315.1, GCF_000284295.1 GCF_000284155.1, GCF_000284115.1, GCF_000284075.1, GCF_000283915.1, GCF_000283595.1 GCF_000281175.1, GCF_000280865.2, GCF_000279145.1, GCF_000277895.2, GCF_000277165.1 GCF_000276825.1, GCF_000270245.1, GCF_000270085.1, GCF_000269985.1, GCF_000266945.1 GCF_000266885.1, GCF_000265505.1, GCF_000265465.1, GCF_000265425.1, GCF_000265405.1 GCF_000265385.1, GCF_000265295.1, GCF_000264765.2, GCF_000263195.1, GCF_000262305.1 GCF_000261345.2, GCF_000260985.4, GCF_000260965.1, GCF_000259275.1, GCF_000258535.2 GCF_000258405.1, GCF_000255295.1, GCF_000255135.1, GCF_000255115.2, GCF_000253375.1 GCF_000253315.1, GCF_000253275.1, GCF_000253175.1, GCF_000253035.1, GCF_000253015.1 GCF_000252995.1, GCF_000252445.1, GCF_000250635.1, GCF_000247715.1, GCF_000247605.1 GCF_000247565.1, GCF_000246855.1, GCF_000243155.2, GCF_000243135.2, GCF_000243115.2 GCF_000242915.1, GCF_000242635.2, GCF_000242595.2, GCF_000242455.2, GCF_000242335.1 GCF_000242255.2, GCF_000240185.1, GCF_000238395.3, GCF_000238255.3, GCF_000238215.1 GCF_000237845.1, GCF_000237305.1, GCF_000237205.1, GCF_000237085.1, GCF_000236925.1 GCF_000236705.1, GCF_000236685.1, GCF_000236665.1, GCF_000235605.1, GCF_000235585.1 GCF_000235405.2, GCF_000233915.3, GCF_000233715.2, GCF_000233595.1, GCF_000231405.2 GCF_000231385.2, GCF_000230995.2, GCF_000230655.2, GCF_000227745.2, GCF_000227705.2 GCF_000227685.2, GCF_000227665.2, GCF_000227465.1, GCF_000226625.1, GCF_000226565.1 GCF_000226315.1, GCF_000226295.1, GCF_000225345.1, GCF_000224985.1, GCF_000224675.1 GCF_000224085.1, GCF_000224005.2, GCF_000223215.1, GCF_000222975.1, GCF_000222485.1 GCF_000221985.1, GCF_000221025.1, GCF_000220945.1, GCF_000220705.2, GCF_000219915.2 GCF_000219805.1, GCF_000219725.1, GCF_000219355.1, GCF_000219215.1, GCF_000219045.1 GCF_000218895.1, GCF_000218855.1, GCF_000218625.1, GCF_000218565.1, GCF_000218545.1 GCF_000217815.1, GCF_000217795.1, GCF_000217655.1, GCF_000215975.1, GCF_000215705.1 GCF_000215645.1, GCF_000215105.1, GCF_000215085.1, GCF_000214825.1, GCF_000214705.1 GCF_000214435.1, GCF_000214375.1, GCF_000214355.1, GCF_000214235.1, GCF_000214215.1 GCF_000214175.1, GCF_000214155.1, GCF_000213655.1, GCF_000213255.1, GCF_000213235.1 GCF_000212735.1, GCF_000212695.1, GCF_000212675.2, GCF_000212415.1, GCF_000212395.1 GCF_000212375.1, GCF_000210915.2, GCF_000208405.1, GCF_000208385.1, GCF_000204645.1 GCF_000204255.1, GCF_000204155.1, GCF_000204135.1, GCF_000202835.1, GCF_000200595.1 GCF_000199675.1, GCF_000197735.1, GCF_000196875.2, GCF_000196855.1, GCF_000196795.1 GCF_000196615.1, GCF_000196555.1, GCF_000196535.1, GCF_000196495.1, GCF_000196475.1 GCF_000196455.1, GCF_000196435.1, GCF_000196355.1, GCF_000196315.1, GCF_000196275.1 GCF_000196215.1, GCF_000196175.1, GCF_000196115.1, GCF_000196095.1, GCF_000196075.1 GCF_000196035.1, GCF_000195975.1, GCF_000195955.2, GCF_000195555.1, GCF_000195335.1 GCF_000195315.1, GCF_000195295.1, GCF_000195275.1, GCF_000194605.1, GCF_000194135.1 GCF_000194115.1, GCF_000192865.1, GCF_000192745.1, GCF_000191145.1, GCF_000191045.1 GCF_000190735.1, GCF_000190635.1, GCF_000190595.1, GCF_000190575.1, GCF_000190555.1 GCF_000190435.1, GCF_000189775.2, GCF_000189295.2, GCF_000187935.1, GCF_000186885.1 GCF_000186385.1, GCF_000186345.1, GCF_000186265.1, GCF_000186245.1, GCF_000186225.1 GCF_000185805.1, GCF_000184705.1, GCF_000184685.1, GCF_000184435.1, GCF_000184345.1 GCF_000183745.1, GCF_000183725.1, GCF_000183425.1, GCF_000183405.1, GCF_000183155.1 GCF_000183135.1, GCF_000182745.2, GCF_000179915.2, GCF_000179635.2, GCF_000178975.2 GCF_000178955.2, GCF_000178875.2, GCF_000178835.2, GCF_000178115.2, GCF_000177635.2 GCF_000177535.2, GCF_000177235.2, GCF_000176915.2, GCF_000176855.2, GCF_000176035.2 GCF_000175295.2, GCF_000175215.2, GCF_000172155.1, GCF_000170955.2, GCF_000166935.1 GCF_000166775.1, GCF_000166695.1, GCF_000166415.1, GCF_000166395.1, GCF_000166355.1 GCF_000166335.1, GCF_000166135.1, GCF_000166055.1, GCF_000165715.2, GCF_000165505.1 GCF_000165465.1, GCF_000164905.1, GCF_000164695.2, GCF_000164675.2, GCF_000163895.2 GCF_000157895.3, GCF_000157355.2, GCF_000156995.2, GCF_000155735.2, GCF_000155675.2 GCF_000155515.2, GCF_000154785.2, GCF_000153485.2, GCF_000152825.2, GCF_000152245.2 GCF_000148645.1, GCF_000147715.2, GCF_000147695.2, GCF_000147355.1, GCF_000147335.1 GCF_000146505.1, GCF_000146185.1, GCF_000146165.2, GCF_000146065.2, GCF_000146045.2 GCF_000145945.2, GCF_000145615.1, GCF_000145275.1, GCF_000145255.1, GCF_000145235.1 GCF_000145215.1, GCF_000145035.1, GCF_000144695.1, GCF_000144645.1, GCF_000144625.1 GCF_000144605.1, GCF_000144405.1, GCF_000143985.1, GCF_000143965.1, GCF_000143845.1 GCF_000143725.1, GCF_000143685.1, GCF_000143145.1, GCF_000143085.1, GCF_000093085.1 GCF_000093025.1, GCF_000092965.1, GCF_000092925.1, GCF_000092905.1, GCF_000092865.1 GCF_000092845.1, GCF_000092825.1, GCF_000092785.1, GCF_000092645.1, GCF_000092505.1 GCF_000092425.1, GCF_000092405.1, GCF_000092245.1, GCF_000092225.1, GCF_000092205.1 GCF_000092125.1, GCF_000092105.1, GCF_000092045.1, GCF_000092025.1, GCF_000091785.1 GCF_000091545.1, GCF_000091325.1, GCF_000091305.1, GCF_000091125.1, GCF_000083545.1 GCF_000069965.1, GCF_000069225.1, GCF_000069185.1, GCF_000067205.1, GCF_000067165.1 GCF_000063585.1, GCF_000062885.1, GCF_000060345.1, GCF_000058485.1, GCF_000055785.1 GCF_000046845.1, GCF_000027145.1, GCF_000026745.1, GCF_000026185.1, GCF_000026105.1 GCF_000026085.1, GCF_000026005.1, GCF_000025985.1, GCF_000025965.1, GCF_000025945.1 GCF_000025925.1, GCF_000025905.1, GCF_000025885.1, GCF_000025725.1, GCF_000025705.1 GCF_000025645.1, GCF_000025605.1, GCF_000025485.1, GCF_000025345.1, GCF_000025305.1 GCF_000025265.1, GCF_000025225.2, GCF_000025065.1, GCF_000025005.1, GCF_000024985.1 GCF_000024965.1, GCF_000024925.1, GCF_000024905.1, GCF_000024885.1, GCF_000024865.1 GCF_000024845.1, GCF_000024825.1, GCF_000024805.1, GCF_000024785.1, GCF_000024765.1 GCF_000024725.1, GCF_000024605.1, GCF_000024565.1, GCF_000024545.1, GCF_000024505.1 GCF_000024465.1, GCF_000024425.1, GCF_000024405.1, GCF_000024385.1, GCF_000024365.1 GCF_000024345.1, GCF_000024325.1, GCF_000024285.1, GCF_000024225.1, GCF_000024205.1 GCF_000024125.1, GCF_000024105.1, GCF_000024085.1, GCF_000024025.1, GCF_000024005.1 GCF_000023905.1, GCF_000023865.1, GCF_000023845.1, GCF_000023825.1, GCF_000023785.1 GCF_000023745.1, GCF_000023705.1, GCF_000023565.1, GCF_000023545.1, GCF_000023465.1 GCF_000023445.1, GCF_000023325.1, GCF_000023285.1, GCF_000023265.1, GCF_000023245.1 GCF_000023225.1, GCF_000023145.1, GCF_000023125.1, GCF_000023105.1, GCF_000023065.1 GCF_000022965.1, GCF_000022905.1, GCF_000022745.1, GCF_000022725.1, GCF_000022565.1 GCF_000022525.1, GCF_000022325.1, GCF_000022265.1, GCF_000022145.1, GCF_000022085.1 GCF_000022065.1, GCF_000022025.1, GCF_000022005.1, GCF_000021985.1, GCF_000021945.1 GCF_000021925.1, GCF_000021905.1, GCF_000021865.1, GCF_000021825.1, GCF_000021805.1 GCF_000021765.1, GCF_000021745.1, GCF_000021725.1, GCF_000021685.1, GCF_000021645.1 GCF_000021565.1, GCF_000021545.1, GCF_000021485.1, GCF_000021325.1, GCF_000021285.1 GCF_000021045.1, GCF_000020985.1, GCF_000020965.1, GCF_000020945.1, GCF_000020725.1 GCF_000020685.1, GCF_000020645.1, GCF_000020625.1, GCF_000020565.1, GCF_000020525.1 GCF_000020505.1, GCF_000020485.1, GCF_000020465.1, GCF_000020385.1, GCF_000020365.1 GCF_000020305.1, GCF_000020145.1, GCF_000020125.1, GCF_000020045.1, GCF_000020025.1 GCF_000020005.1, GCF_000019965.1, GCF_000019945.1, GCF_000019905.1, GCF_000019845.1 GCF_000019785.1, GCF_000019725.1, GCF_000019705.1, GCF_000019685.1, GCF_000019665.1 GCF_000019525.1, GCF_000019505.1, GCF_000019405.1, GCF_000019225.1, GCF_000019185.1 GCF_000019165.1, GCF_000019085.1, GCF_000019045.1, GCF_000018945.1, GCF_000018885.1 GCF_000018865.1, GCF_000018785.1, GCF_000018685.1, GCF_000018665.1, GCF_000018605.1 GCF_000018525.1, GCF_000018405.1, GCF_000018325.1, GCF_000018285.1, GCF_000018225.1 GCF_000018205.1, GCF_000018145.1, GCF_000018105.1, GCF_000018045.1, GCF_000018025.1 GCF_000017885.4, GCF_000017865.1, GCF_000017845.1, GCF_000017805.1, GCF_000017685.1 GCF_000017585.1, GCF_000017565.1, GCF_000017545.1, GCF_000017425.1, GCF_000017305.1 GCF_000017265.1, GCF_000017245.1, GCF_000016985.1, GCF_000016905.1, GCF_000016845.1 GCF_000016785.1, GCF_000016745.1, GCF_000016645.1, GCF_000016545.1, GCF_000016425.1 GCF_000016345.1, GCF_000016285.1, GCF_000016185.1, GCF_000016165.1, GCF_000016065.1 GCF_000015865.1, GCF_000015785.2, GCF_000015725.1, GCF_000015665.1, GCF_000015585.1 GCF_000015565.1, GCF_000015505.1, GCF_000015485.1, GCF_000015445.1, GCF_000015345.1 GCF_000015305.1, GCF_000015285.1, GCF_000015245.1, GCF_000015125.1, GCF_000015045.1 GCF_000015025.1, GCF_000014965.1, GCF_000014885.1, GCF_000014865.1, GCF_000014805.1 GCF_000014785.1, GCF_000014765.1, GCF_000014725.1, GCF_000014705.1, GCF_000014505.1 GCF_000014445.1, GCF_000014425.1, GCF_000014265.1, GCF_000014185.1, GCF_000014145.1 GCF_000014025.1, GCF_000014005.1, GCF_000013985.1, GCF_000013905.1, GCF_000013885.1 GCF_000013765.1, GCF_000013705.1, GCF_000013665.1, GCF_000013645.1, GCF_000013605.1 GCF_000013425.1, GCF_000013405.1, GCF_000013345.1, GCF_000013325.1, GCF_000013165.1 GCF_000013145.1, GCF_000013085.1, GCF_000013025.1, GCF_000012985.1, GCF_000012965.1 GCF_000012945.1, GCF_000012925.1, GCF_000012885.1, GCF_000012865.1, GCF_000012825.1 GCF_000012805.1, GCF_000012725.1, GCF_000012685.1, GCF_000012665.1, GCF_000012565.1 GCF_000012485.1, GCF_000012405.1, GCF_000012385.1, GCF_000012365.1, GCF_000012325.1 GCF_000012305.1, GCF_000012085.2, GCF_000011965.2, GCF_000011905.1, GCF_000011805.1 GCF_000011565.2, GCF_000011385.1, GCF_000011345.1, GCF_000011325.1, GCF_000011305.1 GCF_000011245.1, GCF_000010985.1, GCF_000010665.1, GCF_000010525.1, GCF_000010505.1 GCF_000010425.1, GCF_000010305.1, GCF_000010265.1, GCF_000010125.1, GCF_000010085.1 GCF_000009985.1, GCF_000009945.1, GCF_000009905.1, GCF_000009805.1, GCF_000009785.1 GCF_000009765.2, GCF_000009365.1, GCF_000009305.1, GCF_000009265.1, GCF_000009145.1 GCF_000009085.1, GCF_000009045.1, GCF_000008865.2, GCF_000008725.1, GCF_000008625.1 GCF_000008465.1, GCF_000008445.1, GCF_000008325.1, GCF_000008305.1, GCF_000008185.1 GCF_000008045.1, GCF_000007985.2, GCF_000007905.1, GCF_000007765.2, GCF_000007625.1 GCF_000007605.1, GCF_000007505.1, GCF_000007205.1, GCF_000007125.1, GCF_000007085.1 GCF_000007025.1, GCF_000006985.1, GCF_000006945.2, GCF_000006925.2, GCF_000006765.1 GCF_000006685.1

**Supplementary Information 1.3.** Protein sequences used to generate the alignment in Supplementary Figure 2

>OG362_RS07370 | TM

QALHDAGGLEFQVKGTNVQISSDMCPQLASALNNTQLNEMIKDCTAAQRQHALDSLLNR

>XNR_RS05000 | TM

QAVHVGNELPFQVTSGTFTIYSDACPALSGRRFTSSGAFNDAMKLCIDHQRQQALDDLLSRS

>CP975_RS26985 | TM

AQALNVGTDLPFTIEGGSKVSSDTCHNLPTGTQIPAEQVNAALNECVNQVRRNALDNLLSRS

>K1J60_RS11550 | TM

QAVNTGNAPLFKIVGGTDISVTSEKCPAVAGSTNVRLADFNAAISDCIDAQRQVALDTLLSRS

>K7396_RS09365 | TM

ADALHDGSALPLKILGGKFQSTSDICDLPTETSGQMLQQAVEQCLQHQRALALNSLLNRS

>NR995_RS07620 | TM

QALDQISELPFRILSGKTQPTSDRCPGLTGTLPSDVFTDRLSACLEAQRGIALDGLLRRS

>SAM23877_RS26430 | TM

QAVRTGNQPLYKIVDFEGLRVASNDCPGVTNGNLSLSEFNAAISECIDHERQAALDRLLSRS

>H9W91_RS28360 | TM

QALNVGSELPFKIVEGRVASTVCNLPPNPSADDLNRAMGACVNEQRQHALDNLLSRS

>OIE72_RS09030 | TM

QAVRTGNEPLYKIVDFTDLKVSSSTCPVVDNGGLSLSDFNAAISDCMDHQRKVALDNLLSRS

>LK895_RS07485 | TM

AQALHVGTDLPFKLLPDSKIQLTNNACPALTPGLSADEANAALKACNESQRKHALDTLLNRS

>EJC51_RS35435 | TM

NALNVGSDLPFKVVTGTVSSESCNITSTQLPAPDLNHALNECVNEQRQHALDNLLSRS

>QR300_RS12510 | TM

QALHVGNELPFKLVGGSVQPTNNTCPEIIGQSSPDQFNAVLNTCMKEQRQLALDGLLRRS

>HKX69_RS08265 | TM

QAISTGNQPLFKIVSGSAIKVTSDNCPAVTAINSGPVPLSDFNEAIAHCVDQQRQAALDNLLSRS

>IGS71_RS09820 | TM

QALHEGSGVDFRVTGSNLELTSGTCPQLSGASSNEELNAMLKECTAAQRQHALDGLLSRS

>SU9_RS26335 | TM

AADALHDGSALPLKILGGKFQSTSNLCDLPTKTSGPLLQEAVEQCLQQQRALALNSLLNRS

>SAVERM_RS12550 | TM

QALRTGSEPLFKILDGNGIKVASDDCPGVSTNNIPLTAFNDAISACTDHQRQVALDNLLSRS

>C5746_RS31245 | TM

QALQVGSELPFKIVSGQVTSDVCNFHGITTPDAANAAMNTCVNHQRQQALDTLLNRS

>GQF42_RS32020 | TM

QAISTGNQPLFKIVSFQSLRVSSDNCPAITTTSLSLQEFNDAISQCVDHQRQAALDDLLSRS

>H7H31_RS07020 | TM

DALHKGNELPFQIINADYRATSDVCDLPDRGNSAQFNHAVAQCMQGQREMALDGLLRRS

>G4Z16_RS06790 | TM

EALHEGSKLPFRILNANAQLTSDSCPGLTGTLPSEQFMERLGECTDAQRAVALDELLQRS

>CD934_RS07910 | TM

QALHDGGSGQSFQVTGTNIDITSETCPQLDTATTNSQLNDMLKQCNAAQRQHALDDLLSRS

>DN051_RS11225 | TM

ANALNVGSDLPFKILSGQVASETCNFPSQPTATELNHAMNECVNEQRQHALDNLLSRS

>OG436_RS28770 | TM

ADALHDGSALPLKILGGKFQSTSDICDLPTETSGQLLQQAVEQCLQHQRAVALNSLLNRS

>I6J39_RS27535 | TM

QALHDAGSLELKVGGTNVLIASDTCPQLSSATNIDQVNETIKACTAAQRQHALDTLLNRS

>PYS65_RS09315 | TM

ANALKVGSDLPFRILSGQVSSDTCQLRSAQLPADELNRALNECVNEQRQHALDNLLSRS

>CVT27_RS26525 | TM

QALGVGSKLPFEIVSGRVASDICDLPTTPSPEAFNAAMNACVNNQRKEALETLLNRS

>IHE65_RS10955 | TM

QAVNTGNAPLFKIVGGTDISVTSEKCPAVDAGSTNVRLSDFNAAISACIDAQRQVALDTLLSRS

>IPT68_RS27775 | TM

DAMNVGSELPFKILSGQVASETCNFPGSPTAAELNQAMNACVNEQRQHALDNLLSRS

>CP977_RS26050 | TM

TAQALSDSAAQLPFKVVDGTVQPTTSWCELPVKSTGDQLNDAVTLCLRHQGDRALEDLLRR

>OHT68_RS12785 | TM

ANALNVGSNLPFKIVSGGVTSDVCKLSGSELPADELNHALNQCVNDQRQHALDNLLSRS

>NRO40_RS22695 | TM

TRQALSISTDDLPFKLLKGEVEPNFDWCRLPVEGVTPDQFNDAMAACLQHQRDLAQDELLRR

>CP976_RS32095 | TM

EALNVGSELPFKIVEGKVTSDICNLPDQASPSEFNHAMNQCVNDQRKAALDNLLSRS

>QU709_RS10070 | TM

QAISTGNQPPFKIISASTDVKIASPDCPAVNNASGLTLDQFNSAISACIDHQRTIALDNLLSRS

>STRCI_RS30195 | TM

ASALNVGSDLPFKILSGQVSSDMCNLGATRLPADELNHALNECVNEQREHALDTLLSRS

>CP983_RS11290 | TM

QAINTGNEPLFKIVGGAELKVTSDNCPAINNASNLTYPQFNDAVSACIDDQRRVALDDLLSR

>S1361_RS29090 | TM

QAISTGNQPLFKIVSGSAIKVTSDNCPAVTAINSGPVPLSDFNDAIAHCVDQQRQGALDNLLSR

>I3J06_RS05000 | TM

QALTSGSSLPFKLLPESRIQRTNDSCPALMPGLSAEQANSVLSACFAHEREVALDDLLQRS

>N8I84_RS29800 | TM

HALNVGSELPFKILQGQVASDTCNLPQQTSASVLNDALDACVNEQRQHALDNLLSQ

>EJ357_RS34470 | TM

QAISTGNQPPFKIVGATDEIKVASADCPAVNNATGLTLTQFNSAISACIDHQRQVALDDLLSRS

>K7C20_RS27670 | TM

ADALHDGSALPLKILGGKFQSTSDICDLPTETSGQLLQQAVEQCLQHQRAVALNSLLNRS

>R2B67_RS07110 | TM

QALHDAGSLEFKVGGNNVLIASDTCPQLGAATNNDQLNETIKACTAAQRQHALDSLLNR

>D9753_RS08765 | TM

QAISSGNQPLFKIVSFQKLQVSSDNCPAINTTSLPLEDFKDAISRCVDHQRQGALDNLLSRS

>C4B68_RS09950 | TM

QALNVGTELPFKITEGRVTSTICNLPDSASPDVFNNAMNACVNEQRQHALDNLLSRS

>OHA91_RS10500 | TM

QALRQGNALPFQIVGGQVKVTSSSCPGVVGLELTPDQFNAAIGQCILEQRRHALDDLLSR

>L3078_RS33695 | TM

QAVNTGNAPLFKIVGGTDISVTSEKCPAVDAGSTNVRLADFNAAIAACIDDQRKVALDTLLSR

>M4V62_RS12130 | TM

QALNVGSDLPFKIVSGQVASDTCNFPSSPSAGELNNAMNACVNEQRQHALDNLLSRS

>OG858_RS33935 | TM

QAVSTGNTPVFKIEDGKDISVSSNICPAVDANTAPTNLRLDDFNAAISACIDHERQVALDTLLSR

>EIZ62_RS07215 | TM

QALHIGSELPFKIVNGQVTSNVCNFPDEAGPDEFNRAMNSCANEQRQHALDDLLRRS

>OJ254_RS00100 | TM

AADALHSGSALPLKILGGKFESTSDICDLPTQTSGPLLQQAVEQCLQQQRALALNSLLNRS

>GFH48_RS11345 | TM

QALNVGSELPFKIISGQVGSSTCNFPSAPSASELNTAMNACANQQRQHALDDLLSRS

>BFF78_RS11865 | TM

HALNVGSELPFKILSGSVSSSTCNFPSQPSASELNTAMNDCVNQQRQHALDDLLSRS

>CNQ36_RS29360 | TM

QALHDGGGGQSFQVTGTNIDISSQTCPQLDSATTNGQLNDMLKQCNAVQRQHALDDLLSRS

>IM697_RS33830 | TM

QAINTGNEPLFKIVSFRELQVSSDNCPGIKPGLTISEFNEAISACIDSQRRVALDDLLSRS

>J4032_RS09570 | TM

QAVQVGSQLPFKIVDGSVTSEVCNFPDKAPPAEFNEAMNACVNEQRQHALDELLTRS

>CP966_RS27480 | TM

NALNVGSELPFKILTGQVASDICNFPNEPTASELNGAMNECVNQQRQHALDDLLSRS

>B1H19_RS29985 | TM

ANTLSVGNQLPFKIVGASELHVTSDTCPELTPAVRNVAQLNSAVAECLQHQRTFALNTLLNRS

>CP974_RS23385 | TM

TRQALYISTDDLPFKLLKGEVDPNFDWCRLPAEGVTAEQFNDAMAGCLQHQRDLALDDLLRR

>H4W23_RS29855 | TM

TAQTLGESAAKLPFEIVTGKVQPTTSWCELPVQGSGKQFNDAVSVCLRHQGDLALEDLLRR

>C0216_RS07905 | TM

QALREGNALPFKIVSISGQKVVVSSPTCPGVGPEQTLDQFHAAIQACMLEQRRHALDDLLSR

>IAG43_RS24185 | TM

TAQALHNISELPFRLLPDSKIELTDNSCEALRAGLSADQANAALKSCFAHQRELALDDLLRR

>SGFS_RS17585 | TM

QALHEGGGPSFQVTGANLDITSSTCPAVNTVPDSEINSVLKQCDAIERQQALDTLLSRS

>test1122_RS21635 | TM

QALQEGSKLPIRITGLDLQVSSETCQLPTIGTSDDLNRALDACLQHQRRVALESLLKHS

>AVL59_RS10495 | TM

QAITTGNATLFKIVSFQNLQVASDNCPAITNHLPLADFNEAISRCADQQRQTALDNLLSRS

>PET44_RS24565 | TM

QALREGNALPFKIVSVSGQKVEVSSPTCSGVGTDQSLDQFNAAIQACILDQRKHALDDLLSR

>SGR_RS08160 | TM

QALSVGTDLPFEIVSGKVTSEICDLPANASPNDFNAAMNACVNHQRKAALETLLNRS

>HEP81_RS10830 | TM

QAISTGNQPLFKIVSFQELKVSSDNCPAINTTSLPLADFNNTISQCVDHQRQAALDNLLSRS

>K9S39_RS12985 | TM

ANALSAGNQLPFKIVGAKQLEIYSSTCPGLTAVGDIPQLNSEVSQCLLHQRAAALDTLLQR

>HUT13_RS22940 | TM

AEALHKGNALPFLITSDTRVQITSDSCQGISGNVTSDQFKDWLAGCVDIQRDVALKALLRRS

>OIE73_RS29030 | TM

ANALNVGSDLPFEIISGQVRSDICRLRSAQLPADELNAALNACVNKQRQHALDNLLSRS

>DWG14_RS11080 | TM

AQALNVGSKLPFEITTGSVTSDICRNLPSSGTSEVMNRAINACVNDQRQHALDDLLSRS

>J2N69_RS27985 | TM

QALNVGSDLPFRVTGKSSVTSDSCANFRLLPQRPTEAQLNAALNECVNTMRQNALDNLLSRS

>DWB77_RS09870 | TM

QALHAGSAPFKIMGGQNITLSSGSCQLPASGTFQEINDAIGVCMAQQRQHALDNLLSRS

>RFN58_RS31475 | TM

QALNVGSELPFKIVEGKVTSEICNLPDQASPAEFNNAMNHCVNEQRQNALDNLLSRS

>CEB94_RS30235 | TM

QALNVGSELPFKIVEGKVTSDICNLPGQASPSEFNDAMNACVNEQRRNALDNLLSRS

>KI385_RS32920 | TM

ADALHDGSALPLKILGGKFQSTSDICDLPTETSGQLLQEAVNQCLQHQRAVALNSLLNRS

>RI060_RS10320 | TM

EALNVGSELPFKIVEGKVTSDICNLPSQASPAEFNHAMNQCVNDQRKAALDNLLSRS

>V8J11_RS28500 | TM

DAIRRGSEFPLQIVRIDYKPSDTCHLPSQGNNDAFNRAVAQCMQQQRDYALDGLLRRS

>CP970_RS10850 | TM

QALNVGSDLPFRIEGGSKVSSQTCHNLPAEARIPADQLNSVLNDCVNDLRQNALDNLLSRS

>NNW98_RS08465 | TM

QALNVGSELPFKIVEGKVTSDICNLPGQASPSEFNEAMNRCVNDQRQHALDNLLSRS

>G9U55_RS24385 | TM

QAVHVGNELPFQVTNGTFTIYSDACPALSGRRFTSSGAFNDAMKLCIDHQRQQALDDLLSRS

>OHB09_RS25185 | TM

QALRTGSEPLFKIVEFNDLKVTSNDCPGVNNKLSLTEFNDAISACTDHQRQVALDHLLSRS

>SLLC_RS08245 | TM

QALREGNALPFKIVGGQKVEVSSTTCSGVGIDQSIDQFNAAIGQCILDQRKHALDDLLRR

>HA039_RS07905 | TM

QALHVGSKLPFTLLSGAVSSDTCRLPAQLTPDEANAAINSCVNHQRAQALDDLLTRS

>BN2145_RS10410 | TM

QALNVGSDLPFKIVEGKVTSDVCNLPDQASPSTFNSAMNDCVNEQRQHALDSLLSRS

>P8A22_RS09170 | TM

QALHVGSELPFKIVDGHVSSKVCNLLGEGRSPDSVNAAMNSCVNHQRQQALDTLLNRS

>JEQ17_RS13195 | TM

QALNVGSDLPFRIVSGGVTSPTCNFPSEPSATELNNAMNACVNEQRQHALDNLLSRS

>LGI35_RS32270 | TM

QALNVGSDLPFTVTNGSVSSGTCRNFSGLPDHPTTGQLNSALNECVNEMRQHALDNLLSRS

>BJ961_RS09055 | TM

QAVRTGNEPLYKIVDFTDLKVSSSTCPVVDNGNLSLSDFNAAISDCMDHQRKVALDNLLSRS

>SLCG_RS11660 | TM

QALTSGNQPPFKIAGGENIKVISDKCPAVNGATDLPLDTFNTIISKCIDQQRHASLDNLLSRS

>BXT88_RS28370 | TM

ADALHDGSALPLKILGGKFESTSDICDLPTQTSGPLLQQAVEQCLQHQRALALNSLLNRS

>HUT09_RS27310 | TM

QGLHVGNELPFKLLPDSKIQLTSNACPALTPGLSADEANAALKACNGEQRQQALDTLLNRS

>CAG99_RS03920 | TM

RALERGTELPFTFPPGTTTVQITSPVCPASAGPVEGEQFERWLAECTDKQRAIALDQLLRSS

>SLUN_RS29760 | TM

QALHIGSELPFKIVNGSVKSDICNFPSEAPPEQFNAAMNACVNEQRQHALDDLLRRS

>K7I03_RS07765 | TM

AADAMREGSYLPFRFIDGNLESTSNACPSLRPGRIDARELENAVQVCMNHQRAIALNGLLRRS

>WEB32_RS25725 | TM

DALRQGNDLPFKILNAQVEFTSDTCSNLSSSRESGAFMRGLEQCMQTQRAMALSSLLKRS

>JGK51_RS09955 | TM

QAISTGNQPLFKIVSFQELKVSSDNCPAINTTSLPLADFNNTISQCVDHQRQVALDNLLSRS

>NLG24_RS14855 | TM

ADVLQGSSKLPFQIVKMDYSLTDPTCELPSQSSTTLFNEAVARCMNHQRALALDALLRRS

>STRNI_RS11980 | TM

ADALHDGSALPLKILGGKFQSTSDICDLPTETSGQLLQEAVNSCLQHQRAVALNSLLNRS

>CP967_RS07360 | TM

QALDVGSDLPFEVVNGQVSSEVCDLPTKATPEAFNDALNACANQQRDHALDTLLNRS

>OG275_RS29095 | TM

QAINEGSELALKVTGVNVQLTSPTCPGLNEAVNNDQLNSSLKACMAAQRQQALDDLLTRS

>DC008_RS26455 | TM

QALRTGNEPLFKIVQFQSLKVTSNNCPGIATSDLSLAEFNDAINACMDHERKAALDNLLSRS

>JYK04_RS31245 | TM

QALRQGNAIPFTIVRGQNIEVTSTTCSGVSGINQPFEQFTAAINQCVLEQRRHALDDLLSR

>FYC74_RS09870 | TM

QAVRSGNQPLYKIVDFTDLRVSSSDCPVVDNGNLSLSDFNAAISDCMDHQRKVALDNLLSRS

>JNO44_RS31180 | TM

AAAALHDGSALPLKILGGKFQSTSDICDLPTETSGALLQEAVNNCLMHQRAVALNNLLNRS

>J0917_RS09775 | TM

HALNVGSQLPFRITSGTVVSNTCNFPSQTTASDLNNAMNACVNEQRQHALDNLLSRS

>DVK44_RS26935 | TM

QALHVGSELPFKIVSGKVSSDVCNFPDSAQPADFNIAMNACVNHQRQQALDDLLSRS

>Spa2297_RS24835 | TM

QAVRTGNEPLYKIVDFTDLRVSSSDCPVVDNGNLSLSAFNAAISDCIDHQRKVALDNLLSRS

>F9278_RS36905 | TM

QAVSTGNTPVFKIEGGTNISVSSNICPAVDADTAPTNLQLDDFNRAIAACIDHERQVALDTLLSRS

>DXZ73_RS31040 | TM

YLLAAHALDAGSTPLIKLNNGSQVTVPDSSLCPALRPGLTADEANNVLSNCTSHLRALALDSLLKR

>CP981_RS28955 | TM

ADALHDGSALPLKILGGKFESTSDICDLPTQTSGQLLQEAVNSCLQHQRAVALNSLLNRS

>LK06_RS25455 | TM

QAISTGNQPLFKIVSFQELKVSSDSCPAINTKSLALSDFNDAIAHCVDQQRQAALDNLLSRS

>CP972_RS26510 | TM

ANALNVGSDLPFEILSGQVRSDICQLRSAQLPAGELNAALNDCVNEQRQHALDNLLSRS

>NFX46_RS09135 | TM

ANALNVGSDLPFKILAGQVASDICNFPNEPTASELNRAMNECANQQRQHALDDLLSRS

>SPRI_RS09970 | TM

QALHVGVADLPFKIVEGKVQPTTDWCTLPEEGSGEQFNQAVSACLQHQRELALDDLLRR

>OHO81_RS12125 | TM

AQALNVGSELPFKITTGRVASDVCNLPTDVPADVMNKAMDSCVNDQRQHALDNLLSRS

>A4E84_RS30100 | TM

QALNVGSELPFKIVEGKVTSDICNLPDQASPAEFNNAMNHCVNEQRQNALDNLLSRS

>HED23_RS09090 | TM

QALDVGSDLPFEVVNGQVTSEICDLPVKASPDDFNAALNVCANQQRDHALDDLLNRS

>FGW37_RS24395 | TM

AADALTQGNQLPFRILNANVETSASCRSLPQGSATNSDTFMNGLRLCMEHQRAMALDGLLKRS

>SRIM_RS30980 | TM

ANALQEGSELPVKILRGNFAATSNVCNLPNESSGEIFTQAIQSCLQHQRAQALNTLLNRS

>P7W03_RS26950 | TM

QAVRTGNQPLYKIVDFEDLQVASTDCPGVTNGNLSLSQFNAAISDCIDHQRQVALDRLLSRS

>FQU76_RS26645 | TM

QALHEGSTLPFKIISGKFQETMSGCNLPADGTNEEFNTALAQCFAHQRELALNDLLRRS

>F0345_RS22855 | TM

QAVHVSNELPFQVTKGTFTIYSDACPALSGRRFTSSGAFNDAMKLCIDHQRQQALDDLLSRS

>OG949_RS08815 | TM

QALHVGSELPFKIVTGKVSSDVCNFPDQAPPEEFNAAMNACVNHQRQQALDDLLSRS

>SCAB_RS11380 | TM

QAVSTGNTPVFKIEGGDNISVSSALCPTVDANTAPTNLKLDDFNAAISACIDDHRQKALDTLLSR

>IAG44_RS09910 | TM

QAINTGNEPPFKIVTGNSMRVTSSSCPAVNAATNADGEVYLSTFNAAISACVDERRQDSLDNLLSR

>I1A49_RS32535 | TM

QALHVGNELPFKLVGGSVQPTNNTCPEIIGQSSPDQFNAVLNTCMKEQRQLALDGLLRRS

>LXH13_RS29530 | TM

QALHEGSGQTFQLSGTNLDISSSTCPKLNQATSNDQFNSILASCEAVQRQHALDDLLSRS

>D0Z67_RS22315 | TM

QAISTGNQPLFKIVGYQDLRVSSSNCPAINNTTSLSLQDFNTAISQCVDHQRQVALDNLLSRS

>CP982_RS30995 | TM

QALNVGSELPFKIVSGRVESKVCDLPSRASPDEFNAAMNACVNEQRQHALDNLLSRS

>CP968_RS08665 | TM

QALREGNALPFKIVSGVEVRVTSSTCPGVGGEGQPLDQFNAAINACVLEQRRHALDDLLSR

>DDQ41_RS24655 | TM

QALHDGIRQSVEVGAAPGANVTITSPTCPRINDLVDNSERNAALKLCIAEQRQRALDELLTRS

>OG288_RS12590 | TM

QALHEGSGVDFRVTGSNLELTSNTCPQLSGAGNNDELNAMLKECTAVQRQHALDGLLSRS

>LDH80_RS11000 | TM

TAQALSDSTSQLPFKVVNGTVQPTTSWCRLPESPTGDQLNDAVTLCLRHQSDLALDDLLRR

>B7R87_RS26840 | TM

QALHDGSNSNLPFKIISGKFQETTSGCNLPADGTSEEFTSALTQCFAHQREVALNDLLRRS

>DDW44_RS23595 | TM

QALHDGIRQSVEVGAAPGANVTITSPTCPRINDLVDNGQRNAALKLCIAEQRQRALDELLTRS

>IGS69_RS26470 | TM

QALNVGSDLPFKIVEGKVTSDICNLPDQASPSEFNNAMNACVNEQRRHALDSLLSRS

>MMF93_RS25415 | TM

QAMDNLSELPFRILSGKTQPTSDRCPGLTGTLPSDAFMDRLSACLDAQRRIALDGLLRRS

>vnz_RS27635 | TM

QALSDSTSQLPFKVVNGTVQPTSAICQLPVNPTGDQLNDAVTLCLRHQSDRALDDLLRRS

>FDM97_RS25645 | TM

QVLNVSVSELPFKVVTGQVQPTTSWCRLPESGTGEQLNEAVSACLQHQRDLALDDLLRR

>SVTN_RS28325 | TM

YLFTAQALSDSTSQLPFKVVNGTVQPTTSWCTLPESPTGDQLNNAVTLCLRHQSDIALEDLLRR

>OG711_RS10260 | TM

QAIGVGNKMPFTIVSGELQVSSQSCPDLADTPRTQDAINDALAQCLNHRRQQALDELLSR

>R2E43_RS09305 | TM

QAVRTGNEPLYKIVDFTDLKVSSSTCPVVDNGGLSLSDFNAAISDCMDHQRKVALDNLLSRS

>CP969_RS28190 | TM

QALHDGSGQSFKVIGTNITITSETCPQLQGATDNSQLNEMLKQCNAVQRQHALDDLLSRS

>OG542_RS10060 | TM

EALNVGSELPFKIVEGKVTSDICNLPSQASPAEFNHAMNQCVNDQRKAALDNLLSRS

>SXIN_RS07295 | TM

AEALDKGNALPFKLLEGSFRPTSSTCPNLTDRTFENHEEFNAALEVCMDYQRQLALDNLLRRS

>CP980_RS08265 | TM

QALREGNGLPFKIVGGTDIQVTSTCPGVVGKGQKYEQFNDVINTCILEQRRHALDDLLSR

>KPP03845_RS27990 | TM

QALREGNALPFTIVSGGPIQVTSTTCPGLANVNGNGMLTSEAFQSAISQCALDQRRHALDDLLSR

>SXIM_RS22710 | TM

AEALHKGNALPFLITSDTRVQITSDSCQGISGNVTSDQFKDWLSGCVDIQRDVALKALLRRS

>IAG42_RS08425 | TM

QALNVGSELPFKIVEGTVSSSVCNIPDGTKLPASELNSALNDCVAQQRNHALDVLLSR

>J8403_RS12780 | TM

QALHVGNELPFKLVGGSVQPTNNTCPEIIGQSSPDQFNAVLNTCMKEQRQLALDGLLRRS

>MOV08_RS12175 | TM

AAAALHDGSALPLKILGGKFQSTSDICDLPTETSGALLQEAVNNCLMHQRAVALNNLLNRS

>NRK68_RS25920 | TM

QALRQGNALPFQIVGGQVKVTSSSCPGVVGLELTPDQFNAAIGQCILEQRRHALDDLLSR

**Supplementary Information 1.4.** Protein sequences used to generate the alignment in Supplementary Figure 4

>OG362_RS07370 Kitasatospora_papulosa_strain_NBC_01269.gbff Streptomyces [Kitasatospora] papulosa recipro:1 MAASPAPMKAPPKPTWEPKAQESPYPWLRPTIRIRLTLLYGGMFLIAGILLLSIIYMLAA QALHDAGGLEFQVKGTNVQISSDMCPQLASALNNTQLNEMIKDCTAAQRQHALDSLLNRA LLALVGLSVIAFAFGYAMAGRVLSPLGRITRTARRVAGTDLTRRIELDGPDDELKELADT FDEMLDRLERAFTAQQRFVGNASHELRTPLAINRTLLEVHLSDPGAPPELQQLGKTLLAT NERSEQLVEGLLLLARSDNQIVERKPVDLAEVADRAIDQTRSEAADKGVRILGERASAVV QGNGVLLERIALNLVQNAVRYNVAEDGWVEVTTELQHGQALLVVSNTGPVVPAYEIDNLF EPFRRLRTERTGSDKGVGLGLSIARSVARAHGGRIIAEPREGGGLVMRVTLPV  
>XNR_RS05000 Streptomyces_albidoflavus.gbff Streptomyces albidoflavus recipro:1 VAASPAPRPAPPKPTSAPPRPTWDPRRPPQQAWLRPTIRIRLTLLYGGMFLIAGILLLSI IYLLAAQAVHVGNELPFQVTSGTFTIYSDACPALSGRRFTSSGAFNDAMKLCIDHQRQQA LDDLLSRSLLALVGLSVIAFAFGYAMAGRVLAPLGRITRTARSVAGSDLSRRIELDGPDD ELKELADTFDDMLDRLERAFTAQQRFVANASHELRTPLAINRTLLEVQLSDPGAPPELQQ LGKTLLATNERSEQLVEGLLLLARSDNQIVERKPVDLGEVATRAVEQVRGEAEQREVELR ADIAEAVVQGNGVLLERIALNLTQNAVRYNLPPGEGGWVEVSTELQHGQAVLVVTNTGPV VPAYEIDQLFEPFRRLRTERTGSDKGVGLGLSIVRSVTRAHGGRITAEPREGGGLVMRVT LPL  
>CP975_RS26985 Streptomyces_alboniger_strain_ATCC_12461.gbff Streptomyces alboniger recipro:1 VAATPAPPAAPPKPTWDPRKVEAPFPWLRPTIRIRLTLLYGGMFLIAGILLLSIIYLLAA QALNVGTDLPFTIEGGSKVSSDTCHNLPTGTQIPAEQVNAALNECVNQVRRNALDNLLSR SLLALLGLAVIAFAFGYAMAGRVLSPLGRITRTARRVAGTDLTRRIELDGPDDELKELSD TFDEMLDRLERAFTAQQRFVGNASHELRTPLAINRTLLEVHLSDPGAPPELQQLGKTLLA TNERSEQLVEGLLLLARSDNQIVERKPVDLAEVADRAVDQVRAEADAKAVEIRGERGPAV VQGNGVLLERIALNLVQNAVRYNVPDEGWVEVTTEAQHGQAVLVVSNTGPVVPAYEIDNL FEPFRRLRTERTGSDKGVGLGLSIARSVARAHGGRIIAEPREGGGLVMRVTLPI  
>K1J60_RS11550 Streptomyces_akebiae_strain_MG28.gbff Streptomyces akebiae recipro:1 MATTPAPPLAPPKPTWDPRRPQNPLPWLRPTIRIRLTLLYGGMFLIAGILLLSIIYLLAA QAVNTGNAPLFKIVGGTDISVTSEKCPAVAGSTNVRLADFNAAISDCIDAQRQVALDTLL SRSLLALLGLAVIAFAFGYAMAGRVLAPLGRITRTARAVAGSDLSRRIELDGPDDELKEL ADTFDEMLERLQRAFTAQQRFVGNASHELRTPLAINRTLLEVHLSDPNAPTELQQLGKTL LATNERSEQLVEGLLLLARSDNQIVERKPVDLAEVASQAIDQVRSEADAKKVEIRGERAP AVVQGNGVLLERIALNLVQNAVRYNVQGAGGWVEVTTELQHGQALLVVTNTGPVVPAYEI DNLFEPFRRLRTERTGSDKGVGLGLSIVRSVARAHGGHIAARPREGGGLVMRVTLPV  
>K7396_RS09365 Streptomyces_angustmyceticus_strain_JCM_4053.gbff Streptomyces angustmyceticus recipro:1 MPSLPSFSKAATPPPPVPPKPTWDPRPVDVRPFPWLRPTIRIRLTLLYGGMFLMAGIVLL TIIYMLAADALHDGSALPLKILGGKFQSTSDICDLPTETSGQMLQQAVEQCLQHQRALAL NSLLNRSLLALLGLTVVAFAFGYAMAGRVLSPLGRITRTAQRVAGSDLHRRIELGGPDDE LKELADTFDEMLDRLDRAFESQRRFVSNASHELRTPLAINRTLLEVQLADPGASAELVQL GRTLLATNERSEQLVEGLLLLARSENKVVDRRPVDLAEVASQAVDQTRAEALAAGVELRG VREPVVVQGSGVLLERIALNLVQNAVRYNVADGGWVEVSTRARPGCAVLVVSNTGPVVPA YEVENLFEPFRRLRTERTGSDKGVGLGLSIVRSVVRAHDGTITAVPREGGGLDMRVVLPL  
>NR995_RS07620 Streptomyces_albus_strain_DSM_40763.gbff Streptomyces albus recipro:1 MPPSTSATGTDWGGNGTPAAPRTPPKPNWDPRDAQRPSPWLRPTIRIRLTLLYGGMFLIA GVLLLTIIYLLAAQALDQISELPFRILSGKTQPTSDRCPGLTGTLPSDVFTDRLSACLEA QRGIALDGLLRRSLLALVGLAVAAFAFGYVMAGRVLSPLGRITRTARQVAGSDLHKRIEL EGPDDELKELADTFDEMLDRLNRAFTAQQRFVANASHELRTPLAINRTLLEVQLADPEGS PELQQLAKTLLATNERSEQLVEGLLLLARSENELVDRKPVDLAEVASQAVEQARGEAETK GVELRGVRQPTYVQGNGVLLERVALNLVQNAVRYNLRDGGWVSVSTEAQPGQAVLVVENT GPVVPAYELDNIFEPFRRLRTERTGSDKGVGLGLSIVRSVARAHGGSVAAVPREEGGLVM RVTLPL  
>SAM23877_RS26430 Streptomyces_ambofaciens_ATCC_23877.gbff Streptomyces ambofaciens ATCC 23877 recipro:1 VATTPAPPGAPPKPTWAPRSAAPLPWLRPTIRIRLTLLYGGMFLIAGILLLSIIYLLAAQ AVRTGNQPLYKIVDFEGLRVASNDCPGVTNGNLSLSEFNAAISECIDHERQAALDRLLSR SLLALLGLAVIAFAFGYAMAGRVLSPLGRITRTARAVAGSDLSRRIELDGPDDELKELAD TFDDMLERLQRAFTAQQRFVGNASHELRTPLAINRTLLEVHLSDPNAPVELQQLGKTLLA TNERSEQLVEGLLLLARSDNQIVERKPVDLAEVAGQAIDQVHAEAGTKGVEVRGAREPAV VQGNGVLLERIALNLVQNAVRYNVPEQGWVEVTTAVENGQAVLVVTNTGPVVPAYEIDNL FEPFRRLRTERTGSDKGVGLGLSIARSVARAHGGHIYAQPREGGGLVMRVTLPV  
>H9W91_RS28360 Streptomyces_alfalfae_strain_XN-04.gbff Streptomyces alfalfae recipro:1 VASTPAPPTAPPKPTWDPRKVEPPFPWLRPTIRIRLTLLYGGMFLIAGILLLSIIYLLAA QALNVGSELPFKIVEGRVASTVCNLPPNPSADDLNRAMGACVNEQRQHALDNLLSRSLLA LLGLAVIAFAFGYAMAGRVLSPLGRITRTARRVAGTDLSRRIELDGPDDELKELSDTFDE MLDRLERAFTAQQRFVGNASHELRTPLAINRTLLEVHLSDPGAPPELHQLGKTLLATNER SEQLVEGLLLLARSDNQIVERKPVDLAEVADRAVDQVLAEADAKGVEIRGERGAAVVQGN GVLLERIALNLVQNAVRYNVPGNEEGGWVEVTTEAQHGQAVLVVSNTGPVVPAYEIDNLF EPFRRLRTERTGSDKGVGLGLSIARSVARAHGGRIIAEPREGGGLVMRVTLPI  
>OIE72_RS09030 Streptomyces_anthocyanicus_strain_NBC_01777.gbff Streptomyces anthocyanicus recipro:1 VATTPAPPGAPPKPTWDPRSATPLPWLRPTIRIRLTLLYGGMFLIAGILLLSIIYLLAAQ AVRTGNEPLYKIVDFTDLKVSSSTCPVVDNGGLSLSDFNAAISDCMDHQRKVALDNLLSR SLLALLGLAVIAFAFGYAMAGRVLSPLGRITRTARAVAGSDLSRRIELDGPDDELKELAD TFDDMLERLQRAFTAQQRFVGNASHELRTPLAINRTLLEVHLSDPGAPVELQQLGKTLLA TNERSELLVEGLLLLARSDNQIVERKPVDLAEVAGQAIDQVHAEAESKGVEVRGTREAAV VQGNGVLLERIALNLVQNAVRYNVAGQGWVEVATAVENGQAVLVVTNTGPVVPAYEVDNL FEPFRRLRTERTGSDKGVGLGLSIARSVARAHGGHISAQPREGGGLVMRVTLPV  
>LK895_RS07485 Streptomyces_anulatus_strain_YINM00001.gbff Streptomyces anulatus recipro:1 MATTSEPPAAPPKPTWEPKQQEPPYPWLRPTIRIRLTLLYGGMFLIAGIVLLSIIYMLAA QALHVGTDLPFKLLPDSKIQLTNNACPALTPGLSADEANAALKACNESQRKHALDTLLNR SLLALVGLSVIAFAFGYAMAGRVLSPLGRITRTARRVAGTDLTRRIELDGPDDELKELAD TFDDMLDRLERAFTAQQRFVGNASHELRTPLAINRTLLEVHLSDPEAPPELQQLGKTLLA TNERSEQLVEGLLLLARSDNQIVERKPVDLAEVAERAIDQTRAEALAKKVEIRGERSTAV VQGNGVLLERIALNLVQNAVRYNVPEDGWVEVTTEARDGQALIVVSNTGPVVPAYEIDNL FEPFRRLRTERTGSDKGVGLGLSIARSVARAHGGRIIAEPREGGGLVMRVTLPV  
>EJC51_RS35435 Streptomyces_aquilus_strain_GGCR-6.gbff Streptomyces aquilus recipro:1 VATTPAPPQAPPKPTWDPRRPEPPFPWLRPTIRIRLTLLYGGMFLIAGILLLSIIYLLAA NALNVGSDLPFKVVTGTVSSESCNITSTQLPAPDLNHALNECVNEQRQHALDNLLSRSLL ALLGLAIIAFAFGYAMAGRVLSPLGRITRTARAVAGSDLSRRIELDGPDDELKELADTFD DMLERLQRAFTAQQRFVGNASHELRTPLAINRTLLEVHLSDPGAPVELQQLGKTLLATNE RSEQLVEGLLLLARSDNQIVERKPVDLAEVAEQAVDQVHAEAEAKGVEIRGERLSAVVQG NGVLLERIALNLVQNAVRYNVPNEEAQREALPNKGGGGRREGGWVEVTTEVQHGQAVLVV SNTGPVVPAYEIDNLFEPFKRLRGADRTGSDKGVGLGLSIVRSVARAHGGHIYAQPREGG GLVMRVTLPI  
>QR300_RS12510 Streptomyces_antimycoticus_strain_FIM95-F1.gbff Streptomyces antimycoticus recipro:1 MATTPTPLPPTAPPKPSWDPQGASRPNPWLRPTIRIRLTLLYGGMFLIAGVVLLTIIYLL AAQALHVGNELPFKLVGGSVQPTNNTCPEIIGQSSPDQFNAVLNTCMKEQRQLALDGLLR RSLIALLGLSVIAFAFGYAMAGRVLSPLGRITRTARQVAGSDLSRRIELDGPDDELKELA DTFDEMLERLDRAFTAQQRFVANASHELRTPLAINRTLLEVQLSDPQASPELVQLGKTLL ATNERSEQLVEGLLLLARSDNEIVDRKPVDLAEVASQAMEQVRAEAEGKGVELRGQRAPA VVQGNGVLLERIALNLVQNAVRYNIAEDGWVEVTTESRPGQAVLVVANTGPVVPAYEMDN IFEPFRRLRTERTGSDKGVGLGLSIARSVARAHGGRIAAEPREGGGLVMRVVLPV  
>HKX69_RS08265 Streptomyces_argyrophyllae_strain_Jing01.gbff Streptomyces argyrophyllae recipro:1 VATTPAPPQAPPKPTWDPRRPQPPFPWLRPTIRIRLTLLYGGMFLIAGILLLSIIYLLAA QAISTGNQPLFKIVSGSAIKVTSDNCPAVTAINSGPVPLSDFNEAIAHCVDQQRQAALDN LLSRSLLALLGLAVIAFAFGYAMAGRVLSPLGRITRTARQVAGSDLSRRIELDGPDDELK ELADTFDDMLERLERAFTAQQRFVGNASHELRTPLAINRTLLEVHLSDPNAPVELQQLGK TLLATNERSEQLVEGLLLLARSDNQIVERKPVDLAEVATQAVDQVHAEADAKGVKIRGER KPAVVQGNGVLLERIALNLVQNAVRYNVPEDGWVEVDTEVEHGQAVLTVTNTGPVVPAYE IDNLFEPFRRLRTERTGSDKGVGLGLSIARSVARAHGGHILARPREGGGLVMRVTLPL  
>IGS71_RS09820 Streptomyces_aurantiacus_strain_JCM_4677.gbff Streptomyces aurantiacus recipro:1 MTTTPAPPKAPPKPTWDPRKPDPPFPWLRPTIRIRLTLLYGGMFLIAGILLLSIIYLLAA QALHEGSGVDFRVTGSNLELTSGTCPQLSGASSNEELNAMLKECTAAQRQHALDGLLSRS LMALLGLAVIAFAFGYAMAGRVLSPLGRITRTARAVAGSDLSRRIELDGPDDELKELADT FDDMLERLQRAFTGQQRFVGNASHELRTPLAINRTLLEVHLSDPGAPVELQQLGKTLLAT NERSEQLVEGLLLLARSDNQIVERKPVDLAEVASQAIDQVRGEAEAKGVEVRGERAAAVV QGNGVLLERIALNLLQNAVRYNVPDDGWVEVVTGVQHGQAVLTVANTGPVVPAYEIDNLF EPFRRLRTERTGSDKGVGLGLSIARSVARAHGGHIVAEPREGGGLVMRVALPI  
>SU9_RS26335 Streptomyces_auratus_AGR0001.gbff Streptomyces auratus recipro:1 MPSLPSFSKAAAPPPPLPPKPTWDPRPVNVRPFPWLRPTIRIRLTLLYGGMFLMAGIVLL TIIYMLAADALHDGSALPLKILGGKFQSTSNLCDLPTKTSGPLLQEAVEQCLQQQRALAL NSLLNRSLLALLGLTVVAFAFGYAMAGRVLAPLGRITRTAQRVAGSDLHRRIELGGPDDE LKELADTFDEMLDRLDRAFESQRRFVSNASHELRTPLAINRTLLEVQLADPDASPELVQL GKTLLATNERSEQLVEGLLLLARSENKVVDKKPVDLSEVAAQAVDQSREEAHSKGVELRG VRQQVFVQGNGVLLERIALNLVQNAVRYNVPEDGWVEVATEPQPGCAVLVVANTGPVVPA YEVENLFEPFRRLRTERTGSDKGVGLGLSIVRSVVRAHDGTITASPREGGGLVMRVVLPL  
>SAVERM_RS12550 Streptomyces_avermitilis_MA-4680.gbff Streptomyces avermitilis MA-4680 = NBRC 14893 recipro:1 VATTPAPPAAPPKPTWDPRRQEAPFPWLRPTIRIRLTLLYGGMFLIAGILLLSIIYLLAA QALRTGSEPLFKILDGNGIKVASDDCPGVSTNNIPLTAFNDAISACTDHQRQVALDNLLS RSLLALLGLAVIAFAFGYAMAGRVLSPLGRITRTARAVAGSDLSRRIELDGPDDELKELA DTFDDMLERLQRAFTAQQRFVGNASHELRTPLAINRTLLEVHLSDPGAPMELQQLGKTLL ATNERSEQLVEGLLLLARSDNQIVERKPVDLAEVASQAIDQARSEADAKGVEFRGERASA VVQGNGVLLERIALNLVQNAVRYNVPEDGWVEVTTEVQHGHAVLVVSNTGPVVPAYEIDN LFEPFRRLRTERTGSDKGVGLGLSIARSVARAHGGHIAAQPREGGGLVMRVTLPI  
>C5746_RS31245 Streptomyces_atratus_strain_SCSIO_ZH16.gbff Streptomyces atratus recipro:1 MPTVPAPPTAPPKPTWEPKQQEPPYWLRPTIRIRLTLLYGGMFLIAGILLLAIIYMLAAQ ALQVGSELPFKIVSGQVTSDVCNFHGITTPDAANAAMNTCVNHQRQQALDTLLNRSLLAL VGLSVIAFAFGYAMAGRVLSPLGRITRTARQVAGTDLSRRIELDGPDDELKELADTFDEM LDRLERAFTAQQRFVGNASHELRTPLAINRTLLEVHLSDPQVPPELKQLGKTLLATNERS EQLVEGLLLLARSDNQIVERKPVDLAEVASRAIDQTRGEAEERGVEIRGERAPAVVQGNG VLLERIALNLVQNAVRYNVPEGGWVEVTTEPRSGQAVLVVSNTGPVVPAYEIDNLFEPFR RLRTERTGSDKGVGLGLSIARSVARAHGGRIVAEPREGGGLVMRVSLPV  
>GQF42_RS32020 Streptomyces_broussonetiae_strain_T44.gbff Streptomyces broussonetiae recipro:1 VAATPAPAQAPPKPTWDPRRPQAPFPWLRPTIRIRLTLLYGGMFLIAGILLLSIIYLLAA QAISTGNQPLFKIVSFQSLRVSSDNCPAITTTSLSLQEFNDAISQCVDHQRQAALDDLLS RSLLALLGLAVIAFAFGYAMAGRVLSPLGRITRTARQVAGSDLARRIELDGPDDELKELA DTFDDMLERLQRAFTAQQRFVGNASHELRTPLAINRTLLEVHLSDPNAPAELQQLGKTLL ATNERSEQLVEGLLLLARSDNQIVERKPVDLAEVATQAVDQVHAEAQAKGVEIRGRRKPA VVQGNGVLLERIALNLVQNAVRYNVPEDGWVEVATEVEHGQAVLTVSNTGPVVPAYEIDN LFEPFRRLRTERTGSDKGVGLGLSIVRSVARAHGGHIVARPREGGGLVMRVTLPL  
>H7H31_RS07020 Streptomyces_buecherae_strain_AC541.gbff Streptomyces buecherae recipro:1 MAATPPPTPPLPTAPPKPHWSPSELGRPLPWLRPTIRIRLTLLYGGMFLMAGILLLSIIY LLAADALHKGNELPFQIINADYRATSDVCDLPDRGNSAQFNHAVAQCMQGQREMALDGLL RRSLLALLGLTVVAFAFGYAMAGRVLSPLGRITRTARQVAGSDLSRRIELGGPDDELKEL SDTFDEMLERLGRAFTAQQRFVANASHELRTPLAINRTLLEVQLSDPDASPELTQLGKTL LATNERSEQLVEGLLLLARSDNEIVDRKPVDLAEVATQAVDQARTEAQAKGVELRGERQP AVLQGNGVLLERVALNLVQNAVRYNAPDGWVRVDTVLERAEAVLVVSNTGPVVPAYEVDN IFEPFRRLRTERTGSDKGVGLGLSIVRSVVRAHGGRITAVPREGGGLVIQVSFPV  
>G4Z16_RS06790 Streptomyces_bathyalis_strain_ASO4.gbff Streptomyces bathyalis recipro:1 MAASTTPSEPAEHRRQTPGPASAPPKPTWDPGEPSRPYPWLRPTIRIRLTLLYGGMFLIA GMVLLTLIYLLAAEALHEGSKLPFRILNANAQLTSDSCPGLTGTLPSEQFMERLGECTDA QRAVALDELLQRSLMALLGLAVAAFAFGYVMAGRVLSPLGRITRTARQVASSDLHKRIEL EGPDDELKELSDTFDDMLDRLDRAFTAQQRFVANASHELRTPLAINRTLLEVQLSDPEAS PELQQLGKTLLATNERSEQLVEGLLLLARSENEIVDRKPVDLSEVAAHAVEQVRAEAESK GVEVRGVRQPVYVQGNGVLLERVALNLVQNAVRYNQQEGGWVSVATEALPGHGMLLVENT GPAVPAYEVDNLFEPFRRLRSDRTGSDKGVGLGLSIVRSVARAHGGTVTAERREEGGLAL RVVLPV  
>CD934_RS07910 Streptomyces_calvus_strain_DSM_41452.gbff Streptomyces calvus recipro:1 MAATPAPPTAPPKPTWDPKKPEPPFPWLRPTIRIRLTLLYGGMFLIAGILLLSIIYLLAA QALHDGGSGQSFQVTGTNIDITSETCPQLDTATTNSQLNDMLKQCNAAQRQHALDDLLSR SLGALLGLAIIAFAFGYAMAGRVLSPLGRITRTARAVAGSDLSRRIELDGPDDELKELAD TFDDMLERLQRAFTAQQRFVGNASHELRTPLAINRTLLEVQLSDPNAPVELQQLGKTLLA TNERSEQLVEGLLLLARSDNQIVERGPVDLAEVASQAIDQVHGEAEAKGVDIRGHRAPAV VQGNGVLLERIALNLVQNAVRYNVPEGGWVEVTTEVQHGQAVLVVSNTGPVVPAYEIDNL FEPFRRLRTERTGSDKGVGLGLSIVRSVARAHGGHIYARPREGGGLVMRVTLPI  
>DN051_RS11225 Streptomyces_cadmiisoli_strain_ZFG47.gbff Streptomyces cadmiisoli recipro:1 VAANPTPAQAPPKPTWDPRKPDPFPLLRPTIRIRLTLLYGGMFLIAGILLLSIIYLLAAN ALNVGSDLPFKILSGQVASETCNFPSQPTATELNHAMNECVNEQRQHALDNLLSRSLLAL LGLAVIAFAFGYAMAGRVLSPLGRITRTARAVAGSDLSRRIELDGPDDELKELADTFDDM LERLQRAFTAQQRFVGNASHELRTPLAINRTLLEVHLSDPGAPVELQQLGKTLLATNERS EQLVEGLLLLARSDNQIVERKPVDLAEVASQAVDQVHAEAQAKGVEIRGKREPAVVQGNG VLLERIALNLVQNAVRYNVPEGGWVEVTTELQHGHAVLVVANTGPVVPAYEIDNLFEPFR RLRTERTGSDKGVGLGLSIVRSVARAHGGHIVAQPREGGGLVMRVTFPI  
>OG436_RS28770 Streptomyces_caniferus_strain_NBC_00270.gbff Streptomyces caniferus recipro:1 MPSLPSFSKAATPPPPVPPKPTWDPRPVDVRPFPWLRPTIRIRLTLLYGGMFLMAGIVLL TIIYMLAADALHDGSALPLKILGGKFQSTSDICDLPTETSGQLLQQAVEQCLQHQRAVAL NSLLNRSLLALLGLTVVAFAFGYAMAGRVLSPLGRITRTAQRVAGSDLHRRIELGGPDDE LKELADTFDEMLDRLDRAFESQRRFVANASHELRTPLAINRTLLEVQLADPGASPELAQL GKTLLATNERSEQLVEGLLLLARSENKVVDKKPVDLSEVASQAVDQTREEAQAKGVELRG VRSRVVVQGNGVLLERIALNLVQNAVRYNVPEEGWVEVSTEPAPGCAVLVVANTGPVVPA YEVENLFEPFRRLRTERTGSDKGVGLGLSIVRSVVRAHDGSITAVPREGGGLVMRVVLPL  
>I6J39_RS27535 Streptomyces_californicus_strain_FDAARGOS_1209.gbff Streptomyces californicus recipro:1 MATTPEPPAAPPKPTWEPKQHEPPYPWLRPTIRIRLTLLYGGMFLIAGIVLLSIIYMLAA QALHDAGSLELKVGGTNVLIASDTCPQLSSATNIDQVNETIKACTAAQRQHALDTLLNRS LLALVGLSVIAFAFGYAMAGRVLSPLGRITRTARRVAGTDLTRRIELDGPDDELKELADT FDDMLDRLERAFTAQQRFVGNASHELRTPLAINRTLLEVHLSDPGAPPELHQLGKTLLAT NERSEQLVEGLLLLARSDNQIVERKPVDLAEVAERAIDQTRTEAVEKGVEIRGERGSAVV QGNGVLLERIALNLVQNAVRYNVPEDGWVEVATEVRHGQALLTVSNSGPVVPAYEVDNLF EPFRRLRTERTGSDKGVGLGLSIARSVARAHGGRIIAEPREGGGLVMRVTLPV  
>PYS65_RS09315 Streptomyces_cathayae_strain_HUAS_5.gbff Streptomyces cathayae recipro:1 VATTPAPAPGSPQTPPKPTWDPRRPVPPFPWLRPTIRIRLTLLYGGMFLIAGILLLSIIY LFAANALKVGSDLPFRILSGQVSSDTCQLRSAQLPADELNRALNECVNEQRQHALDNLLS RSLLALLGLAVIAFAFGYAMAGRVLSPLGRITRTARAVAGSDLSRRIELDGPDDELKELA DTFDDMLERLQRAFTAQQRFVGNASHELRTPLAINRTLLEVHLSDPDAPVELQQLGKTLL ATNERSEQLVEGLLLLARSDNQIVERKPVDLAEVASQAIDQVHAEAQAKGVEIRGDHKPA VVQGNGVLLERIALNLVQNAVRYNVAEQGWVEVTTQVQHGQAVLVVTNTGPVVPAYEVDN LFEPFRRLRTERTGSDKGVGLGLSIVRSVARAHGGHIYAQPREGGGLVMRVTLPI  
>CVT27_RS26525 Streptomyces_cavourensis_strain_1AS2a.gbff Streptomyces cavourensis recipro:1 MAATPPPPTAPPKPTWEPKQQEPPYPWLRPTIRIRLTLLYGGMFLIAGILLLSIIYMLAA QALGVGSKLPFEIVSGRVASDICDLPTTPSPEAFNAAMNACVNNQRKEALETLLNRSLLA LVGLSIIAFAFGYAMAGRVLSPLGRITRTARRVAGTDLTRRIELDGPDDELKELSDTFDD MLDRLERAFTAQQRFVGNASHELRTPLAINRTLLEVHLSDPQAPPELQQLGKTLLATNER SEQLVEGLLLLARSDNQIVERKPVDLAEVADRAIDQARAEAVERNVEIRGERAGAVVQGN GVLLERIALNLVQNAVRYNVAEDGWVEVTTSVQPGQALLVVSNTGPVVPAYEIDNLFEPF RRLRTERTGSDKGVGLGLSIARSVARAHGGRIIAEPREGGGLVMRVTLPV  
>IHE65_RS10955 Streptomyces_caniscabiei_strain_ID03-3A.gbff Streptomyces caniscabiei recipro:1 MATTPAPPLAPPKPTWDPRRPQNPLPWLRPTIRIRLTLLYGGMFLIAGILLLSIIYLLAA QAVNTGNAPLFKIVGGTDISVTSEKCPAVDAGSTNVRLSDFNAAISACIDAQRQVALDTL LSRSLLALLGLAVIAFAFGYAMAGRVLAPLGRITRTARAVAGSDLSRRIELDGPDDELKE LADTFDEMLERLQRAFTAQQRFVGNASHELRTPLAINRTLLEVHLSDPNAPTELQQLGKT LLATNERSEQLVEGLLLLARSDNQIVERKPVDLAEVASRAIDQVRSEADAKKVELRGERA PAVVQGNGVLLERIALNLVQNAVRYNVQGAGGWVEVTTELQHGQALLVVTNTGPVVPAYE IDNLFEPFRRLRTERTGSDKGVGLGLSIVRSVARAHGGHIAARPREGGGLVMRVTLPV  
>IPT68_RS27775 Streptomyces_chromofuscus_strain_DSM_40273.gbff Streptomyces chromofuscus recipro:1 VAAHPAPPQAPPKPTWDPRKAAPPFPLLRPTIRIRLTLLYGGMFLIAGIMLLSIIYLLAA DAMNVGSELPFKILSGQVASETCNFPGSPTAAELNQAMNACVNEQRQHALDNLLSRSLLA LLGLAVIAFAFGYAMAGRVLSPLGRITRTARAVAGSDLSRRIELDGPDDELKELADTFDD MLERLQRAFTAQQRFVGNASHELRTPLAINRTLLEVHLSDPKAPVELQQLGKTLLATNER SEQLVEGLLLLARSDNQIVERKPVDLAEVASQAVDQVHAEADAKGVQIRGTREPAVVQGN GVLLERIALNLVQNAVRYNVPEGGWVEVTTEVQHGQAVLVVANTGPVVPAYEIDNLFEPF RRLRTERTGSDKGVGLGLSIVRSVARAHGGHIAARPREGGGLVMRVTFPI  
>CP977_RS26050 Streptomyces_cinereoruber_strain_ATCC_19740.gbff Streptomyces cinereoruber recipro:1 MATTPAPHPQAPPKPTWDPRDPVRPLLRPTIRIRLTLLYGGMFLIAGILLLSIIYLFTAQ ALSDSAAQLPFKVVDGTVQPTTSWCELPVKSTGDQLNDAVTLCLRHQGDRALEDLLRRSL FALLGLSIIAFAFGYAMAGRVLSPLGRITRTARQVAGSDLSRRIELDGPDDELKELADTF DEMLERLERAFTAQQRFVANASHELRTPLAINRTLLEVHLSDPGAPVELQQLGKTLLATN ERSEQLVEGLLLLARSDNQIIERKAVDLAEVAERGIDQVHAEAEAKGVEIRGERAPAVVQ GNGVLLERIALNLLQNAVRYNVPEGGWVEVATEVEHGRAVLLVSNTGPVVPAYEIDNLFE PFRRLRQERTGSDKGVGLGLSIARSVARAHGGRIMAEPREGGGLVMRVTLPI  
>OHT68_RS12785 Streptomyces_canus_strain_NBC_00277.gbff Streptomyces canus recipro:1 VASTPAPPQAPPKPTWDPRRPAPPLPWLRPTIRIRLTLLYGGMFLIAGILLLSIIYLLAA NALNVGSNLPFKIVSGGVTSDVCKLSGSELPADELNHALNQCVNDQRQHALDNLLSRSLL ALLGLAIIAFAFGYAMAGRVLSPLGRILRTARSVAGSDLSRRIELDGPDDEIKELADTFD DMLERLERAFTAQQRFVGNASHELRTPLAINRTLLEVHLSDPGAPVELQQLGKTLLATNE RSEQLVEGLLLLARSDNQIVERKPVDLAEVAEQAVDQVYGEAEAKGVAIRGDRKTAVVQG NGVLLERIALNLVQNAVRYNVPEDGWVEVTTEVQHGHAVLTVSNTGPVVPAYEVDNLFEP FKRLRGADRTGSDKGVGLGLSIVRSVARAHGGHITAQPREGGGLVMRVTLPV  
>NRO40_RS22695 Streptomyces_changanensis_strain_HL-66.gbff Streptomyces changanensis recipro:1 VTGTPTPPQAPPKPTWDPRIPARPLLRPTIRIRLTLLYGGMFLIAGVLLLSIIYLLTRQA LSISTDDLPFKLLKGEVEPNFDWCRLPVEGVTPDQFNDAMAACLQHQRDLAQDELLRRSL FALLGLSIIAFAFGYAMAGRVLSPLGRITRTARQVAGSDLSRRIKMAGPDDELKELADTF DDMLDRLERAFTAQQRFVANASHELRTPLAINRTLLEVHLSDPAAPVEMRELGKALLATN ERSEQLVEGLLLLARSENQIVERKPVDVAEVASRAIDQVRGEAEAKGVEIRGERAPVVVQ GNGVLLERVALNLVQNAVRYNVPEDGWVEVTTGVEQGRAVLVVSNTGPVVPAYEIDNLFE PFRRLREERTGSDRGVGLGLSIVRSVARAHGGRVTAEPREGGGLVMRVTLPQ  
>CP976_RS32095 Streptomyces_coeruleorubidus_strain_ATCC_13740.gbff Streptomyces coeruleorubidus recipro:1 VAATPAPPQAPPKPTWDPRRPAPPFPWLRPTIRIRLTLLYGGMFLIAGILLLSIIYLLAA EALNVGSELPFKIVEGKVTSDICNLPDQASPSEFNHAMNQCVNDQRKAALDNLLSRSLLA LLGLAVIAFAFGYAMAGRVLSPLGRITRTARAVAGSDLSRRIELDGPDDELKELADTFDD MLERLQRAFTAQQRFVGNASHELRTPLAINRTLLEVHLSDPGAPVELQQLGKTLLATNER SEQLVEGLLLLARSDNQVVERGPVDLAEVASQAIDQVHGEAEGKGVRIRGEQKPAVVQGN GVLLERIALNLVQNAVRYNVAEGGWVEVTTEVQHGQAVLVVSNTGPVVPAYEIDNLFEPF RRLRTERTGSDKGVGLGLSIVRSVARAHGGHIYAQPREGGGLVMRVTLPI  
>QU709_RS10070 Streptomyces_coralus_strain_SX92.gbff Streptomyces coralus recipro:1 MAATPAPPQAPPKPTWDPRRPEPPFPWLRPTIRIRLTLLYGGMFLIAGILLLSIIYLLAA QAISTGNQPPFKIISASTDVKIASPDCPAVNNASGLTLDQFNSAISACIDHQRTIALDNL LSRSLLALLGLAIIAFAFGYAMAGRVLSPLGRITRTARAVAGSDLSRRIELDGPDDELKE LADTFDEMLERLQRAFTAQQRFVGNASHELRTPLAINRTLLEVHLSDPNAPVELQQLGKT LLATNERSEQLVEGLLLLARSDNQVVERKPVDLAEVAEQAVDQVHGEAAAKGVVIRGEQK PAVVQGNGVLLERIALNLVQNAVRYNVPEGGWVEVTTDVQHGQAVLVVSNTGPVVPAYEI DNLFEPFRRLRTERTGSDKGVGLGLSIVRSVARAHGGHISAQPREGGGLVMRVALPI  
>STRCI_RS30195 Streptomyces_cinnabarinus_strain_DSM_40467.gbff Streptomyces cinnabarinus recipro:1 MASTPAPPQAPPKPTWDPRRPVQPFPWLRPTIRIRLTLLYGGMFLIAGILLLSIIYLLAA SALNVGSDLPFKILSGQVSSDMCNLGATRLPADELNHALNECVNEQREHALDTLLSRSLL ALLGLAVIAFAFGYAMAGRVLSPLGRITRTARAVAGSDLSRRIELDGPDDELKELADTFD DMLERLQRAFTAQQRFVGNASHELRTPLAINRTLLEVHLSDPGAPMELQQLGKTLLATNE RSEQLVEGLLLLARSDNQIVERKPVDLAEVAEQAVDQVHGEAEAKGVEIRGKRDPAVVQG NGVLLERIALNLVQNAVRYNIPEGGWVEVTTELQHGQALLVVSNTGPVVPAYEIDNLFEP FRRLRTERTGSDKGVGLGLSIARSVARAHGGHIAAQPREGGGLEMRVALPV  
>CP983_RS11290 Streptomyces_chartreusis_strain_ATCC_14922.gbff Streptomyces chartreusis recipro:1 MAATPAPPQAPPKPTWDPRRSEPPFPWLRPTIRIRLTLLYGGMFLIAGILLLSIIYLLAA QAINTGNEPLFKIVGGAELKVTSDNCPAINNASNLTYPQFNDAVSACIDDQRRVALDDLL SRSLLALLGLAIIAFAFGYAMAGRVLSPLGRITRTARAVAGSDLSRRIELDGPDDELKEL ADTFDDMLERLQRAFTAQQRFVGNASHELRTPLAINRTLLEVHLSDPNAPVELQQLGKTL LATNERSEQLVEGLLLLARSDNQIVERKPVDLAEVAEQAVDQVHAEAEAKGVVIRGEQKT AVVQGNGVLLERIALNLVQNAVRYNVPEDGWVEVTTEIQHGQAVLVVSNTGPVVPAYEID NLFEPFRRLRTERTGSDKGVGLGLSIVRSVVRAHGGHISAQPREGGGLVMRVTLSV  
>S1361_RS29090 Streptomyces_cyanogenus_strain_S136.gbff Streptomyces cyanogenus recipro:1 VATTPAPPQAPPKPTWDPRRPQPPFPWLRPTIRIRLTLLYGGMFLIAGILLLSIIYLLAA QAISTGNQPLFKIVSGSAIKVTSDNCPAVTAINSGPVPLSDFNDAIAHCVDQQRQGALDN LLSRSLLALLGLAVIAFAFGYAMAGRVLSPLGRITRTARQVAGSDLSRRIELDGPDDELK ELADTFDDMLERLQRAFTAQQRFVGNASHELRTPLAINRTLLEVHLSDPNAPVELQQLGK TLLATNERSEQLVEGLLLLARSDNQIVERKPVDLAEVATQAIDQVHAEAEAKGVSIRGER KPAVVQGNGVLLERIALNLVQNAVRYNVAEKGWVEVTTEIRHGQAVLTVTNTGPVVPAYE IDNLFEPFRRLRTERTGSDKGVGLGLSIVRSVARAHGGHILARPREGGGLVMRVTFPV  
>I3J06_RS05000 Streptomyces_clavuligerus_strain_F1D7.gbff Streptomyces clavuligerus recipro:1 MAATPAPLPPAAPPKPTWDPRSPVRPLLRPTIRIRLTLLYGGMFLIAGVLLLSIIYLLAA QALTSGSSLPFKLLPESRIQRTNDSCPALMPGLSAEQANSVLSACFAHEREVALDDLLQR SLFALLGLSVIAFAFGYAMAGRVLSPLGQITRTARRVAGTDLSRRIELDGPDDELKELAD TFDEMLDRLDRAFTAQQRFVANASHELRTPLAINRTLLEVHLSDPGAPVELQQLGRTLLA TNERSEQLVEGLLLLARSDNQTFERKPVDLAEVASRAIDQSRAEAEAKGVEIRGERAPAV VQGNGVLLERIALNLVQNAVRYNVPEDGWVEVATEAGHGQAVLVVSNTGPVVPAYEIDNI WEPFRRLRQDRTGSDKGVGLGLSIARSVARAHGGRIIAEPREGGGLVMRVTLPL  
>N8I84_RS29800 Streptomyces_cynarae_strain_HUAS_13-4.gbff Streptomyces cynarae recipro:1 VATTPAPPTAPPKPTWDPRRPELPFPWLRPTIRIRLTLLYGGMFLIAGILLLSIIYLFAA HALNVGSELPFKILQGQVASDTCNLPQQTSASVLNDALDACVNEQRQHALDNLLSQSLLA LLGLAVIAFAFGYAMAGRVLSPLGRITRTARAVAGSDLSRRIELDGPDDELKELADTFDD MLERLQRAFMAQQRFVGNASHELRTPLAINRTLLEVHLSDPGAPTELQQLGKTLLATNER SEQLVEGLLLLARSDNQIVERKPVDIAEVASQAVDQVHAEAEAKGVEIRGERAPVVVQGN GVLLERIALNLVQNAVRYNIPEGGWVEVTTQAQHGQAVLVVSNTGPVVPAYEIDNLFEPF TRLRTERTGSDKGVGLGLSIARSVARAHGGHISAQPREGGGLVMRVTLPL  
>EJ357_RS34470 Streptomyces_cyaneochromogenes_strain_MK-45.gbff Streptomyces cyaneochromogenes recipro:1 MAATPAPPQAPPKPTWAPRRVEPPFPWLRPTIRIRLTLLYGGMFLIAGILLLSIIYLLAA QAISTGNQPPFKIVGATDEIKVASADCPAVNNATGLTLTQFNSAISACIDHQRQVALDDL LSRSLLALLGLAVIAFAFGYAMAGRVLSPLGRITRTARAVAGSDLSRRIELDGPDDELKE LADTFDEMLERLQRAFTAQQRFVGNASHELRTPLAINRTLLEVHLSDPNAPVELQQLGKT LLATNERSEQLVEGLLLLARSDNQIVERKPVDLAEVAEQAVDQVHAEAESKGVVIRGEQK PAVVQGNGVLLERIALNLVQNAVRYNVPEGGWVEVTTEVQHGHAVLVVTNTGPVVPAYEI DNLFEPFRRLRTERTGSDKGVGLGLSIVRSVARAHGGHIAAQPREGGGLVMRVTLPI  
>K7C20_RS27670 Streptomyces_decoyicus_strain_NRRL_2666.gbff Streptomyces decoyicus recipro:1 MPSLPSFSKAAAPPPPVPPKPTWDPRPVDVRPFPWLRPTIRIRLTLLYGGMFLMAGIVLL TIIYMLAADALHDGSALPLKILGGKFQSTSDICDLPTETSGQLLQQAVEQCLQHQRAVAL NSLLNRSLLALLGLTVVAFAFGYAMAGRVLSPLGRITRTAQRVAGSDLHRRIELGGPDDE LKELADTFDEMLDRLDRAFESQRRFVSNASHELRTPLAINRTLLEVQLADPEASPELQQL GKTLLATNERSEQLVEGLLLLARSENKVVDKRPVDLSEVAAQAVDQSRAEAHGKGVEFRG VRGQVFVQGNGVLLERIALNLVQNAVRYNVPEDGWVEVSTEPQPGCAVLVVANTGPVVPA YEVENLFEPFRRLRTERTGSDKGVGLGLSIVRSVVRAHDGTITAQPREGGGLVMRVVLPL  
>R2B67_RS07110 Streptomyces_cyaneofuscatus_strain_89-2-2.gbff Streptomyces cyaneofuscatus recipro:1 MAATPPPPTAPPKPTWEPKQQEPPYPWLRPTIRIRLTLLYGGMFLIAGILLLSIIYMLAA QALHDAGSLEFKVGGNNVLIASDTCPQLGAATNNDQLNETIKACTAAQRQHALDSLLNRA LLALVGLSVIAFAFGYAMAGRVLSPLGRITRTARRVAGTDLTRRIELDGPDDELKELADT FDDMLDRLERAFTAQQRFVGNASHELRTPLAINRTLLEVHLSDPEAPPELQQLGKTLLAT NERSEQLVEGLLLLARSDNQIVERKPVDLAEVADRAIDQARAEAVARKVEIRGERSAAVV QGNGVLLERIALNLVQNAVRYNVAEDGWVEVDTSLQPGQALLVVSNTGPVVPAYEIDNLF EPFRRLRTERTGSDKGVGLGLSIARSVARAHGGRIIAEPREGGGLVMRVTLPV  
>D9753_RS08765 Streptomyces_dangxiongensis_strain_Z022.gbff Streptomyces dangxiongensis recipro:1 VAATPAPPQAPPKPTWDPSRAQPPFPWLRPTIRIRLTLLYGGMFLIAGILLLSIIYLLAA QAISSGNQPLFKIVSFQKLQVSSDNCPAINTTSLPLEDFKDAISRCVDHQRQGALDNLLS RSLLALLGLAVIAFAFGYAMAGRVLSPLGRITRTARQVAGSDLSRRIELDGPDDELKELA DTFDDMLERLQRAFTAQQRFVGNASHELRTPLAINRTLLEVHLSDPDAPVELQQLGRTLL ATNERSEQLVEGLLLLARSDNQIVERKPVDLAEVATQAVDQVHAEAEAKGVEIRGERKPA VVRGNGVLLERIALNLVQNAVRYNVAEHGWVEVHTEVEHGQAVLTVSNTGPVVPAYEIDN LFEPFRRLRTERTGSDKGVGLGLSIVRSVTRAHGGHILARPREGGGLVMRVTLPV  
>C4B68_RS09950 Streptomyces_dengpaensis_strain_XZHG99.gbff Streptomyces dengpaensis recipro:1 VATTPAPPTAPPKPTWDPRRAEPPFPWLRPTIRIRLTLLYGGMFLIAGIMLLSIIYLLAA QALNVGTELPFKITEGRVTSTICNLPDSASPDVFNNAMNACVNEQRQHALDNLLSRSLLT LLGLAIIAFAFGYAMAGRVLAPLGRMTRTARAVAGSDLSRRIELDGPDDELKELADTFDD MLERLQRAFTAQQRFVGNASHELRTPLAINRTLLEVHLSDPHAPVELQQLGKTLLATNER SEQLVEGLLLLARSDNQIVERKPVDLAEVASQAVDQVHAEAEAKGVEIRGQRAPAVVQGN GVLLERIALNLVQNAVRYNVPQGGWVEVTTEVQHGQAVLVVSNTGPVVPAYEIDNLFEPF RRLRTERTGSDKGVGLGLSIARSVARAHGGHISAEPREGGGLVMRVTLPV  
>OHA91_RS10500 Streptomyces_erythrochromogenes_strain_NBC_00303.gbff Streptomyces erythrochromogenes recipro:1 VATTPAPPTAPPKPTWDPGQPEGPFPWLRPTIRIRLTLLYGGMFLIAGILLLSIIYLLAA QALRQGNALPFQIVGGQVKVTSSSCPGVVGLELTPDQFNAAIGQCILEQRRHALDDLLSR SLMALLGLSIIAFAFGYAMAGRVLSPLGKITRTARRVVGSDLTRRIELDGPDDELKELAD TFDEMLDRLERAFTAQQRFVANASHELRTPLAINRTLLEVHLSDPGAPVELQQLGKTLLA TNERSEQLVEGLLLLARSENQIVERKPVDLAEVASRAVDQVRGEADAKGVEIRGEREPAV VQGNGVLLERIALNLVQNAVRYNVPEDGWVEVTTEARHGQAVLLVSNTGPVVPAYEVDNL FEPFRRLRTERTGSDKGVGLGLSIARSVARAHGGRIQAMPREGGGLVMRVTLPL  
>L3078_RS33695 Streptomyces_deccanensis_strain_KCTC_19241.gbff Streptomyces deccanensis recipro:1 MATTPAPPLAPPKPTWDPRRPQNPLPWLRPTIRIRLTLLYGGMFLIAGILLLSIIYLLAA QAVNTGNAPLFKIVGGTDISVTSEKCPAVDAGSTNVRLADFNAAIAACIDDQRKVALDTL LSRSLLALLGLAVIAFAFGYAMAGRVLAPLGRITRTARAVAGSDLSRRIELDGPDDELKE LADTFDEMLERLQRAFTAQQRFVGNASHELRTPLAINRTLLEVHLSDPNAPTELQQLGKT LLATNERSEQLVEGLLLLARSDNQIVERKPVDLAEVASRAIDQVRSEADAKKVEIRGERA PAVVQGNGVLLERIALNLVQNAVRYNVQGAGGWVEVTTELQHGQALLVVTNTGPVVPAYE IDNLFEPFRRLRTERTGSDKGVGLGLSIVRSVARAHGGHIAARPREGGGLVMRVTLPV  
>M4V62_RS12130 Streptomyces_durmitorensis_strain_MS405.gbff Streptomyces durmitorensis recipro:1 VAATPAPPAAPPKPTWDPRKAEAPFPWLRPTIRIRLTLLYGGMFLIAGILLLSIIYLLAA QALNVGSDLPFKIVSGQVASDTCNFPSSPSAGELNNAMNACVNEQRQHALDNLLSRSLLA LLGLAVIAFAFGYAMAGRVLSPLGRITRTARRVAGTDLSRRIELDGPDDELKELSDTFDE MLDRLERAFTAQQRFVGNASHELRTPLAINRTLLEVHLSDPGAPPELHQLGKTLLATNER SEQLVEGLLLLARSDNQIVERKPVDLAEVADRAVDQVLTEADAKGVEIRGERGPAVVQGN GVLLERIALNLVQNAVRYNVPDEGWVEVTTEAQHGQAVLVVANTGPVVPAYEIDNLFEPF RRLRTERTGSDKGVGLGLSIARSVARAHGGRIIAEPREGGGLVMRVTLPL  
>OG858_RS33935 Streptomyces_europaeiscabiei_strain_NBC_00515.gbff Streptomyces europaeiscabiei recipro:1 MAPIPAPPQAPPKPTWDPGRAQTPFPWLRPTIRIRLTLLYGGMFLIAGILLLSIIYLLAA QAVSTGNTPVFKIEDGKDISVSSNICPAVDANTAPTNLRLDDFNAAISACIDHERQVALD TLLSRSLLALLGLAVIAFAFGYAMAGRVLAPLGRITRTARAVAGSDLSRRIELDGPDDEL KELADTFDDMLERLQRAFTAQQRFVGNASHELRTPLAINRTLLEVHLSDPGAPAELQQLG KTLLATNERSEQLVEGLLLLARSDNQIVERKPVDLAEVASQAIDQVRSEAESKKVEIRGE WAPAVVQGNGVLLERIALNLVQNAVRYNVPGPGGWVEVTTELQHGQAVLVVTNTGPVVPA YEIDNLFEPFRRLRTERTGSDKGVGLGLSIVRSVARAHGGHIAARPREGGGLVMRVTLPV  
>EIZ62_RS07215 Streptomyces_ficellus_strain_NRRL_8067.gbff Streptomyces ficellus recipro:1 VATTPAPPQAPPKPTWDPRDPVRPWLRPTIRIRLTLLYGGMFLIAGILLLSIIYLLAAQA LHIGSELPFKIVNGQVTSNVCNFPDEAGPDEFNRAMNSCANEQRQHALDDLLRRSLFALL GLSVIAFAFGYAMAGRVLSPLGRITRTARNVAGTDLSRRIELDGPDDELKELADTFDDML DRLDRAFTAQQRFVANASHELRTPLAINRTLLEVHLSDPGAPVELHQLGKTLLATNERSE QLVEGLLLLARSDNEVVERKPVDLAEVASRAIDQTRSEAEAKGVEIRGERAPATVQGNGV LLERIALNLVQNAVRYNVDDGWVEVTTGTQPGQAVLVVSNTGPVVPAYEIDNLFEPFRRL RQERTGSDKGVGLGLSIARSVARAHGGRIIAEPREGGGLVMRVTLPV  
>OJ254_RS00100 Streptomyces_endophytica_strain_HNM0140.gbff Streptomyces endophytica recipro:1 MPSLPSFSKPAAPPQPIPPKPAWDPRPPVNVRPFPWLRPTIRIRLTLLYGGMFLMAGIVL LTIIYMLAADALHSGSALPLKILGGKFESTSDICDLPTQTSGPLLQQAVEQCLQQQRALA LNSLLNRSLLALLGLTVVAFAFGYAMAGRVLSPLGRITRTAQRVAGSDLHRRIELGGPDD ELKELADTFDEMLDRLDRAFESQRRFVANASHELRTPLAINRTLLEVQLADPDASPELAQ LGKTLLATNERSEQLVEGLLLLARSENKVVDKKPVDLSEVAAQAVDQIREEAQAKGVVLR GVRQQVFVQGNGVLLERIALNLVQNAVRYNVPEGWVEVSTEPQPGCAVLVVTNTGPVVPA YEVENLFEPFRRLRTERTGSDKGVGLGLSIVRSVVRAHDGTITAEPREGGGLVMRVVLPL  
>GFH48_RS11345 Streptomyces_fagopyri_strain_QMT-28.gbff Streptomyces fagopyri recipro:1 VATTPAPSTAPPKPTWDPRRTEPPFPWLRPTIRIRLTLLYGGMFLIAGIMLLSIIYLLAA QALNVGSELPFKIISGQVGSSTCNFPSAPSASELNTAMNACANQQRQHALDDLLSRSLLA LLGLAIIAFAFGYAMAGRVLSPLGRITRTARAVAGSDLSRRIELDGPDDELKELADTFDD MLERLQRAFTAQQRFVGNASHELRTPLAINRTLLEVHLSDPGAPVELQQLGKTLLATNER SEQLVEGLLLLARSDNQIVERKPVDLAEVASQAVDQVNGEAEAKGVRIRGERAAAVVQGN GVLLERIALNLVQNAVRYNVPEGGWVEVTTVVQHGHAVLLVSNTGPVVPAYEIDNLFEPF RRLRTERTGSDKGVGLGLSIARSVARAHGGHIAAEPREGGGLVMRVTLPI  
>BFF78_RS11865 Streptomyces_fodineus_strain_TW1S1.gbff Streptomyces fodineus recipro:1 MAAAPAPPQAPPKPTWDPRRPQPPFPWLRPTIRIRLTLLYGGMFLIAGILLLSIIYLLAA HALNVGSELPFKILSGSVSSSTCNFPSQPSASELNTAMNDCVNQQRQHALDDLLSRSLLA LLGLAVIAFAFGYAMAGRVLFPLGRITRTARQVAGSDLSRRIELDGPDDELKELADTFDD MLERLQRAFTAQQRFVGNASHELRTPLAINRTLLEVHLSDPNAPVELQQLGKTLLATNER SEQLVEGLLLLARSDNQIVERGPVDLAEVATQAIDQVHAEAEAKGVKIRGERKPAVVQGN GVLLERIALNLVQNAVRYNVAEEGWVEVNTEVQHAQAVLTVTNTGPVVPAYEIDNLFEPF RRLRTERTGSDKGVGLGLSIVRSVARAHGGHIAAQPREGGGLVMRVTLPV  
>CNQ36_RS29360 Streptomyces_fungicidicus_strain_TXX3120.gbff Streptomyces fungicidicus recipro:1 MAATPAPPTAPPKPTWDPRKADPPFPWLRPTIRIRLTLLYGGMFLIAGILLLSIIYLLAA QALHDGGGGQSFQVTGTNIDISSQTCPQLDSATTNGQLNDMLKQCNAVQRQHALDDLLSR SLGALLGLAIIAFAFGYAMAGRVLSPLGRITRTARAVAGSDLSRRIELDGPDDELKELAD TFDDMLERLQRAFTAQQRFVGNASHELRTPLAINRTLLEVHLSDPGAPVELQQLGKTLLA TNERSEQLVEGLLLLARSDNQIVERKPVDLAEVATQAIDQVHGEAEAKGVVIRGERRQAV VQGNGVLLERIALNLVQNAVRYNVPEDGWVEVTTDVQHGQAVLVVSNTGPVVPAYEIDNL FEPFRRLRTERTGSDKGVGLGLSIVRSVARAHGGHIYAQPREGGGLVMRVTLPI  
>IM697_RS33830 Streptomyces_ferrugineus_strain_CCTCC_AA2014009.gbff Streptomyces ferrugineus recipro:1 MAATPAPPQAPPKPTWDPRRAEPPFPWLRPTIRIRLTLLYGGMFLIAGILLLSIIYLLAA QAINTGNEPLFKIVSFRELQVSSDNCPGIKPGLTISEFNEAISACIDSQRRVALDDLLSR SLLALLGLAIIAFAFGYAMAGRVLSPLGRITRTARAVAGSDLSRRIELDGPDDELKELAD TFDEMLERLQRAFTAQQRFVGNASHELRTPLAINRTLLEVHLSDPTAPVELQQLGKTLLA TNERSEQLVEGLLLLARSDNQIVERKPVDLAEVAEQAVDQVHAEAEAKGVVIRGEQKPAV VQGNGVLLERIALNLVQNAVRYNVPEDGWVEVITDVQHGQAVLVVSNTGPVVPAYEIDNL FEPFRRLRTERTGSDKGVGLGLSIVRSVVRAHGGHISAEPREGGGLVMRVTFPV  
>J4032_RS09570 Streptomyces_formicae_strain_1H-GS9.gbff Streptomyces formicae recipro:1 VAAAPAPPQAPPKPTWAPGEPVRPWFRPTIRIRLTLLYGGMFLIAGILLLSIIYLLAAQA VQVGSQLPFKIVDGSVTSEVCNFPDKAPPAEFNEAMNACVNEQRQHALDELLTRSLFALV GLSVIAFAFGYAMAGRVLSPLGQITRTARRVVGSDLSRRIELDGPDDELKELADTFDEML ERLERAFTAQQRFVANASHELRTPLAINRTLLEVHLSDPGAPDELRQLGKTLLATNERSE QLVEGLLLLARSDNQIVERKPVDLAEVATRAVDQARGEAEAKGVEFRGRRSPVVVQGNGV LLERIALNLVQNAVRYNVREGGWVEVTTELSHGQAVLVVSNTGPVVPAYEIDNLFEPFRR LRQERTGSDKGVGLGLSIARSVARAHGGRIIAEPREGGGLVMRVFLPV  
>CP966_RS27480 Streptomyces_galilaeus_strain_ATCC_14969.gbff Streptomyces galilaeus recipro:1 MATTPAPPQAPPKPTWDPRRPQAPFPWLRPTIRIRLTLLYGGMFLIAGILLLSIIYLLAA NALNVGSELPFKILTGQVASDICNFPNEPTASELNGAMNECVNQQRQHALDDLLSRSLLA LLGLAVIAFAFGYAMAGRVLAPLGRILRTARSVAGSDLSRRIELDGPDDELKELADTFDD MLERLQRAFTAQQRFVGNASHELRTPLAINRTLLEVHLSDPNAPVELQQLGKTLLATNER SEQLVEGLLLLARSDNQIVERKPVDLAEVAGQAIDQVHGEAEAKGVKIRGKRDSAVVQGN GVLLERIALNLVQNAVRYNVPEDGWVEVTTEVLHGQAVLVVSNTGPVVPAYEIDNLFEPF RRLRTERTGSDKGVGLGLSIARSVARAHGGHIAAQPREGGGLVMRVSLPL  
>B1H19_RS29985 Streptomyces_gilvosporeus_strain_F607.gbff Streptomyces gilvosporeus recipro:1 MPSLPSFSKAHTPPPAAPPKPTWDPRPQVNIRPFPWLRPTIRIRLTLLYGGMFLMAGIVL LTIIYMLAANTLSVGNQLPFKIVGASELHVTSDTCPELTPAVRNVAQLNSAVAECLQHQR TFALNTLLNRSLLALLGLTVVAFAFGYAMAGRVLSPLGRITRTAQRVAGSDLHRRIELGG PDDELKELADTFDEMLDRLDRAFDAQRRFVSNASHELRTPLAINRTLLEVQLADPDASPE LTQLGKTLLATNERSEQLVEGLLLLARSENKVVAKKPVDLAEVASQAVDQTREEAHAKGV ELRGVRRQVFVQGNGVLLERIALNLVQNAVRYNVPQDGWVEVTTEPQPGCAVLVVSNTGP VVPAYEIENLFEPFRRLRTERTGSDKGVGLGLSIVRSVVRAHDGSITAEPREGGGLVMRV VLPL  
>CP974_RS23385 Streptomyces_fradiae_ATCC_10745.gbff Streptomyces fradiae ATCC 10745 = DSM 40063 recipro:1 VADTPTPRAAPPKPTWDPRAPARPLLRPTIRIRLTLLYGGMFLIAGVLLLSIIYLLTRQA LYISTDDLPFKLLKGEVDPNFDWCRLPAEGVTAEQFNDAMAGCLQHQRDLALDDLLRRSL FALLGLSIIAFAFGYAMAGRVLSPLGRITRTARQVAGSDLSRRIKLVGPDDELKELADTF DEMLDRLERAFTAQQRFVANASHELRTPLAINRTLLEVHLSDPAAPVEMRELGKALLATN ERSEQLVEGLLLLARSENQIVERKPVDIAEVASRAIDQVRGEAEERGVEFRGERAPAVVQ GNGVLLERIALNLVQNAVRYNVPEDGWVEVTTAVEGGQAVLVVSNTGPVVPAYEIDNLFE PFRRLREERTGSDRGVGLGLSIVRSVARAHGGRITAVPREGGGLVMRVTFPR  
>H4W23_RS29855 Streptomyces_gardneri_strain_ATCC_15439.gbff Streptomyces gardneri recipro:1 MPTTSAPHPQAPPKPTWDPRDPVRPLLRPTIRIRLTLLYGGMFLIAGILLLSIIYLFTAQ TLGESAAKLPFEIVTGKVQPTTSWCELPVQGSGKQFNDAVSVCLRHQGDLALEDLLRRSL FALLGLSIIAFAFGYAMAGRVLSPLGRITRTARQVAGSDLARRIELDGPDDELKELADTF DEMLERLERAFTAQQRFVANASHELRTPLAINRTLLEVHLSDPGAPVELQQLGKTLLATN ERSEQLVEGLLLLARSDNQIIERKPVDLAEVASRAVDQTHAEAAAKGVEIRGERAPAVVQ GNGVLLERIALNLLQNAVRYNVPEGGWVEVGTEIEHGQAVLVVSNTGPVVPAYEIDNLFE PFRRLRQERTGSDKGVGLGLSIARSVARAHGGRIIAEPREGGGLVMRVTLPI  
>C0216_RS07905 Streptomyces_globosus_strain_LZH-48.gbff Streptomyces globosus recipro:1 VAATPAPPTAPPRPTWDPGQPEGPFPWLRPTIRIRLTLLYGGMFLIAGILLLSIIYLLAA QALREGNALPFKIVSISGQKVVVSSPTCPGVGPEQTLDQFHAAIQACMLEQRRHALDDLL SRSLMALLGLSVIAFAFGYAMAGRVLSPLGKITRTARRVVGSDLTRRIELDGPDDELKEL ADTFDEMLDRLERAFTAQQRFVANASHELRTPLAINRTLLEVHLSDPGAPVELQQLGKTL LATNERSEQLVEGLLLLARSENQIVERKPVDLAEVASRAIDQARAEAAAKGVEIRGERAS AVVQGNGVLLERIALNLVQNAVRYNVPEGGWVEVTTEAVPGHAVLVVSNTGPVVPAYEVD NLFEPFRRLRTERTGSDKGVGLGLSIARSVARAHGGRITAVPRDGGGLVMRVTLPL  
>IAG43_RS24185 Streptomyces_genisteinicus_strain_CRPJ-33.gbff Streptomyces genisteinicus recipro:1 VAAVPAPPEAPPKPTWDPRDPVQPWLRPTIRIRLTLLYGGMFLIAGILLLSIIYLFTAQA LHNISELPFRLLPDSKIELTDNSCEALRAGLSADQANAALKSCFAHQRELALDDLLRRSL FALLGLSIIAFAFGYAMAGRVLSPLGRITRTARQVAGSDLTRRIELDGPDDELKELADTF DEMLDRLERAFTAQQRFVANASHELRTPLAINRTLLEVHLSDPGAPVELQQLGKTLLATN ERSEQLVEGLLLLARSDNQIVERKPVDLAEVASRAIDQVRSEAEALGVEVRGERGPAVVQ GNGVLLERIALNLVQNAVRYNVAENGWVEVSTEIRQGEAVLTVSNTGPVVPAYEVDNIFE PFRRLRQERTGSDKGVGLGLSIARSVARAHGGRIIAEPREGGGLVMRVTLPT  
>SGFS_RS17585 Streptomyces_graminofaciens_strain_A-8890.gbff Streptomyces graminofaciens recipro:1 VTTTPAPPLAPPKPTWDPRRPQTSLPWLRPTIRIRLTLLYGGMFLIAGILLLSIIYLLAA QALHEGGGPSFQVTGANLDITSSTCPAVNTVPDSEINSVLKQCDAIERQQALDTLLSRSL LALLGLAVIAFAFGYAMAGRVLAPLGRITRTARAVAGSDLSRRIELDGPDDELKELADTF DDMLERLQRAFTAQQRFVGNASHELRTPLAINRTLLEVHLSDPHAPTELQQLGRTLLATN ERSEQLVEGLLLLARSDNQIVERKPVDLAEVASQAIDQVRTEADAKGVEIRGEHAPAVVQ GNGVLLERIALNLVQNAVRYNVAEGGWVEVTTELQHGRAVLVVTNTGPVVPAYEIDNLFE PFRRLRTERTGSDKGVGLGLSIVRSVARAHGGHIAAQPREGGGLVMRVTLPV  
>test1122_RS21635 Streptomyces_gobiensis_strain_1_25.gbff Streptomyces gobiensis recipro:1 MTAPTSPPPSGTGTGTGPPPPPSAPPKPTWGPPEPPRSYLWLRPTIRIRLTVLYGGMFLV AGMLLLTIIYLLAAQALQEGSKLPIRITGLDLQVSSETCQLPTIGTSDDLNRALDACLQH QRRVALESLLKHSLLALLGLAVAAFAIGYVMSGRVLSPLGRITRTARQVAGSDLHRRIEL DGPDDELKELSDTFDEMLDRLDRAFTAQQRFVANASHELRTPLAINRTLLEVQLSDPAAS PDVQQLGKTLLATNERSEQLVEGLLLLARSENEIIDRKPVDLAEVAGRAVEQSRTEAEEK GVELRGVRQPAYVQGNGVLLERVALNLVQNAVRYNLREGGWVSVSTVARPGEAVLTVENT GPQVPAYEIDNLFEPFRRLRTERTGSDKGVGLGLSIARSVARAHGGTITAEPREEGGLVM RVALPV  
>AVL59_RS10495 Streptomyces_griseochromogenes_strain_ATCC_14511.gbff Streptomyces griseochromogenes recipro:1 MAATPAPPQAPPKPTWDPRRPQPPFPWLRPTIRIRLTLLYGGMFLIAGILLLSIIYLLAA QAITTGNATLFKIVSFQNLQVASDNCPAITNHLPLADFNEAISRCADQQRQTALDNLLSR SLLALLGLAVIAFAFGYAMAGRVLSPLGRITRTARQVAGSDLSRRIELDGPDDELKELAD TFDDMLERLERAFTAQQRFVGNASHELRTPLAINRTLLEVHLSDPNAPVELQQLGKTLLA TNERSEQLVEGLLLLARSDNQIVERKPVDLAEVATQAIDQVHGEAEAKGVKIRGERKPAV VQGNGVLLERIALNLVQNAVRYNVPEDGWVEVGTEVEHQQAVLTVTNTGPVVPAYEIDNL FEPFRRLRTERTGSDKGVGLGLSIVRSVARAHGGHIAARPREGGGLVMRVTLPL  
>PET44_RS24565 Streptomyces_goshikiensis_strain_CGMCC_4.1796.gbff Streptomyces goshikiensis recipro:1 MAATPAPPAAPPKPTWDPGQPEGPFPWLRPTIRIRLTLLYGGMFLIAGILLLSIIYLLAA QALREGNALPFKIVSVSGQKVEVSSPTCSGVGTDQSLDQFNAAIQACILDQRKHALDDLL SRSLMALLGLSVIAFAFGYAMAGRVLSPLGKITRTARRVVGSDLTRRIELDGPDDELKEL ADTFDEMLDRLERAFTAQQRFVANASHELRTPLAINRTLLEVHLSDPGAPVELQQLGKTL LATNERSEQLVEGLLLLARSDNQIVERKPVDLAEVASRALDQARGEAETKGVEIRGECAP AVVQGNGVLLERIALNLVQNAVRYNVPEGGWVEVTTEVQHGHAVLLVSNTGPVVPAYEVD NLFEPFRRLRTERTGSDKGVGLGLSIARSVARAHGGRIQATPREGGGLVMRVTLPV  
>SGR_RS08160 Streptomyces_griseus_subsp._griseus_NBRC_13350.gbff Streptomyces griseus subsp. recipro:1 MATTQGPPAAPPKPTWEPKQQEPPYPWLRPTIRIRLTLLYGGMFLIAGIVLLSIIYMLAA QALSVGTDLPFEIVSGKVTSEICDLPANASPNDFNAAMNACVNHQRKAALETLLNRSLLA LVGLSVIAFAFGYAMAGRVLSPLGRITRTARRVAGTDLTRRIELDGPDDELKELADTFDD MLDRLERAFTAQQRFVGNASHELRTPLAINRTLLEVHLSDPEAPPELQQLGKTLLATNER SEQLVEGLLLLARSDNQIVERKPVDLAEVAERAIDQARSEAVAKKVEIRGERSTAVVQGN GVLLERIALNLVQNAVRYNVPEDGWVEVTTEARDGQALLVVSNTGPVVPAYEIDNLFEPF RRLRTERTGSDKGVGLGLSIARSVARAHGGRIIAEPREGGGLVMRVTLPV  
>HEP81_RS10830 Streptomyces_griseofuscus_strain_DSM_40191.gbff Streptomyces griseofuscus recipro:1 MASTPAPPQAPPKPTWDPRRPEPPFPWLRPTIRIRLTLLYGGMFLIAGILLLSIIYLLAA QAISTGNQPLFKIVSFQELKVSSDNCPAINTTSLPLADFNNTISQCVDHQRQAALDNLLS RSLLALLGLAVIAFAFGYAMAGRVLSPLGRITRTARAVAGSDLSRRIELDGPDDELKELA DTFDEMLERLQRAFTAQQRFVGNASHELRTPLAINRTLLEVHLSDPGAPVELQQLGKTLL ATNERSEQLVEGLLLLARSDNQIVERKPVDLAEVAGQAIDQVRSEAEAKGVEIRGERAPA VVQGNGVLLERIALNLVQNAVRYNVAEGGWVEVDTEVQHGQAVLTVSNTGPVVPAYEIDN LFEPFRRLRTERTGSDKGVGLGLSIVRSVARAHGGHIAARPREGGGLVMRVTLPL  
>K9S39_RS12985 Streptomyces_halobius_strain_3_2.gbff Streptomyces halobius recipro:1 MPSLPSFSKAASTPPPAAPPKPTWDPKPVNVRPFPWLRPTIRIRLTLLYGGMFLMAGIVL LTIIYMLAANALSAGNQLPFKIVGAKQLEIYSSTCPGLTAVGDIPQLNSEVSQCLLHQRA AALDTLLQRSLMALMGLTVVAFAFGYAMAGRVLSPLGRITRTAQRVAGSDLHRRIELGGP DDELKELADTFDEMLDRLDRAFESQRRFVSNASHELRTPLAINRTLLEVQLADPDASPEL AQLGKTLLATNERSEQLVEGLLLLARSENKVVDKKPVDLSEVAAQAVEQTREEAHTKGVA LRGVRQQVFVQGNGVLLERIALNLVQNAVRYNVPEEGWVEVTTEPQPGCAVLVVTNTGPV VPAYEIENLFEPFRRLRTERTGSDKGVGLGLSIVKSVVRAHDGAITAEPREGGGLVVRVV LPL  
>HUT13_RS22940 Streptomyces_harbinensis_strain_NA02264.gbff Streptomyces harbinensis recipro:1 MTAPSSPSSPPPPPPPVQPPYPGRSGLRRPAAPPRPDFDPPEADPPRSWLRPTIRIRLTL LYGGMFLFAGMLLLTIIYLLAAEALHKGNALPFLITSDTRVQITSDSCQGISGNVTSDQF KDWLAGCVDIQRDVALKALLRRSLMALLGLAVAAFAFGYVMAGRVLSPLGRITRTARQVA GSDLHRRIELDGPDDELKELADTFDEMLDRLDRAFTAQQRFVANASHELRTPLAINRTLL EVQLSDPHASPEVVQLGNTLLATNRRSEQLVEGLLLLARSDNQLVERKPVDLAEVAGQAL EQTRGEAQERGVELAGVRPPVYVQGNGVLLERVALNLVQNAVRHNVAEGGWVRVDTQARP GQAVLIVENTGPMVPAYEVDNMFEPFRRLGKDRTGSDKGVGLGLSIVRSVARAHGGHVVA VPRETGGLVMRVTLPV  
>OIE73_RS29030 Streptomyces_hirsutus_strain_NBC_01753.gbff Streptomyces hirsutus recipro:1 VATTPTPTPPPPPAPPKPTWDPRRPVPPFPWLRPTIRIRLTLLYGGMFLIAGILLLSIIY LLAANALNVGSDLPFEIISGQVRSDICRLRSAQLPADELNAALNACVNKQRQHALDNLLS RSLLALLGLAVIAFAFGYAMAGRVLSPLGRITRTARAVAGSDLSRRIELDGPDDELKELA DTFDDMLERLQRAFTAQQRFVGNASHELRTPLAINRTLLEVHLSDPGAPVELQQLGKTLL ATNERSEQLVEGLLLLARSDNQIVERKPVDLAEVASQAIDQVHAEAQAKGVEIRGVRKPA VVRGNGVLLERIALNLLQNAVRYNVAEQGWVEVTTDVEHGQAVLVVSNTGPVVPAYEVDN LFEPFRRLRTERTGSDKGVGLGLSIVRSVARAHGGHIYAQPREGGGLVMRVTLPI  
>DWG14_RS11080 Streptomyces_griseorubiginosus_strain_3E-1.gbff Streptomyces griseorubiginosus recipro:1 VASTPAPPQAPPKPTWDPRRPAPQLPWLRPTIRIRLTLLYGGMFLIAGILLLSIIYLVAA QALNVGSKLPFEITTGSVTSDICRNLPSSGTSEVMNRAINACVNDQRQHALDDLLSRSLL ALLGLAIIAFAFGYAMAGRVLSPLGRILRTARAVAGSDLSRRIELDGPDDEIKELADTFD DMLERLERAFTAQQRFVGNASHELRTPLAINRTLLEVHLSDPGAPPELQQLGKTLLATNE RSEQLVEGLLLLARSDNQIVERKPVDLAEVAEQAVDQVRAEAEAKGVAIRSEQRSAVVQG NGVLLERIALNLVQNAVRYNVPEDGWVEVTTEVQHGQAVLTVSNTGPVVPAYEVDDLFEP FKRLRGADRTGSDKGVGLGLSIVRSVARAHGGHISAQPREGGGLVMRVTLPV  
>J2N69_RS27985 Streptomyces_huasconensis_strain_D23.gbff Streptomyces huasconensis recipro:1 VAATPAPPAAPPKPTWEPRKVEAPFPWLRPTIRIRLTLLYGGMFLIAGILLLSIIYLLAA QALNVGSDLPFRVTGKSSVTSDSCANFRLLPQRPTEAQLNAALNECVNTMRQNALDNLLS RSLLALLGLAVIAFAFGYAMAGRVLSPLGRITRTARRVAGTDLTRRIELDGPDDELKELS DTFDEMLDRLERAFTAQQRFVGNASHELRTPLAINRTLLEVHLSDPGAPPELHQLGKTLL ATNERSEQLVEGLLLLARSDNQIVERKPVDLAEVADRAVDQVRGEAEAKGVEIRGERGAA VVQGNGVLLERIALNLVQNAVRYNVPSQEGQGEALQEKGGGGRREGGWVEVTTEAQHGQA VLVVSNTGPVVPAYEIDNLFEPFRRLRTERTGSDKGVGLGLSIARSVARAHGGRIIAEPR EGGGLMMRVTLPI  
>DWB77_RS09870 Streptomyces_hundungensis_strain_BH38.gbff Streptomyces hundungensis recipro:1 MAATPAPPTAPPKPAWAPKSAEPPFPWLRPTIRIRLTLLYGGMFMIAGILLLSIIYLLAA QALHAGSAPFKIMGGQNITLSSGSCQLPASGTFQEINDAIGVCMAQQRQHALDNLLSRSL LALVGLSVIAFAFGYAMAGRVLSPLGRITRTARRVAGTDLTRRIELDGPDDELKELSDTF DEMLDRLERAFTAQQRFVANASHELRTPLAINRTLLEVHLSDPGAPVELQQLGKTLLATN ERSEQLVEGLLLLARSDNEIIDRKPVDLAEVASRAIDQARTEAEAKGVEIRGERAAAVVQ GNGVLLERIALNLVQNAVRYNAPEDGWVEVVTALKDGQATLIVTNTGPVVPAYEIDNIFE PFRRLRTERTGSDKGVGLGLSIARSVARAHGGRIIAEPREGGGLVMRVTLPV  
>RFN58_RS31475 Streptomyces_iakyrus_strain_CGMCC_4.1912.gbff Streptomyces iakyrus recipro:1 VATTPAPPQAPPKPTWDPRRPVPPFPWLRPTIRIRLTLLYGGMFLIAGILLLSIIYLLAA QALNVGSELPFKIVEGKVTSEICNLPDQASPAEFNNAMNHCVNEQRQNALDNLLSRSLLA LLGLAVIAFAFGYAMAGRVLSPLGRITRTARAVAGSDLSRRIELDGPDDELKELADTFDD MLERLQRAFTAQQRFVGNASHELRTPLAINRTLLEVHLSDPQAPPELQQLGKTLLATNER SEQLVEGLLLLARSDNQIVERGPVDLAEVAEQAIDQVRGEAAGKGVSIRGEQKPAVVQGN GVLLERIALNLVQNAVRYNVPEDGWVEVTTDVQHGQAVLVVANTGPVVPAYEIDNLFEPF RRLRTERTGSDKGVGLGLSIVRSVARAHGGHIYAQPREGGGLVMRVTLPI  
>CEB94_RS30235 Streptomyces_hawaiiensis_strain_ATCC_12236.gbff Streptomyces hawaiiensis recipro:1 VAASPAPPQAPPKPTWDPRRPVPPFPWLRPTIRIRLTLLYGGMFLIAGILLLSIIYLLAA QALNVGSELPFKIVEGKVTSDICNLPGQASPSEFNDAMNACVNEQRRNALDNLLSRSLLA LLGLAVIAFAFGYAMAGRVLSPLGRITRTARAVAGSDLSRRIELDGPDDELKELADTFDD MLERLQRAFTAQQRFVGNASHELRTPLAINRTLLEVHLSDPKAPMELQQLGKTLLATNER SEQLVEGLLLLARSDNQIVERGPVDLAEVASQAIDQVHAEAEGKGVTIRGEQKSAVVQGN GVLLERIALNLVQNAVRYNVAEGGWVEVTTDVQHGQAVLVVSNTGPVVPAYEIDNLFEPF RRLRTERTGSDKGVGLGLSIVRSVARAHGGHIYAQPREGGGLVMRVSLPI  
>KI385_RS32920 Streptomyces_inhibens_strain_NRRL30439.gbff Streptomyces inhibens recipro:1 MPSLPSFSKAATPPPPMPPKPTWDPRPVNVRPFPWLRPTIRIRLTLLYGGMFLMAGIVLL TIIYMLAADALHDGSALPLKILGGKFQSTSDICDLPTETSGQLLQEAVNQCLQHQRAVAL NSLLNRSLLALLGLTIVAFAFGYAMAGRVLSPLGRITRTAQRVAGSDLHRRIELGGPDDE LKELADTFDEMLDRLDRAFESQRRFVANASHELRTPLAINRTLLEVQLADPDASPELAQL GKTLLATNERSEQLVEGLLLLARSENKVVDKKPVDLSEVASQAVDQAREEAQTKGLELRG VRQQVFVQGNGVLLERIALNLVQNAVRYNIPEDGWVEVTTEPQPGCAVLVVTNTGPVVPA YEVENLFEPFRRLRTERTGSDKGVGLGLSIVRSVVRAHDGTITAEPREGGGLVVRVVLPL  
>RI060_RS10320 Streptomyces_janthinus_strain_JCM_4387.gbff Streptomyces janthinus recipro:1 VAATPAPPQAPPKPTWDPRRPAQPFPWLRPTIRIRLTLLYGGMFLIAGILLLSIIYLLAA EALNVGSELPFKIVEGKVTSDICNLPSQASPAEFNHAMNQCVNDQRKAALDNLLSRSLLA LLGLAVIAFAFGYAMAGRVLSPLGRITRTARAVAGSDLSRRIELDGPDDELKELADTFDD MLERLQRAFTAQQRFVGNASHELRTPLAINRTLLEVHLSDPGAPMELQQLGKTLLATNER SEQLVEGLLLLARSDNQIVERGPVDLAEVASQAIDQVHGEAEDKGVRIRGEQKPAVVQGN GVLLERIALNLVQNAVRYNVAEDGWVEVTTEVQHGQALLVVSNTGPVVPAYEIDNLFEPF RRLRTERTGSDKGVGLGLSIVRSVARAHGGHIYAQPREGGGLVMRVTLPI  
>V8J11_RS28500 Streptomyces_hygroscopicus_subsp._hygroscopicus_strain_DSM_41954.gbf f Streptomyces hygroscopicus subsp. recipro:1 MATTPTPLPPTVPPKPSWDPQHTSPPNPWLRPTIRIRLTLLYGGMFLIAGVVLLTIIYLL AADAIRRGSEFPLQIVRIDYKPSDTCHLPSQGNNDAFNRAVAQCMQQQRDYALDGLLRRS LIALLGLAVVAFAFGYAMAGRVLSPLGRITRTARQVAGSDLSRRIELDGPDDELKELADT FDEMLERLDRAFTAQQRFVANASHELRTPLAINRTLLEVQLSDPNAPPEVVQLGKTLLAT NERSEQLVEGLLLLARSDNEIVDRKPVDLAEVASQALEQVRAEAEDKGVELRGERGPAVV QGNGVLLERIALNLVQNAVRYNVAEGGWVEVTTESRQGQAVLVVANTGPVVPAYEMDNIF EPFRRLRTERTGSDKGVGLGLSIARSVARAHGGRIAAEPREGGGLVMRVVLPI  
>CP970_RS10850 Streptomyces_kanamyceticus_strain_ATCC_12853.gbff Streptomyces kanamyceticus recipro:1 VAATPAPPAAPPKPTWDPRKADPPFPWLRPTIRIRLTLLYGGMFLIAGILLLSIIYLLAA QALNVGSDLPFRIEGGSKVSSQTCHNLPAEARIPADQLNSVLNDCVNDLRQNALDNLLSR SLLALLGLAVIAFAFGYAMAGRVLSPLGRITRTARRVAGTDLSRRIELDGPDDELKELSD TFDEMLDRLERAFTAQQRFVGNASHELRTPLAINRTLLEVHLSDPAAPPELQQLGKTLLA TNERSEQLVEGLLLLARSDNQIVERKPVDLAEVANRAVDQVRSEADVKGVEIRGERALAV VQGNGVLLERIALNLVQNAVRYNVPQDGWVEVTTEAQHGQAVLVVSNTGPVVPAYEIDTL FEPFRRLRTERTGSDKGVGLGLSIARSVARAHGGRIIAEPREGGGLVMRVTLPL  
>NNW98_RS08465 Streptomyces_koelreuteriae_strain_CRLD-Y-1.gbff Streptomyces koelreuteriae recipro:1 VAATPAPPQAPPKPTWDPRRPAPPIPWLRPTIRIRLTLLYGGMFLIAGILLLSIIYLLAA QALNVGSELPFKIVEGKVTSDICNLPGQASPSEFNEAMNRCVNDQRQHALDNLLSRSLLA LLGLAVIAFAFGYAMAGRVLSPLGRITRTARTVAGSDLSRRIELDGPDDELKELADTFDD MLERLQRAFTAQQRFVGNASHELRTPLAINRTLLEVHLSDPGAPVELQQLGKTLLATNER SEQLVEGLLLLARSDNQIVERGPVDLAEVASQAIDQVHGEAEDKGVRIRGEQKPAVVQGN GVLLERIALNLVQNAVRYNVAEDGWVEVTTEIQHGQAILVVSNTGPVVPAYEIDNLFEPF RRLRTERTGSDKGVGLGLSIVRSVARAHGGHIYAQPREGGGLVMRVTLPI  
>G9U55_RS24385 Streptomyces_koyangensis_strain_SCSIO_5802.gbff Streptomyces koyangensis recipro:1 VAASPAPRPAPPKPTSAPPRPTWDPRRPPQQAWLRPTIRIRLTLLYGGMFLIAGILLLSI IYLLAAQAVHVGNELPFQVTNGTFTIYSDACPALSGRRFTSSGAFNDAMKLCIDHQRQQA LDDLLSRSLLALVGLSVIAFAFGYAMAGRVLAPLGRITRTARSVAGSDLSRRIELDGPDD ELKELADTFDDMLDRLERAFTAQQRFVANASHELRTPLAINRTLLEVQLSDPGAPPELQQ LGKTLLATNERSEQLVEGLLLLARSDNQIVERKPVDLGEVATRAVEQVRGEAEQREVELR ADIAEAVVQGNGVLLERIALNLTQNAVRYNLPPGEGGWVEVSTELQHGQAVLVVANTGPV VPAYEIDQLFEPFRRLRTERTGSDKGVGLGLSIVRSVTRAHGGRITAEPREGGGLVMRVT LPL  
>OHB09_RS25185 Streptomyces_jietaisiensis_strain_NBC_00521.gbff Streptomyces jietaisiensis recipro:1 VAATPAPPAAPPKPTWDPNRPEPPFPWLRPTIRIRLTLLYGGMFLIAGILLLSIIYLLAA QALRTGSEPLFKIVEFNDLKVTSNDCPGVNNKLSLTEFNDAISACTDHQRQVALDHLLSR SLLALLGLAVIAFAFGYAMAGRVLSPLGRITRTARAVAGSDLSRRIELDGPDDELKELAD TFDDMLERLQRAFTAQQRFVGNASHELRTPLAINRTLLEVHLSDPGAPVELQQLGKTLLA TNERSEQLVEGLLLLARSDNQIVERKPVDLAEVAAQAIDQVHAEAEAKGVEIRSTRAPAV VQGNGVLLERIALNLVQNAARYNVPEGGWVEVTTEVQHGQAVLVVSNTGPVVPAYEIDNL FEPFRRLRTERTGSDKGVGLGLSIVRSVARAHGGHISARPREGGGLVMRVTLPI  
>SLLC_RS08245 Streptomyces_lavendulae_subsp._lavendulae_strain_DelLP.gbff Streptomyces lavendulae subsp. recipro:1 MAATPAPPTAPPKPTWDPGQPEGPFPWLRPTIRIRLTLLYGGMFLIAGILLLSIIYLLAA QALREGNALPFKIVGGQKVEVSSTTCSGVGIDQSIDQFNAAIGQCILDQRKHALDDLLRR SLMALLGLSIIAFAFGYAMAGRVLSPLGKITRTARRVVGSDLTRRIELDGPDDELKELAD TFDEMLDRLERAFTAQQRFVANASHELRTPLAINRTLLEVHLSDPGAPVELQQLGKTLLA TNERSEQLVEGLLLLARSENQIVERKPVDLAEVASRAIDQARAEAVAKGVEIRGERALAV IQGNGVLLERIALNLVQNAVRYNVPEGGWVEVTTEVQHGQAVLLVSNTGPVVPAYEVDNL FEPFRRLRTERTGSDKGVGLGLSIARSVARAHGGRILATPREGGGLVMRVTLPL  
>HA039_RS07905 Streptomyces_liangshanensis_strain_QMT-12.gbff Streptomyces liangshanensis recipro:1 MASNPLPPSAPPKPTWDPRRPDQTLPWVRPTIRIRLTLLYGGMFLIAGILLLSIIYLLAA QALHVGSKLPFTLLSGAVSSDTCRLPAQLTPDEANAAINSCVNHQRAQALDDLLTRSLFA LVGLSIIAFAFGYAMAGRVLSPLGRITRTARRVVGSDLSRRIELDGPDDELKELADTFDE MLERLERAFTAQQRFVANASHELRTPLAINRTLLEVHLSDPGAPGELQQLGKTLLATNER SEQLVEGLLLLARSDNQLVERKPVDLAEVAARAVDQTAGEALAKNVVIRGERAPAVVQGN GVLLERIALNLVQNAVRYNVPEDGWVEVTTSMEPGQAVLVVSNTGPVVPAYEIDNLFEPF RRLRTERTGSDKGVGLGLSIARSVARAHGGRIIAEPREGGGLVMRVTLPV  
>BN2145_RS10410 Streptomyces_leeuwenhoekii_C34.gbff Streptomyces leeuwenhoekii recipro:1 VATTPPPPQAPPKPTWDPRSAAPFPWLRPTIRIRLTLLYGGMFLIAGILLLSIIYLLAAQ ALNVGSDLPFKIVEGKVTSDVCNLPDQASPSTFNSAMNDCVNEQRQHALDSLLSRSLLAL LGLAVIAFAFGYAMAGRVLSPLGRITRTARAVAGSDLSRRIELDGPDDELKELADTFDDM LERLQRAFTAQQRFVGNASHELRTPLAINRTLLEVHLSDPNAPVELQQLGKTLLATNERS EQLVEGLLLLARSDNQIIERKPVDVAEVATQAIDQVHAEAEAKGVRIRGERKPAVVQGNG VLLERIALNLVQNAVRYNVAENGWVEVTTDVQHGQAILVVSNTGPVVPAYEIDNIFEPFR RLRTERTGSDKGVGLGLSIVRSVARAHGGHISAQPREGGGLVMRVSLPV  
>P8A22_RS09170 Streptomyces_laculatispora_strain_Mut2.gbff Streptomyces laculatispora recipro:1 MPVPTVPPPATAPPKPTWEPKQQEPPYPWLRPTIRIRLTLLYGGMFLIAGILLLSIIYML AAQALHVGSELPFKIVDGHVSSKVCNLLGEGRSPDSVNAAMNSCVNHQRQQALDTLLNRS LLALVGLSIIAFAFGYAMAGRVLSPLGRITRIARRVAGTDLSRRIELDGPDDELKELADT FDDMLDRLERAFTAQQRFVGNASHELRTPLAINRTLLEVHLSDPQAPPELKQLGKTLLAT NERSEQLVEGLLLLARSDNQIVERKPVDLAEVASRALDQTRGEAEAKGVELRDELAPAVV QGNGVLLERIALNLVQNAVRYNVPDGGWVEVTTELLPGQALLVVSNTGPVVPGYEIDNLF EPFRRLRTERTGSDKGVGLGLSIARSVARAHGGRIIAEPREGGGLVMRVSLPV  
>JEQ17_RS13195 Streptomyces_liliifuscus_strain_ZYC-3.gbff Streptomyces liliifuscus recipro:1 VATTPAPPTAPPKPTWDPRKPEPPFPWLRPTIRIRLTLLYGGMFLIAGILLLSIIYLLAA QALNVGSDLPFRIVSGGVTSPTCNFPSEPSATELNNAMNACVNEQRQHALDNLLSRSLLA LLGLAVIAFAFGYAMAGRVLSPLGRITRTARAVAGSDLSRRIELDGPDDEFKELADTFDD MLERLQRAFTAQQRFVGNASHELRTPLAINRTLLEVHLSDPGAPVELQQLGKTLLATNER SEQLVEGLLLLARSDNQIVERKPVDLAEVASQAIDQARGEAEAKGVAIRGKRDAAVVQGS GVLLERIALNLVQNAVRYNVAEEGWVEVTTEVQHGQAVLVVSNTGPVVPAYEIDNLFEPF RRLRTERTGSDKGVGLGLSIARSVARAHGGHIAAEPREGGGLVMRVTLPV  
>LGI35_RS32270 Streptomyces_longhuiensis_strain_BH-MK-02.gbff Streptomyces longhuiensis recipro:1 VASTPAPPAAPPKPTWDPRKAEPPYPWLRPTIRIRLTLLYGGMFLIAGIVLLSIIYLLAA QALNVGSDLPFTVTNGSVSSGTCRNFSGLPDHPTTGQLNSALNECVNEMRQHALDNLLSR SLLALLGLAVIAFAFGYAMAGRVLSPLGRITRTARRVAGTDLSRRIELDGPDDELKELAD TFDDMLDRLERAFTAQQRFVGNASHELRTPLAINRTLLEVHLSDPGAPMELQQLGKTLLA TNERSEQLVEGLLLLARSDNQIVERKPVDLAEVASQAIDQARSEADAKGVEIRGERKEAV VQGNGVLLERIALNLVQNAVRYNVPEGGWVEVATEAQHGQAVLVVSNTGPVVPAYEIDNL FEPFRRLRTERTGSDKGVGLGLSIARSVARAHGGRIIAEPREGGGLVMRVTLPI  
>BJ961_RS09055 Streptomyces_lienomycini_strain_DSM_41475.gbff Streptomyces lienomycini recipro:1 VATTPAPPGAPPKPTWDPRSATPLPWLRPTIRIRLTLLYGGMFLIAGILLLSIIYLLAAQ AVRTGNEPLYKIVDFTDLKVSSSTCPVVDNGNLSLSDFNAAISDCMDHQRKVALDNLLSR SLLALLGLAVIAFAFGYAMAGRVLSPLGRITRTARAVAGSDLSRRIELDGPDDELKELAD TFDDMLERLQRAFTAQQRFVGNASHELRTPLAINRTLLEVHLSDPNAPVELQQLGKTLLA TNERSEQLVEGLLLLARSDNQIVERKPVDLAEVAGQAIDQVHAEAESKGVEIRGTREAAV VQGNGVLLERIALNLVQNAVRYNVAEQGWVEVATAVENGQAVLVVTNTGPVVPAYEVDNL FEPFRRLRTERTGSDKGVGLGLSIARSVARAHGGHIYAQPREGGGLVMRVALPV  
>SLCG_RS11660 Streptomyces_lincolnensis_strain_LC-G.gbff Streptomyces lincolnensis recipro:1 MAATPAPPQAPPKPTWDPRRPEPPFPWLRPTIRIRLTLLYGGMFLIAGILLLSIIYLLAA QALTSGNQPPFKIAGGENIKVISDKCPAVNGATDLPLDTFNTIISKCIDQQRHASLDNLL SRSLLALLGLAIIAFAFGYAMAGRVLSPLGRITRTARAVAGSDLSRRIELDGPDDELKEL ADTFDEMLERLQRAFTAQQRFVGNASHELRTPLAINRTLLEVHLSDPGAPMELQQLGKTL LATNERSEQLVEGLLLLARSDNQIVERKPVDLAEVAEQAVDQVNGEAEAKGVVIRGEQKP AVVQGNGVLLERIALNLVQNAVRYNVPEGGWVEVTTDIQHGQAVLVVSNTGPVVPAYEID NLFEPFRRLRTERTGSDKGVGLGLSIVRSVARAHGGHISAQPREGGGLVMRVALPI  
>BXT88_RS28370 Streptomyces_lydicus_strain_GS93_isolate_23.gbff Streptomyces lydicus recipro:1 MPSLPSFSKPAAPPPPMPPKPTWDPRPVNVRPFPWLRPTIRIRLTLLYGGMFLMAGIVLL TIIYMLAADALHDGSALPLKILGGKFESTSDICDLPTQTSGPLLQQAVEQCLQHQRALAL NSLLNRSLLALLGLTVVAFAFGYAMAGRVLSPLGRITRTAQRVAGSDLHRRIELGGPDDE LKELADTFDEMLDRLDRAFESQRRFVANASHELRTPLAINRTLLEVQLADPEASPELAQL GKTLLATNERSEQLVEGLLLLARSENKVVDKKPVDLSEVAAQAVDQSREEAQAKGVSLRG VRQQVFVQGNGVLLERIALNLVQNAVRYNVPDGWVEVSTEPLPGCAVLVVANTGPVVPAY EVENLFEPFRRLRTERTGSDKGVGLGLSIVRSVARAHDGTITAEPRDGGGLVMRVVLPL  
>HUT09_RS27310 Streptomyces_microflavus_strain_NA06532.gbff Streptomyces microflavus recipro:1 MTTTPDPPVAPPKPTWEPKQQEPLYPWLRPTIRIRLTLLYGGMFLIAGIMLLSIIYMLAA QGLHVGNELPFKLLPDSKIQLTSNACPALTPGLSADEANAALKACNGEQRQQALDTLLNR SLLALVGLSIIAFAFGYAMAGRVLSPLGRITRTARRVAGTDLTRRIELDGPDDELKELAD TFDDMLDRLERAFTAQQRFVGNASHELRTPLAINRTLLEVHLSDPEAPPELQQLGKTLLA TNERSEQLVEGLLLLARSDNQIIERKPVDLAEVADRAIDQARSEAVEKNVEIRGERSAAV VQGNGVLLERIALNLVQNAVRYNVAEDGWVEVTTSLQTGQALLVVSNTGPVVPAYEIDNL FEPFRRLRTERTGSDKGVGLGLSIARSVARAHGGRIIAEPREGGGLVMRVTLPV  
>CAG99_RS03920 Streptomyces_marincola_strain_SCSIO_03032.gbff Streptomyces marincola recipro:1 MAPPAPLPSFPSHPPMPAAPPRPHWDPQDTDRPRSWLRPTIRIRLTLLYGGMFLIAGVVL LTIIYLLAARALERGTELPFTFPPGTTTVQITSPVCPASAGPVEGEQFERWLAECTDKQR AIALDQLLRSSLLALLGLAVAAFAFGYVMAGRVLSPLGRITRTARRVAGSDLHRRIELDG PDDELKELADTFDEMLDRLDRAFTAQQRFVANASHELRTPLAINRTLLEVQLSDPDATPE LTQLGNTLLATNERSEQLVEGLLLLARSENELVDRKPVDLAEVADRAVDQARGEARQRGV RLTGARPPLYVHGNGVLLERVALNLVQNAVRYNEPEDGWVRVETEARPGQAVLVVENTGP VVPAYEVDNMFEPFRRLRTQRTGSDRGVGLGLSIVRSVARAHGGYVRAEPRENGGLVMRV VIPV  
>SLUN_RS29760 Streptomyces_lunaelactis_strain_MM109.gbff Streptomyces lunaelactis recipro:1 MATAPAPPQAPPKPTWDPRDPVRPLLRPTIRIRLTLLYGGMFLIAGILLLSIIYLLAAQA LHIGSELPFKIVNGSVKSDICNFPSEAPPEQFNAAMNACVNEQRQHALDDLLRRSLFALL GLSVIAFAFGYAMAGRVLSPLGKITRTARQVAGSDLTRRIKLDGPDDELKELADTFDEML DRLERAFSAQQRFVANASHELRTPLAINRTLLEVHLSDPGVPAELQQLGKTLLATNKRSE QLVEGLLLLARSDNQIVERKPVDLAEVATRAVDQVRAEAEANGVEIRGERAPAVVQGNGV LLERIALNLVQNAVRYNIPEGGWVHVSTELQQGEAVLVVENTGPVVPAYEIDNLFEPFRR LRQERTGSDKGVGLGLSIARSVARAHRGRIIAEPREGGGLVMRVTLPV  
>K7I03_RS07765 Streptomyces_mobaraensis_strain_DSM_40587.gbff Streptomyces mobaraensis recipro:1 MASTPPSPANTPPAASQPPPRPAAPPRPAWDPRQPVVRPFPWLRPTIRIRLTLLYGGMFL MAGVLLLTIIYLLAADAMREGSYLPFRFIDGNLESTSNACPSLRPGRIDARELENAVQVC MNHQRAIALNGLLRRSLLALLGLAVIAFAFGYAMAGRVLSPLGKITRTARRVAATDLTRR IELGGPDDELKELSDTFDEMLDRLERAFTAQQRFVANASHELRTPLAINRTLLEVHLSDP GASPELQQLGKTLLATNERSEQLVEGLLLLARSDNEIVDRKPVDLAEVASQALDQARGEA QTKGVELRGERLPAVVQGNGVLLERVALNLVQNAVRYNVPDDGWVEVTTESRPGEAVLVV ENTGPVVPAYELDNIFEPFRRLRTERTGSDKGVGLGLSIVRSVARAHGGRITAEPREGGG LVMRVVLPV  
>WEB32_RS25725 Streptomyces_netropsis_strain_M-14.gbff Streptomyces netropsis recipro:1 MPTPATPAPPATPPTPPKPTWDPRQPVVRPFPWLRPTIRIRLTLLYGGMFLMAGVLLLTI IYLLAADALRQGNDLPFKILNAQVEFTSDTCSNLSSSRESGAFMRGLEQCMQTQRAMALS SLLKRSLLALLGLAVIAFAFGYAMAGRVLSPLGKMTRTARRVAGTDLSRRIELGGPDDEL KELSDTFDEMLDRLERAFTAQQRFVANASHELRTPLAINRTLLEVHLSDPGASPELQQLG KTLLATNERSEQLVEGLLLLARSDNEIVDRKPVDLAEVASQALDQARAEAQGKGVELRGT REPAVVQGNGVLLERIALNLVQNAVRYNTPDGWVEVTTRSLPGQALLVVENTGPIVPAYE VDNIFEPFRRLRTERTGSDKGVGLGLSIVRSVARAHGGRITAEPREGGGLIMRVVLPT  
>JGK51_RS09955 Streptomyces_murinus_strain_Am1.gbff Streptomyces murinus recipro:1 MATTPAPPQAPPKPTWDPRRPQPPFPWLRPTIRIRLTLLYGGMFLIAGILLLSIIYLLAA QAISTGNQPLFKIVSFQELKVSSDNCPAINTTSLPLADFNNTISQCVDHQRQVALDNLLS RSLLALLGLAVIAFAFGYAMAGRVLSPLGRITRTARAVAGSDLSRRIELDGPDDELKELA DTFDEMLERLQRAFTAQQRFVGNASHELRTPLAINRTLLEVHLSDPGAPVELQQLGKTLL ATNERSEQLVEGLLLLARSDNQIVERKPVDLAEVAGQAIDQVRSEAEAKGVEIRGERAPA VVQGNGVLLERIALNLVQNAVRYNVAEAGWVEVDTEVQHGQAVLTVSNTGPVVPAYEIDN LFEPFRRLRTERTGSDKGVGLGLSIVRSVARAHGGHIVARPREGGGLVMRVTLPL  
>NLG24_RS14855 Streptomyces_milbemycinicus_strain_SIPI-054.gbff Streptomyces milbemycinicus recipro:1 MATTPIPPTAPPKPSWDPRPAATPRPWLRPTIRIRLTLLYGGMFLIAGVVLLTIIYLLAA DVLQGSSKLPFQIVKMDYSLTDPTCELPSQSSTTLFNEAVARCMNHQRALALDALLRRSL LALLGLAVVAFAFGYAMAGRVLSPLGRITRTARQVAGSDLARRIKLDGPDDELKELADTF DEMLERLDRAFTAQQRFVANASHELRTPLAINRTLLEVQLSDPQASPDLTQLGKTLLATN ERSEQLVEGLLLLARSDNEIVDRKPVDLAEVASQALEQVRTEAQDKGVAVRGVRQSAVVQ GNGVLLERIALNLIQNAVRYNIPQGGWVEVTTEVREGAGQAVLVVANTGPVVPAYELDNI FEPFRRLRTERTGSDKGVGLGLSIARSVARAHGGQITAEPRDGGGLVMRVVLPV  
>STRNI_RS11980 Streptomyces_nigrescens_strain_DSM_40276.gbff Streptomyces nigrescens recipro:1 MPSLPSFSKAAGPPPSAPPKPTWDPRPVNVRPFPWLRPTIRIRLTLLYGGMFLMAGIVLL TIIYMLAADALHDGSALPLKILGGKFQSTSDICDLPTETSGQLLQEAVNSCLQHQRAVAL NSLLNRSLLALLGLTVVAFAFGYAMAGRVLSPLGRITRTAQRVAGSDLHRRIELGGPDDE LKELADTFDQMLDRLDRAFESQRRFVANASHELRTPLAINRTLLEVQLADPAASPELAQL GKTLLATNERSEQLVEGLLLLARSENKVVDKKPVDLSEVAAQAVDQTREEAHAKGVELRG VRHQVLVQGNGVLLERIALNLVQNAVRYNVPEEGWVEVVTEPQPGCAVLVVANTGPVVPA YEVENLFEPFRRLRTERTGSDKGVGLGLSIVRSVVRAHDGTITAQPREGGGLVMRVVLPL  
>CP967_RS07360 Streptomyces_nitrosporeus_strain_ATCC_12769.gbff Streptomyces nitrosporeus recipro:1 VPPSPAPVKAPPKPTWEPKPQDSPYPWLRPTIRIRLTLLYGGMFLIAGILLLSIIYMLAA QALDVGSDLPFEVVNGQVSSEVCDLPTKATPEAFNDALNACANQQRDHALDTLLNRSLLA LVGLSVIAFAFGYAMAGRVLSPLGRITRTARRVVGTDLTRRIELDGPDDELKELADTFDE MLDRLERAFTAQQRFVGNASHELRTPLAINRTLLEVHLSDPEAPPELHQLGRTLLATNER SEQLVEGLLLLARSDNQIVERKPVDLAEVAGRAIDQTRAEAATRGVEIRGERAPAVVQGN GVLLERIALNLVQNAVRYNTAENGWVEVGTELQHGQALLTVSNTGPVVPAYEIDNLFEPF RRLRTERTGSDKGVGLGLSIARSVARAHGGRIIAEPREGGGLVMRVTLPV  
>OG275_RS29095 Streptomyces_niveus_strain_NBC_01447.gbff Streptomyces niveus recipro:1 VATTPAPPTAPPKPTWDPRRPDQALPWVRPTIRIRLTLLYGGMFLIAGILLLSIIYLLAA QAINEGSELALKVTGVNVQLTSPTCPGLNEAVNNDQLNSSLKACMAAQRQQALDDLLTRS LFALTGLSIIAFAFGYAMAGRVLSPLGRITRTARRVVGSDLSRRIELDGPDDELKELADT FDEMLERLERAFTAQQRFVANASHELRTPLAINRTLLEVQLSDPAAPVELQQLGRTLLAT NERSEQLVEGLLLLARSDNQLVERKPVDLAEVATRAVDQTRGEAEAKGVELRGECVPAVV QGNGVLLERIALNLVQNAVRYNVKEDGWVEVTTAVEHGEAVLLVSNTGPVVPAYEIDNIF EPFRRLRTERTGSDKGVGLGLSIARSVARAHGGRIIAEPREGGGLVMRVTLPV  
>DC008_RS26455 Streptomyces_nigra_strain_452.gbff Streptomyces nigra recipro:1 MAATPAPPATPPKPTWDPRKPDQPFPWLRPTIRIRLTLLYGGMFLIAGILLLSIIYLLAA QALRTGNEPLFKIVQFQSLKVTSNNCPGIATSDLSLAEFNDAINACMDHERKAALDNLLS RSLLALLGLAVIAFAFGYAMAGRVLSPLGRITRTARAVAGSDLSRRIELDGPDDELKELA DTFDEMLERLQRAFTAQQRFVGNASHELRTPLAINRTLLEVHLSDPGAPVELQQLGKTLL ATNERSEQLVEGLLLLARSENQIIERKPVDLAEVAEQAVDQVHTEADAKGVTIRGERKPA VIQGNGVLLERIALNLVQNAVRYNVPEGGWVEVTTDVEHGQAVLDVTNTGPVVPAYEIDN LFEPFRRLRTERTGSDKGVGLGLSIVRSVARAHGGHIAAEPREGGGLVMRVTFPV  
>JYK04_RS31245 Streptomyces_nojiriensis_strain_JCM_3382.gbff Streptomyces nojiriensis recipro:1 MATTPAPPAAPPKPTWDPGQPEGPFPWLRPTIRIRLTLLYGGMFLIAGILLLSIIYLLAA QALRQGNAIPFTIVRGQNIEVTSTTCSGVSGINQPFEQFTAAINQCVLEQRRHALDDLLS RSLMALLGLSIIAFAFGYAMAGRVLSPLGKITRTARRVVGSDLTRRIELDGPDDELKELA DTFDDMLDRLERAFTAQQRFVANASHELRTPLAINRTLLEVHLSDPGAPIELQQLGKTLL ATNERSEQLVEGLLLLARSENQIVERKPVDLAEVASRAVDQVRGEAEAKGVEIRGERAPA VVQGNGVLLERIALNLVQNAVRYNVPEDGWVEVVTEAQHDQAVLLVSNTGPVVPAYEVDN LFEPFRRLRTERTGSDKGVGLGLSIARSVARAHGGRIQAMPREGGGLVMRVTLPL  
>FYC74_RS09870 Streptomyces_olivaceus_strain_SCSIO_T05.gbff Streptomyces olivaceus recipro:1 VATTPAPPGAPPKPTWDPRSATPLPWLRPTIRIRLTLLYGGMFLIAGILLLSIIYLLAAQ AVRSGNQPLYKIVDFTDLRVSSSDCPVVDNGNLSLSDFNAAISDCMDHQRKVALDNLLSR SLLALLGLAVIAFAFGYAMAGRVLSPLGRITRTARAVAGSDLSRRIELDGPDDELKELAD TFDDMLERLQRAFTAQQRFVGNASHELRTPLAINRTLLEVHLSDPNAPVELQQLGKTLLA TNERSEQLVEGLLLLARSDNQIVERKPVDLAEVAGQALDQAHGEAEAKGVEIRGTRQAAV VQGNGVLLERIALNLVQNAVRYNVAEQGWVEVTTSLENGQAVLVVTNTGPVVPAYEVDNL FEPFRRLRTERTGSDKGVGLGLSIARSVARAHGGHIYAQPREGGGLVMRVALPV  
>JNO44_RS31180 Streptomyces_noursei_strain_A-2-1.gbff Streptomyces noursei recipro:1 MPSLPSFSSSKSAPPPPPVPPKPAWDPKPVNVRPFPWLRPTIRIRLTLLYGGMFLMAGIV LLTIIYMLAAAALHDGSALPLKILGGKFQSTSDICDLPTETSGALLQEAVNNCLMHQRAV ALNNLLNRSLLALLGLTIVAFAFGYAMAGRVLSPLGRITRTAQRVAGSDLHRRIELGGPD DELKELADTFDEMLDRLDRAFESQRRFVSNASHELRTPLAINRTLLEVQLADPQASPEVQ QLGKTLLATNERSEQLVEGLLLLARSENKIVDKRPVDLSEVASQAVDQTREEAQAKGVQL RGVRQQVFVQGNGVLLERIALNLVQNAVRYNVPEGGWVEVTTEPQPGCAVLVVSNTGPVV PAYEVENLFEPFRRLRTERTGSDKGVGLGLSIVRSVVRAHDGTITAEPREGGGLDMRVVL PL  
>J0917_RS09775 Streptomyces_nodosus_strain_ZJB2016050.gbff Streptomyces nodosus recipro:1 MAATPAPPAAPPKPTWDPRRPEPPFPWLRPTIRIRLTLLYGGMFLIAGILLLSIIYLFAA HALNVGSQLPFRITSGTVVSNTCNFPSQTTASDLNNAMNACVNEQRQHALDNLLSRSLLA LLGLAVIAFAFGYAMAGRVLSPLGRITRTARAVAGSDLSRRIELDGPDDELKELADTFDE MLERLQRAFTAQQRFVGNASHELRTPLAINRTLLEVHLSDPGAPVELQQLGKTLLATNER SEQLVEGLLLLARSDNQIVERKPVDLAEVASQAIDQVHGEAETKGVEIRGERAPAVVQGN GVLLERIALNLVQNAVRYNVPEDGWVEVTTELQHGQAVLTVSNTGPVVPAYEIDNLFEPF TRLRTERTGSDKGVGLGLSIARSVARAHGGHITAQPREGGGLVMRVVLPV  
>DVK44_RS26935 Streptomyces_paludis_strain_GSSD-12.gbff Streptomyces paludis recipro:1 VATIPPPPAAPPKPTWDPRRPEQSMPWVRPTIRIRLTLLYGGMFLIAGILLLSIIYLLAA QALHVGSELPFKIVSGKVSSDVCNFPDSAQPADFNIAMNACVNHQRQQALDDLLSRSLLA LVGLSVIAFAFGYAMAGRVLSPLGRITRTARRVVGTDLARRIELDGPDDELKELADTFDE MLERLERAFTAQQRFVANASHELRTPLAINRTLLEVHLSDPGAPMELQQLGKTLLATNER SEQLVEGLLLLARSDNQLVERKPVDLAEVATRAVDQALSEAEAKGVEIRGERVPAVVQGN GVLLERIALNLVQNAVRYNIPEGGWVEVVTESPPGQAVLVVSNTGPVVPAYEIDNLFEPF RRLRTERTGSDKGVGLGLSIARSVARAHGGRIFAEPREGGGLMMRVTLPV  
>Spa2297_RS24835 Streptomyces_parvulus_strain_2297.gbff Streptomyces parvulus recipro:1 VATTPAPPGAPPKPTWDPRSATPLPWLRPTIRIRLTLLYGGMFLIAGILLLSIIYLLAAQ AVRTGNEPLYKIVDFTDLRVSSSDCPVVDNGNLSLSAFNAAISDCIDHQRKVALDNLLSR SLLALLGLAVIAFAFGYAMAGRVLSPLGRITRTARAVAGSDLSRRIELDGPDDELKELAD TFDDMLERLQRAFTAQQRFVGNASHELRTPLAINRTLLEVHLSDPNAPVELQQLGKTLLA TNERSEQLVEGLLLLARSDNQIVERKPVDLAEVAGQAIDQVHAEAESKGVVVRGTREAAV VQGNGVLLERIALNLVQNAVRYNVAEDGWVEVTTAVEGGQAVLVVTNTGPVVPAYEVDNL FEPFRRLRTERTGSDKGVGLGLSIARSVARAHGGHIYAQPREGGGLVMRVTLPI  
>F9278_RS36905 Streptomyces_phaeolivaceus_strain_GY16.gbff Streptomyces phaeolivaceus recipro:1 VAATPAPPQAPPKPTWDPRRGQNPLPWLRPTIRIRLTLLYGGMFLIAGILLLSIIYLLAA QAVSTGNTPVFKIEGGTNISVSSNICPAVDADTAPTNLQLDDFNRAIAACIDHERQVALD TLLSRSLLALLGLAVIAFAFGYAMAGRVLAPLGRITRTARAVAGSDLSRRIELDGPDDEL KELADTFDDMLERLQRAFTAQQRFVGNASHELRTPLAINRTLLEVHLSDPGAPAELQQLG KTLLATNERSEQLVEGLLLLARSDNQIVERKPVDLAEVASQAIDQVRSEADAKKVEIRGE WAPAVVQGNGVLLERIALNLVQNAVRYNVPGPGGWVEVTTELQHGQAVLVVTNTGPVVPA YEIDNLFEPFRRLRTERTGSDKGVGLGLSIVRSVARAHGGHIAARPREGGGLVMRVTLPV  
>DXZ73_RS31040 Streptomyces_olivoreticuli_subsp.olivoreticuli_strain_ATCC_31159.gbf f Streptomyces olivoreticuli subsp. recipro:1 MPATPAPPAAPHTPPKPTWDPRQPVVRPFPWLRPTIRIRLTLLYGGMFLMAGVLLLTIIY LLAAHALDAGSTPLIKLNNGSQVTVPDSSLCPALRPGLTADEANNVLSNCTSHLRALALD SLLKRSLLALLGLAVIAFAFGYAMAGRVLSPLGKMTRTARRVAATDLSRRIELGGPEDEL KELSDTFDEMLDRLERAFTAQQRFVANASHELRTPLAINRTLLEVHLSDPGASPELQQLG KTLLATNERSEQLVEGLLLLARSDNEIVDRKPVDLAEVASQAIDQTRAEAQAKGVELRGT RQPAVVQGNGVLLERIALNLVQNAVRYNTADGWVEVTTEAQPGRAVLVVENTGPIVPAYE VDNIFEPFRRLRTERTGSDKGVGLGLSIVRSVARAHGGRITAEPRDGGGLIMRVVLPT  
>CP981_RS28955 Streptomyces_platensis_strain_ATCC_23948.gbff Streptomyces platensis recipro:1 MPSLPSFSKTAGPPPAAPPKPTWDPRPVNVRPFPWLRPTIRIRLTLLYGGMFLMAGIVLL TIIYMLAADALHDGSALPLKILGGKFESTSDICDLPTQTSGQLLQEAVNSCLQHQRAVAL NSLLNRSLLALLGLTVVAFAFGYAMAGRVLSPLGRITRTAQRVAGSDLHRRIELGGPDDE LKELADTFDQMLDRLDRAFESQRRFVANASHELRTPLAINRTLLEVQLADPAASPELAQL GKTLLATNERSEQLVEGLLLLARSENRVVDKKPVDVSEVAAQAVDQTREEAHAKGVELRG ARQQVFVQGNGVLLERIALNLVQNAVRYNVPEGGWVEVVTEPQPGCAVLVVANTGPVVPA YEVENLFEPFRRLRTERTGSDKGVGLGLSIVRSVVRAHDGSITAQPREGGGLIMRVVLPL  
>LK06_RS25455 Streptomyces_pluripotens_strain_MUSC_135.gbk.gbff Streptomyces pluripotens recipro:1 VAATPAPPQAPPKPTWDPRRPQPPFPWLRPTIRIRLTLLYGGMFLIAGILLLSIIYLLAA QAISTGNQPLFKIVSFQELKVSSDSCPAINTKSLALSDFNDAIAHCVDQQRQAALDNLLS RSLLALLGLAVIAFAFGYAMAGRVLSPLGRITRTARAVAGSDLSRRIELDGPDDELKELA DTFDDMLERLQRAFTAQQRFVGNASHELRTPLAINRTLLEVHLSDPNAPVELQQLGKTLL ATNERSEQLVEGLLLLARSDNQIVERKPVDLAEVATQAIDQVHAEAEAKGVEIRGERKPA VVQGNGVLLERIALNLVQNAVRYNVPESGWVEVETGVEHGQAVLTVTNTGPVVPAYEIDN LFEPFRRLRTERTGSDKGVGLGLSIVRSVARAHGGHIAARPREGGGLVMRVTLPL  
>CP972_RS26510 Streptomyces_prasinus_strain_ATCC_13879.gbff Streptomyces prasinus recipro:1 VAAAPTPTPPSPPAPPKPTWDPRRPVPPFPWLRPTIRIRLTLLYGGMFLIAGILLLSIIY LLAANALNVGSDLPFEILSGQVRSDICQLRSAQLPAGELNAALNDCVNEQRQHALDNLLS RSLLTLLGLAVIAFAFGYAMAGRVLSPLGRITRTARAVAGSDLSRRIELDGPDDELKELA DTFDDMLERLQRAFTAQQRFVGNASHELRTPLAINRTLLEVHLSDPDAPVELQQLGKTLL ATNERSEQLVEGLLLLARSDNQIVERKPVDLAEVASQAVDQVHAEAQAKGVEIRGVQKPA VVRGNGVLLERIALNLVQNAVRYNVAEGGWVEVTTDVEHGQAVLVVSNTGPVVPAYEIDN LFEPFRRLRTERTGSDKGVGLGLSIVRSVARAHGGHIYAQPREGGGLVMRVTLPI  
>NFX46_RS09135 Streptomyces_phaeoluteigriseus_strain_Qhu-M197.gbff Streptomyces phaeoluteigriseus recipro:1 VATTPAPPQAPPKPTWDPRRPQAPFPWLRPTIRIRLTLLYGGMFLIAGILLLSIIYLLAA NALNVGSDLPFKILAGQVASDICNFPNEPTASELNRAMNECANQQRQHALDDLLSRSLLA LLGLAVIAFAFGYAMAGRVLAPLGRILRTARSVAGSDLSRRIELDGPDDELKELADTFDD MLERLQRAFTAQQRFVGNASHELRTPLAINRTLLEVHLSDPDAPVELQQLGKTLLATNER SEQLVEGLLLLARSDNQIVERKPVDLAEVAGQAIDQLHGEAEVKGVKIRGKRDSAVVQGN GVLLERIALNLVQNAVRYNVPEDGWVEVTTEVLHGQAVLVVSNTGPVVPAYEIDNLFEPF RRLRTERTGSDKGVGLGLSIARSVARAHGGHIAAQPREGGGLVMRVSLPL  
>SPRI_RS09970 Streptomyces_pristinaespiralis_strain_HCCB_10218.gbff Streptomyces pristinaespiralis recipro:1 VATVPAPPEAPPKPTWDPREPVRPWLRPTIRIRLTLLYGGMFLIAGILLLSIIYLFTAQA LHVGVADLPFKIVEGKVQPTTDWCTLPEEGSGEQFNQAVSACLQHQRELALDDLLRRSLF ALLGLSIIAFAFGYAMAGRVLSPLGRITRTARQVAGSDLSRRIELDGPDDELKELADTFD EMLDRLERAFTAQQRFVANASHELRTPLAINRTLLEVHLSDPGAPVELQQLGKTLLATNE RSEQLVEGLLLLARSDNQIVERKPVDLAEVASRAMDQVRSEADEKGVEVRGERRAAVVQG NGVLLERIALNLVQNAVRYNVPGGWIEVITEVEHGQAVLLVTNTGPVVPAYEIDNLFEPF RRLRQERTGSDKGVGLGLSIARSVARAHGGRISAVPREGGGLVMRVTLPV  
>OHO81_RS12125 Streptomyces_pseudovenezuelae_strain_NBC_00598.gbff Streptomyces pseudovenezuelae recipro:1 VASTPAPPQAPPKPTWDPRRPAPPLPWLRPTIRIRLTLLYGGMFLIAGILLLSIIYLVAA QALNVGSELPFKITTGRVASDVCNLPTDVPADVMNKAMDSCVNDQRQHALDNLLSRSLLA LLGLAIIAFAFGYAMAGRVLSPLGRILRTARAVAGSDLSRRIELDGPDDELKELADTFDD MLERLERAFTAQQRFVGNASHELRTPLAINRTLLEVHLSDPGAPVELQQLGKTLLATNER SEQLVEGLLLLARSDNQIVERKPVDLAEVAEQAVDQVHGEAAAKGVAIRGEQKSAMVQGN GVLLERVALNLVQNAVRYNVPEDGWVEVTTAVEHGHAVLTVSNTGPVVPAYEVDNLFEPF KRLRGADRTGSDKGVGLGLSIVRSVARAHGGHITAQPREGGGLVMRVTLPV  
>A4E84_RS30100 Streptomyces_qaidamensis_strain_S10.gbff Streptomyces qaidamensis recipro:1 VAATPAPPQAPPKPTWDPRRPVPPFPWLRPTIRIRLTLLYGGMFLIAGILLLSIIYLLAA QALNVGSELPFKIVEGKVTSDICNLPDQASPAEFNNAMNHCVNEQRQNALDNLLSRSLLA LLGLAVIAFAFGYAMAGRVLSPLGRITRTARAVAGSDLSRRIELDGPDDELKELADTFDD MLERLQRAFTAQQRFVGNASHELRTPLAINRTLLEVHLSDPGAPTELQQLGKTLLATNER SEQLVEGLLLLARSDNQIVERGPVDLAEVAEQAIDQVRGEAAGRGVSIRGEQKPAVVQGN GVLLERIALNLVQNAVRYNVAEDGWVEVTTDVQHGQAVLVVANTGPVVPAYEIDNLFEPF RRLRTERTGSDKGVGLGLSIVRSVARAHGGHIYAQPREGGGLVMRVTLPI  
>HED23_RS09090 Streptomyces_pratensis_strain_S10.gbff Streptomyces pratensis recipro:1 MPASPAPVKAPPKPTWEPKAQESPYPWLRPTIRIRLTLLYGGMFLIAGILLLSIIYMLAA QALDVGSDLPFEVVNGQVTSEICDLPVKASPDDFNAALNVCANQQRDHALDDLLNRSLLA LVGLSVIAFAFGYAMAGRVLSPLGRITRTARRVVGTDLTRRIELDGPDDELKELADTFDE MLDRLERAFTAQQRFVGNASHELRTPLAINRTLLEVHLSDPEAPPELHQLGKTLLATNER SEQLVEGLLLLARSDNQIIERKPVDLAEVADRAIDQTMAEAAAKGVEVRGERAQAVVQGN GVLLERIALNLVQNAVRYNVAEDGWVEVTTELQHGQALLVVTNTGPVVPAYEIDNLFEPF RRLRTERTGSDKGVGLGLSIARSVARAHGGRIIAEPREGGGLVMRVTLPV  
>FGW37_RS24395 Streptomyces_rectiverticillatus_strain_B15-08.gbff Streptomyces rectiverticillatus recipro:1 MASAPTPTPPATPPKPTWDPRQPVVRPFPWLRPTIRIRLTLLYGGMFLMAGVLLLTIIYL LAADALTQGNQLPFRILNANVETSASCRSLPQGSATNSDTFMNGLRLCMEHQRAMALDGL LKRSLLALLGLAVIAFAFGYAMAGRVLSPLGRMTRTARRVAATDLSRRIELDGPEDELKE LSDTFDEMLDRLERAFTAQQRFVANASHELRTPLAINRTLLEVTLSDPGAPPELHQLGKT LLATNERSEQLVEGLLLLARSDNEIVDRKPVDLAEVASRALDQTRSEAQGKGVELRGTRQ PAVVQGNGVLLERIALNLVQNAVRYNTPDGWVEVTTEAQPGQAVLVVENTGPVVPAYELD NIFEPFRRLRTERTGSDKGVGLGLSIVRSVARAHGGRITAEPREGGGLIMRVVLPT  
>SRIM_RS30980 Streptomyces_rimosus_subsp.rimosus_ATCC_10970.gbff Streptomyces rimosus subsp. recipro:1 MPTSSRTPTPPTTPPKPTWDPKPVNVRPFPWLRPTIRIRLTLLYGGMFLMAGIVLLTIIY MLAANALQEGSELPVKILRGNFAATSNVCNLPNESSGEIFTQAIQSCLQHQRAQALNTLL NRSLLALLGLTVVAFAFGYAMAGRVLSPLGRITRTAQRVAGSDLHRRIELGGPDDELKEL ADTFDEMLDRLDRAFESQRRFVANASHELRTPLAINRTLLEVQLADPDASPELAQLGKTL LATNERSEQLVEGLLLLARSENKIVDKKPVDLAEVASQALDQARAEAQAKGVEIRGVRQQ AFVQGNGVLLERIALNLVQNAVRYNIKDEGWVEVTTEPQQGCAVLVVSNTGPAVPAYEVE NLFEPFRRLRTERTGSDKGVGLGLSIVRSVVRAHDGTITATPREGGGLEMRVVLPV  
>P7W03_RS26950 Streptomyces_rochei_strain_JK1.gbff Streptomyces rochei recipro:1 VATTPAPPGVPPKPTWDPRSASPLPWLRPTIRIRLTLLYGGMFLIAGILLLSIIYLLAAQ AVRTGNQPLYKIVDFEDLQVASTDCPGVTNGNLSLSQFNAAISDCIDHQRQVALDRLLSR SLLALLGLAVIAFAFGYAMAGRVLSPLGRITRTARAVAGSDLSRRIELDGPDDELKELAD TFDDMLERLQRAFTAQQRFVGNASHELRTPLAINRTLLEVHLSDPHAPVELQQLGKTLLA TNERSEQLVEGLLLLARSDNQIVERKPVDLAEVAGQAIDQVHAEADTKGVAIRGTREPAV VQGNGVLLERIALNLVQNAVRYNVAEQGWVEVTTSVENGQAVLVVTNTGPVVPAYEIDNL FEPFRRLRTERTGSDKGVGLGLSIARSVARAHGGHIYAQPREGGGLVMRVTLPV  
>FQU76_RS26645 Streptomyces_qinzhouensis_strain_SSL-25.gbff Streptomyces qinzhouensis recipro:1 VSAAPAPPKVPPKPSWDPRIPVRPLLRPTIRIRLTLLYGGMFLIAGILLLSIIYLLAAQA LHEGSTLPFKIISGKFQETMSGCNLPADGTNEEFNTALAQCFAHQRELALNDLLRRSLFA LLGLSIIAFAFGFAMAGRVLSPLGRITRTARQVAGSDLSRRIQLDGPEDELKELADTFDE MLARLERAFTAQQRFVANASHELRTPLAINRTLLEVHLSDPGAPVELQQLGKTLLATNER SEQLVEGLLLLARSENQIVDHKPVDLAEVAARAVDQTRGEAAERGVEIRGEREPAVVPGN GVLLERVALNLVQNAVRYNVPDGGWVEVTTGTGPGRAELVVENTGPVVPAYEIDNIFEPF RRLRQERTGSDKGVGLGLSIVRSVVRAHGGRIIAEPREGGGLVMRVTLPL  
>F0345_RS22855 Streptomyces_rutgersensis_strain_NBH77.gbff Streptomyces rutgersensis recipro:1 VAASPAPPPAPPRTPPAPPRPTWDPRRPPQQAWLRPTIRIRLTLLYGGMFLIAGILLLSI IYLLAAQAVHVSNELPFQVTKGTFTIYSDACPALSGRRFTSSGAFNDAMKLCIDHQRQQA LDDLLSRSLLALVGLSVIAFAFGYAMAGRVLAPLGRITRTARSVAGSDLSRRIELDGPDD ELKELADTFDDMLDRLQRAFTAQQRFVANASHELRTPLAINRTLLEVQLSDPGAPPELQQ LGKTLLATNERSEQLVEGLLLLARSDNQIVERKPVDLGEVATRAVEQVRGEAEQRDVELR PRIAEAVVQGNGVLLERIALNLTQNAVRYNLPPGEGGWVEVSTELQHGQAVLVVTNTGPV VPAYETDQLFEPFRRLRTERTGSDKGVGLGLSIVRSVTRAHGGRITAEPREGGGLVMRVT LPL  
>OG949_RS08815 Streptomyces_scopuliridis_strain_NBC_01786.gbff Streptomyces scopuliridis recipro:1 VASTPAPPAAPPKPTWDPRRPDQSLPWVRPTIRIRLTLLYGGMFLIAGILLLSIIYLLAA QALHVGSELPFKIVTGKVSSDVCNFPDQAPPEEFNAAMNACVNHQRQQALDDLLSRSLFA LVGLSVIAFAFGYAMAGRVLSPLGRITRTARRVVGSDLSRRIELDGPDDELKELADTFDE MLERLERAFSAQQRFVANASHELRTPLAINRTLLEVHLSDPGAPVELQQLGKTLLATNER SEQLVEGLLLLARSDNQIVERKPVDLGEVATRAVDQALSEAEAKAVEIRGERVPAIVQGN GVLLERIALNLVQNAVRYNIPEGGWVEVTTAIEQGQAILVVSNTGPVVPAYEIDNLFEPF RRLRTERTGSDKGVGLGLSIARSVARAHGGRIHAEPREGGGLVMRVTLPV  
>SCAB_RS11380 Streptomyces_scabiei_87.22.gbff Streptomyces scabiei 87.22 recipro:1 VAPAPAPPQAPPKPTWDPRRAQNPLPWLRPTIRIRLTLLYGGMFLIAGILLLSIIYLLAA QAVSTGNTPVFKIEGGDNISVSSALCPTVDANTAPTNLKLDDFNAAISACIDDHRQKALD TLLSRSLLALLGLAVIAFAFGYAMAGRVLAPLGRITRTARAVAGSDLSRRIELDGPDDEL KELADTFDEMLDRLQRAFTAQQRFVGNASHELRTPLAINRTLLEVHLSDPGAPTELQQLG KTLLATNERSEQLVEGLLLLARSDNQIVERKPVDLAEVASRAIDQVRSEAEAKKVEIRGE WAPAVVQGNGVLLERIALNLVQNAVRYNVPGPGGWVEVTTELQHGQAVLVVTNTGPVVPA YEIDNLFEPFRRLRTERTGSDKGVGLGLSIVRSVARAHGGHIAARPREGGGLVMRVTLPV  
>IAG44_RS09910 Streptomyces_roseirectus_strain_CRXT-G-22.gbff Streptomyces roseirectus recipro:1 MPFPWLRPTIRIRLTLLYGGMFLIAGILLLSIIYLLAAQAINTGNEPPFKIVTGNSMRVT SSSCPAVNAATNADGEVYLSTFNAAISACVDERRQDSLDNLLSRSLLALLGLAVIAFAFG YAMAGRVLTPLGRITRTARAVAGSDLSRRIELDGPDDELKELADTLDDMLARLQRAFTAQ QRFVGNASHELRTPLAINRTLLEVHLSDPNAPVELQQLGKTLLATNERSEQLVEGLLLLA RSDNQIVERKPVDLAEVAGQAIDQAHGEAEAKGVTIRGERQSAVVQGNGVLLERIALNLV QNAVRYNVPEEGWVEVTTEVQHGQAVLVVSNTGPVVPAYEIDNLFEPFRRLRTERTGSDK GVGLGLSIVRSVARAHGGHISAQPREGGGLVMRVTLPL  
>I1A49_RS32535 Streptomyces_solisilvae_strain_HNM0141.gbff Streptomyces solisilvae recipro:1 MATTPTPSPPPPTVPPKPSWDPHAGSRPNPLLRPTIRIRLTLLYGGMFLIAGVVLLTIIY LLAAQALHVGNELPFKLVGGSVQPTNNTCPEIIGQSSPDQFNAVLNTCMKEQRQLALDGL LRRSLIALLGLSVIAFAFGYAMAGRVLSPLGRITRTARQVAGSDLSRRIELDGPDDELKE LADTFDEMLERLDRAFTAQQRFVANASHELRTPLAINRTLLEVQLSDPQASPELVQLGKT LLATNERSEQLVEGLLLLARSDNEIVDRKPVDLAEVASQAMEQVRAEAEDKGVELRGQRA PAVVQGNGVLLERIALNLVQNAVRYNVAEDGWVEVTTESRQGQAVLVVANTGPVVPAYEM DNIFEPFRRLRTERTGSDKGVGLGLSIARSVARAHGGRIAAEPREGGGLVMRVVLPV  
>LXH13_RS29530 Streptomyces_spinosirectus_strain_CRSS-Y-16.gbff Streptomyces spinosirectus recipro:1 MAATPALPTAPPKPTWDPRKPEPPFPWLRPTIRIRLTLLYGGMFLIAGILLLSIIYLLAA QALHEGSGQTFQLSGTNLDISSSTCPKLNQATSNDQFNSILASCEAVQRQHALDDLLSRS LLALLGLAVIAFAFGYAMAGRVLSPLGRILRTARAVAGSDLSRRIELDGPDDEIKELADT FDDMLERLQRAFTAQQRFVGNASHELRTPLAINRTLLEVHLSDPGAPVELQQLGKTLLAT NERSEQLVEGLLLLARSDNQIVERKPVDLAEVAEQAVDQVRAEADAKGVVIRGEQKPAIV QGNGVLLERIALNLVQNAVRYNVPDKGWVEVTTDVQHGQAVLVVTNTGPVVPAYEIDNLF EPFRRLRTERTGSDKGVGLGLSIVRSVARAHGGHISAQPREGGGLVMRVALPI  
>D0Z67_RS22315 Streptomyces_seoulensis_strain_KCTC_9819.gbff Streptomyces seoulensis recipro:1 VAATPAPPQTPPKPTWDPRPQPPFPWLRPTIRIRLTLLYGGMFLIAGILLLSIIYLLAAQ AISTGNQPLFKIVGYQDLRVSSSNCPAINNTTSLSLQDFNTAISQCVDHQRQVALDNLLS RSLLALLGLAVIAFAFGYAMAGRVLSPLGRITRTARQVAGSDLSRRIELDGPDDELKELA DTFDDMLERLQRAFTAQQRFVGNASHELRTPLAINRTLLEVHLSDPDAPVELQQLGKTLL ATNERSEQLVEGLLLLARSDNQIVERGPVDLAEVAGQAIDQVRGEAEARSVEIRSELEPA VVRGNGVLLERIALNLVQNAVRYNVAPDETQREGEAAESGWVEVATAVEHGRAVLTVVNT GPVVPAYEIDNLFEPFRRLRTERTGSDKGVGLGLSIVRSVARAHGGHIAAVPREGGGLVM RVTLPL  
>CP982_RS30995 Streptomyces_spectabilis_strain_ATCC_27465.gbff Streptomyces spectabilis recipro:1 VAATPAPPAAPPKPTWDPRKVEPPFPWLRPTIRIRLTLLYGGMFLIAGILLLSIIYLLAA QALNVGSELPFKIVSGRVESKVCDLPSRASPDEFNAAMNACVNEQRQHALDNLLSRSLLA LLGLAVIAFAFGYAMAGRVLSPLGRITRTARRVAGTDLKRRIELDGPDDELKELSDTFDE MLDRLERAFTAQQRFVANASHELRTPLAINRTLLEVQLSDPHAPPELQQLGKTLLATNER SEQLVEGLLLLARSDNQIVERKPVDLAEVANRAVDQARAEAEEKGVEIRGERGLAIVQGN GVLLERIALNLVQNAVRYNVKEAGWVEVTTEAQHGQAVLVVANTGPVVPAYEIDNLFEPF RRLRTERTGSDKGVGLGLSIARSVARAHGGRIIAEPREGGGLVMRVTLPI  
>CP968_RS08665 Streptomyces_subrutilus_strain_ATCC_27467.gbff Streptomyces subrutilus recipro:1 VAASPAPPSAPPKPTWDPGQPEGPFPWLRPTIRIRLTLLYGGMFLIAGILLLSIIYLLAA QALREGNALPFKIVSGVEVRVTSSTCPGVGGEGQPLDQFNAAINACVLEQRRHALDDLLS RSLMALLGLSIIAFAFGYAMAGRVLSPLGKITRTARRVVGSDLTRRIELDGPDDELKELA DTFDEMLDRLERAFTAQQRFVANASHELRTPLAINRTLLEVHLSDPGAPLELQQLGKTLL ATNERSEQLVEGLLLLARSDNQIVERKPVDLAEVASRALDQARGEAEAKGVEIGGERALV VVQGNGVLLERIALNLVQNAVRYNVPEGGWIEVTTEAQHGQAVLLVSNTGPVVPAYEVDN LFEPFRRLRTERTGSDKGVGLGLSIARSVARAHGGRIQATPREGGGLVMRVTLPL  
>DDQ41_RS24655 Streptomyces_spongiicola_strain_HNM0071.gbff Streptomyces spongiicola recipro:1  
>DDQ41_RS24655 Streptomyces_spongiicola_strain_HNM0071.gbff Streptomyces spongiicola recipro:1 VATAPAPPTAPPKPTWDPREPGGPLLRPTIRIRLTLLYGGMFLIAGILLLSIIYLLAAQA LHDGIRQSVEVGAAPGANVTITSPTCPRINDLVDNSERNAALKLCIAEQRQRALDELLTR SLFALLGLSVIAFAFGYAMAGRVLSPLGKITRTARRVVGTDLSRRIELDGPDDELKELAD TFDEMLERLERAFSAQQRFVANASHELRTPLAINRTLLEVHLSDPGAPAELRQLGKTLLA TNERSEQLVEGLLLLARSENQIVERKPVDLAEVAGRAVDQTRAEAEAGGVEIRGARAPAV VQGNGVLLERIALNLVQNAVRYNIADGGWVEVTTGVEHGLAVLVVANTGPVVPGYEIDNL FEPFRRLRQERTGSDRGVGLGLSIARSVARAHGGRIIAEPREGGGLVMRVTLAL  
>OG288_RS12590 Streptomyces_tauricus_strain_NBC_00189.gbff Streptomyces tauricus recipro:1 MTTTPAPPKAPPKPTWDPRKPEPPFPWLRPTIRIRLTLLYGGMFLIAGILLLSIIYLLAA QALHEGSGVDFRVTGSNLELTSNTCPQLSGAGNNDELNAMLKECTAVQRQHALDGLLSRS LMALLGLAVIAFAFGYAMAGRVLSPLGRITRTARAVAGSDLSRRIELDGPDDELKELADT FDDMLERLQRAFTAQQRFVGNASHELRTPLAINRTLLEVHLSDPGAPMELQQLGKTLLAT NERSEQLVEGLLLLARSDNQIVERKPVDLAEVASQAIDQVRGEADVKGVEVRGERAAAVV QGNGVLLERIALNLVQNAVRYNVAQDGWVEVVTHVQHGQAVLTVSNTGPVVPAYEIDNLF EPFRRLRTERTGSDKGVGLGLSIARSVARAHGGHIVAEPREGGGLVMRVALPI  
>LDH80_RS11000 Streptomyces_tanashiensis_strain_Kala.gbff Streptomyces tanashiensis recipro:1 MATTPAPHPQAPPKPTWDPRDPVRPLLRPTIRIRLTLLYGGMFLIAGILLLSIIYLFTAQ ALSDSTSQLPFKVVNGTVQPTTSWCRLPESPTGDQLNDAVTLCLRHQSDLALDDLLRRSL FALLGLSIIAFAFGYAMAGRVLSPLGRITRTARQVAGSDLSRRIELDGPDDELKELADTF DEMLDRLERAFTAQQRFVANASHELRTPLAINRTLLEVHLSDPGAPVELQQLGKTLLATN ERSEQLVEGLLLLARSDNQIIERKPVDLAEVAERGVDQVHAEAVAKGVEIRRELDPAVVQ GNGVLLERIALNLLQNAVRYNVPEDGWVEVTTETKDGQAVLVVSNTGPVVPAYEIDNLFE PFRRLRQERTGSDKGVGLGLSIARSVARAHGGRIIAEPREGGGLVMRVTLPI  
>B7R87_RS26840 Streptomyces_tsukubensis_strain_NRRL_18488.gbff Streptomyces tsukubensis recipro:1 VPAAPAPPKVPPKPSWDPRTPVRPLLRPTIRIRLTLLYGGMFLIAGILLLSIIYLLAAQA LHDGSNSNLPFKIISGKFQETTSGCNLPADGTSEEFTSALTQCFAHQREVALNDLLRRSL FALLGLSIIAFAFGYAMAGRVLSPLGRITRTARQVAGSDLSRRIKLDGPEDELKELADTF DEMLARLERAFTAQQRFVANASHELRTPLAINRTLLEVHLSDPGAPVELQQLGKTLLATN ERSEQLVEGLLLLARSENQIVEHKPVDLAEVATRAVDQTRGEAAERGVEIRGEREPAVVS GNGVLLERVALNLVQNAVRYNVPDGGWVEVTTRTGTGRAELVVENTGPVVPAYETDNIFE PFRRLRQERTGSDKGVGLGLSIVRSVVRAHGGRIIAEPREGGGLVMRVTLPL  
>DDW44_RS23595 Streptomyces_tirandamycinicus_strain_HNM0039.gbff Streptomyces tirandamycinicus recipro:1 VATAPAPPTAPPKPTWDPREPVGPLLRPTIRIRLTLLYGGMFLIAGILLLSIIYLLAAQA LHDGIRQSVEVGAAPGANVTITSPTCPRINDLVDNGQRNAALKLCIAEQRQRALDELLTR SLFALLGLSVIAFAFGYAMAGRVLSPLGKITRTARRVVGTDLSRRIELDGPDDELKELAD TFDEMLERLERAFSAQQRFVANASHELRTPLAINRTLLEVHLSDPGAPAELRQLGKTLLA TNERSEQLVEGLLLLARSENQIVERKPVDLAEVAGRAVDQTRAEAEAGGVEIRGERAPAV VQGSGVLLERIALNLVQNAVRYNIADGGWVEVTTKAEHGQAVLVVANTGPVVPGYEIDNL FEPFRRLRQERTGSDRGVGLGLSIARSVARAHGGRIIAEPREGGGLVMRVTLPL  
>IGS69_RS26470 Streptomyces_tuirus_strain_JCM_4255.gbff Streptomyces tuirus recipro:1 VAATPAPPQAPPKPTWDPRRPVPPFPWLRPTIRIRLTLLYGGMFLIAGILLLSIIYLLAA QALNVGSDLPFKIVEGKVTSDICNLPDQASPSEFNNAMNACVNEQRRHALDSLLSRSLLA LLGLAVIAFAFGYAMAGRVLSPLGRITRTARAVAGSDLSRRIELDGPDDELKELADTFDD MLERLQRAFTAQQRFVGNASHELRTPLAINRTLLEVHLSDPKAPMELQQLGKTLLATNER SEQLVEGLLLLARSDNQIVERGPVDLAEVAEQAVDQVHGEAESKGVVIRGEQKPAVVQGN GVLLERIALNLVQNAVRYNITEDGWVEVTTDVQHGQAVLVVSNTGPVVPAYEIDNLFEPF RRLRTERTGSDKGVGLGLSIVRSVARAHGGHIYAQPREGGGLVMRVTLPI  
>MMF93_RS25415 Streptomyces_tubbatahanensis_strain_DSD3025.gbff Streptomyces tubbatahanensis recipro:1  
>MMF93_RS25415 Streptomyces_tubbatahanensis_strain_DSD3025.gbff Streptomyces tubbatahanensis recipro:1 MATSPTPTGSDWGGGPSAPRTPPKPNWDPRETPRPSPWLRPTIRIRLTLLYGGMFLIAGM LLLAIIYLLAAQAMDNLSELPFRILSGKTQPTSDRCPGLTGTLPSDAFMDRLSACLDAQR RIALDGLLRRSLLALLGLAVAAFAFGYVMAGRVLSPLGRITRTARQVAGSDLHKRIELEG PDDELKELADTFDEMLDRLNRAFTAQQRFVANASHELRTPLAINRTLLEVQLADPEGSPE LQQLAKTLLATNERSEQLVEGLLLLARSENELVDRKPVDLAEVASQAVEQARGEADTKGV ELRGVRQPTYVQGNGVLLERVALNLVQNAVRYNLRDGGWVSVGTEAQPGWAVLVVENTGP VVPAYEVDNLFEPFRRLRTERTGSDKGVGLGLSIVRSVARAHGGTVSAVPREEGGLVMRV TLPL  
>vnz_RS27635 Streptomyces_venezuelae_strain_NRRL_B-65442.gbff Streptomyces venezuelae recipro:1 MATTPAPHPQAPPKPTWDPRDPVRPLLRPTIRIRLTLLYGGMFLIAGILLLSIIYLFTAQ ALSDSTSQLPFKVVNGTVQPTSAICQLPVNPTGDQLNDAVTLCLRHQSDRALDDLLRRSL FALLGLSIIAFAFGYAMAGRVLSPLGRITRTARQVAGSDLSRRIELDGPDDELKELADTF DEMLDRLERAFTAQQRFVANASHELRTPLAINRTLLEVHLSDPGAPVELQQLGKTLLATN ERSEQLVEGLLLLARSDNQIIERKPVDLAEVAERGVDQVHAEAESRGVEIRGERAPAVVQ GNGVLLERITLNLLQNAVRYNVPEGGWVEVTTETQHGQAVLVVSNTGPVVPAYEIDNIFE PFRRLRQERTGSDKGVGLGLSIARSVARAHGGRIIAEPREGGGLVMRVTLPI  
>FDM97_RS25645 Streptomyces_vilmorinianum_strain_YP1.gbff Streptomyces vilmorinianum recipro:1 VAATPAPPQAPPKPTWDPRDPVRPLLRPTIRIRLTLLYGGMFLIAGILLLSIIYLFTAQV LNVSVSELPFKVVTGQVQPTTSWCRLPESGTGEQLNEAVSACLQHQRDLALDDLLRRSLF ALLGLSIIAFAFGYAMAGRVLSPLGRITRTARQVAGSDLARRIELDGPDDELKELADTFD EMLERLERAFTAQQRFVANASHELRTPLAINRTLLEVHLSDPGAPVELQQLGKTLLATNE RSEQLVEGLLLLARSDNQIVERKPVDLAEVASRAIDQVHGEAEAKGVEIRGERGPAVVQG NGVLLERIALNLVQNAVRYNVAEGGWVEVTTETQHGQALLVVSNTGPVVPAYEIDNLFEP FRRLRQERTGSDKGVGLGLSIARSVARAHGGRIIAEPREGGGLVMRVTLPI  
>SVTN_RS28325 Streptomyces_vietnamensis_strain_GIMV4.0001.gbff Streptomyces vietnamensis recipro:1 MATTPAPHPQAPPKPTWDPRDPVRPLLRPTIRIRLTLLYGGMFLIAGILLLSIIYLFTAQ ALSDSTSQLPFKVVNGTVQPTTSWCTLPESPTGDQLNNAVTLCLRHQSDIALEDLLRRSL FALLGLSIIAFAFGYAMAGRVLSPLGRITRTARQVASSDLSRRIELDGPDDELKELADTF DDMLDRLERAFTAQQRFVANASHELRTPLAINRTLLEVHLSDPGAPVELHQLGKTLLATN ERSEQLVEGLLLLARSDNEIIERKPVDLAEVAERGVDQVHAEAEAKGVEIRGERESAVVQ GNGVLLERIVLNLLQNAVRYNVPEGGWVEVTTEAKDGQAVLVVSNTGPVVPAYEIDNLFE PFRRLRQERTGSDKGVGLGLSIARSVARAHGGRIIAEPREGGGLVMRVTLPI  
>OG711_RS10260 Streptomyces_uncialis_strain_NBC_01365.gbff Streptomyces uncialis recipro:1 MAATPPPPTAPPKPTWDPARPEPPFPWLRPTIRIRLTLLYGGMFLIAGILLLSIIYLLAA QAIGVGNKMPFTIVSGELQVSSQSCPDLADTPRTQDAINDALAQCLNHRRQQALDELLSR SLMALLGLSVIAFAFGYAMAGRVLSPLGRITRTARQVAGSDLTRRIELDGPDDELKELAD TFDDMLERLQRAFTAQQRFVGNASHELRTPLAINRTLLEVHLSDPEAPPELHQLGKTLLA TNERSEQLVEGLLLLARSDNQIVERKPVDLAEVASRAIDQAHGEAQQKGVEIRGERAPAV VQGNGVLLERIALNLVQNAVRYNVPEDGWVEVTTEVQHGQAVLVVSNTGPVVPAYEIDNL FEPFRRLRTERTNSDKGVGLGLSIARSVARAHRGRIIAEPREGGGLVMRVTLPI  
>R2E43_RS09305 Streptomyces_violaceoruber_strain_CGMCC_4.1801.gbff Streptomyces violaceoruber recipro:1 VATTPAPPGAPPKPTWDPRSATPLPWLRPTIRIRLTLLYGGMFLIAGILLLSIIYLLAAQ AVRTGNEPLYKIVDFTDLKVSSSTCPVVDNGGLSLSDFNAAISDCMDHQRKVALDNLLSR SLLALLGLAVIAFAFGYAMAGRVLSPLGRITRTARAVAGSDLSRRIELDGPDDELKELAD TFDDMLERLQRAFTAQQRFVGNASHELRTPLAINRTLLEVHLSDPGAPVELQQLGKTLLA TNERSELLVEGLLLLARSDNQIVERKPVDLAEVAGQAIDQVHAEAESKGVEVRGTREAAV VQGNGVLLERIALNLVQNAVRYNVAGQGWVEVATAVENGQAVLVVTNTGPVVPAYEVDNL FEPFRRLRTERTGSDKGVGLGLSIARSVARAHGGHISAQPREGGGLVMRVTLPV  
>CP969_RS28190 Streptomyces_viridosporus_T7A_strain_ATCC_39115.gbff Streptomyces viridosporus recipro:1 MAATPAPPTAPPKPTWDPRKPEPPFPWLRPTIRIRLTLLYGGMFLIAGILLLSIIYLLAA QALHDGSGQSFKVIGTNITITSETCPQLQGATDNSQLNEMLKQCNAVQRQHALDDLLSRS LGALLGLAVIAFAFGYAMAGRVLSPLGRITRTARAVAGSDLSRRIELDGPDDELKELADT FDDMLERLERAFTAQQRFVGNASHELRTPLAINRTLLEVHLSDPTAPVELQQLGKTLLAT NERSEQLVEGLLLLARSDNQIVERKAVDLAEVASQAIDQVHAEAEATGVEIRSSSEPAVV QGNGVLLERVALNLVQNAVRYNVPEGGWVEVTTRVQHGQAVLEVSNTGPVVPAYEIDNLF EPFRRLRTERTGSDKGVGLGLSIVRSVARAHGGHVSARPREGGGLVMRFTLPV  
>OG542_RS10060 Streptomyces_violaceus_strain_NBC_00450.gbff Streptomyces violaceus recipro:1 VAATPAPPQAPPKPTWDPRRPAPPFPWLRPTIRIRLTLLYGGMFLIAGILLLSIIYLLAA EALNVGSELPFKIVEGKVTSDICNLPSQASPAEFNHAMNQCVNDQRKAALDNLLSRSLLA LLGLAVIAFAFGYAMAGRVLSPLGRITRTARAVAGSDLSRRIELDGPDDELKELADTFDD MLERLQRAFTAQQRFVGNASHELRTPLAINRTLLEVHLSDPGAPMELQQLGKTLLATNER SEQLVEGLLLLARSDNQIVERGPVDLAEVASQAIDQVHGEAEDKGVRIRGEKKPAVVQGN GVLLERIALNLVQNAVRYNVAEDGWVEVTTEIQHGQALLVVSNTGPVVPAYEIDNLFEPF RRLRTERTGSDKGVGLGLSIVRSVARAHGGHIYAQPREGGGLVMRVTLPI  
>SXIN_RS07295 Streptomyces_xinghaiensis_S187.gbff Streptomyces xinghaiensis recipro:1 VAGPPAPPAAPPKPTWDPQTAPGGPRPWLRPTIRIRLTLLYGGMFLIAGIVLLSIIYLLA AEALDKGNALPFKLLEGSFRPTSSTCPNLTDRTFENHEEFNAALEVCMDYQRQLALDNLL RRSLLALLGLSVAAFAFGYAMAGRVLSPLGRITRTARRVVGSDLSRRIELDGPDDELKEL ADTFDEMLNRLDRAFDAQRRFVANASHELRTPLAINRTLLEVQLSDPEAGPELTQLGKTL LATNERSEQLVEGLLLLARSENEIVDRKPVDLAEVASQAVEQVREEARGKGVELRGERRL AVVLGNGVLLERVALNLLQNAVRYNVPDGWVEISTGQWEGGASLVVSNTGPVVPAYELEN IFEPFRRLRSERTGSDKGVGLGLSIVRSVVRAHGGHVSAEPREGGGLVMRVTLPV  
>CP980_RS08265 Streptomyces_vinaceus_strain_ATCC_27476.gbff Streptomyces vinaceus recipro:1 VAATPAPPTVPPRPTWDPGQPEGPFPWLRPTIRIRLTLLYGGMFLIAGILLLSIIYLLAA QALREGNGLPFKIVGGTDIQVTSTCPGVVGKGQKYEQFNDVINTCILEQRRHALDDLLSR SLMALLGLSIIAFAFGYAMAGRVLSPLGKITRTARRVVGSDLTRRIELDGPEDELKELAD TFDEMLDRLERAFTAQQRFVANASHELRTPLAINRTLLEVHLSDPGAPVELQQLGKTLLA TNERSEQLVEGLLLLARSDNQIVERKPVDLAEVASRAIDQARGEAATKGVEIRGERAPAV VQGNGVLLERIALNLVQNAVRYNVPEGGWVEVTTESQHGQAILVVSNTGPVVPAYEVDNL FEPFRRLRTERTGSDKGVGLGLSIARSVARAHGGRIAATPREGGGLVMRVTLPL  
>KPP03845_RS27990 Streptomyces_xanthophaeus_strain_KPP03845.gbff Streptomyces xanthophaeus recipro:1 MAAIPAPPAAPPKPNWDPGQPEGPFPWLRPTIRIRLTLLYGGMFLIAGILLLSIIYLLAA QALREGNALPFTIVSGGPIQVTSTTCPGLANVNGNGMLTSEAFQSAISQCALDQRRHALD DLLSRSLMALLGLSIIAFAFGYAMAGRVLSPLGKITRTARRVVGSDLTRRIELDGPDDEL KELADTFDEMLDRLERAFTAQQRFVANASHELRTPLAINRTLLEVHLSDPGAPVELQQLG KTLLATNERSEQLVEGLLLLARSENQIVERKPVDLAEVASRAIEQARGEAETKGVEIRGE RALAVVQGNGVLLERIALNLVQNAVRYNVPDGGWVEVTTEVQHGQAVLLVSNTGPVVPAY EVDNLFEPFRRLRTERTGSDKGVGLGLSIARSVARAHGGRIQATPREGGGLVMRVTLPL  
>SXIM_RS22710 Streptomyces_xiamenensis_strain_MCCC_1A01550.gbff Streptomyces xiamenensis recipro:1 MAAASSPSSPPPPPPPSQPPHASGRPAPRRPAAPPRPDFDPPEADPQRSWLRPTIRIRLT LLYGGMFLFAGMLLLTIIYLLAAEALHKGNALPFLITSDTRVQITSDSCQGISGNVTSDQ FKDWLSGCVDIQRDVALKALLRRSLMALLGLAVAAFAFGYVMAGRVLSPLGRITRTARQV AGSDLHRRIELDGPDDELKELADTFDEMLDRLDRAFTAQQRFVANASHELRTPLAINRTL LEVQLSDPQASPEVVQLGNTLLATNRRSEQLVEGLLLLARSDNQIVERKPVDLAEVAGQA LEQTRGEAQAKGVELSGVRPPVYVQGNGVLLERVALNLLQNAVRHNLAEGGWVRVDTEAR PGQAVLIVENSGPMVPAYEVDNMFEPFRRLGKDRTGSDKGVGLGLSIVRSVARAHGGHVV AVPRESGGLVMRVTLPV  
>IAG42_RS08425 Streptomyces_xanthii_strain-CRXT-Y-14.gbff Streptomyces xanthii recipro:1 MAATPAPPSAPPKPTWKPGAKDEPPFPWLRPTIRIRLTLLYGGMFLIAGILLLSIIYLLA AQALNVGSELPFKIVEGTVSSSVCNIPDGTKLPASELNSALNDCVAQQRNHALDVLLSRS LMALLGLAVIAFAFGYAMAGRVLSPLGRITRTARRVAGTDLSRRIELDGPDDELKELSDT FDEMLDRLERAFTAQQRFVGNASHELRTPLAINRTLLEVHLSDPGAPVELQQLGKTLLAT NERSEQLVEGLLLLARSDNQIVERKPVDLAEVASQAIDQARAEADEKGVEIRGERKEAVV QGNGVLLERIALNLVQNAIRYNVREGGWVQVDTEAQHGQAVLVVANTGPVVPAYEIDNLF EPFRRLRTERTGSDKGVGLGLSIARSVARAHGGRITATPREVGGLVMRVTLPI  
>J8403_RS12780 Streptomyces_yatensis_strain_DSM_41771.gbff Streptomyces yatensis recipro:1 MATTPTPLPPTAPPKPSWDPQGASRPNPWLRPTIRIRLTLLYGGMFLIAGVVLLTIIYLL AAQALHVGNELPFKLVGGSVQPTNNTCPEIIGQSSPDQFNAVLNTCMKEQRQLALDGLLR RSLIALLGLSVIAFAFGYAMAGRVLSPLGRITRTARQVAGSDLSRRIELDGPDDELKELA DTFDEMLERLDRAFTAQQRFVANASHELRTPLAINRTLLEVQLSDPQASPELVQLGKTLL ATNERSEQLVEGLLLLARSDNEIVDRKPVDLAEVASQALEQVRAEAEGKGVELRGQRAPA VVQGNGVLLERIALNLVQNAVRYNIAEDGWVEVTTESRPGQAVLVVANTGPVVPAYEMDN IFEPFRRLRTERTGSDKGVGLGLSIARSVARAHGGRIAAEPREGGGLVMRVVLPV  
>MOV08_RS12175 Streptomyces_yunnanensis_strain_P86.gbff Streptomyces yunnanensis recipro:1 MPSLPSFSSSKSAPPPPPVPPKPAWDPKPVNVRPFPWLRPTIRIRLTLLYGGMFLMAGIV LLTIIYMLAAAALHDGSALPLKILGGKFQSTSDICDLPTETSGALLQEAVNNCLMHQRAV ALNNLLNRSLLALLGLTIVAFAFGYAMAGRVLSPLGRITRTAQRVAGSDLHRRIELGGPD DELKELADTFDEMLDRLDRAFESQRRFVSNASHELRTPLAINRTLLEVQLADPQASPEVQ QLGKTLLATNERSEQLVEGLLLLARSENKIVDKRPVDLSEVASQAVDQTREEAQAKGVQL RGVRQQVFVQGNGVLLERIALNLVQNAVRYNVPEGGWVEVATEPQPGCAVLVVSNTGPVV PAYEVENLFEPFRRLRTERTGSDKGVGLGLSIVRSVVRAHDGTITAEPREGGGLDMRVVL PL  
>NRK68_RS25920 Streptomyces_yangpuensis_strain_CM253.gbff Streptomyces yangpuensis recipro:1 VATTPAPPTAPPKPTWDPGQPEGPFPWLRPTIRIRLTLLYGGMFLIAGILLLSIIYLLAA QALRQGNALPFQIVGGQVKVTSSSCPGVVGLELTPDQFNAAIGQCILEQRRHALDDLLSR SLMALLGLSIIAFAFGYAMAGRVLSPLGKITRTARRVVGSDLTRRIELDGPDDELKELAD TFDEMLDRLERAFTAQQRFVANASHELRTPLAINRTLLEVHLSDPGAPVELQQLGKTLLA TNERSEQLVEGLLLLARSENQIVERKPVDLAEVASRAVDQVRGEAEAKGVEIRGERAPAV VQGNGVLLERIALNLVQNAVRYNVPEGGWVEVTTEAQHGQAVLLVSNTGPVVPAYEVDNL FEPFRRLRTERTGSDKGVGLGLSIARSVARAHGGRIQAMPREGGGLVMRVTLPL
