# Supplementary material for: The conserved two-component systems CutRS and CssRS control the protein secretion stress response in Streptomyces
Source: mBio. 2025 Dec 15;17(1):e02991-25. doi: 10.1128/mbio.02991-25 (PMC12802291; doi:10.1128/mbio.02991-25)
Supplement: Table S1 — Bacterial strains and plasmids used in this study. [file mbio.02991-25-s0006.docx]

**Supplementary Table 1.** Bacterial strains and plasmids used in this study.

| **Bacterial strain** **or Plasmid** | **Description** **and/or Resistance** | **Supplier and/or reference** |
| --- | --- | --- |
| *E. coli* DH5alpha | *E. coli* strain used for cloning | ThermoFisher |
| *E. coli* Top10 | F– mcrA Δ(mrr-hsdRMS-mcrBC) Φ80lacZΔM15 ΔlacX74 recA1 a | Invitrogen |
| *E. coli* ET12567/pUZ8002 | A methylation deficient (∆dcm∆dam) strain of E. coli containing the driver plasmid pUZ8002 for conjugation to Streptomyces species. | John Innes Centre |
| *E. coli* BL21 | *fhuA2 [lon] ompT gal (λ DE3) [dcm]* ∆*hsdSλ DE3 = λ sBamHIo* ∆*EcoRI-B int::(lacI::PlacUV5::T7 gene1) i21* ∆*nin5* | Studier and Moffatt (1986) |
| ECO001 | *E. coli* BL21 6XHIS *vnz_cutR. Containing; pTCM015, pLysS. KanR, CmlR* | This work |
| *ET* pTCM002 | *E. coli* ET12567/pUZ8002 containing pTCM002 for conjugation | This work |
| ET pTCM008 | *E. coli* ET12567/pUZ8002 containing pTCM008 for conjugation | This work |
| ET pAB007 | *E. coli* ET12567/pUZ8002 containing pAB007 for conjugation | This work |
| ET pAB012 | *E. coli* ET12567/pUZ8002 containing pAB012 for conjugation | This work |
| ET pTCM018 | *E. coli* ET12567/pUZ8002 containing pTCM018 for conjugation | This work |
| ET pTCM019 | *E. coli* ET12567/pUZ8002 containing pTCM019 for conjugation | This work |
| ET pTCM020 | *E. coli* ET12567/pUZ8002 containing pTCM020 for conjugation | This work |
| ET pTCM012 | *E. coli* ET12567/pUZ8002 containing pTCM012 for conjugation | This work |
| pTCM001 | *E. coli* ET12567/pUZ8002 containing pTCM001 for conjugation | This work |
| *S. venezuelae* ∆*cutRS  + SvCutRS FLAG CutR* | *S. venezuelae* ∆*cutRS* complemented in trans with the Sv*cutRS* operon encoding C-terminally 3xFlag tagged CutR under its native promoter | This work |
| *S. venezuelae* ∆*vnz_18430* | *S. venezuelae* NRRL B-65442 with an unmarked, in-frame deletion to remove the *htrA3* gene | This work |
| *S. venezuelae* ∆*vnz_18430 + pAB012* | *S. venezuelae* ∆*vnz_18430*  complemented in trans with the *htrB gene* under the ermE* promoter | This work |
| *S. venezuelae ∆cutRS +* *cutS* | *S. venezuelae* ∆*cutRS* complemented in trans with the *cutS* gene under the ermE* promoter | This work |
| *S. venezuelae ∆cutRS +* *cutR* | *S. venezuelae* ∆*cutRS* complemented in trans with the *cutR* gene under the ermE* promoter | This work |
| *S. venezuelae* NRRL B-65442 | *S. venezuelae* NRRL B-65442 | This work |
| *S. venezuelae* ∆*cutRS + pTCM001* | *S. venezuelae ∆cutRS complemented in trans with the cutRS operon under the ermE* promoter* | This work |
| *S. venezuela*e ∆*cssRS* | *S. venezuelae ∆cssRS* | This work |
| *S. venezuelae* ∆*cutRS* ∆*cssRS* | *S. venezuelae vnz_cutRS::apr oriT AprR* | This work |
| *S. venezuelae* M1700 | *S. venezuelae Δcml* | This work |
| *S. venezuelae* M1701 | *S. venezuelae Δjad* | This work |
| *S. venezuelae* M1702 | *S. venezuelae ΔcmlΔjad* | This work |
| pSS170 | *oriT*, ΦBT1 *attB-int*, *hyg^R^* ^\|^ Hyg^R^ | Gift from Susan Schlimpert, JIC. |
| pIJ10257 | *oriT*, ΦBT1 *attB-int,* *hyg^R^*, *ermEp** \| Hyg^R^ | Hong *et al.*, 2005 |
| pET28a | pBR322 origin and fI origin, *Km^R^*, expression vector \| KanR | Novagen |
| St1∆*cutRS* | Supercos-1-cosmid containing *vnz_cutRS::apr oriT* with flanking DNA \| KanR, AmpR, AprR | This work, MM Al-Bassam |
| pTCM002 | pIJ10257 *vnz_cutRS* \| HygR | This work |
| pTCM008 | pSS170 *vnz_cutRSp vnz_cutRS 3xFLAG* \| HygR | This work, Genewiz |
| pTCM015 | pET28a 6xHIS *vnz_cutR* \| KanR | This work |
| pAB007 | pSS170 with cutRS (CutS C85S, C103S) expressed under its native promoter  \| HygR | This work, Genscript |
| pAB012 | pIJ10257+htrB expressed under ermEp \| HygR | This work, Azenta |
| pTCM018 | pCRISPomyces-2 *vnz_cssRS* flanking DNA and gRNA \| AprR | This work |
| pTCM019 | pIJ10257 *vnz_cutR* \| HygR | This work |
| pTCM020 | pIJ10257 *vnz_cutS* \| HygR | This work |
| pTCM012 | pCRISPomyces-2 *vnz_18430* flanking DNA and gRNA \| AprR | This work |
| pTCM015 | pET28a 6xHIS *vnz_cutR* \| KanR | This work |
| pTCM001 | pSS170 *vnz_cutRSp vnz_cutRS* \| HygR | This work |
| pIJ12738 | pKC1132 with MCS and I-SceI site from pUC57-Simple_SceI \| AprR | Fernández-Martínez & Bibb 2014 |
| pIJ12742 | pGM1190 with ermE*p-I-SceI gene \| ThiR +C35:C44 | Fernández-Martínez & Bibb 2014 |
