# Supplementary material for: The conserved two-component systems CutRS and CssRS control the protein secretion stress response in Streptomyces
Source: mBio. 2025 Dec 15;17(1):e02991-25. doi: 10.1128/mbio.02991-25 (PMC12802291; doi:10.1128/mbio.02991-25)
Supplement: Table S2 — Oligonucleotide sequences used in this study. [file mbio.02991-25-s0007.docx]

**Supplementary Table 2.** Oligonucleotide sequences used in this study.

| **Primer** | **Primer sequence (5’−3’)** | **Function** |
| --- | --- | --- |
| pSS170_seqF | GGTCTGACGCTCAGTGGAAC | Forward primer to sequence pSS170 vector inserts |
| pSS170_seqR | GCTCAGTATCACCGCCAGTG | Reverse primer to sequence pSS170 vector inserts |
| pIJ10257_seqF | GATCTTGACGGCTGGCGAGAG | Forward primer to sequence pIJ10257 vector inserts |
| pIJ10257_seqR | GCGTCAGCATATCATCAGCGAGC | Reverse primer to sequence pIJ10257 vector inserts |
| pCRISP_F | AGGCTAGTCCGTTATCAACTTGAAA | Forward primer to sequence pCRISPomyces-2 editing template inserts |
| pCRISP_R | TCGCCACCTCTGACTTGAGCGTCGA | Reverse primer to sequence pCRISPomyces-2 editing template inserts |
| NH112ChlPCR1F | GATCAGAGATCTTGAGGTCGAGGACCGTCAGG | Forward primer for amplification of chloramphenicol cluster upstream homologous region |
| NH115ChlPCR2R | CTGTAGAAGCTTTCAATGCCTCCAGGAACGCACG | Reverse primer for amplification of chloramphenicol cluster upstream homologous region |
| NH116ChlPCR3F | CTGTCGAAGCTTGATGATGGACCGCGTGTAGC | Forward primer for amplification of chloramphenicol cluster downstream homologous region |
| NH119ChlPCR4R | GCTGACACTAGTAGCGATTCCGGTACGCGAGC | Revers primer for amplification of chloramphenicol cluster downstream homologous region |
| NH120JadPCR1F | GATCAGAGATCTCCACCGACCTGAGCGTCTCC | Forward primer for amplification of jadomycin cluster upstream homologous region |
| NH123JadPCR2R | GATCGTAAGCTTACACGCCGGTCGCCTGACACC | Reverse primer for amplification of jadomycin cluster upstream homologous region |
| NH124JadPCR3F | GTACAGAAGCTTTGGGCCGATCCTAGCATTGG | Forward primer for amplification of jadomycin cluster downstream homologous region |
| NH127JadPCR4R | ACCGATACTAGTGCGTGTAGTTGCGCGTCTGG | Revers primer for amplification of jadomycin cluster downstream homologous region |
| AB105 | GACGTGGCCGTACTCAA | Forward primer for amplification of vnz_18430 (htrA3) q-RT-PCR |
| AB106 | GTTCTTGGCGCTGATGATG | Reverse primer for amplification of vnz_18430 (htrA3) q-RT-PCR |
| AB117 | GCGCGCTGATCAATATGAAG | Forward primer for amplification of  vnz_19340 (htrB) q-RT-PCR |
| AB118 | AGCCGCTCGAAGAACTG | Reverse primer for amplification of  vnz_19340 (htrB) q-RT-PCR |
| AB133 | GAAGACCGTCGCCAAGAA | Forward primer for amplification of vnz_27210 (hrdB) q-RT-PCR |
| AB134 | CTCTTCTTCCTCGCCCTTG | Reverse primer for amplification of vnz_27210 (hrdB) q-RT-PCR |
| RLOTCSKO148 | ACGCGAGATGCGGGTCTACACCTA | cssRS pcrisp plasmid gRNA FWD |
| RLOTCSKO149 | AAACTAGGTGTAGACCCGCATCTC | cssRS pcrisp plasmid gRNA REV |
| RLOTCSKO150F | GCTCGGTTGCCGCCGGGCGTTTTTTAT CTAGAGTGAAGCTCTTCCCGTCGGCCC TGGTC | cssRS pcrisp plasmid arm1 FWD |
| RLOTCSKO151R | CAGCGCCGTACGCACGTCAGCTCAGCG TCGCACGAGGCCCGTTCGC | cssRS pcrisp plasmid arm1 REV |
| RLOTCSKO152F | GGGCCTCGTGCGACGCTGAGCTGACGT GCGTACGGCGCTGGCATGCGTA | cssRS pcrisp plasmid arm2 FWD |
| RLOTCSKO153R | GCGGCCTTTTTACGGTTCCTGGCCTCTA GACCCGCTGGAACTCCGACAACCTCAA C | cssRS pcrisp plasmid arm2 REV |
| TCM089 | gagagacataTGCGCGTACTCGTCGTCG | cutR FWD for insertion into pIJ10257 |
| TCM090 | ctctctaagcttTCAGATCCGGTACCCGGAGC | cutR REV for insertion into pIJ10257 |
| TCM087 | gagagacataTGGCCACCACCCCAGC | cutS FWD for insertion into pIJ10257 |
| TCM088 | ctctctaagcttTCAGATCGGCAGAGTGACGC | cutS REV for insertion into pIJ10257 |
| TCM027 | ctcggttgccgccgggcgttttttatctagaCCTGCTTGGGACGGTTGGT | vnz_18430 deletion F1B |
| TCM028 | CAGCCTAGCCGGTCAGCTGTCGCCGGTGCCGTTCTCGGTGCTCACAGCTC | vnz_18430 deletion R1B |
| TCM029 | ACGAGGAGAGCGAGCTGTGAGCACCGAGAACGGCACCGGCGACAGCTGAC | vnz_18430 deletion F2 |
| TCM030 | acgcggcctttttacggttcctggcctctagaCGGTAGCAGGAGGCCAGC | vnz_18430 deletion R2 |
| TCM031 | TAGACATCCTGGCCCTCCTA | vnz_18430 deletion Seq F |
| TCM032 | GCAGGTCGGAAGACTCAGTCG | vnz_18430 deletion Test F |
| TCM033 | GCCTCGACGGTTTTCAAGACC | vnz_18430 deletion Test R |
| TCM034 | acgcGTGCGCCTGAGAGTGGGGCT | vnz_18430 gRNA F2 |
| TCM035 | aaacAGCCCCACTCTCAGGCGCAC | vnz_18430 gRNA R2 |
| TCM259 | GCTGTGCGTTCGCACCCCCACCTTTATATACGGACAGAAC | ReDCaT vnz_18430p LH F |
| TCM260 | GTTCTGTCCGTATATAAAGGTGGGGGTGCGAACGCACAGCcctaccctacgtcctcctgc | ReDCaT vnz_18430p LH R |
| TCM261 | GCTGTGCGTTCGCACCCCCACCTTTATATACGGACAGA | ReDCaT vnz_18430p RH -2 truncation F |
| TCM262 | TCTGTCCGTATATAAAGGTGGGGGTGCGAACGCACAGCcctaccctacgtcctcctgc | ReDCaT vnz_18430p RH -2 truncation R |
| TCM263 | GCTGTGCGTTCGCACCCCCACCTTTATATACGGACA | ReDCaT vnz_18430p RH -4 truncation F |
| TCM264 | TGTCCGTATATAAAGGTGGGGGTGCGAACGCACAGCcctaccctacgtcctcctgc | ReDCaT vnz_18430p RH -4 truncation R |
| TCM265 | GCTGTGCGTTCGCACCCCCACCTTTATATACGGA | ReDCaT vnz_18430p RH -6 truncation F |
| TCM266 | TCCGTATATAAAGGTGGGGGTGCGAACGCACAGCcctaccctacgtcctcctgc | ReDCaT vnz_18430p RH -6 truncation R |
| TCM267 | GCTGTGCGTTCGCACCCCCACCTTTATATACG | ReDCaT vnz_18430p RH -8 truncation F |
| TCM268 | CGTATATAAAGGTGGGGGTGCGAACGCACAGCcctaccctacgtcctcctgc | ReDCaT vnz_18430p RH -8 truncation R |
| TCM269 | GCTGTGCGTTCGCACCCCCACCTTTATATA | ReDCaT vnz_18430p RH -10 truncation F |
| TCM270 | TATATAAAGGTGGGGGTGCGAACGCACAGCcctaccctacgtcctcctgc | ReDCaT vnz_18430p RH -10 truncation R |
| TCM271 | GCTGTGCGTTCGCACCCCCACCTTTATA | ReDCaT vnz_18430p RH -12 truncation F |
| TCM272 | TATAAAGGTGGGGGTGCGAACGCACAGCcctaccctacgtcctcctgc | ReDCaT vnz_18430p RH -12 truncation R |
| TCM273 | GCTGTGCGTTCGCACCCCCACCTTTA | ReDCaT vnz_18430p RH -14 truncation F |
| TCM274 | TAAAGGTGGGGGTGCGAACGCACAGCcctaccctacgtcctcctgc | ReDCaT vnz_18430p RH -14 truncation R |
| TCM275 | GCTGTGCGTTCGCACCCCCACCTT | ReDCaT vnz_18430p RH -16 truncation F |
| TCM276 | AAGGTGGGGGTGCGAACGCACAGCcctaccctacgtcctcctgc | ReDCaT vnz_18430p RH -16 truncation R |
| TCM107 | GTGCGTGTAAATAAAGTGGTGCGTGCGCGATATTTTTGCC | ReDCaT vnz_08815p F4 |
| TCM108 | GGCAAAAATATCGCGCACGCACCACTTTATTTACACGCACcctaccctacgtcctcctgc | ReDCaT vnz_08815p R4 |
| TCM109 | CGCGATATTTTTGCCAGTGGGATTTGAACTGATCACAACT | ReDCaT vnz_08815p F5 |
| TCM110 | AGTTGTGATCAGTTCAAATCCCACTGGCAAAAATATCGCGcctaccctacgtcctcctgc | ReDCaT vnz_08815p R5 |
| TCM111 | GAACTGATCACAACTTGGTCACTAGGGTCGGGCTTCGAAC | ReDCaT vnz_08815p F6 |
| TCM112 | GTTCGAAGCCCGACCCTAGTGACCAAGTTGTGATCAGTTCcctaccctacgtcctcctgc | ReDCaT vnz_08815p R6 |
| TCM113 | GGTCGGGCTTCGAACCTTCGCGCGGTTGATCACCCATCCG | ReDCaT vnz_08815p F7 |
| TCM114 | CGGATGGGTGATCAACCGCGCGAAGGTTCGAAGCCCGACCcctaccctacgtcctcctgc | ReDCaT vnz_08815p R7 |
| TCM115 | TTGATCACCCATCCGGGGTGGCGGCGGAGGAACCGCCTGC | ReDCaT vnz_08815p F8 |
| TCM116 | GCAGGCGGTTCCTCCGCCGCCACCCCGGATGGGTGATCAAcctaccctacgtcctcctgc | ReDCaT vnz_08815p R8 |
| TCM117 | GGAGGAACCGCCTGCCCCTCACCGGATGGGCGGCTCTGAG | ReDCaT vnz_08815p F9 |
| TCM118 | CTCAGAGCCGCCCATCCGGTGAGGGGCAGGCGGTTCCTCCcctaccctacgtcctcctgc | ReDCaT vnz_08815p R9 |
| TCM119 | CACCGGATGGGCGGCTCTGAGGAAGAAGGAGCTCGCCTTC | ReDCaT vnz_08815p F10 |
| TCM120 | GAAGGCGAGCTCCTTCTTCCTCAGAGCCGCCCATCCGGTGcctaccctacgtcctcctgc | ReDCaT vnz_08815p R10 |
