# Supplementary material for: The conserved two-component systems CutRS and CssRS control the protein secretion stress response in Streptomyces
Source: mBio. 2025 Dec 15;17(1):e02991-25. doi: 10.1128/mbio.02991-25 (PMC12802291; doi:10.1128/mbio.02991-25)
Supplement: Table S3 — Day 2 timepoint TMT-proteomics data. [file mbio.02991-25-s0008.docx]

**Supplementary Table 3.** The significant (Adj. P-value) changes between the *S. venezuelae* wild-type (WT) and ∆*cutRS* strains on YPD agar at the Day 2 timepoint detected by TMT-proteomics. The abundance of CutRS in the wild-type samples appears exaggerated due to the data processing against the ∆cutRS samples and does not represent normal levels.

| **Accession** | **Description** | **Abundance**  **WT vs. ∆*cutRS*** | **Adj. P-Value** |
| --- | --- | --- | --- |
| vnz_27390 | DNA-binding response regulator, CutR | 69.01 | 1.25545E-15 |
| vnz_27395 | sensor kinase, CutS | 32.421 | 1.25545E-15 |
| vnz_23735 | magnesium transporter | 20.676 | 1.25545E-15 |
| vnz_02410 | TetR family transcriptional regulator | 9.947 | 1.47189E-11 |
| vnz_18430 | protease | 8.134 | 1.25545E-15 |
| vnz_02160 | peptodoglycan binding protein | 7.28 | 1.25545E-15 |
| vnz_34200 | condensation protein | 6.477 | 2.1019E-09 |
| vnz_24800 | undecaprenyl-phosphate alpha-N-acetylglucosaminyl 1-phosphate transferase | 5.161 | 2.37302E-06 |
| vnz_31560 | acyl-ACP desaturase | 4.854 | 2.52687E-10 |
| vnz_17550 | pirin | 4.664 | 4.23235E-07 |
| vnz_25685 | 1-pyrroline-5-carboxylate dehydrogenase | 4.599 | 1.21261E-09 |
| vnz_37195 | hypothetical protein | 4.465 | 8.98647E-07 |
| vnz_25680 | proline dehydrogenase | 4.16 | 3.18617E-08 |
| vnz_10035 | antibiotic biosynthesis monooxygenase | 3.754 | 2.64839E-07 |
| vnz_12810 | hypothetical protein | 3.526 | 8.3386E-07 |
| vnz_13030 | PTS lactose transporter subunit IIC | 3.187 | 0.001246694 |
| vnz_31850 | peptidase | 3.072 | 2.3939E-05 |
| vnz_02150 | antibiotic biosynthesis monooxygenase | 3.071 | 2.3939E-05 |
| vnz_19120 | hypothetical protein | 2.901 | 0.003172425 |
| vnz_13070 | hypothetical protein | 2.851 | 0.000102979 |
| vnz_32830 | xylose isomerase | 2.849 | 0.000104178 |
| vnz_34985 | ABC transporter permease | 2.797 | 0.007009516 |
| vnz_23320 | malonic semialdehyde reductase | 2.694 | 0.000244318 |
| vnz_35755 | bifunctional 5,10-methylene-tetrahydrofolate dehydrogenase/5,10-methylene-tetrahydrofolate cyclohydrolase | 2.681 | 0.000316215 |
| vnz_32890 | hypothetical protein | 2.679 | 0.006364895 |
| vnz_04920 | hypothetical protein | 2.674 | 0.000381722 |
| vnz_03495 | hypothetical protein | 2.609 | 0.000536658 |
| vnz_08815 | peptodoglycan hydrolase | 2.56 | 0.000750973 |
| vnz_12585 | alcaligin biosynthesis protein | 2.557 | 0.000658529 |
| vnz_32835 | xylulose kinase | 2.507 | 0.002814659 |
| vnz_24765 | transcriptional regulator | 2.502 | 0.001136481 |
| vnz_06655 | bifunctional acetaldehyde-CoA/alcohol dehydrogenase | 2.497 | 0.001169868 |
| vnz_04645 | glycine dehydrogenase (aminomethyl-transferring) | 2.491 | 0.001206532 |
| vnz_08395 | hypothetical protein | 2.473 | 0.018783683 |
| vnz_03615 | serine or threonine protein kinase | 2.404 | 0.019591383 |
| vnz_28550 | Tellurite resistance TerB | 2.304 | 0.004468636 |
| vnz_04855 | 5-oxoprolinase | 2.279 | 0.004552847 |
| vnz_03490 | short-chain dehydrogenase | 2.254 | 0.006005857 |
| vnz_30475 | trans-2-enoyl-CoA reductase | 2.25 | 0.041624566 |
| vnz_28840 | hypothetical protein | 2.211 | 0.043257552 |
| vnz_23700 | RNA polymerase sigma factor SigE | 2.185 | 0.035808902 |
| vnz_26790 | AI-2E family transporter | 2.175 | 0.005467563 |
| vnz_15870 | peptidase | 2.161 | 0.031283781 |
| vnz_12595 | IucA/IucC family protein | 2.15 | 0.011705951 |
| vnz_01460 | L-threonine 3-dehydrogenase | 2.139 | 0.017628857 |
| vnz_12590 | GNAT family N-acetyltransferase | 2.124 | 0.014029337 |
| vnz_35865 | deacetylase | 2.118 | 0.046878736 |
| vnz_07070 | lytic transglycosylase | 2.1 | 0.046843675 |
| vnz_10060 | HAD family hydrolase | 2.031 | 0.024942267 |
| vnz_21320 | zinc metalloprotease HtpX | 2.004 | 0.026790074 |
| vnz_35645 | hydrolase | 1.985 | 0.032675705 |
| vnz_01455 | glycine C-acetyltransferase | 1.941 | 0.043531504 |
| vnz_28775 | ABC transporter substrate-binding protein | 0.498 | 0.048793715 |
| vnz_19575 | phosphate transport system regulatory protein PhoU | 0.497 | 0.048180263 |
| vnz_25285 | enoyl-CoA hydratase | 0.493 | 0.043257552 |
| vnz_09775 | ABC transporter substrate-binding protein | 0.491 | 0.041624566 |
| vnz_28945 | hydrolytic protein | 0.478 | 0.029432039 |
| vnz_00790 | pentalenene synthase | 0.467 | 0.022269036 |
| vnz_25720 | 3-isopropylmalate dehydrogenase | 0.463 | 0.01974971 |
| vnz_22735 | enoyl-CoA hydratase | 0.458 | 0.017121575 |
| vnz_36500 | aldo/keto reductase | 0.458 | 0.040308258 |
| vnz_24535 | tetratricopeptide repeat protein | 0.456 | 0.040308258 |
| vnz_05240 | 30S ribosomal protein S13 | 0.454 | 0.015282573 |
| vnz_36455 | DUF4916 domain-containing protein | 0.452 | 0.014319775 |
| vnz_18745 | aldo/keto reductase | 0.45 | 0.034279559 |
| vnz_18945 | DNA-binding protein | 0.45 | 0.013925335 |
| vnz_27130 | TerD-family protein | 0.448 | 0.031104162 |
| vnz_00495 | alpha/beta hydrolase | 0.447 | 0.012399445 |
| vnz_23210 | transglycosylase domain-containing protein | 0.447 | 0.031104162 |
| vnz_25455 | peptide ABC transporter substrate-binding protein | 0.441 | 0.01014234 |
| vnz_27570 | 3-hydroxyisobutyryl-CoA hydrolase | 0.441 | 0.030084405 |
| vnz_00785 | ABC transporter substrate-binding protein | 0.441 | 0.010251754 |
| vnz_21420 | adenosine deaminase | 0.44 | 0.030347681 |
| vnz_20600 | DUF4440 domain-containing protein | 0.438 | 0.009377445 |
| vnz_30015 | AMP-binding protein | 0.438 | 0.026560585 |
| vnz_03985 | aminopeptidase | 0.437 | 0.008941397 |
| vnz_23750 | magnesium transporter CorA | 0.437 | 0.014932656 |
| vnz_16120 | hypothetical protein | 0.437 | 0.025999539 |
| vnz_27560 | methylmalonate-semialdehyde dehydrogenase (acylating) | 0.434 | 0.008078771 |
| vnz_30400 | gamma-glutamyltransferase | 0.434 | 0.019591383 |
| vnz_04045 | glycoside hydrolase | 0.431 | 0.00745693 |
| vnz_19500 | hypothetical protein | 0.429 | 0.006925827 |
| vnz_34780 | iron ABC transporter substrate-binding protein | 0.423 | 0.005660039 |
| vnz_00250 | cold shock domain protein CspD | 0.421 | 0.005188983 |
| vnz_22545 | RNA polymerase subunit sigma-70 | 0.42 | 0.015669602 |
| vnz_34365 | PIG-L domain-containing protein | 0.417 | 0.014932656 |
| vnz_33670 | hypothetical protein | 0.417 | 0.004577858 |
| vnz_04545 | iron-sulfur protein | 0.412 | 0.035808902 |
| vnz_17240 | glyoxalase | 0.406 | 0.028116256 |
| vnz_08070 | bifunctional metallophosphatase/5'-nucleotidase | 0.405 | 0.002901408 |
| vnz_14685 | aminoacyl-tRNA deacylase | 0.403 | 0.020085408 |
| vnz_24375 | hypothetical protein | 0.395 | 0.02068525 |
| vnz_18375 | hypothetical protein | 0.393 | 0.006275862 |
| vnz_13260 | type VII secretion-associated serine protease mycosin | 0.384 | 0.006280513 |
| vnz_30760 | ABC transporter | 0.384 | 0.013344194 |
| vnz_27580 | enoyl-CoA hydratase | 0.379 | 0.004475855 |
| vnz_34820 | amidohydrolase | 0.374 | 0.00320885 |
| vnz_18545 | antibiotic biosynthesis monooxygenase | 0.374 | 0.002848269 |
| vnz_29925 | hypothetical protein | 0.37 | 0.006934059 |
| vnz_27575 | 3-hydroxyisobutyrate dehydrogenase | 0.369 | 0.003790051 |
| vnz_16125 | aldehyde dehydrogenase | 0.369 | 0.000589519 |
| vnz_06580 | ATPase | 0.369 | 0.017121575 |
| vnz_21085 | NADH-quinone oxidoreductase subunit G | 0.368 | 0.00348862 |
| vnz_13875 | diaminopimelate decarboxylase | 0.365 | 0.004480846 |
| vnz_12555 | methylcrotonoyl-CoA carboxylase | 0.359 | 0.001575895 |
| vnz_07820 | hypothetical protein | 0.355 | 0.005058644 |
| vnz_19250 | phosphate ABC transporter ATP-binding protein | 0.351 | 0.001246694 |
| vnz_05670 | GNAT family N-acetyltransferase | 0.348 | 0.000208789 |
| vnz_27115 | repressor LexA | 0.347 | 0.00112215 |
| vnz_30335 | SARP family transcriptional regulator | 0.347 | 0.021466064 |
| vnz_17465 | hypothetical protein | 0.336 | 0.02064424 |
| vnz_27945 | ATP-dependent endonuclease | 0.334 | 0.002162728 |
| vnz_26840 | hypothetical protein | 0.333 | 0.0063528 |
| vnz_10030 | methyltransferase | 0.332 | 0.0005141 |
| vnz_11305 | peptidase M4 family protein | 0.331 | 0.030376654 |
| vnz_09630 | 3-oxoacyl-ACP reductase | 0.331 | 0.021840132 |
| vnz_35035 | Crp/Fnr family transcriptional regulator | 0.33 | 7.3693E-05 |
| vnz_33885 | dienelactone hydrolase | 0.33 | 0.028066055 |
| vnz_32450 | methylmalonyl-CoA mutase | 0.329 | 0.001060796 |
| vnz_16250 | hypothetical protein | 0.329 | 0.000367857 |
| vnz_31915 | dynein regulation protein LC7 | 0.328 | 0.002641086 |
| vnz_11985 | hypothetical protein | 0.324 | 0.044537006 |
| vnz_09585 | iron transporter | 0.323 | 0.010018314 |
| vnz_21060 | NADH dehydrogenase | 0.319 | 0.010121629 |
| vnz_28750 | hypothetical protein | 0.315 | 9.62606E-05 |
| vnz_27645 | hypothetical protein | 0.314 | 0.000180096 |
| vnz_23005 | hypothetical protein | 0.313 | 0.023875111 |
| vnz_14890 | UTP--glucose-1-phosphate uridylyltransferase | 0.313 | 2.67171E-05 |
| vnz_01565 | hypothetical protein | 0.312 | 0.029005707 |
| vnz_12560 | acetyl/propionyl-CoA carboxylase subuit alpha | 0.31 | 0.000102979 |
| vnz_25230 | glycogen phosphorylase | 0.31 | 0.000122856 |
| vnz_37030 | spore coat protein | 0.309 | 0.025359596 |
| vnz_21075 | NADH-quinone oxidoreductase subunit E | 0.306 | 0.0155288 |
| vnz_21410 | epimerase | 0.306 | 0.024085581 |
| vnz_23110 | Flp pilus assembly protein CpaB | 0.305 | 0.010957996 |
| vnz_22550 | hypothetical protein | 0.304 | 0.000518508 |
| vnz_34770 | methionyl-tRNA formyltransferase | 0.304 | 1.42284E-05 |
| vnz_34845 | N-acetyl-gamma-glutamyl-phosphate reductase | 0.304 | 7.24351E-05 |
| vnz_01260 | phosphatase | 0.304 | 0.029045578 |
| vnz_21095 | NADH-quinone oxidoreductase subunit I | 0.301 | 0.000333232 |
| vnz_24590 | GNAT family N-acetyltransferase | 0.298 | 5.89992E-05 |
| vnz_18640 | hypothetical protein | 0.295 | 9.61365E-05 |
| vnz_04050 | hypothetical protein | 0.292 | 0.001513254 |
| vnz_32680 | dihydrodipicolinate synthase family protein | 0.291 | 0.016718589 |
| vnz_16130 | transcriptional regulator | 0.291 | 5.92109E-05 |
| vnz_35110 | hypothetical protein | 0.29 | 0.015509222 |
| vnz_14410 | peptide ABC transporter ATP-binding protein | 0.289 | 0.011503561 |
| vnz_05680 | glycerophosphodiester phosphodiesterase | 0.286 | 0.00015989 |
| vnz_25635 | phosphodiesterase | 0.284 | 0.010236105 |
| vnz_11590 | hypothetical protein | 0.284 | 3.63273E-05 |
| vnz_25280 | hypothetical protein | 0.284 | 0.004552847 |
| vnz_30010 | AMP-dependent synthetase | 0.282 | 2.71344E-06 |
| vnz_20715 | hypothetical protein | 0.282 | 0.010913407 |
| vnz_24295 | N-acetylmuramoyl-L-alanine amidase | 0.277 | 0.000102648 |
| vnz_08290 | hypothetical protein | 0.276 | 1.11526E-05 |
| vnz_27095 | iron transporter | 0.271 | 0.00494467 |
| vnz_00400 | mini-circle protein | 0.269 | 5.23589E-05 |
| vnz_19085 | MFS transporter | 0.267 | 5.1426E-05 |
| vnz_07150 | hypothetical protein | 0.265 | 4.6127E-05 |
| vnz_13270 | hypothetical protein | 0.265 | 0.002216271 |
| vnz_07165 | cation acetate symporter | 0.264 | 0.005136297 |
| vnz_15810 | hypothetical protein | 0.263 | 0.004955864 |
| vnz_18310 | hypothetical protein | 0.26 | 0.004604768 |
| vnz_16245 | hypothetical protein | 0.255 | 6.0964E-06 |
| vnz_19335 | DNA-binding response regulator, CssR | 0.255 | 6.94139E-06 |
| vnz_09575 | peptidase M75 | 0.255 | 2.64839E-07 |
| vnz_19320 | hypothetical protein | 0.251 | 1.6943E-05 |
| vnz_02660 | hypothetical protein | 0.251 | 2.77538E-05 |
| vnz_34840 | bifunctional ornithine acetyltransferase/N-acetylglutamate synthase | 0.248 | 1.30397E-07 |
| vnz_12565 | hydroxymethylglutaryl-CoA lyase | 0.243 | 6.20887E-06 |
| vnz_32470 | DUF2470 domain-containing protein | 0.243 | 0.000262121 |
| vnz_02645 | ATP-binding protein | 0.241 | 5.7874E-06 |
| vnz_09560 | biliverdin-producing heme oxygenase | 0.24 | 5.75403E-08 |
| vnz_16710 | short-chain dehydrogenase/reductase | 0.237 | 4.23697E-08 |
| vnz_12285 | potassium-transporting ATPase subunit A | 0.236 | 0.001561163 |
| vnz_12480 | aminopeptidase | 0.236 | 3.01268E-06 |
| vnz_14340 | hypothetical protein | 0.235 | 1.37998E-06 |
| vnz_13465 | peptidase | 0.234 | 6.56898E-07 |
| vnz_11185 | ArsR family transcriptional regulator | 0.233 | 0.000308124 |
| vnz_12570 | acyl-CoA dehydrogenase | 0.231 | 4.13654E-07 |
| vnz_12205 | hypothetical protein | 0.224 | 8.71798E-09 |
| vnz_25290 | hypothetical protein | 0.224 | 5.89992E-05 |
| vnz_29910 | hypothetical protein | 0.221 | 0.000655828 |
| vnz_23115 | septum formation initiator | 0.217 | 8.72602E-07 |
| vnz_34785 | non-ribosomal peptide synthetase | 0.214 | 2.62668E-09 |
| vnz_34830 | acetylornithine aminotransferase | 0.214 | 0.000212302 |
| vnz_36480 | YfcE family phosphodiesterase | 0.21 | 0.000537407 |
| vnz_34720 | hypothetical protein | 0.208 | 1.70906E-05 |
| vnz_19330 | two-component sensor histidine kinase, CssS | 0.207 | 3.77722E-06 |
| vnz_37170 | hypothetical protein | 0.207 | 2.59524E-07 |
| vnz_13900 | hypothetical protein | 0.206 | 9.02134E-10 |
| vnz_18830 | phosphoribosylamine--glycine ligase | 0.206 | 7.89995E-06 |
| vnz_35220 | alkyl/aryl-sulfatase | 0.205 | 0.000247396 |
| vnz_16155 | hypothetical protein | 0.202 | 1.40436E-05 |
| vnz_13370 | peptidase S41 | 0.2 | 2.6817E-07 |
| vnz_34700 | hypothetical protein | 0.196 | 0.000132971 |
| vnz_27205 | GntR family transcriptional regulator | 0.196 | 0.0001069 |
| vnz_22480 | transferase | 0.194 | 0.000102974 |
| vnz_14405 | hypothetical protein | 0.194 | 7.18849E-06 |
| vnz_11990 | sugar-binding protein | 0.194 | 0.000140046 |
| vnz_01985 | oxidoreductase | 0.188 | 7.79621E-05 |
| vnz_26885 | glutamate ABC transporter ATP-binding protein | 0.187 | 5.43359E-11 |
| vnz_26880 | glutamate-binding protein | 0.185 | 3.90051E-11 |
| vnz_29085 | hypothetical protein | 0.185 | 2.32932E-06 |
| vnz_31930 | histidine kinase | 0.183 | 3.86665E-05 |
| vnz_11750 | hypothetical protein | 0.179 | 1.3247E-11 |
| vnz_12280 | potassium-transporting ATPase subunit B | 0.179 | 1.37162E-09 |
| vnz_23030 | hypothetical protein | 0.177 | 2.37232E-05 |
| vnz_22005 | DNA-binding response regulator | 0.176 | 1.65993E-08 |
| vnz_27105 | IucA/IucC family protein | 0.175 | 2.41945E-09 |
| vnz_09580 | peroxidase | 0.175 | 7.0528E-12 |
| vnz_24915 | hypothetical protein | 0.172 | 7.63294E-12 |
| vnz_18555 | DNA-binding response regulator | 0.171 | 3.57894E-09 |
| vnz_25605 | hypothetical protein | 0.163 | 1.12308E-10 |
| vnz_12275 | potassium-transporting ATPase subunit C | 0.162 | 9.82352E-08 |
| vnz_09100 | regulator | 0.162 | 1.39594E-05 |
| vnz_23160 | hypothetical protein | 0.158 | 1.75744E-10 |
| vnz_34690 | branched-chain amino acid aminotransferase | 0.155 | 9.55313E-12 |
| vnz_10425 | hypothetical protein | 0.153 | 1.30397E-07 |
| vnz_34865 | hypothetical protein | 0.15 | 2.71344E-06 |
| vnz_23130 | secretion protein | 0.148 | 1.74354E-06 |
| vnz_34695 | ligase | 0.148 | 2.10816E-06 |
| vnz_36840 | hypothetical protein | 0.145 | 1.52689E-06 |
| vnz_35455 | oxidoreductase | 0.143 | 1.53248E-06 |
| vnz_34775 | L-lysine 6-monooxygenase | 0.142 | 1.25545E-15 |
| vnz_34710 | bacilysin biosynthesis protein BacA | 0.138 | 8.79081E-07 |
| vnz_01780 | hypothetical protein | 0.138 | 7.74679E-12 |
| vnz_11480 | MFS transporter | 0.137 | 9.5906E-07 |
| vnz_23860 | reductase | 0.129 | 1.51518E-12 |
| vnz_23450 | GntR family transcriptional regulator | 0.122 | 1.25545E-15 |
| vnz_34680 | radical SAM protein | 0.113 | 1.25545E-15 |
| vnz_30865 | hypothetical protein | 0.109 | 1.25545E-15 |
| vnz_34790 | MbtH family protein | 0.108 | 1.25545E-15 |
| vnz_16495 | WhiB family transcriptional regulator | 0.108 | 8.23933E-10 |
| vnz_26105 | hypothetical protein | 0.105 | 3.73633E-09 |
| vnz_36560 | serine protease | 0.104 | 1.25545E-15 |
| vnz_06785 | hypothetical protein | 0.102 | 1.63454E-09 |
| vnz_08080 | aspartate aminotransferase family protein | 0.098 | 1.60769E-11 |
| vnz_35045 | SAM-dependent methyltransferase | 0.091 | 1.25545E-15 |
| vnz_15140 | transcriptional regulator | 0.09 | 1.25545E-15 |
| vnz_23155 | hypothetical protein | 0.087 | 1.25545E-15 |
| vnz_26875 | glutamate ABC transporter permease | 0.083 | 7.30856E-11 |
| vnz_15655 | RNA polymerase subunit sigma-24 | 0.083 | 1.25545E-15 |
| vnz_26870 | amino acid ABC transporter permease | 0.081 | 1.25545E-15 |
| vnz_31740 | ATP-binding protein | 0.078 | 1.25545E-15 |
| vnz_28820 | DNA-binding response regulator | 0.078 | 1.96181E-11 |
| vnz_13895 | diacylglyceryl transferase | 0.069 | 1.70844E-12 |
| vnz_18230 | LacI family transcriptional regulator | 0.062 | 1.25545E-15 |
| vnz_02655 | dynein regulation protein LC7 | 0.059 | 1.25545E-15 |
| vnz_35830 | hypothetical protein | 0.058 | 1.25545E-15 |
| vnz_22485 | N-acetylneuraminate synthase | 0.057 | 1.25545E-15 |
| vnz_28050 | cation/H(+) antiporter | 0.056 | 1.25545E-15 |
| vnz_01265 | hypothetical protein | 0.056 | 1.25545E-15 |
| vnz_18160 | flavohemoprotein | 0.056 | 1.25545E-15 |
| vnz_22680 | DeoR family transcriptional regulator | 0.055 | 1.25545E-15 |
| vnz_02640 | cytochrome | 0.054 | 1.25545E-15 |
| vnz_19340 | serine protease | 0.053 | 1.25545E-15 |
| vnz_08075 | lysine 6-monooxygenase | 0.045 | 1.25545E-15 |
| vnz_02650 | hypothetical protein | 0.037 | 1.25545E-15 |
| vnz_01515 | protein phosphatase | 0.033 | 1.25545E-15 |
| vnz_07365 | hypothetical protein | 0.031 | 1.25545E-15 |
| vnz_22475 | hypothetical protein | 0.023 | 1.25545E-15 |
