# Supplementary material for: The conserved two-component systems CutRS and CssRS control the protein secretion stress response in Streptomyces
Source: mBio. 2025 Dec 15;17(1):e02991-25. doi: 10.1128/mbio.02991-25 (PMC12802291; doi:10.1128/mbio.02991-25)
Supplement: Table S4 — Day 9 timepoint TMT-proteomics data. [file mbio.02991-25-s0009.docx]

**Supplementary Table 4.** The significant (Adj. P-value) changes between the *S. venezuelae* wild-type (WT) and ∆*cutRS* strains on YPD agar at the Day 9 timepoint detected by TMT-proteomics. The abundance of CutRS in the wild-type samples appears exaggerated due to the data processing against the ∆cutRS samples and does not represent normal levels.

| **Accession** | **Description** | **Abundance WT vs. ∆*cutRS*** | **Adj. P-Value** |
| --- | --- | --- | --- |
| vnz_27390 | DNA-binding response regulator, CutR | 63.336 | 0.002361516 |
| vnz_27285 | cell wall endopeptidase | 53.007 | 0.017552891 |
| vnz_16915 | hypothetical protein | 28.241 | 0.030213951 |
| vnz_24810 | ATP synthase F0 subunit A | 0.054 | 0.044561017 |
| vnz_23525 | peptide ABC transporter substrate-binding protein | 0.053 | 0.043465645 |
| vnz_00640 | catalase | 0.05 | 0.039255361 |
| vnz_14925 | mechanosensitive ion channel protein MscL | 0.048 | 0.026796443 |
| vnz_24215 | sugar transporter | 0.047 | 0.043465645 |
| vnz_08025 | branched chain amino acid ABC transporter substrate-binding protein | 0.044 | 0.025108802 |
| vnz_27580 | enoyl-CoA hydratase | 0.044 | 0.047147118 |
| vnz_05730 | bifunctional ornithine acetyltransferase/N-acetylglutamate synthase | 0.043 | 0.020567337 |
| vnz_05735 | N-acetyl-gamma-glutamyl-phosphate reductase | 0.042 | 0.043465645 |
| vnz_23020 | phosphoenolpyruvate carboxykinase | 0.04 | 0.018726254 |
| vnz_12675 | aspartate 4-decarboxylase | 0.039 | 0.017017972 |
| vnz_24945 | multidrug ABC transporter ATP-binding protein | 0.039 | 0.026796443 |
| vnz_27560 | methylmalonate-semialdehyde dehydrogenase (acylating) | 0.037 | 0.01596251 |
| vnz_24035 | catalase | 0.03 | 0.007835833 |
| vnz_34780 | iron ABC transporter substrate-binding protein | 0.029 | 0.007835833 |
| vnz_13155 | 4-hydroxyphenylpyruvate dioxygenase | 0.027 | 0.006437726 |
| vnz_30445 | acyltransferase | 0.027 | 0.026158785 |
| vnz_09285 | sugar ABC transporter substrate-binding protein | 0.026 | 0.005338942 |
| vnz_30090 | urate oxidase | 0.026 | 0.013715794 |
| vnz_27615 | methylisocitrate lyase | 0.026 | 0.043465645 |
| vnz_23560 | ABC transporter permease | 0.026 | 0.047390913 |
| vnz_21120 | NADH-quinone oxidoreductase subunit N | 0.025 | 0.043465645 |
| vnz_28530 | flotillin | 0.024 | 0.004301692 |
| vnz_28685 | enoyl-CoA hydratase | 0.024 | 0.02679309 |
| vnz_21110 | NADH-quinone oxidoreductase subunit L | 0.024 | 0.02948506 |
| vnz_09475 | amino acid:proton antiporter | 0.023 | 0.007233281 |
| vnz_21090 | NADH-quinone oxidoreductase subunit H | 0.023 | 0.019885021 |
| vnz_06645 | DUF3533 domain-containing protein | 0.023 | 0.030277992 |
| vnz_12865 | FAD-dependent oxidoreductase | 0.023 | 0.044561017 |
| vnz_02335 | MFS transporter | 0.022 | 0.043465645 |
| vnz_12895 | peptidase | 0.021 | 0.01596251 |
| vnz_02440 | ABC transporter | 0.021 | 0.017552891 |
| vnz_35040 | 2-methylisoborneol synthase | 0.02 | 0.007233281 |
| vnz_24300 | Crp/Fnr family transcriptional regulator | 0.018 | 0.001118719 |
| vnz_23575 | peptide ABC transporter ATP-binding protein | 0.018 | 0.013593771 |
| vnz_19260 | phosphate ABC transporter permease subunit PstC | 0.018 | 0.013715794 |
| vnz_34370 | hydroxylase | 0.017 | 0.001118719 |
| vnz_13130 | hypothetical protein | 0.017 | 0.017552891 |
| vnz_02650 | hypothetical protein | 0.016 | 0.00268772 |
| vnz_19255 | phosphate ABC transporter, permease protein PstA | 0.016 | 0.010945827 |
| vnz_23555 | peptide ABC transporter substrate-binding protein | 0.015 | 0.000652177 |
| vnz_10905 | MFS transporter | 0.015 | 0.011832784 |
| vnz_19265 | phosphate ABC transporter substrate-binding protein PstS | 0.014 | 0.000421117 |
| vnz_22480 | transferase | 0.014 | 0.008266524 |
| vnz_33520 | protein in whiE locus | 0.013 | 0.008266524 |
| vnz_16085 | hypothetical protein | 0.013 | 0.012001349 |
| vnz_35020 | hypothetical protein | 0.012 | 0.005338942 |
| vnz_35035 | Crp/Fnr family transcriptional regulator | 0.01 | 0.000105493 |
| vnz_25590 | phospholipase C, phosphocholine-specific | 0.009 | 4.58296E-05 |
| vnz_13030 | PTS lactose transporter subunit IIC | 0.008 | 0.000421117 |
| vnz_35890 | hypothetical protein | 0.008 | 0.002202744 |
| vnz_07365 | hypothetical protein | 0.006 | 5.07335E-06 |
| vnz_17470 | phosphatase | 0.004 | 1.36324E-06 |
| vnz_22475 | hypothetical protein | 0.002 | 1.91977E-05 |
